# Changes in Biomass and Diversity of Soil Macrofauna along a Climatic Gradient in European Boreal Forests

**DOI:** 10.3390/insects13010094

**Published:** 2022-01-14

**Authors:** Mikhail V. Kozlov, Vitali Zverev, Vladimir I. Gusarov, Daniil I. Korobushkin, Nina P. Krivosheina, Jaakko Mattila, Marko Mutanen, Anna Popova, Alexander S. Prosvirov, Pekka Punttila, Guy Söderman, Marzena Stańska, Astrid Taylor, Varpu Vahtera, Natalia A. Zubrii, Elena L. Zvereva

**Affiliations:** 1 Department of Biology, University of Turku, 20014 Turku, Finland; vitzve@utu.fi (V.Z.); elezve@utu.fi (E.L.Z.); 2 Natural History Museum, University of Oslo, 0562 Oslo, Norway; vladimir.gusarov@nhm.uio.no; 3 A.N. Severtsov Institute of Ecology and Evolution, Russian Academy of Sciences, 119071 Moscow, Russia; dkorobushkin@yandex.ru (D.I.K.); dipteranina@rambler.ru (N.P.K.); velja220@mail.ru (A.P.); 4 Finnish Museum of Natural History, University of Helsinki, 00014 Helsinki, Finland; jaakko.mattila@helsinki.fi; 5 Ecology and Genetics Research Unit, University of Oulu, 90014 Oulu, Finland; Marko.Mutanen@oulu.fi; 6 Department of Entomology, Faculty of Biology, Moscow State University, 119234 Moscow, Russia; carrabus69@mail.ru; 7 Biodiversity Centre, Finnish Environment Institute (SYKE), 00790 Helsinki, Finland; pekka.punttila@syke.fi; 8 Finnish Entomological Society c/o Finnish Museum of Natural History, University of Helsinki, 00014 Helsinki, Finland; guy.soderman@pp.inet.fi; 9 Institute of Biological Sciences, Faculty of Sciences, Siedlce University of Natural Sciences and Humanities, 08-110 Siedlce, Poland; marzena.stanska@uph.edu.pl; 10 Department of Ecology, Swedish University of Agricultural Sciences, 756 51 Uppsala, Sweden; Astrid.Taylor@slu.se; 11 Biodiversity Unit, University of Turku, 20014 Turku, Finland; varpu.vahtera@utu.fi; 12 N. Laverov Federal Center for Integrated Arctic Research, Ural Branch of the Russian Academy of Sciences, 163000 Arkhangelsk, Russia; 9052930111@mail.ru

**Keywords:** climate change, biomass, biotic interactions, diversity, environmental gradient, feeding guilds, latitudinal variation, macroecology, macrofauna

## Abstract

**Simple Summary:**

We used a 1000 km long latitudinal gradient in north-western Russia to study the potential impacts of a changing climate on soil invertebrates visible by a naked eye (insects, spiders, earthworms etc.). We extracted these animals from soil, weighed them and identified them to the species level. We found that the diversity of soil invertebrates decreased towards the north, whereas the latitudinal pattern in biomass depended on the animal’s feeding habit. The biomass of species feeding on live plant roots and fungal mycelia decreased towards the north, whereas the biomass of species feeding on dead plant tissues and live invertebrates showed no significant latitudinal changes. The discovery of this variation in latitudinal biomass patterns suggests that soil invertebrates from different feeding guilds may respond differently to climate change. As a result, the biomass ratio between consumers and their food resources (e.g., herbivores and plants, predators and prey) may change. We poorly understood how this change will affect the future structure and functions of boreal forest ecosystems.

**Abstract:**

Latitudinal gradients allow insights into the factors that shape ecosystem structure and delimit ecosystem processes, particularly climate. We asked whether the biomass and diversity of soil macrofauna in boreal forests change systematically along a latitudinal gradient spanning from 60° N to 69° N. Invertebrates (3697 individuals) were extracted from 400 soil samples (20 × 20 cm, 30 cm depth) collected at ten sites in 2015–2016 and then weighed and identified. We discovered 265 species living in soil and on the soil surface; their average density was 0.486 g d·w·m^−2^. The species-level diversity decreased from low to high latitudes. The biomass of soil macrofauna showed no latitudinal changes in early summer but decreased towards the north in late summer. This variation among study sites was associated with the decrease in mean annual temperature by ca 5 °C and with variation in fine root biomass. The biomass of herbivores and fungivores decreased towards the north, whereas the biomass of detritivores and predators showed no significant latitudinal changes. This variation in latitudinal biomass patterns among the soil macrofauna feeding guilds suggests that these guilds may respond differently to climate change, with poorly understood consequences for ecosystem structure and functions.

## 1. Introduction

Soil is the critical and dynamic regulatory centre for the majority of processes occurring in both natural and managed terrestrial ecosystems [1]. Biota forms a vital part of soil, and some scientists argue that the extent and importance of life in the soil define soil as living medium [2]. Enormous numbers of microorganisms, including bacteria and fungi, as well as larger organisms that range from nematodes to moles, play essential roles in soil processes and functions, including nutrient cycling, decomposition and bioturbation, and maintain provision of ecosystem services [3,4]. Nevertheless, belowground structures, functions and processes are among the most poorly understood areas in ecology [5], even though the understanding of the complex interplay between climate and interactions among soil organisms is essential for a full anticipation of how terrestrial ecosystems will respond to climate change and other disturbances [6,7].

Experimental studies addressing impacts of abiotic drivers of global change on soil biota remain logistically challenging and therefore scarce. The accumulated knowledge pertains mostly to the effects of temperature, precipitation and CO_2_ elevation on the abundance of different groups of soil organisms [8]. The findings of these studies, which are often limited to a certain taxon and which manipulate only one of many co-occurring environmental factors, are difficult to use as predictors of the fate of soil biota in natural ecosystems as the climate warms. Another approach to addressing climate change impacts on soil biota is offered by macroecology, which aims at the development of quantitative predictions on the abundance and distribution of large numbers of species, usually over broad areas [9].

Climate is the primary driver of latitudinal/altitudinal patterns in biotic interactions, and geographical gradients have recently been promoted as natural laboratories for studying the potential impacts of a changing climate on terrestrial organisms at immediately relevant spatial scales [10]. However, the majority of studies exploring latitudinal patterns in terrestrial ecosystems are limited to aboveground subsystems, and the search of publications for the recent meta-analysis [11] has not discovered any data on herbivory or carnivory in soil communities. The empirical data obtained for the belowground subsystems (with the exception of microbiota) are scarce and often insufficient for reliable generalisations, especially at large spatial scales (but see [12,13,14]). As a result, little is known about the environmental factors that shape soil animal communities [15]. In particular, no clear relationships have been discovered thus far between latitude and belowground species richness [16], and the existence of a general latitudinal pattern in the biomass of soil communities remains debatable [12,17].

In this study, we explored latitudinal patterns in biomass and in species diversity of soil macrofauna (defined as invertebrates over 2 mm in length [18]) in boreal forests of northern Europe. We selected macrofauna for this study for three reasons. First, some of these organisms, primarily earthworms and ants, play a key role in regulating the physical, chemical and microbiological properties of soils [19]. Second, macrofauna includes detritivores, herbivores, fungivores and predators [20], thereby allowing the simultaneous exploration of latitudinal changes at multiple trophic levels of soil biota. This opportunity is of particular importance in the face of climate warming, because differential sensitivities of feeding guilds to environmental factors may lead to their differential responses to climate change and thus to an inevitable alteration of the interactions between trophic levels [21,22]. Third, experimental studies on the impacts of abiotic drivers of global change on soil macrofauna are scarce [8], and data on their biomass (with the exception of earthworms) in natural ecosystems across biomes are in short supply [12,23].

The current level of knowledge regarding the responses of soil biota to different abiotic and biotic factors does not allow prediction of even the direction of latitudinal changes in soil faunal biomass within the boreal forest zone. If soil macrofauna, like many ectothermic organisms [24], is limited by ambient temperatures or controlled by net primary production [25], then this biomass should decrease from low to high latitudes, following the concerted decreases in temperature, plant biomass and productivity [10,26,27]. If, however, the majority of soil invertebrates rely profoundly on the carbon inputs from plant roots, then the soil faunal biomass in our latitudinal gradient should not change, given the absence of latitudinal changes in fine root biomass [28]. Finally, if macrofaunal biomass is primarily controlled by soil microbial carbon and nitrogen stocks, which reach the highest values in northern high latitudes [29], then the biomass of soil macrofauna should increase with an increase in latitude.

We asked (1) whether the biomass and diversity of soil macrofauna change with latitude, (2) which factors best explain these latitudinal changes, and (3) whether latitudinal patterns in biomass and diversity are similar among feeding guilds of soil macrofauna and are consistent between the adjacent trophic levels. To answer these questions, we studied soil macrofauna in ten unmanaged coniferous forest sites along a 1000 km long latitudinal gradient in north-western Russia.

## 2. Materials and Methods

### 2.1. Study Sites

**Figure 1:**
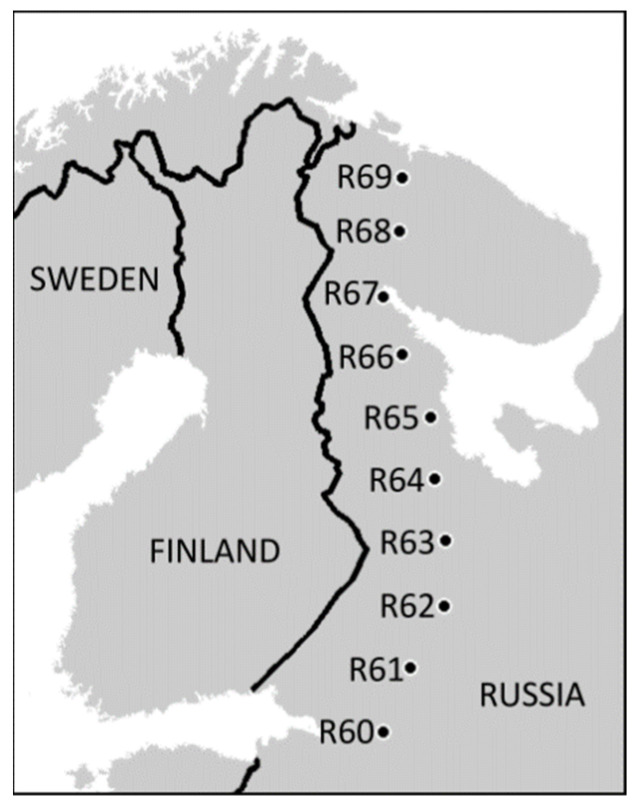
Locations of the study sites (reproduced with permission from [28]). For detailed information on study sites consult Tables S1 and S2 in the Supplementary Materials.

Our latitudinal gradient spans from the northern limit of broadleaved forests at 60° N near St. Petersburg to the northern tree line at 69° N close to Murmansk, Russia (Figure 1, Table S1 in Supporting information). The ten forested sites were selected in 2008 as being closest to the rounded degrees of latitude along the road connecting the above-mentioned cities. The selection occurred without any a priori knowledge of the soil characteristics. All sites are located in uneven-aged, unmanaged, old-growth boreal forests (maximum site-specific tree age ranging 50 to 300 years) consisting of Scots pine (*Pinus sylvestris*), birches (common *Betula pubescens* and rare *B. pendula*), Norway spruce (*Picea abies*), European aspen (*Populus tremula*) and goat willow (*Salix caprea*). The field layer vegetation was dominated by dwarf shrubs (primarily *Vaccinium myrtillus* and *V. vitis-idaea*), except for the northernmost site, where the herb *Cornus suecica* predominated.

The basal area of the tree stands was measured using a relascope at five haphazardly selected points, and the cover of field layer vegetation was evaluated visually in 10 systematically selected 1 × 1 m plots. The plant species diversity was quantified by the ln-based Shannon H diversity index from these individual measurements (i.e., from numbers of trees of different species and from percentages of the cover of field layer species) and then averaged for site-specific values (Table S1). The fine root biomass was measured from samples (40 per site) collected simultaneously with samples for study of soil macrofauna using a cylindrical metal corer (for more details consult [28]). The long-term (1990–2019) mean annual air temperature (Table S2) decreased by ca. 5°C from 60° N to 69° N (*r* = −0.97, *n* = 10 sites, *p* < 0.0001), whereas mean annual precipitation (Table S2) did not change with latitude (*r* = −0.53, *n* = 10 sites, *p* = 0.11).

The soils in all study sites (identified according to [30]) were gleyic albic podzols and folic albic podzols formed on sandy material. Soil characteristics (listed in Table S2) were measured from samples collected on 20–22 August 2014; for the description of methods, consult [28]. The thickness of the organic layer decreased, whereas the volumetric stone content of the mineral soil increased towards the north. The remaining soil characteristics (moisture, pH and concentrations of nitrogen, phosphorus and potassium) generally did not change with latitude [28].

### 2.2. Collection and Identification of Invertebrates

We slightly modified the method developed by Gilyarov [31], which is widely used in soil zoology. Soil for extraction of macrofauna was excavated to a 30 cm depth from a 20 × 20 cm area. A total of 400 samples of this volume were collected during four sampling sessions arranged in the last weeks of June and August of both 2015 and 2016. The size and number of samples were selected to balance the probability of capturing rare animals and the technical feasibility of the project. At each site, 40 samples were collected within a plot 100 × 100 m in size along the pre-defined lines (different for each of four sampling sessions); the distances between the neighbouring samples were 5–10 m. In 2015, we collected 10 samples of this size from each site during each of the two sampling periods. Processing of these samples showed that soil mineral horizons contained far fewer invertebrates than were found in the humus layer. Therefore, in 2016, we collected mineral soil up to a 30 cm depth only from five of ten samples per site. From the remaining five samples, we collected only the organic soil layer, which was 6–12 cm in depth. The samples were transported to the laboratory and hand-sorted in a haphazard order within 5 days from the collection date; all animals were alive at the time of sorting. Soil was manually broken into small pieces and sieved through a 4 mm grid; all discovered invertebrates were stored in 70% ethanol. The same persons processed the samples from different localities, so that potential differences in the efficiency of individual collectors could not bias our results.

Most of invertebrates were identified based on their morphological characters. The responsibilities of the co-authors were as follows: V.I.G., adult rove beetles; N.A.Z., ground beetles and their larvae; A.S.P., larvae of click beetles; G.S., Hemiptera and some beetles; J.M., the remaining beetles; D.I.K. and V.V., millipedes and centipedes; N.P.K., fly larvae; P.P., ants; M.S., spiders; A.T., earthworms. Voucher specimens are deposited in Finnish Museum of Natural History, University of Helsinki, Finland; Zoological Museum, University of Oulu, Finland; Institute of Biological Sciences, Faculty of Sciences, Siedlce University of Natural Sciences and Humanities, Poland; Natural History Museum, University of Oslo, Norway; Department of Entomology, Faculty of Biology, Moscow State University, Moscow, Russia; and Russian Museum of Biodiversity Hotspots, Arkhangelsk, Russia.

Tissue samples collected from 329 specimens that could not be identified based on morphological characters (mostly insect larvae) were submitted to the Canadian Centre for DNA Barcoding (Guelph, ON, Canada). The sequencing was carried out using standard Sanger-based protocols [32]. We obtained 237 sequences and identified the sequenced specimens using the BOLD database (www.boldsystems.org, accessed on 11 January 2022). The remaining 92 specimens, representing samples that failed to produce sequences suitable for identification, were attributed to species, family or order based on their visual similarity with the successfully sequenced specimens. Sample photographs, collection data and taxonomic information, as well as sequences and GenBank accession numbers of the analysed specimens, can be retrieved through the public dataset at dx.doi.org/10.5883/DS-SOILM (accessed on 11 January 2022). 

### 2.3. Weighing of Invertebrates and Correction for Weight Loss during Preservation

The need to preserve collected specimens for identification and for long-term storage in accessible repositories did not allow direct measurement of their dry weight. We therefore preserved all individuals in ethanol and weighed them to the nearest 0.01 mg at 6–8 weeks after their collection date; this period is sufficient for weight stabilisation [33,34]. The dry weight was calculated by multiplying the wet weight by a taxon-specific correction coefficient that accounted for both weight gain and weight loss due to preservation in ethanol. We calculated these coefficients from data obtained from invertebrates collected in addition to our regular samples and then divided into two subsets. The individuals from the first subset were weighed alive, dried at 105 °C and weighed again, whereas the individuals from the second subset were weighed alive, preserved in 70% ethanol for 6–8 weeks, then weighed again, dried at 105 °C for 24 h and weighed again. Analysis of these weights yielded the following correction coefficients: 0.18 (Chironomidae larvae), 0.24 (ants, beetles, cockroaches, true bugs, millipedes, centipedes and worms) and 0.42 (spiders and larvae of beetles, non-chironomid flies and moths).

### 2.4. Feeding Strategies of Soil Macrofauna

We classified soil macrofauna (Data S1) into four trophic groups [8]: herbivores (feed on live plant tissues, mainly roots), predators (feed on live animals), detritivores (feed on dead plant and/or animal tissues) and fungivores (feed on fungal mycelia). The information on feeding habits of individual species and higher taxa (Table S3) was obtained from multiple data sources, including modern studies that use stable isotope ratios to identify positions of different organisms in soil food webs [20]. For soil invertebrates that feed on two or more types of food, we divided their biomass between two or more feeding guilds proportionally to the contribution of each type of food to their diets. When estimating this contribution for ants, we concentrated on food consumed in soil or on soil surface. Therefore we excluded aphid tending on trees and shrubs, but included below-ground aphid tending. We used the data for *Formica rufa* group [35] as a rough approximation for the ratio between honeydew and prey (4:1) for all ant species that use aphid honeydew. Data on click beetle (Elateridae) larvae are mostly after Dolin [36], with some additions from our previous study [37].

### 2.5. Statistical Analysis

We excluded from our database (Data S1) invertebrate species that do not live in soil or on the soil surface; we also excluded 417 ants collected from one soil sample as a statistical outlier. We found that the average biomass of soil invertebrates did not differ (*F*_1,198_ = 0.004, *p* = 0.95) between the samples collected in 2016 with and without the mineral horizon (mean ± S.E.: 20.46 ± 6.62 mg and 19.96 ± 3.97 mg, respectively). Consequently, we did not apply any correction factor to the data from the 100 samples that were lacking the soil mineral horizon.

We pooled information across 10 samples collected from one site during each of four sampling sessions, and we used these 40 values of biomass and 40 values of diversity in the subsequent analyses. The biomass values were log_10_(x + 0.1) transformed to normalise the distribution of the residuals. Diversity was quantified with the Shannon H index, based on individuals identified to the species or genus level; this index was calculated using the PAST program [38].

Our key research question concerned the latitudinal patterns; therefore, the latitude of the study site was included as a covariate in the linear mixed model ANCOVAs, which explored the sources of variation in the biomass and diversity of the soil macrofauna. In these models, feeding guild (for biomass only), year and month were treated as fixed factors, whereas the study site was treated as random factor. The simultaneous involvement of both site and latitude in our analyses is justified by the fact that our sites differ not only in latitude, but also in a number of other characteristics (plant community structure, in particular). All analyses were carried out with the SAS GLIMMIX procedure [39] with the standard errors and denominator degrees of freedom adjusted as recommended by Kenward and Roger [40]. The significance of the random factor (site) was evaluated by calculating the likelihood ratio and testing it against the chi-squared distribution [41].

We separately conducted six forward stepwise regression analyses to identify the models that best explain the spatial variation in the diversity and biomass of soil macrofauna, as well as in the biomass of each of four feeding guilds. These analyses included 15 explanatory variables: latitude, mean annual temperature, mean annual precipitation, stand basal area, diversity of trees, cover and diversity of field layer vegetation, fine root biomass, soil moisture, thickness of organic soil horizon, the weight percentage of the soil fraction of <2 mm grain size, soil pH, total N in soil and bioavailable P and K in soil (Tables S1 and S2).

## 3. Results

### 3.1. Overall Biomass and Species Richness

In total, we collected 3697 individuals of 265 species of invertebrate macrofauna. Most of the collected invertebrates (Table 1, Data S1) were identified to the species or genus level (2950 and 373 individuals, respectively). We excluded 33 specimens (comprising 0.7% of the total biomass) from the data analysis because they were not living in/on soil but would occasionally fall from the surrounding vegetation.

The biomass of the soil macrofauna in our study region averaged 0.486 ± 0.118 g d·w·m^−2^ and did not differ between the two study years; however, it was slightly greater in late summer than in early summer (Table 2). Detritivores (primarily earthworms) contributed most (36.6%) to the macrofaunal biomass, followed by predators (18.3%), herbivores (15.1%) and fungivores (0.1%). The remaining 28.9% of the biomass was represented by 28 invertebrate species with mixed feeding habits. The predatory macrofauna was most diverse (178 species), followed by detritivores (21 species), herbivores (20 species) and fungivores (8 species).

**Table 1:** Numbers of the collected species and individuals and biomass of major taxonomic groups of soil macrofauna.

Class	Order	Family	No. of Species	No. of Individuals	Biomass, mg d.w.
Clitellata	Haplotaxida	Lumbricidae	4	177	2647.0
Arachnida	Araneae	Hahniidae	2	68	13.1
		Linyphiidae	47	499	165.7
		Lycosidae	4	59	77.2
		Theridiidae	3	219	85.9
		Thomisidae	2	25	41.4
		Other 11 families	16	49	54.1
Diplopoda	Julida	Julidae	1	11	256.2
	Polydesmida	Polydesmidae	1	5	13.0
	Polyzoniida	Polyzonidae	1	3	5.0
Chilopoda	Lithobiomorpha	Lithobiidae	2	222	218.3
Insecta	Blattoptera	Ectobiidae	1	5	7.3
	Hemiptera	Lygaeidae	4	41	36.0
		Other 6 families	6	28	52.1
	Hymenoptera	Formicidae	10	714	313.2
		Pamphiliidae	2	4	109.5
	Coleoptera	Carabidae	14	53	168.7
		Cantharidae	8	94	127.8
		Curculionidae	11	49	283.0
		Elateridae	11	605	2353.9
		Staphylinidae	72	478	292.1
		Other 7 families	13	26	632.0
	Diptera	Chironomidae	3	22	1.0
		Rhagionidae	3	94	223.7
		Other 12 families	21	62	353.6
	Lepidoptera	Hepialidae	2	22	339.2
		Oecophoridae	1	1	2.6

### 3.2. Latitudinal Patterns in Biomass

**Figure 2:**
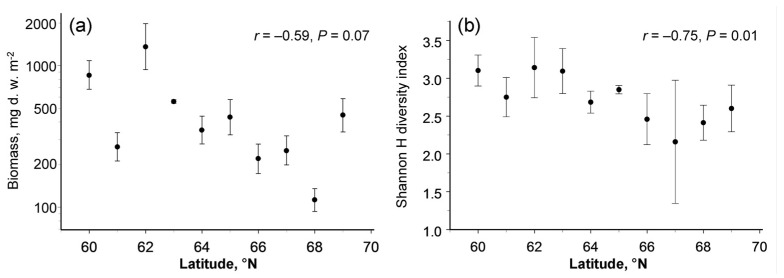
Latitudinal patterns in (**a**) biomass and (**b**) diversity of soil macrofauna (means and 95% confidence intervals, each based on four sampling dates).

The latitudinal pattern in the biomass of the soil macrofauna varied between seasons (latitude × month interaction term in Table 2); the biomass showed no latitudinal changes in early summer (*r* = −0.35, *n* = 10 sites, *p* = 0.33) but decreased towards the north in late summer (*r* = −0.68, *n* = 10 sites, *p* = 0.03). The biomass averaged across the seasons demonstrated a marginally significant (*p* = 0.07) poleward decrease (Figure 2a), which was associated with the decrease in mean annual temperature and with variation in fine root biomass (Table 3).

**Table 2:** Sources of variation in biomass and diversity (measured by Shannon H index) of soil macrofauna (SAS GLIMMIX procedure; mixed model ANCOVA, type III test).

Effect	Explanatory Variable	Biomass		Diversity	
		Test Statistics	*p*	Test Statistics	*p*
Fixed	Latitude	*F*_1,8_ = 4.31	0.07	*F*_1,8_ = 10.58	0.0117
	Year	*F*_1,24_ = 2.65	0.12	*F*_1,24_ = 3.45	0.08
	Month	*F*_1,24_ = 8.83	0.0066	*F*_1,24_ = 0.02	0.88
	Latitude × Year	*F*_1,24_ = 2.80	0.11	*F*_1,24_ = 3.65	0.07
	Latitude × Month	*F*_1,24_ = 8.72	0.0069	*F*_1,24_ = 0.09	0.77
	Year × Month	*F*_1,24_ = 0.08	0.78	*F*_1,24_ = 0.44	0.52
	Latitude × Year × Month	*F*_1,24_ = 0.14	0.71	*F*_1,24_ = 0.58	0.45
Random	Site	*χ^2^*_1_ = 15.74	<0.0001	*χ^2^*_1_ = 2.54	0.06

**Figure 3:**
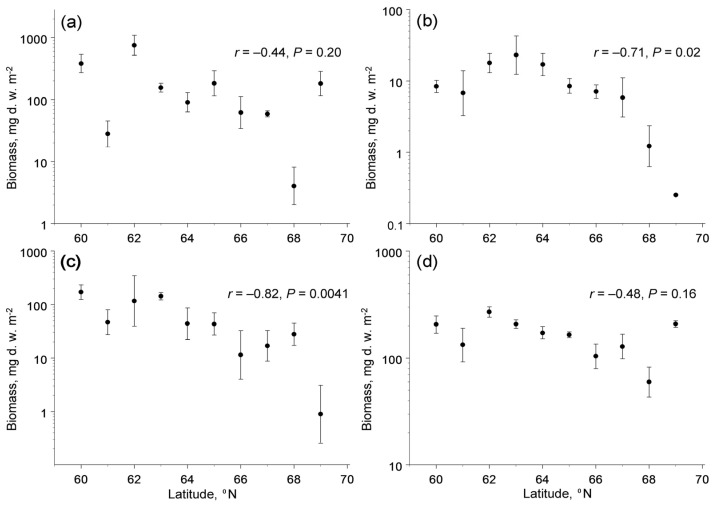
Latitudinal patterns in biomass (mg d.w. m^−2^) of four trophic groups of soil macrofauna (means and 95% confidence intervals, each based on four sampling dates): (**a**) detritivores; (**b**) fungivores; (**c**) herbivores; (**d**) predators. Note that the scales of the vertical axes differ among the panels.

The biomass of the four trophic groups showed different changes with latitude (latitude × feeding habit interaction: *F*_3,133_ = 4.79, *p* = 0.0033). The poleward decrease in biomass was significant in herbivores and fungivores, but not significant in detritivores and predators (Figure 3). The biomass of herbivores and fungivores, in addition to latitude, correlated with concentrations of available potassium in soil, whereas the among-site variation in biomass of detritivores and predators was not explained by climate, soil quality or characteristics of plant communities (Table 3).

### 3.3. Latitudinal Pattern in Diversity

The taxonomic diversity of the soil macrofauna, quantified by the Shannon H index, significantly decreased with an increase in latitude (Table 2; Figure 2b). None of the explanatory variables other than latitude entered the stepwise regression analysis (Table 3).

**Table 3:** Characteristics of the best-fit linear models explaining variation in the diversity (Shannon H index) and biomass of soil macrofauna: the outcomes of the forward stepwise regression analyses. None of the explanatory variables entered the analyses of biomass of detritivores and predators.

Response Variable	Feeding Guild	Explanatory Variable	Slope	Standard Error	*F*	*p*	Partial *R*^2^
Diversity	All combined	Latitude	−0.082	0.025	10.58	0.0117	0.570
Biomass	All combined	Mean annual temperature	0.105	0.040	6.14	0.0382	0.434
		Fine root biomass	−0.00082	0.00034	5.71	0.0482	0.254
	Fungivores	Latitude	−0.126	0.026	8.26	0.0207	0.508
		Potassium in soil	−0.00148	0.00032	20.87	0.0026	0.368
	Herbivores	Latitude	−0.1668	0.025	15.84	0.0041	0.664
		Potassium in soil	−0.00137	0.00031	19.65	0.0030	0.247

## 4. Discussion

### 4.1. Latitudinal Changes in Diversity and Biomass of Soil Macrofauna

In this study, we used observational data within a correlative framework—a common approach when addressing regional- or global-scale geographic patterns [9,11]. We found a poleward decline in the diversity of soil macrofauna along our latitudinal gradient; the latitudinal decline in biomass was observed only in late summer and was limited to herbivores and fungivores. While latitudinal patterns in aboveground communities are well documented [11,26,42,43], our study is likely the first to explore within-biome latitudinal patterns in soil macrofauna based on replicated sampling along a climatic gradient combined with the species-level identities of the collected invertebrates.

Recent studies have suggested that aboveground and belowground biota may demonstrate different biogeographical patterns at the global scale [44,45]. However, within boreal forests we found a poleward decrease in the species-level diversity of soil macrofauna, and this finding agrees with patterns observed in aboveground biota, both in the global studies [43,46] and in the same latitudinal gradient, particularly for leaf miners [47], psocids [48] and spiders [42].

The poleward decrease in biomass of herbivores and fungivores detected in our study is in line with our prediction based on the identified declines in both ambient temperature and net primary production with increasing latitudes [10,26,49]. By contrast, the absence of latitudinal changes in the biomass of detritivores and predators agrees with the latitudinal pattern of fine root biomass [28], although this variable did not enter the stepwise regression analysis. At the same time, our results disagree with the outcomes of the meta-analysis of experimental studies which have demonstrated no significant effects of warming and a positive effect of precipitation on the abundance (and, consequently, the biomass) of all taxa and trophic groups of the soil biota in forest ecosystems [8].

The contradictions between the outcomes of the experimental and observational studies regarding the effects of climatic variables on soil macrofauna may emerge because experimental conditions do not account for either arrival of new species or the development of species’ evolutionary adaptations, and the local processes discovered in manipulative studies are difficult to scale up to the ecosystem or regional level [9]. Therefore, complementing experimental data with the analysis of macroecological patterns may improve our understanding of factors shaping soil communities and, consequently, the accuracy of predictions on how global change may affect soil biota.

The expected increase in ambient air temperatures of 3.5–5.0 °C by the end of the current century [50] is likely to allow a northwards spread of many species of soil macrofauna and an increase in their biomass, although the latter effect may be limited to species feeding on live plant tissues and fungal mycelia. The magnitude of the expected climate warming is similar to the climatic differences between the two ends of our gradient (Table S2). Consequently, the diversity of soil invertebrates in our northernmost study sites may approach the level currently observed at our southernmost sites, whereas their overall biomass will increase to a lesser extent. A further likelihood is that stronger increases in temperature at the more northern regions [50] will make the latitudinal gradient in the diversity and biomass of soil macrofauna shallower relative to its current state.

### 4.2. Latitudinal Changes in Biotic Interactions

The biomass ratio of consumers to producers (e.g., herbivores to plants, predators to prey) reflects the structure of a community and its ecosystem properties, such as nutrient cycling and the energy flow from producers to higher trophic levels [51,52]. Therefore, the biomass data can be used to determine whether the community structure changes along latitudinal [53] or elevational [54] climatic gradients. The existence of data on the annual production of foliage by woody plants [27] and on the biomass of fine roots [28] in our latitudinal gradient, along with data on the aboveground insect herbivory [27] and on predation on leafmining insects [55], offers a unique opportunity to discuss how interactions between trophic levels change with climate in both aboveground and belowground ecosystem compartments.

The biomass of root-feeding insects at the northern end of our latitudinal gradient, as estimated from the linear regression, comprised ca 3% of the biomass at its southern end (Figure 3c), whereas the biomass of fine roots (i.e., the food of root-feeders) did not change with latitude [28]. Assuming that the efficiency of conversion of ingested food by insect herbivores does not change with latitude, and accounting for a 40% decrease in food consumption per unit of the insect body mass due to the decrease in insect metabolic demands with a decrease in ambient temperatures of 5 °C [56], we estimate that the belowground insect herbivory (i.e., the proportion of fine root biomass consumed by insects) decreased nearly 50-fold from 60° N to 69° N. This extreme decrease in the community-wide belowground herbivory clearly contrasts with the absence of latitudinal changes in the community-wide aboveground insect herbivory [27] and is much greater than the 2-fold to 4-fold decrease in aboveground herbivory in deciduous woody plants in the same latitudinal gradient [27]. We attribute this difference to nearly 1.5-fold greater latitudinal decrease in soil temperatures from 60° N to 69° N (data after [57]) relative to air temperatures during the summer.

The latitudinal changes in belowground predation are difficult to evaluate, because we do not have data on the biomass of soil microfauna (which contributes substantial resources for invertebrate predators) and because we cannot quantify the within-guild predation. Nevertheless, the biomass of both predators and non-predatory soil macrofauna (i.e., the food resource for predators) did not change with latitude, implying an absence of substantial latitudinal changes in the belowground invertebrate predation. This result is consistent with the absence of latitudinal changes in the biomass ratio of invertebrate carnivores to insect herbivores in tree canopies in Australia [53], but it contrasts with the 2-fold to 4-fold poleward decrease in bird and ant predation on arboreal herbivores observed in our latitudinal gradient [55].

Finally, the biomass of current-year foliage of common plant species that, after abscission, forms a substantial part of the food resources for detritivores showed a nearly twofold decrease from low to high latitudes in our gradient [27], whereas the biomass of fine roots (which, after their death, also become food for detritivores) did not change with latitude [28]. The average biomass of fine roots in our study plots (843 g d.w. m^−2^: [28]) is much greater than the biomass of the current-year foliage (100–300 g d.w. m^−2^: [27]); therefore, their sum (i.e., the amount of dead plant tissues available to detritivores) shows only a slight poleward decrease. Since the biomass of detritivores did not change significantly within our gradient (Figure 3a), but their metabolic demands and their resulting food consumption decreased towards the north [56], we conclude that the proportion of plant litter consumed by these invertebrates shows little (if any) changes within our climatic gradient. Thus, the contribution of soil macrofauna to the decomposition of plant litter and ecosystem dynamics will likely remain the same as the climate warms.

Overall, our findings indicate that the intensities of individual biotic interactions involving soil macrofauna exhibit different latitudinal patterns: herbivory declined greatly with an increase in latitude, in agreement with the Latitudinal Biotic Interaction Hypothesis [11,58], whereas predation and detritophagy did not show any latitudinal changes. Furthermore, the latitudinal patterns in herbivory and predation differed between belowground and aboveground communities in the same latitudinal gradient. This finding suggests that the mismatches observed between geographical patterns in the characteristics of aboveground and belowground ecosystem compartments [45] are not limited to biodiversity; rather, they may also involve biotic interactions.

## 5. Conclusions

Our data collected from a 1000 km long latitudinal gradient contribute to the general understanding of macroecological patterns in the biomass and diversity of soil macrofauna, although the immediate drivers of several of the observed patterns remain to be identified. Nevertheless, “space-for-time” substitution, which is widely used in ecology to predict future changes in ecosystem structure and functions from contemporary spatial patterns [10,59], suggests that the strength of herbivory, predation and detritophagy in soil food webs involving invertebrate macrofauna will change non-proportionally with respect to each other as the climate warms, with poorly understood consequences for ecosystem structure and functions.

## Data Availability

The data on soil macrofauna are available as electronic Supplementary Materials.

## Supplementary Materials

The following supporting information can be downloaded at: https://www.mdpi.com/article/10.3390/insects13010094/s1, Table S1: Characteristics of study sites and plant communities, Table S2: Characteristics of climate and of soil organic horizon, Table S3: Distribution of soil macrofauna families by trophic groups, Data S1: Characteristics of collected soil invertebrates.
