# Supplementary material for: Changes in Biomass and Diversity of Soil Macrofauna along a Climatic Gradient in European Boreal Forests
Source: Insects. 2022 Jan 14;13(1):94. doi: 10.3390/insects13010094 (PMC8779977; doi:10.3390/insects13010094)
Supplement: Supplementary file 1 [file insects-13-00094-s001.zip › insects-1527669-supplementary.pdf]

Changes in Biomass and Diversity of Soil Macrofauna along a Climatic Gradient in European Boreal Forests

Mikhail V. Kozlov [1,*], Vitali Zverev [1], Vladimir I. Gusarov [2], Daniil I. Korobushkin [3], Nina P. Krivosheina [3], Jaakko Mattila [4], Marko Mutanen [5], Anna Popova [3], Alexander S. Prosvirov [6], Pekka Punttila [7], Guy Söderman [8], Marzena Stańska [9], Astrid Taylor [10], Varpu Vahtera [11], Natalia A. Zubrii [12] and Elena L. Zvereva [1]

[1] Department of Biology, University of Turku, 20014 Turku, Finland; vitzve@utu.fi (V.Z.); elezve@utu.fi (E.L.Z.)
[2] Natural History Museum, University of Oslo, 0562 Oslo, Norway; vladimir.gusarov@nhm.uio.no
[3] A.N. Severtsov Institute of Ecology and Evolution, Russian Academy of Sciences, 119071 Moscow, Russia; dkorobushkin@yandex.ru (D.I.K.); dipteranina@rambler.ru (N.P.K.); velja220@mail.ru (A.P.)
[4] Finnish Museum of Natural History, University of Helsinki, 00014 Helsinki, Finland; jaakko.mattila@helsinki.fi
[5] Ecology and Genetics Research Unit, University of Oulu, 90014 Oulu, Finland; Marko.Mutanen@oulu.fi
[6] Department of Entomology, Faculty of Biology, Moscow State University, 119234 Moscow, Russia; carrabus69@mail.ru
[7] Biodiversity Centre, Finnish Environment Institute (SYKE), 00790 Helsinki, Finland; pekka.punttila@syke.fi
[8] Finnish Entomological Society c/o Finnish Museum of Natural History, University of Helsinki, 00014 Helsinki, Finland; guy.soderman@pp.inet.fi
[9] Institute of Biological Sciences, Faculty of Sciences, Siedlce University of Natural Sciences and Humanities, 08-110 Siedlce, Poland; marzena.stanska@uph.edu.pl
[10] Department of Ecology, Swedish University of Agricultural Sciences, 756 51 Uppsala, Sweden; Astrid.Taylor@slu.se
[11] Biodiversity Unit, University of Turku, 20014 Turku, Finland; varpu.vahtera@utu.fi
[12] N. Laverov Federal Center for Integrated Arctic Research, Ural Branch of the Russian Academy of Sciences, 163000 Arkhangelsk, Russia; 9052930111@mail.ru
* Correspondence: mikoz@utu.fi

Supporting information

Table S1. Characteristics of study sites and plant communities.

Site	Coordinates		Plant community					
	**Latitude, N**	**Longitude, E**	**Basal area, m^2 ha^{-1}**	**Tree species composition[a]**	**Tree diversity[b]**	**Field layer cover, %**	**Field layer diversity[b]**	**FRB[c], g m^{-2}**
R60	59° 58'	32° 11'	18.4	54S, 38P, 7B, 1A	0.69	29	0.18	560
R61	61° 00'	33° 03'	21.2	71S, 23P, 6B	0.60	34	0.73	988
R62	61° 58'	34° 14'	23.4	83P, 16B, 1S	0.49	29	0.78	738
R63	63° 00'	34° 22'	12.2	64P, 36B	0.62	18	0.92	716
R64	64° 01'	34° 04'	18.2	44B, 24S, 21P, 11A	1.08	34	0.56	1080
R65	65° 01'	34° 00'	12.6	46S, 37B, 17P	0.83	19	1.29	843
R66	66° 01'	32° 59'	14.2	49P, 47B, 3W, 1S	0.66	38	0.56	1157
R67	66° 56'	32° 12'	18.6	58P, 17S, 17B, 8A	1.11	38	0.38	850
R68	68° 01'	32° 57'	9.0	55P, 36B, 9S	0.79	35	1.19	893
R69	68° 52'	33° 07'	12.2	61P, 28B, 11S	0.84	46	0.85	605

[a] Percents of basal area by species: A, European aspen (*Populus tremula*); B, birches (mostly *Betula pubescens*, with rare additions of *B. pendula*); P, Scots pine (*Pinus sylvestris*); S, Norway spruce (*Picea abies*); W, goat willow (*Salix caprea*) (after Zvereva et al. 2020). [b] Shannon H diversity index. [c] FRB, fine root biomass (after Finér et al., 2019).

References

Finér, L.; Zverev, V.; Palviainen, M.; Romanis, T.; Kozlov, M.V. Variation in fine root biomass along a 1000 km long latitudinal climatic gradient in mixed boreal forests. *Forest Ecol. Manage.* **2019**, *432*, 649–655.

Zvereva, E.L.; Zverev, V.; Usoltsev, V.A.; Kozlov, M.V. Latitudinal pattern in community-wide herbivory does not match the pattern in herbivory averaged across common plant species. *J. Ecol.* **2000**, *108*, 2511–2520.

Table S2 Characteristics of climate[a] and of soil organic horizon[b].

Site	Climate		Soil organic horizon						
	T, °C	P, mm	Moisture, %	Thickness, mm	Particles <2 mm, %	pH	N, mg kg^{-1}	P, mg kg^{-1}	K, mg kg^{-1}
R60	3.88	850	64.2	92	24.7	4.15	13838	177	793
R61	2.90	912	52.9	113	21.0	3.81	12271	223	946
R62	2.29	796	35.2	63	15.5	5.16	6224	95	585
R63	1.37	850	48.6	67	28.6	4.57	3843	74	402
R64	1.16	796	62.2	167	47.7	3.76	11106	60	401
R65	0.84	745	61.4	98	25.2	4.02	9657	220	578
R66	0.55	741	61.3	58	31.6	4.87	7308	152	594
R67	-0.31	792	69.0	130	39.1	4.10	6850	69	652
R68	-1.55	829	63.5	90	20.0	4.63	10030	58	515
R69	-0.84	792	74.7	73	25.3	4.73	12082	211	1184

[a] The long-term (1990–2019) mean annual air temperature (T) and precipitation (P) extracted from NASAPOWER archive (power.larc.nasa.gov). [b] After Finér et al. (2019).

Reference

Finér, L.; Zverev, V.; Palviainen, M.; Romanis, T.; Kozlov, M.V. Variation in fine root biomass along a 1000 km long latitudinal climatic gradient in mixed boreal forests. *Forest Ecol. Manage.* **2019**, *432*, 649–655.

Table S3. Distribution of soil macrofauna families by trophic groups.

Feeding guild	Order	Family
Detritivores	Annelida	Lumbricidae
	Blattoptera	Ectobiidae
	Coleoptera	Hydrophilidae
	Diptera	Bibionidae, Heleomyzidae, Lauxaniidae, Lonchaeidae, Tipulidae, Trichoceridae
	Myriapoda	Julidae, Polydesmidae, Polyzonidae
Fungivores	Coleoptera	Latridiidae, Leiodidae
Herbivores	Coleoptera	Curculionidae
	Hemiptera	Cydnidae, Lygaeidae, Ortheziidae, Tingidae
	Lepidoptera	Hepialidae
Predators	Araneae	All families
	Coleoptera	Cantharidae
	Diptera	Asilidae, Empididae, Hybotidae, Muscidae, Rhagionidae, Therevidae
	Hemiptera	Ceratocombidae, Loriculidae
	Myriapoda	Lithobiidae
Combined*	Hymenoptera	Formicidae
	Diptera	Cecidomyidae, Chironomidae
	Coleoptera	Carabidae, Cryptophagidae, Elateridae, Nitidulidae, Scarabaeidae, Staphylinidae

*For species-level information consult Data S1 (below).

Data S1. Characteristics of collected soil invertebrates (space-delimited file).

Metadata

Column 1: Year of data collection.

Column 2: Month of data collection.

Column 3: Locality (consult Table S1 for coordinates).

Column 4: Sample identifier (1 to 10).

Column 5: Soil horizon, from which invertebrates were extracted.

Column 6: Order or other higher-rank taxon.

Column 7: Family.

Column 8: Genus_species.

Column 9: Number of specimens.

Column 10: Dry weight (mg). Note: all spider individuals found in the same sample were wighed together. As the result, in samples containing two or more species of spiders we were unable to divide total weight between species. In these situations we ascribed the total weight to the first identified species and zero weight to all other species.

Column 11: The species lives in/on soil (yes/no). Data on species, which do not live in soil, were excluded from all analyses.

Column 12: The species feeds in/on soil (yes/no). Data on species, which do not feed in soil, were excluded from the analyses of latitudinal pattern in biomass of individual feeding guilds.

Column 13: The species feeds with plant roots (yes/no).

Column 14: Proportion of weight attributed to phytophagy (0-1).

Column 15: Proportion of weight attributed to predation (0-1).

Column 16: Proportion of weight attributed to saprophagy (0-1).

Column 17: Proportion of weight attributed to mycophagy (0-1).

Column 18: Feeding guild. Data on species with unknown feeding habit were excluded from the analyses of latitudinal pattern in biomass of individual feeding guilds.

Data

```
2015 June R60 1 humus Coleoptera Elateridae Dalopius_marginatus 1 2.53 yes yes yes 0 0.8 0.1 0.1 combined
2015 June R60 1 humus_mineral Coleoptera Staphylinidae Geostiba_circellaris 1 0.10 yes yes no 0 1 0 0 predator
2015 June R60 1 mineral Coleoptera Elateridae Athous_subfuscus 2 9.58 yes yes yes 0 0.8 0.1 0.1 combined
2015 June R60 2 humus Annelida Lumbricidae Dendrobaena_octaedra 1 14.34 yes yes no 0 0 1 0 detritivore
2015 June R60 2 humus Lepidoptera Hepialidae Phymatopus_hecta 1 20.53 Yes Yes yes 1 0 0 0 herbivore
2015 June R60 2 humus_mineral Araneae Hahniidae Hahnia_pusilla 3 0.97 yes yes no 0 1 0 0 predator
2015 June R60 2 humus_mineral Araneae Hahniidae Hahnia_sp 3 0.00 yes yes no 0 1 0 0 predator
2015 June R60 2 humus_mineral Coleoptera Staphylinidae Geostiba_circellaris 1 0.10 yes yes no 0 1 0 0 predator
```

2015 June R60 2 humus_mineral Coleoptera Staphylinidae Ischnosoma_sp 1 0.35 yes yes no 0 1 0 0 predator
2015 June R60 3 humus Hemiptera Lygaeidae Drymus_brunneus 1 0.43 yes yes no 1 0 0 0 herbivore
2015 June R60 3 humus_mineral Hymenoptera Formicidae Myrmica_ruginodis 1 0.55 Yes Yes no 0 1 0 0 predator
2015 June R60 3 humus_mineral Araneae Linyphiidae Gen_sp 1 0.00 yes yes no 0 1 0 0 predator
2015 June R60 3 humus_mineral Araneae Linyphiidae Maro_minutus 1 0.35 yes yes no 0 1 0 0 predator
2015 June R60 3 humus_mineral Araneae Linyphiidae Semljicola_faustus 1 0.00 yes yes no 0 1 0 0 predator
2015 June R60 3 humus_mineral Myriapoda Lithobiidae Lithobius_curtipes 1 2.44 yes yes no 0 1 0 0 predator
2015 June R60 3 humus_mineral Coleoptera Staphylinidae Geostiba_circellaris 1 0.10 yes yes no 0 1 0 0 predator
2015 June R60 3 humus_mineral Coleoptera Staphylinidae Geostiba_circellaris 1 0.11 yes yes no 0 1 0 0 predator
2015 June R60 3 humus_mineral Coleoptera Staphylinidae Othius_subuliformis 1 0.73 yes yes no 0 1 0 0 predator
2015 June R60 3 humus_mineral Araneae Theridiidae Robertus_sp 1 0.00 yes yes no 0 1 0 0 predator
2015 June R60 4 humus Annelida Lumbricidae Lumbricus_sp 1 68.52 yes yes no 0 0 1 0 detritivore
2015 June R60 4 humus Coleoptera Elateridae Dalopius_marginatus 1 4.01 yes yes yes 0 0.8 0.1 0.1 combined
2015 June R60 4 humus Coleoptera Elateridae Dalopius_marginatus 1 3.41 yes yes yes 0 0.8 0.1 0.1 combined
2015 June R60 4 humus Coleoptera Staphylinidae Lathrobium_brunnipes 1 0.33 yes yes no 0 1 0 0 predator
2015 June R60 4 humus Coleoptera Curculionidae Hylobius_pinastri 1 12.91 yes yes yes 1 0 0 0 herbivore
2015 June R60 4 humus_mineral Araneae Hahniidae Hahnia_pusilla 1 0.00 yes yes no 0 1 0 0 predator
2015 June R60 4 humus_mineral Araneae Hahniidae Hahnia_sp 2 0.00 yes yes no 0 1 0 0 predator
2015 June R60 4 humus_mineral Araneae Linyphiidae Gen_sp 2 0.00 yes yes no 0 1 0 0 predator
2015 June R60 4 humus_mineral Araneae Lycosidae Pirata_Piratula_sp 2 0.00 yes yes no 0 1 0 0 predator
2015 June R60 4 humus_mineral Coleoptera Staphylinidae Ischnosoma_splendidum 1 0.30 yes yes no 0 1 0 0 predator
2015 June R60 4 humus_mineral Araneae Theridiidae Robertus_sp 1 1.81 yes yes no 0 1 0 0 predator
2015 June R60 4 mineral Diptera Chironomidae Georthocladius_sp 1 0.04 yes yes yes 0.25 0 0.75 0 combined
2015 June R60 5 humus_mineral Hymenoptera Formicidae Myrmica_ruginodis 1 0.55 Yes Yes no 0 1 0 0 predator
2015 June R60 5 humus_mineral Araneae Hahniidae Hahnia_pusilla 4 0.00 yes yes no 0 1 0 0 predator
2015 June R60 5 humus_mineral Araneae Hahniidae Hahnia_sp 3 0.00 yes yes no 0 1 0 0 predator
2015 June R60 5 humus_mineral Araneae Linyphiidae Bathyphantes_parvulus 1 0.00 yes yes no 0 1 0 0 predator
2015 June R60 5 humus_mineral Araneae Lycosidae Pirata_Piratula_sp 1 2.69 yes yes no 0 1 0 0 predator
2015 June R60 5 humus_mineral Coleoptera Staphylinidae Geostiba_circellaris 1 0.12 yes yes no 0 1 0 0 predator
2015 June R60 5 humus_mineral Coleoptera Staphylinidae Geostiba_circellaris 1 0.14 yes yes no 0 1 0 0 predator
2015 June R60 5 humus_mineral Araneae Theridiidae Robertus_sp 1 0.00 yes yes no 0 1 0 0 predator
2015 June R60 6 humus Coleoptera Elateridae Athous_subfuscus 2 5.50 yes yes yes 0 0.8 0.1 0.1 combined
2015 June R60 6 humus Coleoptera Elateridae Dalopius_marginatus 2 5.07 yes yes yes 0 0.8 0.1 0.1 combined
2015 June R60 6 humus Coleoptera Cantharidae . 1 0.13 yes yes no 0 1 0 0 predator
2015 June R60 6 humus_mineral Araneae Hahniidae Hahnia_pusilla 2 0.00 yes yes no 0 1 0 0 predator
2015 June R60 6 humus_mineral Araneae Hahniidae Hahnia_sp 5 1.73 yes yes no 0 1 0 0 predator
2015 June R60 6 humus_mineral Araneae Linyphiidae Bathyphantes_sp 1 0.00 yes yes no 0 1 0 0 predator
2015 June R60 6 humus_mineral Araneae Linyphiidae Gongylidiellum_latebricola 1 0.00 yes yes no 0 1 0 0 predator
2015 June R60 6 humus_mineral Araneae Linyphiidae Maro_minutus 1 0.00 yes yes no 0 1 0 0 predator
2015 June R60 6 humus_mineral Coleoptera Staphylinidae Geostiba_circellaris 1 0.12 yes yes no 0 1 0 0 predator
2015 June R60 6 humus_mineral Coleoptera Staphylinidae Lathrobium_longulum 1 0.25 yes yes no 0 1 0 0 predator
2015 June R60 6 humus_mineral Araneae Theridiidae Robertus_sp 1 0.00 yes yes no 0 1 0 0 predator
2015 June R60 7 humus Coleoptera Elateridae Ampedus_nigrinus 1 4.78 yes yes no 0 0.5 0.5 0 combined
2015 June R60 7 humus Coleoptera Elateridae Athous_subfuscus 1 4.02 yes yes yes 0 0.8 0.1 0.1 combined
2015 June R60 7 humus Coleoptera Elateridae Dalopius_marginatus 1 1.00 yes yes yes 0 0.8 0.1 0.1 combined
2015 June R60 7 humus Coleoptera Cantharidae Malthodes_fuscus 1 0.08 yes yes no 0 1 0 0 predator
2015 June R60 7 humus Diptera Rhagionidae Rhagio_lineola 1 1.13 yes yes no 0 1 0 0 predator
2015 June R60 7 humus_mineral Araneae Linyphiidae Diplocephalus_latifrons 1 0.36 yes yes no 0 1 0 0 predator
2015 June R60 7 humus_mineral Myriapoda Lithobiidae Lithobius_sp 1 0.52 yes yes no 0 1 0 0 predator
2015 June R60 8 humus Coleoptera Elateridae Dalopius_marginatus 1 3.51 yes yes yes 0 0.8 0.1 0.1 combined
2015 June R60 8 humus Diptera Rhagionidae Rhagio_lineola 1 0.90 yes yes no 0 1 0 0 predator
2015 June R60 8 humus Coleoptera Staphylinidae . 1 0.15 yes yes no 0 1 0 0 predator
2015 June R60 8 humus_mineral Araneae Hahniidae Hahnia_sp 1 0.00 yes yes no 0 1 0 0 predator

2015 June R60 8 humus_mineral Araneae Linyphiidae Gen_sp 1 0.00 yes yes no 0 1 0 0 predator
2015 June R60 8 humus_mineral Myriapoda Lithobiidae Lithobius_curtipes 1 1.63 yes yes no 0 1 0 0 predator
2015 June R60 8 humus_mineral Myriapoda Lithobiidae Lithobius_sp 1 0.72 yes yes no 0 1 0 0 predator
2015 June R60 8 humus_mineral Araneae Lycosidae Pirata_Piratula_sp 2 2.12 yes yes no 0 1 0 0 predator
2015 June R60 8 humus_mineral Araneae Salticidae Gen_sp 1 0.00 yes yes no 0 1 0 0 predator
2015 June R60 8 humus_mineral Araneae Salticidae Neon_reticulatus 1 0.00 yes yes no 0 1 0 0 predator
2015 June R60 8 humus_mineral Araneae Salticidae Neon_sp 1 0.00 yes yes no 0 1 0 0 predator
2015 June R60 8 humus_mineral Coleoptera Staphylinidae . 1 0.69 yes yes no 0 1 0 0 predator
2015 June R60 8 humus_mineral Coleoptera Staphylinidae Geostiba_circellaris 1 0.12 yes yes no 0 1 0 0 predator
2015 June R60 9 humus_mineral Coleoptera Staphylinidae Lathrobium_longulum 1 0.21 yes yes no 0 1 0 0 predator
2015 June R60 10 humus Coleoptera Elateridae Athous_subfuscus 1 0.48 yes yes yes 0 0.8 0.1 0.1 combined
2015 June R60 10 humus Diptera Rhagionidae Rhagio_lineola 1 2.10 yes yes no 0 1 0 0 predator
2015 June R60 10 humus_mineral Hymenoptera Formicidae Myrmica_ruginodis 2 1.13 Yes Yes no 0 1 0 0 predator
2015 June R60 10 humus_mineral Araneae Hahniidae Hahnia_sp 1 0.00 yes yes no 0 1 0 0 predator
2015 June R60 10 humus_mineral Araneae Linyphiidae Tapinocyba_pallens 1 0.00 yes yes no 0 1 0 0 predator
2015 June R60 10 humus_mineral Myriapoda Lithobiidae Lithobius_sp 1 0.18 yes yes no 0 1 0 0 predator
2015 June R60 10 humus_mineral Araneae Lycosidae Pirata_Piratula_sp 1 0.82 yes yes no 0 1 0 0 predator
2015 June R60 10 humus_mineral Coleoptera Staphylinidae Lordithon_thoracicus 1 0.44 yes yes no 0 1 0 0 predator
2015 June R60 10 humus_mineral Coleoptera Staphylinidae Othius_subuliformis 1 0.54 yes yes no 0 1 0 0 predator
2015 June R60 10 mineral Coleoptera Elateridae Athous_subfuscus 1 2.05 yes yes yes 0 0.8 0.1 0.1 combined
2015 June R61 1 humus_mineral Myriapoda Lithobiidae Lithobius_curtipes 1 1.74 yes yes no 0 1 0 0 predator
2015 June R61 1 humus_mineral Araneae Theridiidae Robertus_sp 1 0.27 yes yes no 0 1 0 0 predator
2015 June R61 1 mineral Diptera Asilidae Asilus_sp 1 1.02 yes yes no 0 1 0 0 predator
2015 June R61 2 humus Diptera Rhagionidae Rhagio_lineola 1 0.74 yes yes no 0 1 0 0 predator
2015 June R61 2 humus Diptera Rhagionidae Rhagio_lineola 1 0.80 yes yes no 0 1 0 0 predator
2015 June R61 2 humus_mineral Hymenoptera Formicidae Myrmica_ruginodis 17 7.82 Yes Yes no 0 1 0 0 predator
2015 June R61 2 humus_mineral Araneae Hahniidae Hahnia_pusilla 1 0.00 yes yes no 0 1 0 0 predator
2015 June R61 2 humus_mineral Araneae Hahniidae Hahnia_sp 1 0.00 yes yes no 0 1 0 0 predator
2015 June R61 2 humus_mineral Araneae Linyphiidae Tapinocyba_pallens 1 0.34 yes yes no 0 1 0 0 predator
2015 June R61 2 mineral Coleoptera Elateridae Athous_subfuscus 1 3.62 yes yes yes 0 0.8 0.1 0.1 combined
2015 June R61 2 mineral Diptera Rhagionidae Rhagio_lineola 1 1.22 yes yes no 0 1 0 0 predator
2015 June R61 2 mineral Diptera Rhagionidae Rhagio_lineola 1 1.60 yes yes no 0 1 0 0 predator
2015 June R61 3 humus Coleoptera Elateridae Athous_subfuscus 1 2.14 yes yes yes 0 0.8 0.1 0.1 combined
2015 June R61 3 humus_mineral Hymenoptera Formicidae Myrmica_ruginodis 1 0.77 Yes Yes no 0 1 0 0 predator
2015 June R61 4 humus Coleoptera Cantharidae Malthodes_fuscus 1 0.06 yes yes no 0 1 0 0 predator
2015 June R61 4 humus Diptera Rhagionidae Rhagio_lineola 1 1.58 yes yes no 0 1 0 0 predator
2015 June R61 4 humus Coleoptera Elateridae Paraphotistus_impressus 1 16.74 yes yes yes 0.6 0.13 0.13 0.13 combined
2015 June R61 4 humus_mineral Hymenoptera Formicidae Myrmica_rubra 1 0.81 Yes Yes no 0 1 0 0 predator
2015 June R61 4 humus_mineral Hymenoptera Formicidae Myrmica_ruginodis 1 0.81 Yes Yes no 0 1 0 0 predator
2015 June R61 4 humus_mineral Araneae Hahniidae Hahnia_sp 1 0.12 yes yes no 0 1 0 0 predator
2015 June R61 5 humus Coleoptera Elateridae Athous_subfuscus 1 0.62 yes yes yes 0 0.8 0.1 0.1 combined
2015 June R61 5 humus Coleoptera Cantharidae . 1 0.30 yes yes no 0 1 0 0 predator
2015 June R61 5 humus_mineral Araneae Hahniidae Hahnia_pusilla 1 0.61 yes yes no 0 1 0 0 predator
2015 June R61 5 humus_mineral Araneae Hahniidae Hahnia_sp 2 0.00 yes yes no 0 1 0 0 predator
2015 June R61 5 humus_mineral Araneae Linyphiidae Porrhomma_campbelli 1 0.00 yes yes no 0 1 0 0 predator
2015 June R61 5 mineral Diptera Rhagionidae Rhagio_lineola 1 1.52 yes yes no 0 1 0 0 predator
2015 June R61 5 mineral Coleoptera Curculionidae . 1 2.44 yes yes yes 1 0 0 0 herbivore
2015 June R61 6 humus Coleoptera Cantharidae Malthodes_fuscus 1 0.13 yes yes no 0 1 0 0 predator
2015 June R61 6 humus Coleoptera Cantharidae Malthodes_fuscus 1 0.13 yes yes no 0 1 0 0 predator
2015 June R61 6 humus Diptera Empididae Phyllodromia_melanocephala 1 0.19 yes yes no 0 1 0 0 predator
2015 June R61 6 humus Coleoptera Cantharidae Malthodes_fuscus 1 0.24 no no no 0.5 0.5 0 0 combined
2015 June R61 6 humus_mineral Myriapoda Lithobiidae Lithobius_curtipes 1 1.63 yes yes no 0 1 0 0 predator
2015 June R61 6 humus_mineral Coleoptera Staphylinidae Geostiba_circellaris 1 0.09 yes yes no 0 1 0 0 predator

2015 June R61 6 humus_mineral Coleoptera Staphylinidae Rugilus_rufipes 1 0.62 yes yes no 0 1 0 0 predator
2015 June R61 7 humus Coleoptera Elateridae Athous_subfuscus 1 8.50 yes yes yes 0 0.8 0.1 0.1 combined
2015 June R61 7 humus_mineral Coleoptera Staphylinidae Atheta_myrmecobia 1 0.13 yes yes no 0 1 0 0 predator
2015 June R61 7 humus_mineral Coleoptera Staphylinidae Atheta_myrmecobia 1 0.15 yes yes no 0 1 0 0 predator
2015 June R61 7 humus_mineral Coleoptera Staphylinidae Geostiba_circellaris 1 0.08 yes yes no 0 1 0 0 predator
2015 June R61 7 humus_mineral Coleoptera Staphylinidae Geostiba_circellaris 1 0.09 yes yes no 0 1 0 0 predator
2015 June R61 7 humus_mineral Coleoptera Staphylinidae Geostiba_circellaris 1 0.12 yes yes no 0 1 0 0 predator
2015 June R61 7 mineral Coleoptera Elateridae Athous_subfuscus 2 5.12 yes yes yes 0 0.8 0.1 0.1 combined
2015 June R61 8 humus Coleoptera Elateridae Athous_subfuscus 1 5.59 yes yes yes 0 0.8 0.1 0.1 combined
2015 June R61 8 humus_mineral Hymenoptera Formicidae Myrmica_ruginodis 1 0.59 Yes Yes no 0 1 0 0 predator
2015 June R61 8 humus_mineral Araneae Linyphiidae Tapinocyba_pallens 1 0.00 yes yes no 0 1 0 0 predator
2015 June R61 8 humus_mineral Coleoptera Staphylinidae Ischnosoma_sp 1 0.26 yes yes no 0 1 0 0 predator
2015 June R61 8 humus_mineral Coleoptera Staphylinidae Philonthus_albipes 1 0.80 yes yes no 0 1 0 0 predator
2015 June R61 8 humus_mineral Araneae Theridiidae Robertus_sp 1 0.40 yes yes no 0 1 0 0 predator
2015 June R61 9 humus Coleoptera Leiodidae Agathidium_pisanum 1 0.35 yes yes no 0 0 0 1 fungivore
2015 June R61 9 humus_mineral Myriapoda Lithobiidae Lithobius_curtipes 1 1.40 yes yes no 0 1 0 0 predator
2015 June R61 9 humus_mineral Araneae Salticidae Evarcha_sp 1 0.00 yes yes no 0 1 0 0 predator
2015 June R61 9 humus_mineral Araneae Theridiidae Robertus_scoticus 1 2.86 yes yes no 0 1 0 0 predator
2015 June R61 9 mineral Coleoptera Elateridae Paraphotistus_impressus 1 3.37 yes yes yes 0.6 0.13 0.13 0.13 combined
2015 June R61 10 humus Diptera Rhagionidae Rhagio_lineola 1 1.28 yes yes no 0 1 0 0 predator
2015 June R61 10 humus Coleoptera Staphylinidae . 1 0.08 yes yes no 0 1 0 0 predator
2015 June R61 10 humus_mineral Myriapoda Lithobiidae Lithobius_sp 1 0.19 yes yes no 0 1 0 0 predator
2015 June R62 1 humus Annelida Lumbricidae Dendrodrilus_rubidus_tenuis 1 23.52 yes yes no 0 0 1 0 detritivore
2015 June R62 1 humus Coleoptera Carabidae Bradycellus_caucasicus 1 0.81 yes yes yes 1 0 0 0 herbivore
2015 June R62 1 humus Hemiptera Lygaeidae Drymus_brunneus 1 0.33 yes yes no 1 0 0 0 herbivore
2015 June R62 1 humus Hemiptera Lygaeidae Drymus_brunneus 1 0.34 yes yes no 1 0 0 0 herbivore
2015 June R62 1 humus_mineral Hymenoptera Formicidae Myrmica_ruginodis 2 1.14 Yes Yes no 0 1 0 0 predator
2015 June R62 1 humus_mineral Araneae Hahniidae Hahnia_pusilla 1 0.00 yes yes no 0 1 0 0 predator
2015 June R62 1 humus_mineral Araneae Hahniidae Hahnia_sp 1 0.00 yes yes no 0 1 0 0 predator
2015 June R62 1 humus_mineral Araneae Linyphiidae Asthenargus_paganus 2 0.76 yes yes no 0 1 0 0 predator
2015 June R62 1 humus_mineral Araneae Linyphiidae Gen_sp 3 0.00 yes yes no 0 1 0 0 predator
2015 June R62 1 humus_mineral Myriapoda Lithobiidae Lithobius_curtipes 1 1.21 yes yes no 0 1 0 0 predator
2015 June R62 1 humus_mineral Myriapoda Lithobiidae Lithobius_sp 1 0.56 yes yes no 0 1 0 0 predator
2015 June R62 1 humus_mineral Coleoptera Staphylinidae Ischnosoma_sp 1 0.29 yes yes no 0 1 0 0 predator
2015 June R62 1 humus_mineral Hymenoptera Formicidae Lasius_platythorax 1 0.33 Yes Yes no 0.8 0.2 0 0 combined
2015 June R62 1 mineral Blattoptera Ectobiidae Ectobius_sylvestris 1 0.95 yes yes no 0 0 1 0 detritivore
2015 June R62 1 mineral Blattoptera Ectobiidae Ectobius_sylvestris 1 1.59 yes yes no 0 0 1 0 detritivore
2015 June R62 2 humus Annelida Lumbricidae Dendrodrilus_rubidus_tenuis 1 23.88 yes yes no 0 0 1 0 detritivore
2015 June R62 2 humus Hemiptera Lygaeidae Drymus_brunneus 1 0.36 yes yes no 1 0 0 0 herbivore
2015 June R62 2 humus_mineral Coleoptera Carabidae Pterostichus_strenuus 1 2.43 yes yes no 0 1 0 0 predator
2015 June R62 2 humus_mineral Araneae Clubionidae Clubiona_sp 1 3.28 yes yes no 0 1 0 0 predator
2015 June R62 2 humus_mineral Araneae Linyphiidae Gen_sp 1 0.00 yes yes no 0 1 0 0 predator
2015 June R62 2 humus_mineral Araneae Linyphiidae Maso_sundevalli 1 0.00 yes yes no 0 1 0 0 predator
2015 June R62 2 humus_mineral Myriapoda Lithobiidae Lithobius_curtipes 1 0.46 yes yes no 0 1 0 0 predator
2015 June R62 2 humus_mineral Myriapoda Lithobiidae Lithobius_curtipes 1 0.98 yes yes no 0 1 0 0 predator
2015 June R62 2 humus_mineral Hymenoptera Formicidae Lasius_platythorax 3 1.20 Yes Yes no 0.8 0.2 0 0 combined
2015 June R62 2 mineral Myriapoda Julidae Ommatoiulus_sabulosus 1 33.01 yes yes no 0 0 1 0 detritivore
2015 June R62 2 mineral Coleoptera Elateridae Dalopius_marginatus 1 2.10 yes yes yes 0 0.8 0.1 0.1 combined
2015 June R62 3 humus Diptera Rhagionidae Rhagio_lineola 1 0.59 yes yes no 0 1 0 0 predator
2015 June R62 3 humus Diptera Rhagionidae Rhagio_lineola 1 0.93 yes yes no 0 1 0 0 predator
2015 June R62 3 humus Diptera Rhagionidae Rhagio_lineola 1 1.39 yes yes no 0 1 0 0 predator
2015 June R62 3 humus Coleoptera Curculionidae Strophosoma_capitatum 1 3.73 yes yes yes 1 0 0 0 herbivore
2015 June R62 3 humus Hemiptera Cydnidae Adomerus_biguttatus 1 5.23 yes yes yes 1 0 0 0 herbivore

2015 June R62 3 humus Hemiptera Lygaeidae Drymus_brunneus 1 0.34 yes yes no 1 0 0 0 herbivore
2015 June R62 3 humus_mineral Hymenoptera Formicidae Myrmica_ruginodis 6 2.56 Yes Yes no 0 1 0 0 predator
2015 June R62 3 humus_mineral Araneae Linyphiidae Gen_sp 2 0.00 yes yes no 0 1 0 0 predator
2015 June R62 3 humus_mineral Araneae Linyphiidae Maso_sundevalli 1 0.00 yes yes no 0 1 0 0 predator
2015 June R62 3 humus_mineral Araneae Miturgidae Zora_sp 1 0.00 yes yes no 0 1 0 0 predator
2015 June R62 3 humus_mineral Coleoptera Staphylinidae Ischnosoma_sp 1 0.31 yes yes no 0 1 0 0 predator
2015 June R62 3 humus_mineral Araneae Thomisidae Ozyptila_sp 1 1.87 yes yes no 0 1 0 0 predator
2015 June R62 3 mineral Myriapoda Julidae Ommatoiulus_sabulosus 1 16.51 yes yes no 0 0 1 0 detritivore
2015 June R62 4 humus Annelida Lumbricidae Dendrodrilus_rubidus_tenuis 2 44.88 yes yes no 0 0 1 0 detritivore
2015 June R62 4 humus Coleoptera Elateridae Athous_subfuscus 1 2.44 yes yes yes 0 0.8 0.1 0.1 combined
2015 June R62 4 humus Coleoptera Staphylinidae Lathrobium_brunnipes 1 0.29 yes yes no 0 1 0 0 predator
2015 June R62 4 humus Hemiptera Lygaeidae Drymus_brunneus 1 0.12 yes yes no 1 0 0 0 herbivore
2015 June R62 4 humus_mineral Myriapoda Julidae Ommatoiulus_cf_sabulosus 1 19.20 yes yes no 0 0 1 0 detritivore
2015 June R62 4 humus_mineral Myriapoda Polydesmidae Polydesmus_denticulatus 1 4.62 yes yes no 0 0 1 0 detritivore
2015 June R62 4 humus_mineral Araneae Dictynidae Lathys_heterophthalma 1 0.00 yes yes no 0 1 0 0 predator
2015 June R62 4 humus_mineral Araneae Linyphiidae Gen_sp 2 0.00 yes yes no 0 1 0 0 predator
2015 June R62 4 humus_mineral Araneae Miturgidae Zora_sp 2 1.66 yes yes no 0 1 0 0 predator
2015 June R62 4 humus_mineral Coleoptera Staphylinidae Othius_subuliformis 1 0.49 yes yes no 0 1 0 0 predator
2015 June R62 4 mineral Coleoptera Elateridae Dalopius_marginatus 1 2.81 yes yes yes 0 0.8 0.1 0.1 combined
2015 June R62 5 humus Coleoptera Latridiidae Cortinicara_gibbosa 1 0.10 yes yes no 0 0 0 1 fungivore
2015 June R62 5 humus Annelida Lumbricidae Dendrobaena_octaedra 1 25.44 yes yes no 0 0 1 0 detritivore
2015 June R62 5 humus Diptera Rhagionidae Rhagio_lineola 1 2.77 yes yes no 0 1 0 0 predator
2015 June R62 5 humus_mineral Hymenoptera Formicidae Formica_fusca 1 0.92 Yes Yes no 0 1 0 0 predator
2015 June R62 5 humus_mineral Hymenoptera Formicidae Myrmica_ruginodis 2 1.14 Yes Yes no 0 1 0 0 predator
2015 June R62 5 humus_mineral Araneae Linyphiidae Erigonella_hiemalis 1 0.32 yes yes no 0 1 0 0 predator
2015 June R62 5 humus_mineral Araneae Linyphiidae Gen_sp 1 0.00 yes yes no 0 1 0 0 predator
2015 June R62 5 humus_mineral Myriapoda Lithobiidae Lithobius_sp 1 0.05 yes yes no 0 1 0 0 predator
2015 June R62 5 mineral Coleoptera Elateridae Athous_subfuscus 1 2.47 yes yes yes 0 0.8 0.1 0.1 combined
2015 June R62 5 mineral Diptera Rhagionidae Rhagio_lineola 1 2.57 yes yes no 0 1 0 0 predator
2015 June R62 6 humus Annelida Lumbricidae Dendrodrilus_rubidus_tenuis 2 5.44 yes yes no 0 0 1 0 detritivore
2015 June R62 6 humus Lepidoptera Hepialidae Phymatopus_hecta 1 12.80 Yes Yes yes 1 0 0 0 herbivore
2015 June R62 6 humus Coleoptera Nemonychidae Cimberis_attelaboides 1 0.86 no no no 1 0 0 0 herbivore
2015 June R62 6 humus_mineral Hymenoptera Formicidae Myrmica_ruginodis 1 0.70 Yes Yes no 0 1 0 0 predator
2015 June R62 6 humus_mineral Araneae Hahniidae Hahnia_pusilla 2 1.44 yes yes no 0 1 0 0 predator
2015 June R62 6 humus_mineral Araneae Linyphiidae Centromerus_arcanus 1 0.00 yes yes no 0 1 0 0 predator
2015 June R62 6 humus_mineral Araneae Linyphiidae Gen_sp 5 0.00 yes yes no 0 1 0 0 predator
2015 June R62 6 humus_mineral Araneae Linyphiidae Porrhomma_pallidum 1 0.00 yes yes no 0 1 0 0 predator
2015 June R62 6 humus_mineral Araneae Linyphiidae Walckenaeria_dysderoides 1 0.00 yes yes no 0 1 0 0 predator
2015 June R62 6 mineral Annelida Lumbricidae Dendrodrilus_rubidus_tenuis 1 14.09 yes yes no 0 0 1 0 detritivore
2015 June R62 6 mineral Coleoptera Elateridae Paraphotistus_impressus 1 5.61 yes yes yes 0.6 0.13 0.13 0.13 combined
2015 June R62 7 humus Coleoptera Elateridae Athous_subfuscus 4 22.16 yes yes yes 0 0.8 0.1 0.1 combined
2015 June R62 7 humus Coleoptera Elateridae Sericus_cf_brunneus 1 9.02 yes yes yes 0.5 0 0 0.5 combined
2015 June R62 7 humus_mineral Hymenoptera Formicidae Myrmica_ruginodis 6 2.95 Yes Yes no 0 1 0 0 predator
2015 June R62 7 humus_mineral Araneae Linyphiidae Tapinocyba_pallens 1 0.57 yes yes no 0 1 0 0 predator
2015 June R62 7 humus_mineral Myriapoda Lithobiidae Lithobius_curtipes 1 1.38 yes yes no 0 1 0 0 predator
2015 June R62 7 humus_mineral Myriapoda Lithobiidae Lithobius_curtipes 1 1.49 yes yes no 0 1 0 0 predator
2015 June R62 7 humus_mineral Myriapoda Lithobiidae Lithobius_sp 1 0.62 yes yes no 0 1 0 0 predator
2015 June R62 7 humus_mineral Coleoptera Staphylinidae Geostiba_circellaris 1 0.12 yes yes no 0 1 0 0 predator
2015 June R62 7 humus_mineral Coleoptera Staphylinidae Geostiba_circellaris 1 0.13 yes yes no 0 1 0 0 predator
2015 June R62 7 humus_mineral Coleoptera Staphylinidae Geostiba_circellaris 1 0.14 yes yes no 0 1 0 0 predator
2015 June R62 8 humus Annelida Lumbricidae Dendrobaena_octaedra 1 26.21 yes yes no 0 0 1 0 detritivore
2015 June R62 8 humus Coleoptera Elateridae Athous_subfuscus 1 0.13 yes yes yes 0 0.8 0.1 0.1 combined
2015 June R62 8 humus Coleoptera Elateridae Dalopius_marginatus 4 4.04 yes yes yes 0 0.8 0.1 0.1 combined

2015 June R62 8 humus Coleoptera Curculionidae Strophosoma_capitatum 1 2.95 yes yes yes 1 0 0 0 herbivore
2015 June R62 8 humus_mineral Araneae Linyphiidae Centromerus_arcanus 1 0.00 yes yes no 0 1 0 0 predator
2015 June R62 8 humus_mineral Araneae Linyphiidae Gen_sp 1 1.47 yes yes no 0 1 0 0 predator
2015 June R62 8 humus_mineral Coleoptera Staphylinidae Ischnosoma_sp 1 0.30 yes yes no 0 1 0 0 predator
2015 June R62 8 humus_mineral Coleoptera Staphylinidae Stenus_impressus 1 0.34 yes yes no 0 1 0 0 predator
2015 June R62 8 humus_mineral Araneae Theridiidae Gen_sp 1 0.00 yes yes no 0 1 0 0 predator
2015 June R62 8 mineral Coleoptera Elateridae Paraphotistus_impressus 1 11.83 yes yes yes 0.6 0.13 0.13 0.13 combined
2015 June R62 9 humus Coleoptera Elateridae Athous_subfuscus 1 2.59 yes yes yes 0 0.8 0.1 0.1 combined
2015 June R62 9 humus Coleoptera . . 1 7.11 . . . .
2015 June R62 9 humus_mineral Coleoptera Staphylinidae Sepedophilus_pedicularius 1 0.16 yes yes no 0 0 0 1 fungivore
2015 June R62 9 humus_mineral Myriapoda Lithobiidae Lithobius_curtipes 1 0.18 yes yes no 0 1 0 0 predator
2015 June R62 9 humus_mineral Araneae Miturgidae Zora_sp 1 0.44 yes yes no 0 1 0 0 predator
2015 June R62 10 humus Annelida Lumbricidae Dendrobaena_octaedra 1 0.17 yes yes no 0 0 1 0 detritivore
2015 June R62 10 humus Coleoptera Carabidae Calathus_melanocephalus 1 0.51 yes yes no 0 1 0 0 predator
2015 June R62 10 humus Coleoptera Curculionidae Strophosoma_capitatum 1 1.62 yes yes yes 1 0 0 0 herbivore
2015 June R62 10 humus_mineral Araneae Linyphiidae Agyneta_subtilis 1 0.00 yes yes no 0 1 0 0 predator
2015 June R62 10 humus_mineral Araneae Linyphiidae Micrargus_herbigradus 1 0.00 yes yes no 0 1 0 0 predator
2015 June R62 10 humus_mineral Araneae Linyphiidae Tapinocyba_pallens 1 0.64 yes yes no 0 1 0 0 predator
2015 June R62 10 humus_mineral Myriapoda Lithobiidae Lithobius_curtipes 1 0.46 yes yes no 0 1 0 0 predator
2015 June R62 10 humus_mineral Myriapoda Lithobiidae Lithobius_curtipes 1 0.84 yes yes no 0 1 0 0 predator
2015 June R62 10 humus_mineral Myriapoda Lithobiidae Lithobius_curtipes 1 1.29 yes yes no 0 1 0 0 predator
2015 June R62 10 humus_mineral Myriapoda Lithobiidae Lithobius_curtipes 1 2.13 yes yes no 0 1 0 0 predator
2015 June R62 10 mineral Myriapoda Diplopoda . 1 13.39 Yes Yes no 0 0 1 0 detritivore
2015 June R63 1 humus Annelida Lumbricidae . 1 0.41 yes yes no 0 0 1 0 detritivore
2015 June R63 1 humus Coleoptera Elateridae Athous_subfuscus 1 4.30 yes yes yes 0 0.8 0.1 0.1 combined
2015 June R63 1 humus Coleoptera Elateridae Dalopius_marginatus 1 0.95 yes yes yes 0 0.8 0.1 0.1 combined
2015 June R63 1 humus_mineral Coleoptera Carabidae Pterostichus_oblongopunctatus 1 12.90 yes yes no 0 1 0 0 predator
2015 June R63 1 humus_mineral Araneae Linyphiidae Gen_sp 1 0.00 yes yes no 0 1 0 0 predator
2015 June R63 1 humus_mineral Araneae Linyphiidae Tapinocyba_pallens 1 0.32 yes yes no 0 1 0 0 predator
2015 June R63 1 humus_mineral Myriapoda Lithobiidae Lithobius_curtipes 1 0.19 yes yes no 0 1 0 0 predator
2015 June R63 1 humus_mineral Myriapoda Lithobiidae Lithobius_curtipes 1 0.28 yes yes no 0 1 0 0 predator
2015 June R63 1 humus_mineral Myriapoda Lithobiidae Lithobius_curtipes 1 1.44 yes yes no 0 1 0 0 predator
2015 June R63 1 humus_mineral Araneae Theridiidae Robertus_sp 1 0.00 yes yes no 0 1 0 0 predator
2015 June R63 2 humus Coleoptera Elateridae Athous_subfuscus 1 0.83 yes yes yes 0 0.8 0.1 0.1 combined
2015 June R63 2 humus Coleoptera Curculionidae Strophosoma_capitatum 1 1.78 yes yes yes 1 0 0 0 herbivore
2015 June R63 2 humus_mineral Myriapoda Lithobiidae Lithobius_curtipes 1 0.90 yes yes no 0 1 0 0 predator
2015 June R63 2 humus_mineral Myriapoda Lithobiidae Lithobius_curtipes 1 1.07 yes yes no 0 1 0 0 predator
2015 June R63 3 humus Coleoptera Elateridae Dalopius_marginatus 2 3.13 yes yes yes 0 0.8 0.1 0.1 combined
2015 June R63 3 humus Diptera Rhagionidae Rhagio_lineola 1 0.64 yes yes no 0 1 0 0 predator
2015 June R63 3 humus_mineral Araneae Araneidae Cercidia_prominens 1 0.00 no no no 0 1 0 0 predator
2015 June R63 3 humus_mineral Araneae Linyphiidae Tapinocyba_pallens 1 4.43 yes yes no 0 1 0 0 predator
2015 June R63 3 humus_mineral Myriapoda Lithobiidae Lithobius_sp 1 0.17 yes yes no 0 1 0 0 predator
2015 June R63 3 humus_mineral Coleoptera Staphylinidae Othius_subuliformis 1 0.56 yes yes no 0 1 0 0 predator
2015 June R63 3 humus_mineral Araneae Theridiidae Robertus_lividus 1 0.00 yes yes no 0 1 0 0 predator
2015 June R63 3 humus_mineral Araneae Theridiidae Robertus_sp 2 0.00 yes yes no 0 1 0 0 predator
2015 June R63 3 mineral Coleoptera Curculionidae . 1 2.07 yes yes yes 1 0 0 0 herbivore
2015 June R63 4 humus Coleoptera Elateridae Athous_subfuscus 1 0.07 yes yes yes 0 0.8 0.1 0.1 combined
2015 June R63 4 humus Coleoptera Elateridae Dalopius_marginatus 1 0.90 yes yes yes 0 0.8 0.1 0.1 combined
2015 June R63 4 humus Diptera Rhagionidae Rhagio_lineola 1 0.94 yes yes no 0 1 0 0 predator
2015 June R63 4 humus Diptera Rhagionidae Rhagio_lineola 1 1.68 yes yes no 0 1 0 0 predator
2015 June R63 4 humus Coleoptera Staphylinidae . 1 1.71 yes yes no 0 1 0 0 predator
2015 June R63 4 humus_mineral Araneae Linyphiidae Asthenargus_paganus 1 0.00 yes yes no 0 1 0 0 predator
2015 June R63 4 humus_mineral Araneae Linyphiidae Gen_sp 4 0.37 yes yes no 0 1 0 0 predator

2015 June R63 4 humus_mineral Coleoptera Staphylinidae Geostiba_circellaris 1 0.11 yes yes no 0 1 0 0 predator
2015 June R63 4 humus_mineral Araneae Theridiidae Robertus_sp 1 0.00 yes yes no 0 1 0 0 predator
2015 June R63 5 humus Coleoptera Elateridae Dalopius_marginatus 1 1.14 yes yes yes 0 0.8 0.1 0.1 combined
2015 June R63 5 humus Coleoptera Staphylinidae . 1 0.02 yes yes no 0 1 0 0 predator
2015 June R63 5 humus Coleoptera Elateridae Selatosomus_melancholicus 1 8.46 yes yes yes 0.6 0.13 0.13 0.13 combined
2015 June R63 5 humus Coleoptera Polyphaga . 1 0.06 . . . .
2015 June R63 5 humus_mineral Coleoptera Carabidae Pterostichus_strenuus 1 2.93 yes yes no 0 1 0 0 predator
2015 June R63 5 humus_mineral Coleoptera Staphylinidae Geostiba_circellaris 1 0.10 yes yes no 0 1 0 0 predator
2015 June R63 5 humus_mineral Coleoptera Staphylinidae Geostiba_circellaris 1 0.11 yes yes no 0 1 0 0 predator
2015 June R63 5 humus_mineral Coleoptera Staphylinidae Geostiba_circellaris 1 0.11 yes yes no 0 1 0 0 predator
2015 June R63 5 mineral Coleoptera Elateridae Selatosomus_melancholicus 1 11.22 yes yes yes 0.6 0.13 0.13 0.13 combined
2015 June R63 6 humus Annelida Lumbricidae Dendrobaena_octaedra 2 16.26 yes yes no 0 0 1 0 detritivore
2015 June R63 6 humus Coleoptera Elateridae Paraphotistus_impressus 1 9.57 yes yes yes 0.6 0.13 0.13 0.13 combined
2015 June R63 6 humus_mineral Coleoptera Carabidae Pterostichus_oblongopunctatus 1 10.94 yes yes no 0 1 0 0 predator
2015 June R63 6 humus_mineral Araneae Linyphiidae Tapinocyba_pallens 1 0.12 yes yes no 0 1 0 0 predator
2015 June R63 6 humus_mineral Myriapoda Lithobiidae Lithobius_sp 1 0.04 yes yes no 0 1 0 0 predator
2015 June R63 6 humus_mineral Coleoptera Staphylinidae Oxypoda_annularis 1 0.07 yes yes no 0 1 0 0 predator
2015 June R63 6 humus_mineral Coleoptera Staphylinidae Oxypoda_annularis 1 0.09 yes yes no 0 1 0 0 predator
2015 June R63 6 humus_mineral Coleoptera Staphylinidae Oxypoda_annularis 1 0.09 yes yes no 0 1 0 0 predator
2015 June R63 6 mineral Diptera Asilidae Dioctria_hyalipennis 1 5.34 yes yes no 0 1 0 0 predator
2015 June R63 7 humus Coleoptera Elateridae Athous_subfuscus 1 8.88 yes yes yes 0 0.8 0.1 0.1 combined
2015 June R63 7 humus Coleoptera Elateridae Dalopius_marginatus 1 2.99 yes yes yes 0 0.8 0.1 0.1 combined
2015 June R63 7 humus Coleoptera Curculionidae Strophosoma_capitatum 1 1.43 yes yes yes 1 0 0 0 herbivore
2015 June R63 7 humus_mineral Coleoptera Staphylinidae Xantholinus_laevigatus 1 1.18 yes yes no 0 1 0 0 predator
2015 June R63 7 mineral Coleoptera Elateridae Paraphotistus_impressus 1 4.32 yes yes yes 0.6 0.13 0.13 0.13 combined
2015 June R63 8 humus Coleoptera Elateridae Dalopius_marginatus 1 0.33 yes yes yes 0 0.8 0.1 0.1 combined
2015 June R63 8 humus_mineral Myriapoda Lithobiidae Lithobius_curtipes 1 0.54 yes yes no 0 1 0 0 predator
2015 June R63 8 humus_mineral Hymenoptera Formicidae Lasius_platythorax 1 0.33 Yes Yes no 0.8 0.2 0 0 combined
2015 June R63 9 humus Annelida Lumbricidae Dendrobaena_octaedra 2 17.38 yes yes no 0 0 1 0 detritivore
2015 June R63 9 humus Coleoptera Elateridae Athous_subfuscus 2 5.85 yes yes yes 0 0.8 0.1 0.1 combined
2015 June R63 9 humus Diptera Rhagionidae Rhagio_lineola 1 1.03 yes yes no 0 1 0 0 predator
2015 June R63 9 humus_mineral Coleoptera Carabidae Calathus_micropterus 1 4.10 yes yes no 0 1 0 0 predator
2015 June R63 9 humus_mineral Hymenoptera Formicidae Myrmica_ruginodis 2 1.74 Yes Yes no 0 1 0 0 predator
2015 June R63 9 humus_mineral Araneae Linyphiidae Minyriolus_pusillus 1 0.06 yes yes no 0 1 0 0 predator
2015 June R63 9 humus_mineral Coleoptera Staphylinidae Othius_punctulatus 1 3.83 yes yes no 0 1 0 0 predator
2015 June R63 10 humus Diptera Heleomyzidae Neoleria_sp 1 4.31 yes yes no 0 0 1 0 detritivore
2015 June R63 10 humus Coleoptera Curculionidae Strophosoma_capitatum 1 2.53 yes yes yes 1 0 0 0 herbivore
2015 June R63 10 humus Coleoptera . . 1 1.29 . . . .
2015 June R63 10 humus_mineral Araneae Linyphiidae Walckenaeria_cucullata 1 0.00 yes yes no 0 1 0 0 predator
2015 June R63 10 humus_mineral Myriapoda Lithobiidae Lithobius_curtipes 1 0.94 yes yes no 0 1 0 0 predator
2015 June R63 10 humus_mineral Myriapoda Lithobiidae Lithobius_sp 1 0.34 yes yes no 0 1 0 0 predator
2015 June R63 10 humus_mineral Coleoptera Staphylinidae Acrotona_silvicola 1 0.12 yes yes no 0 1 0 0 predator
2015 June R63 10 humus_mineral Coleoptera Staphylinidae Acrotona_silvicola 1 0.12 yes yes no 0 1 0 0 predator
2015 June R63 10 humus_mineral Coleoptera Staphylinidae Geostiba_circellaris 1 0.11 yes yes no 0 1 0 0 predator
2015 June R63 10 humus_mineral Araneae Theridiidae Robertus_lividus 1 1.46 yes yes no 0 1 0 0 predator
2015 June R63 10 humus_mineral Coleoptera . . 1 0.09 . . . .
2015 June R63 10 mineral Coleoptera Elateridae Paraphotistus_impressus 1 0.35 yes yes yes 0.6 0.13 0.13 0.13 combined
2015 June R63 10 mineral Coleoptera Elateridae Paraphotistus_impressus 1 33.34 yes yes yes 0.6 0.13 0.13 0.13 combined
2015 June R63 10 mineral Coleoptera Curculionidae Strophosoma_capitatum 1 5.46 yes yes yes 1 0 0 0 herbivore
2015 June R63 10 mineral Coleoptera Curculionidae Strophosoma_capitatum 1 5.49 yes yes yes 1 0 0 0 herbivore
2015 June R64 1 humus Myriapoda Polyzonidae Polyzonium_germanicum 1 0.68 yes yes no 0 0 1 0 detritivore
2015 June R64 1 humus Diptera Rhagionidae Rhagio_lineola 1 2.71 yes yes no 0 1 0 0 predator
2015 June R64 1 humus_mineral Myriapoda Lithobiidae Lithobius_curtipes 1 1.00 yes yes no 0 1 0 0 predator

2015 June R64 1 mineral Diptera Rhagionidae Rhagio_tringarius 1 14.12 yes yes no 0 1 0 0 predator
2015 June R64 2 humus_mineral Araneae Linyphiidae Gen_sp 1 0.21 yes yes no 0 1 0 0 predator
2015 June R64 2 humus_mineral Coleoptera Staphylinidae Ischnosoma_splendidum 1 0.33 yes yes no 0 1 0 0 predator
2015 June R64 3 humus Coleoptera Elateridae Eanus_costalis 1 10.92 yes yes no 0 0.5 0.5 0 combined
2015 June R64 3 humus Coleoptera Elateridae Athous_subfuscus 3 10.43 yes yes yes 0 0.8 0.1 0.1 combined
2015 June R64 3 humus Lepidoptera Hepialidae Korscheltellus_fusconebulosa 1 1.52 Yes Yes yes 1 0 0 0 herbivore
2015 June R64 3 humus Hemiptera Lygaeidae Drymus_brunneus 1 0.97 yes yes no 1 0 0 0 herbivore
2015 June R64 3 humus Diptera . . 1 1.17 . . . .
2015 June R64 3 humus_mineral Coleoptera Staphylinidae Oxypoda_annularis 1 0.09 yes yes no 0 1 0 0 predator
2015 June R64 3 humus_mineral Coleoptera Staphylinidae Phloeopora_sp 1 0.06 yes yes no 0 1 0 0 predator
2015 June R64 4 humus Coleoptera Elateridae Athous_subfuscus 2 8.30 yes yes yes 0 0.8 0.1 0.1 combined
2015 June R64 4 humus Diptera Rhagionidae Rhagio_sp 1 6.59 yes yes no 0 1 0 0 predator
2015 June R64 4 humus Coleoptera Elateridae Paraphotistus_impressus 1 18.58 yes yes yes 0.6 0.13 0.13 0.13 combined
2015 June R64 4 humus_mineral Araneae Thomisidae Ozyptila_sp 1 2.78 yes yes no 0 1 0 0 predator
2015 June R64 4 mineral Coleoptera Elateridae Eanus_costalis 1 3.15 yes yes no 0 0.5 0.5 0 combined
2015 June R64 5 humus Coleoptera Elateridae Eanus_costalis 1 0.71 yes yes no 0 0.5 0.5 0 combined
2015 June R64 5 humus Coleoptera Cantharidae . 1 0.11 yes yes no 0 1 0 0 predator
2015 June R64 5 humus Coleoptera Cantharidae Malthodes_brevicollis 1 0.15 yes yes no 0 1 0 0 predator
2015 June R64 5 mineral Coleoptera Elateridae Paraphotistus_impressus 1 14.41 yes yes yes 0.6 0.13 0.13 0.13 combined
2015 June R64 6 humus Annelida Lumbricidae Dendrobaena_octaedra 1 12.24 yes yes no 0 0 1 0 detritivore
2015 June R64 6 humus Diptera Rhagionidae Rhagio_scolopaceus 1 8.37 yes yes no 0 1 0 0 predator
2015 June R64 7 humus Coleoptera Elateridae Athous_subfuscus 1 1.46 yes yes yes 0 0.8 0.1 0.1 combined
2015 June R64 7 humus Coleoptera Elateridae Paraphotistus_impressus 2 54.78 yes yes yes 0.6 0.13 0.13 0.13 combined
2015 June R64 7 humus_mineral Myriapoda Lithobiidae Lithobius_curtipes 1 1.59 yes yes no 0 1 0 0 predator
2015 June R64 8 humus Coleoptera Elateridae Athous_subfuscus 4 5.81 yes yes yes 0 0.8 0.1 0.1 combined
2015 June R64 8 humus Coleoptera Cantharidae . 1 0.08 yes yes no 0 1 0 0 predator
2015 June R64 8 humus Coleoptera Polyphaga . 1 0.49 . . . .
2015 June R64 8 humus_mineral Araneae Clubionidae Clubiona_sp 1 0.00 yes yes no 0 1 0 0 predator
2015 June R64 8 humus_mineral Araneae Theridiidae Robertus_scoticus 3 0.87 yes yes no 0 1 0 0 predator
2015 June R64 8 mineral Coleoptera Elateridae Paraphotistus_impressus 1 5.18 yes yes yes 0.6 0.13 0.13 0.13 combined
2015 June R64 9 humus Coleoptera Leiodidae Amphicyllis_globus 1 0.73 yes yes no 0 0 0 1 fungivore
2015 June R64 9 humus Coleoptera Elateridae Athous_subfuscus 4 10.77 yes yes yes 0 0.8 0.1 0.1 combined
2015 June R64 9 humus_mineral Myriapoda Lithobiidae Lithobius_curtipes 1 0.62 yes yes no 0 1 0 0 predator
2015 June R64 9 humus_mineral Araneae Theridiidae Robertus_sp 1 0.12 yes yes no 0 1 0 0 predator
2015 June R64 10 humus Annelida Lumbricidae Dendrobaena_octaedra 1 49.20 yes yes no 0 0 1 0 detritivore
2015 June R64 10 humus Coleoptera Elateridae Eanus_costalis 1 0.35 yes yes no 0 0.5 0.5 0 combined
2015 June R64 10 humus Coleoptera Curculionidae Polydrusus_fulvicornis 1 1.29 yes yes yes 1 0 0 0 herbivore
2015 June R64 10 humus Hemiptera Lygaeidae Drymus_brunneus 1 0.99 yes yes no 1 0 0 0 herbivore
2015 June R64 10 humus_mineral Araneae Linyphiidae Porrhomma_pallidum 1 0.00 yes yes no 0 1 0 0 predator
2015 June R64 10 humus_mineral Araneae Theridiidae Robertus_scoticus 1 1.00 yes yes no 0 1 0 0 predator
2015 June R64 10 humus_mineral Araneae Theridiidae Robertus_sp 2 0.00 yes yes no 0 1 0 0 predator
2015 June R65 1 humus Diptera Lauxaniidae Homoneura_sp 1 0.67 yes yes no 0 0 1 0 detritivore
2015 June R65 1 humus Diptera Lauxaniidae Homoneura_sp 1 0.80 yes yes no 0 0 1 0 detritivore
2015 June R65 1 humus Annelida Lumbricidae Dendrodrilus_rubidus_tenuis 1 29.76 yes yes no 0 0 1 0 detritivore
2015 June R65 1 humus Coleoptera Elateridae Ampedus_cf_balteatus 1 0.89 yes yes no 0 0.5 0.5 0 combined
2015 June R65 1 humus Coleoptera Elateridae Eanus_costalis 1 1.20 yes yes no 0 0.5 0.5 0 combined
2015 June R65 1 humus Coleoptera Elateridae Athous_subfuscus 1 0.97 yes yes yes 0 0.8 0.1 0.1 combined
2015 June R65 1 humus Coleoptera . . 1 0.13 . . . .
2015 June R65 1 humus_mineral Araneae Linyphiidae Gen_sp 2 0.00 yes yes no 0 1 0 0 predator
2015 June R65 1 humus_mineral Araneae Linyphiidae Tapinocyba_insecta 2 0.00 yes yes no 0 1 0 0 predator
2015 June R65 1 humus_mineral Araneae Lycosidae Gen_sp 1 1.72 yes yes no 0 1 0 0 predator
2015 June R65 1 humus_mineral Araneae Theridiidae Robertus_scoticus 2 0.00 yes yes no 0 1 0 0 predator
2015 June R65 1 mineral Coleoptera Elateridae Eanus_costalis 1 5.25 yes yes no 0 0.5 0.5 0 combined

2015 June R65 1 mineral Coleoptera Elateridae Athous_subfuscus 1 0.68 yes yes yes 0 0.8 0.1 0.1 combined
2015 June R65 1 mineral Coleoptera Elateridae Paraphotistus_impressus 1 4.43 yes yes yes 0.6 0.13 0.13 0.13 combined
2015 June R65 1 mineral Coleoptera Curculionidae Polydrusus_tereticollis 1 4.17 yes yes yes 1 0 0 0 herbivore
2015 June R65 2 humus Annelida Lumbricidae Dendrobaena_octaedra 1 1.16 yes yes no 0 0 1 0 detritivore
2015 June R65 2 humus Annelida Lumbricidae Dendrobaena_octaedra 3 44.31 yes yes no 0 0 1 0 detritivore
2015 June R65 2 humus_mineral Araneae Thomisidae Ozyptila_sp 1 0.33 yes yes no 0 1 0 0 predator
2015 June R65 2 mineral Coleoptera Elateridae Paraphotistus_impressus 1 2.89 yes yes yes 0.6 0.13 0.13 0.13 combined
2015 June R65 2 mineral Coleoptera Curculionidae Polydrusus_tereticollis 1 12.98 yes yes yes 1 0 0 0 herbivore
2015 June R65 3 humus Coleoptera Elateridae Eanus_costalis 1 4.54 yes yes no 0 0.5 0.5 0 combined
2015 June R65 3 humus_mineral Hymenoptera Formicidae Myrmica_ruginodis 1 0.71 Yes Yes no 0 1 0 0 predator
2015 June R65 3 humus_mineral Araneae Linyphiidae Gen_sp 1 0.00 yes yes no 0 1 0 0 predator
2015 June R65 3 humus_mineral Araneae Linyphiidae Tapinocyba_insecta 1 0.00 yes yes no 0 1 0 0 predator
2015 June R65 3 humus_mineral Coleoptera Staphylinidae Drusilla_canaliculata 1 0.90 yes yes no 0 1 0 0 predator
2015 June R65 3 humus_mineral Coleoptera Staphylinidae Othius_subuliformis 1 0.68 yes yes no 0 1 0 0 predator
2015 June R65 3 humus_mineral Araneae Theridiidae Robertus_scoticus 1 0.49 yes yes no 0 1 0 0 predator
2015 June R65 4 humus Annelida Lumbricidae Dendrodrilus_rubidus_tenuis 1 18.48 yes yes no 0 0 1 0 detritivore
2015 June R65 4 humus Coleoptera Elateridae Eanus_costalis 2 2.96 yes yes no 0 0.5 0.5 0 combined
2015 June R65 4 humus Coleoptera Elateridae Paraphotistus_impressus 1 5.59 yes yes yes 0.6 0.13 0.13 0.13 combined
2015 June R65 4 humus_mineral Araneae Linyphiidae Gen_sp 1 1.30 yes yes no 0 1 0 0 predator
2015 June R65 4 humus_mineral Araneae Linyphiidae Tapinocyba_insecta 1 0.00 yes yes no 0 1 0 0 predator
2015 June R65 4 humus_mineral Myriapoda Lithobiidae Lithobius_curtipes 1 0.48 yes yes no 0 1 0 0 predator
2015 June R65 4 humus_mineral Araneae Thomisidae Ozyptila_sp 1 0.00 yes yes no 0 1 0 0 predator
2015 June R65 5 humus Coleoptera Elateridae Ampedus_sp 1 1.41 yes yes no 0 0.5 0.5 0 combined
2015 June R65 5 humus Coleoptera Elateridae Eanus_costalis 4 21.72 yes yes no 0 0.5 0.5 0 combined
2015 June R65 5 humus_mineral Araneae Theridiidae Robertus_sp 1 0.05 yes yes no 0 1 0 0 predator
2015 June R65 5 mineral Coleoptera Elateridae Eanus_costalis 1 6.05 yes yes no 0 0.5 0.5 0 combined
2015 June R65 6 humus Annelida Lumbricidae Dendrobaena_octaedra 2 8.35 yes yes no 0 0 1 0 detritivore
2015 June R65 6 humus Coleoptera Elateridae Eanus_costalis 2 3.29 yes yes no 0 0.5 0.5 0 combined
2015 June R65 6 humus Coleoptera Elateridae Athous_subfuscus 1 2.11 yes yes yes 0 0.8 0.1 0.1 combined
2015 June R65 6 humus Coleoptera Cantharidae Podistra_schoenherri 1 1.18 yes yes no 0 1 0 0 predator
2015 June R65 6 humus_mineral Coleoptera Staphylinidae Bolitochara_pulchra 1 0.42 yes yes no 0 1 0 0 predator
2015 June R65 7 humus Annelida Lumbricidae Dendrobaena_octaedra 1 66.00 yes yes no 0 0 1 0 detritivore
2015 June R65 7 humus Coleoptera Elateridae Athous_subfuscus 3 12.31 yes yes yes 0 0.8 0.1 0.1 combined
2015 June R65 7 humus_mineral Hymenoptera Formicidae Myrmica_ruginodis 5 3.42 Yes Yes no 0 1 0 0 predator
2015 June R65 7 humus_mineral Araneae Linyphiidae Centromerus_arcanus 2 0.00 yes yes no 0 1 0 0 predator
2015 June R65 7 humus_mineral Araneae Linyphiidae Gen_sp 4 0.70 yes yes no 0 1 0 0 predator
2015 June R65 7 humus_mineral Araneae Linyphiidae Tapinocyba_pallens 1 0.00 yes yes no 0 1 0 0 predator
2015 June R65 7 humus_mineral Myriapoda Lithobiidae Lithobius_curtipes 1 0.56 yes yes no 0 1 0 0 predator
2015 June R65 7 mineral Coleoptera Elateridae Athous_subfuscus 1 3.52 yes yes yes 0 0.8 0.1 0.1 combined
2015 June R65 7 mineral Coleoptera Scirtidae Cyphon_padi 1 0.15 no no no 0.5 0 0.5 0 combined
2015 June R65 8 humus Diptera Chironomidae Bryophaenocladius_sp 1 0.05 yes yes yes 0.25 0 0.75 0 combined
2015 June R65 8 humus_mineral Coleoptera Carabidae Calathus_micropterus 1 5.99 yes yes no 0 1 0 0 predator
2015 June R65 8 humus_mineral Hymenoptera Formicidae Myrmica_ruginodis 3 1.60 Yes Yes no 0 1 0 0 predator
2015 June R65 8 humus_mineral Araneae Linyphiidae Gen_sp 1 0.00 yes yes no 0 1 0 0 predator
2015 June R65 8 humus_mineral Myriapoda Lithobiidae Lithobius_curtipes 1 0.41 yes yes no 0 1 0 0 predator
2015 June R65 8 humus_mineral Myriapoda Lithobiidae Lithobius_curtipes 1 0.48 yes yes no 0 1 0 0 predator
2015 June R65 8 humus_mineral Coleoptera Staphylinidae Drusilla_canaliculata 1 0.90 yes yes no 0 1 0 0 predator
2015 June R65 8 humus_mineral Coleoptera Staphylinidae Drusilla_canaliculata 1 0.91 yes yes no 0 1 0 0 predator
2015 June R65 8 humus_mineral Araneae Theridiidae Robertus_sp 1 0.28 yes yes no 0 1 0 0 predator
2015 June R65 8 mineral Coleoptera Elateridae Paraphotistus_impressus 1 2.66 yes yes yes 0.6 0.13 0.13 0.13 combined
2015 June R65 9 humus Annelida Lumbricidae Dendrobaena_octaedra 1 38.11 yes yes no 0 0 1 0 detritivore
2015 June R65 9 humus Coleoptera Elateridae Eanus_costalis 1 2.24 yes yes no 0 0.5 0.5 0 combined
2015 June R65 9 humus Coleoptera Elateridae Athous_subfuscus 1 3.58 yes yes yes 0 0.8 0.1 0.1 combined

2015 June R65 9 humus_mineral Araneae Lycosidae Pardosa_sp 1 3.32 yes yes no 0 1 0 0 predator
2015 June R65 9 humus_mineral Coleoptera Staphylinidae Othius_lapidicola 1 0.90 yes yes no 0 1 0 0 predator
2015 June R65 9 humus_mineral Araneae Thomisidae Ozyptila_trux 1 0.00 yes yes no 0 1 0 0 predator
2015 June R65 9 mineral Coleoptera Elateridae Ampedus_nigrinus 1 1.37 yes yes no 0 0.5 0.5 0 combined
2015 June R65 9 mineral Coleoptera Elateridae Athous_subfuscus 1 2.90 yes yes yes 0 0.8 0.1 0.1 combined
2015 June R65 10 humus Coleoptera Elateridae Eanus_costalis 1 0.98 yes yes no 0 0.5 0.5 0 combined
2015 June R65 10 humus Coleoptera Curculionidae Otiorhynchus_nodosus 1 7.08 yes yes yes 1 0 0 0 herbivore
2015 June R65 10 humus_mineral Myriapoda Lithobiidae Lithobius_curtipes 1 1.27 yes yes no 0 1 0 0 predator
2015 June R65 10 humus_mineral Coleoptera Staphylinidae Atheta_myrmecobia 1 0.09 yes yes no 0 1 0 0 predator
2015 June R65 10 humus_mineral Coleoptera Staphylinidae Atheta_myrmecobia 1 0.09 yes yes no 0 1 0 0 predator
2015 June R65 10 humus_mineral Coleoptera Staphylinidae Atheta_myrmecobia 1 0.10 yes yes no 0 1 0 0 predator
2015 June R65 10 mineral Coleoptera Curculionidae . 1 5.22 yes yes yes 1 0 0 0 herbivore
2015 June R66 1 humus Araneae Linyphiidae Gen_sp 1 0.08 yes yes no 0 1 0 0 predator
2015 June R66 1 humus_mineral Araneae Linyphiidae Microneta_viaria 1 0.00 yes yes no 0 1 0 0 predator
2015 June R66 1 humus_mineral Myriapoda Lithobiidae Lithobius_curtipes 1 0.78 yes yes no 0 1 0 0 predator
2015 June R66 1 humus_mineral Araneae Salticidae Neon_reticulatus 1 0.97 yes yes no 0 1 0 0 predator
2015 June R66 2 humus Coleoptera Elateridae Eanus_costalis 1 1.54 yes yes no 0 0.5 0.5 0 combined
2015 June R66 2 humus Coleoptera Elateridae Athous_subfuscus 1 0.63 yes yes yes 0 0.8 0.1 0.1 combined
2015 June R66 2 humus Coleoptera Staphylinidae Drusilla_canaliculata 1 0.19 yes yes no 0 1 0 0 predator
2015 June R66 2 humus Coleoptera . . 1 0.11 yes yes . . . . . .
2015 June R66 2 humus_mineral Myriapoda Lithobiidae Lithobius_curtipes 1 0.48 yes yes no 0 1 0 0 predator
2015 June R66 3 humus_mineral Hymenoptera Formicidae Myrmica_ruginodis 1 0.66 Yes Yes no 0 1 0 0 predator
2015 June R66 3 humus_mineral Araneae Theridiidae Robertus_scoticus 1 0.39 yes yes no 0 1 0 0 predator
2015 June R66 4 humus Coleoptera Elateridae Paraphotistus_impressus 1 8.30 yes yes yes 0.6 0.13 0.13 0.13 combined
2015 June R66 5 humus Annelida Lumbricidae Dendrobaena_octaedra 2 22.08 yes yes no 0 0 1 0 detritivore
2015 June R66 5 humus Coleoptera Elateridae Athous_subfuscus 2 3.31 yes yes yes 0 0.8 0.1 0.1 combined
2015 June R66 5 humus_mineral Araneae Lycosidae Pirata_Piratula_sp 1 0.70 yes yes no 0 1 0 0 predator
2015 June R66 5 humus_mineral Coleoptera Staphylinidae Oxypoda_annularis 1 0.08 yes yes no 0 1 0 0 predator
2015 June R66 6 humus Coleoptera Elateridae Athous_subfuscus 1 1.18 yes yes yes 0 0.8 0.1 0.1 combined
2015 June R66 6 humus Hemiptera Lygaeidae Drymus_brunneus 1 0.25 yes yes no 1 0 0 0 herbivore
2015 June R66 6 humus_mineral Araneae Linyphiidae Tapinocyba_pallens 1 0.72 yes yes no 0 1 0 0 predator
2015 June R66 6 humus_mineral Araneae Linyphiidae Tibioplus_diversus 2 0.00 yes yes no 0 1 0 0 predator
2015 June R66 6 humus_mineral Myriapoda Lithobiidae Lithobius_curtipes 1 1.67 yes yes no 0 1 0 0 predator
2015 June R66 6 humus_mineral Myriapoda Lithobiidae Lithobius_sp 1 0.90 yes yes no 0 1 0 0 predator
2015 June R66 6 humus_mineral Coleoptera Staphylinidae Othius_lapidicola 1 0.63 yes yes no 0 1 0 0 predator
2015 June R66 6 humus_mineral Araneae Theridiidae Robertus_sp 1 0.00 yes yes no 0 1 0 0 predator
2015 June R66 7 humus Annelida Lumbricidae Dendrobaena_octaedra 1 9.84 yes yes no 0 0 1 0 detritivore
2015 June R66 7 humus Coleoptera Elateridae Eanus_costalis 1 1.15 yes yes no 0 0.5 0.5 0 combined
2015 June R66 7 humus Coleoptera Elateridae Athous_subfuscus 1 2.26 yes yes yes 0 0.8 0.1 0.1 combined
2015 June R66 7 humus Coleoptera Cantharidae Podistra_schoenherri 1 0.58 yes yes no 0 1 0 0 predator
2015 June R66 7 humus Coleoptera Cantharidae Podistra_schoenherri 1 4.87 yes yes no 0 1 0 0 predator
2015 June R66 7 humus Myriapoda Lithobiidae Lithobius_curtipes 1 1.32 yes yes no 0 1 0 0 predator
2015 June R66 7 humus Myriapoda Lithobiidae Lithobius_curtipes 1 1.45 yes yes no 0 1 0 0 predator
2015 June R66 7 humus Coleoptera Staphylinidae Ischnosoma_splendidum 1 0.28 yes yes no 0 1 0 0 predator
2015 June R66 7 humus Coleoptera Staphylinidae Ischnosoma_splendidum 1 0.32 yes yes no 0 1 0 0 predator
2015 June R66 8 humus Coleoptera Elateridae Eanus_costalis 1 4.54 yes yes no 0 0.5 0.5 0 combined
2015 June R66 8 mineral Coleoptera Elateridae Eanus_costalis 1 8.29 yes yes no 0 0.5 0.5 0 combined
2015 June R66 8 mineral Coleoptera Elateridae Athous_subfuscus 1 2.70 yes yes yes 0 0.8 0.1 0.1 combined
2015 June R66 9 humus Coleoptera Elateridae Athous_subfuscus 2 4.47 yes yes yes 0 0.8 0.1 0.1 combined
2015 June R66 9 humus Coleoptera Cantharidae Podistra_schoenherri 1 0.44 yes yes no 0 1 0 0 predator
2015 June R66 9 humus Coleoptera Staphylinidae Oxypoda_formiceticola 1 0.07 yes yes no 0 1 0 0 predator
2015 June R66 9 mineral Coleoptera Curculionidae Polydrusus_tereticollis 1 9.45 yes yes yes 1 0 0 0 herbivore
2015 June R66 10 humus Annelida Lumbricidae Dendrobaena_octaedra 1 20.40 yes yes no 0 0 1 0 detritivore

2015 June R66 10 humus_mineral Araneae Gnaphosidae Haplodrassus_sp 1 0.53 yes yes no 0 1 0 0 predator
2015 June R67 1 humus Annelida Lumbricidae Eiseniella_tetraedra 1 0.29 yes yes no 0 0 1 0 detritivore
2015 June R67 1 humus Coleoptera Elateridae Eanus_costalis 1 1.31 yes yes no 0 0.5 0.5 0 combined
2015 June R67 1 humus_mineral Araneae Linyphiidae Gen_sp 2 0.00 yes yes no 0 1 0 0 predator
2015 June R67 1 humus_mineral Araneae Linyphiidae Minyriolus_pusillus 1 0.23 yes yes no 0 1 0 0 predator
2015 June R67 1 humus_mineral Coleoptera Staphylinidae Oxypoda_annularis 1 0.08 yes yes no 0 1 0 0 predator
2015 June R67 2 humus Diptera Lauxaniidae Homoneura_sp 1 1.44 yes yes no 0 0 1 0 detritivore
2015 June R67 2 humus Diptera Lauxaniidae Homoneura_sp 1 1.51 yes yes no 0 0 1 0 detritivore
2015 June R67 2 humus Diptera Lauxaniidae Homoneura_sp 1 1.58 yes yes no 0 0 1 0 detritivore
2015 June R67 2 humus Diptera Lauxaniidae Homoneura_sp 1 1.95 yes yes no 0 0 1 0 detritivore
2015 June R67 2 humus Annelida Lumbricidae Eiseniella_tetraedra 1 1.04 yes yes no 0 0 1 0 detritivore
2015 June R67 2 humus Coleoptera Elateridae Athous_subfuscus 4 3.10 yes yes yes 0 0.8 0.1 0.1 combined
2015 June R67 2 humus Coleoptera Cantharidae Podistra_schoenherri 1 1.76 yes yes no 0 1 0 0 predator
2015 June R67 2 humus_mineral Hymenoptera Formicidae Myrmica_ruginodis 1 0.55 Yes Yes no 0 1 0 0 predator
2015 June R67 2 humus_mineral Araneae Linyphiidae Centromerus_arcanus 1 0.00 yes yes no 0 1 0 0 predator
2015 June R67 2 humus_mineral Araneae Linyphiidae Dismodicus_elevatus 1 0.00 yes yes no 0 1 0 0 predator
2015 June R67 2 humus_mineral Araneae Linyphiidae Gen_sp 4 0.00 yes yes no 0 1 0 0 predator
2015 June R67 2 humus_mineral Araneae Linyphiidae Pocadicnemis_pumila 1 0.00 yes yes no 0 1 0 0 predator
2015 June R67 2 humus_mineral Araneae Linyphiidae Tapinocyba_pallens 1 0.00 yes yes no 0 1 0 0 predator
2015 June R67 2 humus_mineral Araneae Theridiidae Robertus_sp 1 0.00 yes yes no 0 1 0 0 predator
2015 June R67 2 humus_mineral Araneae Thomisidae Ozyptila_sp 1 4.27 yes yes no 0 1 0 0 predator
2015 June R67 2 mineral Coleoptera Elateridae Athous_subfuscus 1 4.23 yes yes yes 0 0.8 0.1 0.1 combined
2015 June R67 2 mineral Coleoptera Elateridae Paraphotistus_impressus 1 5.53 yes yes yes 0.6 0.13 0.13 0.13 combined
2015 June R67 2 mineral Hemiptera Cicadellidae Bathysmatophorus_reuteri 1 2.41 no no no 1 0 0 0 herbivore
2015 June R67 3 humus_mineral Hymenoptera Formicidae Myrmica_ruginodis 1 0.71 Yes Yes no 0 1 0 0 predator
2015 June R67 3 humus_mineral Araneae Linyphiidae Neriene_clathrata 1 0.00 yes yes no 0 1 0 0 predator
2015 June R67 3 humus_mineral Araneae Linyphiidae Pityohyphantes_phrygianus 1 6.33 yes yes no 0 1 0 0 predator
2015 June R67 3 humus_mineral Araneae Linyphiidae Tapinocyba_pallens 2 0.00 yes yes no 0 1 0 0 predator
2015 June R67 3 humus_mineral Myriapoda Lithobiidae Lithobius_cf_curtipes 1 0.54 yes yes no 0 1 0 0 predator
2015 June R67 3 humus_mineral Coleoptera Staphylinidae Atheta_myrmecobia 1 0.11 yes yes no 0 1 0 0 predator
2015 June R67 3 humus_mineral Coleoptera Staphylinidae Atheta_myrmecobia 1 0.12 yes yes no 0 1 0 0 predator
2015 June R67 3 humus_mineral Coleoptera Staphylinidae Atheta_myrmecobia 1 0.12 yes yes no 0 1 0 0 predator
2015 June R67 3 humus_mineral Coleoptera Staphylinidae Ischnosoma_sp 1 0.42 yes yes no 0 1 0 0 predator
2015 June R67 3 humus_mineral Coleoptera Staphylinidae Mycetoporus_monticola 1 0.30 yes yes no 0 1 0 0 predator
2015 June R67 3 humus_mineral Araneae Theridiidae Robertus_scoticus 3 0.00 yes yes no 0 1 0 0 predator
2015 June R67 3 humus_mineral Araneae Theridiidae Robertus_sp 1 0.00 yes yes no 0 1 0 0 predator
2015 June R67 4 humus Annelida Lumbricidae Dendrobaena_octaedra 1 0.33 yes yes no 0 0 1 0 detritivore
2015 June R67 4 humus Coleoptera Elateridae Eanus_costalis 1 3.69 yes yes no 0 0.5 0.5 0 combined
2015 June R67 4 humus Coleoptera Elateridae Athous_subfuscus 2 3.86 yes yes yes 0 0.8 0.1 0.1 combined
2015 June R67 4 humus_mineral Araneae Linyphiidae Neriene_sp 1 0.00 yes yes no 0 1 0 0 predator
2015 June R67 4 humus_mineral Coleoptera Staphylinidae Oxypoda_annularis 1 0.11 yes yes no 0 1 0 0 predator
2015 June R67 4 humus_mineral Araneae Theridiidae Robertus_sp 1 0.62 yes yes no 0 1 0 0 predator
2015 June R67 5 humus Annelida Lumbricidae Eiseniella_tetraedra 2 0.52 yes yes no 0 0 1 0 detritivore
2015 June R67 5 humus Coleoptera Elateridae Athous_subfuscus 1 1.20 yes yes yes 0 0.8 0.1 0.1 combined
2015 June R67 6 humus Coleoptera Elateridae Eanus_costalis 1 10.50 yes yes no 0 0.5 0.5 0 combined
2015 June R67 6 humus_mineral Araneae Linyphiidae Gen_sp 3 0.00 yes yes no 0 1 0 0 predator
2015 June R67 6 humus_mineral Araneae Linyphiidae Minyriolus_pusillus 5 0.00 yes yes no 0 1 0 0 predator
2015 June R67 6 humus_mineral Araneae Lycosidae Pardosa_sp 1 2.10 yes yes no 0 1 0 0 predator
2015 June R67 6 humus_mineral Araneae Theridiidae Robertus_scoticus 2 0.00 yes yes no 0 1 0 0 predator
2015 June R67 6 humus_mineral Araneae Theridiidae Robertus_sp 1 0.00 yes yes no 0 1 0 0 predator
2015 June R67 7 humus Diptera Trichoceridae Trichocera_annulata 1 1.24 yes yes no 0 0 1 0 detritivore
2015 June R67 7 humus Coleoptera Elateridae Eanus_costalis 2 4.85 yes yes no 0 0.5 0.5 0 combined
2015 June R67 7 humus Coleoptera Elateridae Athous_subfuscus 3 4.67 yes yes yes 0 0.8 0.1 0.1 combined

2015 June R67 7 humus_mineral Hymenoptera Formicidae Myrmica_ruginodis 1 0.63 Yes Yes no 0 1 0 0 predator
2015 June R67 7 humus_mineral Araneae Linyphiidae Tapinocyba_pallens 2 0.00 yes yes no 0 1 0 0 predator
2015 June R67 7 humus_mineral Coleoptera Staphylinidae Oxypoda_annularis 1 0.08 yes yes no 0 1 0 0 predator
2015 June R67 7 humus_mineral Araneae Theridiidae Robertus_scoticus 2 1.40 yes yes no 0 1 0 0 predator
2015 June R67 7 humus_mineral Araneae Theridiidae Robertus_sp 2 0.00 yes yes no 0 1 0 0 predator
2015 June R67 8 humus Coleoptera Elateridae Eanus_costalis 1 1.33 yes yes no 0 0.5 0.5 0 combined
2015 June R67 8 humus Coleoptera Elateridae Athous_subfuscus 2 1.89 yes yes yes 0 0.8 0.1 0.1 combined
2015 June R67 8 humus Diptera . . 1 0.46 . . . .
2015 June R67 8 humus_mineral Araneae Linyphiidae Gen_sp 1 0.00 yes yes no 0 1 0 0 predator
2015 June R67 8 humus_mineral Araneae Theridiidae Robertus_scoticus 1 0.41 yes yes no 0 1 0 0 predator
2015 June R67 8 mineral Coleoptera Elateridae Eanus_costalis 1 1.81 yes yes no 0 0.5 0.5 0 combined
2015 June R67 9 humus Annelida Lumbricidae Dendrobaena_octaedra 1 1.14 yes yes no 0 0 1 0 detritivore
2015 June R67 9 humus Coleoptera Elateridae Athous_subfuscus 1 3.95 yes yes yes 0 0.8 0.1 0.1 combined
2015 June R67 9 humus Coleoptera Staphylinidae Othius_lapidicola 1 1.08 yes yes no 0 1 0 0 predator
2015 June R67 9 humus Coleoptera Elateridae Paraphotistus_impressus 1 4.15 yes yes yes 0.6 0.13 0.13 0.13 combined
2015 June R67 9 humus Hemiptera Lygaeidae Drymus_brunneus 1 0.69 yes yes no 1 0 0 0 herbivore
2015 June R67 9 humus_mineral Araneae Linyphiidae Minyriolus_pusillus 1 0.00 yes yes no 0 1 0 0 predator
2015 June R67 9 humus_mineral Myriapoda Lithobiidae Lithobius_curtipes 1 1.47 yes yes no 0 1 0 0 predator
2015 June R67 9 humus_mineral Araneae Theridiidae Robertus_scoticus 2 0.86 yes yes no 0 1 0 0 predator
2015 June R67 10 humus Annelida Lumbricidae Eiseniella_tetraedra 1 0.25 yes yes no 0 0 1 0 detritivore
2015 June R67 10 humus Coleoptera Elateridae Paraphotistus_impressus 1 4.22 yes yes yes 0.6 0.13 0.13 0.13 combined
2015 June R67 10 humus Coleoptera . . 1 0.34 . . . .
2015 June R67 10 humus_mineral Araneae Linyphiidae Gen_sp 1 0.00 yes yes no 0 1 0 0 predator
2015 June R67 10 humus_mineral Araneae Linyphiidae Minyriolus_pusillus 1 0.00 yes yes no 0 1 0 0 predator
2015 June R67 10 humus_mineral Araneae Linyphiidae Tapinocyba_pallens 2 2.86 yes yes no 0 1 0 0 predator
2015 June R67 10 humus_mineral Myriapoda Lithobiidae Lithobius_curtipes 1 2.08 yes yes no 0 1 0 0 predator
2015 June R67 10 humus_mineral Coleoptera Staphylinidae Oxypoda_annularis 1 0.09 yes yes no 0 1 0 0 predator
2015 June R67 10 humus_mineral Araneae Theridiidae Robertus_scoticus 2 0.00 yes yes no 0 1 0 0 predator
2015 June R67 10 humus_mineral Araneae Theridiidae Robertus_sp 2 0.00 yes yes no 0 1 0 0 predator
2015 June R68 1 humus Araneae Dictynidae Gen_sp 1 0.09 yes yes no 0 1 0 0 predator
2015 June R68 1 humus Diptera Therevidae Thereva_fuscinervis 1 24.07 yes yes no 0 1 0 0 predator
2015 June R68 3 humus_mineral Myriapoda Lithobiidae Lithobius_sp 1 0.14 yes yes no 0 1 0 0 predator
2015 June R68 3 humus_mineral Araneae Theridiidae Robertus_scoticus 1 0.00 yes yes no 0 1 0 0 predator
2015 June R68 3 humus_mineral Araneae Theridiidae Robertus_scoticus 1 0.57 yes yes no 0 1 0 0 predator
2015 June R68 4 humus_mineral Araneae Theridiidae Gen_sp 1 0.04 yes yes no 0 1 0 0 predator
2015 June R68 5 humus Hemiptera Lygaeidae Eremocoris_plebejus 1 1.86 yes no no 1 0 0 0 herbivore
2015 June R68 5 humus_mineral Araneae Linyphiidae Gen_sp 1 0.00 yes yes no 0 1 0 0 predator
2015 June R68 5 humus_mineral Araneae Theridiidae Robertus_scoticus 2 0.00 yes yes no 0 1 0 0 predator
2015 June R68 5 humus_mineral Araneae Theridiidae Robertus_scoticus 1 0.85 yes yes no 0 1 0 0 predator
2015 June R68 6 humus Coleoptera Cantharidae . 1 0.31 yes yes no 0 1 0 0 predator
2015 June R68 6 humus Coleoptera Cantharidae Malthodes_mysticus 1 1.15 yes yes no 0 1 0 0 predator
2015 June R68 6 humus_mineral Hymenoptera Formicidae Myrmica_sulcinodis 1 0.51 Yes Yes no 0 0.8 0.2 0 combined
2015 June R68 6 humus_mineral Hymenoptera Formicidae Myrmica_ruginodis 1 0.63 Yes Yes no 0 1 0 0 predator
2015 June R68 6 humus_mineral Araneae Gnaphosidae Haplodrassus_sp 1 0.89 yes yes no 0 1 0 0 predator
2015 June R68 6 humus_mineral Myriapoda Lithobiidae Lithobius_curtipes 1 0.52 yes yes no 0 1 0 0 predator
2015 June R68 7 humus_mineral Hymenoptera Formicidae Leptothorax_acervorum 1 0.19 Yes Yes no 0 0.8 0.2 0 combined
2015 June R68 7 humus_mineral Hymenoptera Formicidae Myrmica_ruginodis 1 0.91 Yes Yes no 0 1 0 0 predator
2015 June R68 7 humus_mineral Araneae Gnaphosidae Haplodrassus_soerenseni 1 9.22 yes yes no 0 1 0 0 predator
2015 June R68 7 humus_mineral Araneae Linyphiidae Agyneta_conigera 1 0.00 yes yes no 0 1 0 0 predator
2015 June R68 7 humus_mineral Coleoptera Staphylinidae Oxypoda_annularis 1 0.09 yes yes no 0 1 0 0 predator
2015 June R68 7 mineral Hemiptera Lygaeidae Eremocoris_plebejus 1 2.33 yes no no 1 0 0 0 herbivore
2015 June R68 8 humus_mineral Hymenoptera Formicidae Myrmica_ruginodis 2 1.26 Yes Yes no 0 1 0 0 predator
2015 June R68 8 humus_mineral Araneae Gnaphosidae Haplodrassus_sp 1 1.68 yes yes no 0 1 0 0 predator

2015 June R68 8 humus_mineral Myriapoda Lithobiidae Lithobius_curtipes 1 1.12 yes yes no 0 1 0 0 predator
2015 June R68 9 humus_mineral Araneae Gnaphosidae Haplodrassus_sp 1 9.22 yes yes no 0 1 0 0 predator
2015 June R68 9 humus_mineral Myriapoda Lithobiidae Lithobius_sp 1 0.23 yes yes no 0 1 0 0 predator
2015 June R68 10 humus_mineral Hymenoptera Formicidae Myrmica_sulcinodis 1 0.38 Yes Yes no 0 0.8 0.2 0 combined
2015 June R68 10 humus_mineral Hymenoptera Formicidae Myrmica_ruginodis 1 0.63 Yes Yes no 0 1 0 0 predator
2015 June R68 10 humus_mineral Myriapoda Lithobiidae Lithobius_curtipes 1 1.27 yes yes no 0 1 0 0 predator
2015 June R68 10 humus_mineral Myriapoda Lithobiidae Lithobius_curtipes 1 1.66 yes yes no 0 1 0 0 predator
2015 June R68 10 mineral Coleoptera Curculionidae Polydrusus_fulvicornis 1 2.04 yes yes yes 1 0 0 0 herbivore
2015 June R69 1 humus Coleoptera Elateridae Eanus_costalis 1 8.19 yes yes no 0 0.5 0.5 0 combined
2015 June R69 1 humus Diptera Empididae Phyllodromia_melanocephala 1 0.65 yes yes no 0 1 0 0 predator
2015 June R69 1 humus_mineral Diptera Bibionidae Bibio_pomonae 1 59.24 yes yes no 0 0 1 0 detritivore
2015 June R69 1 humus_mineral Coleoptera Carabidae Patrobus_assimilis 1 5.31 yes yes no 0 1 0 0 predator
2015 June R69 1 humus_mineral Araneae Linyphiidae Gen_sp 2 0.86 yes yes no 0 1 0 0 predator
2015 June R69 1 humus_mineral Araneae Linyphiidae Oryphantes_angulatus 1 0.00 yes yes no 0 1 0 0 predator
2015 June R69 1 humus_mineral Coleoptera Staphylinidae Stenus_sp 1 0.28 yes yes no 0 1 0 0 predator
2015 June R69 1 humus_mineral Coleoptera Staphylinidae Stenus_sp 1 0.30 yes yes no 0 1 0 0 predator
2015 June R69 1 humus_mineral Araneae Theridiidae Robertus_sp 1 0.00 yes yes no 0 1 0 0 predator
2015 June R69 1 humus_mineral Coleoptera Curculionidae Otiorhynchus_nodosus 1 2.69 yes yes yes 1 0 0 0 herbivore
2015 June R69 1 mineral Diptera Rhagionidae Rhagio_scolopaceus 1 2.71 yes yes no 0 1 0 0 predator
2015 June R69 2 humus Annelida Lumbricidae Dendrobaena_octaedra 1 17.40 yes yes no 0 0 1 0 detritivore
2015 June R69 2 humus Coleoptera Elateridae Eanus_costalis 2 4.24 yes yes no 0 0.5 0.5 0 combined
2015 June R69 2 humus Coleoptera Cantharidae Podistra_schoenherri 1 0.74 yes yes no 0 1 0 0 predator
2015 June R69 2 humus Coleoptera Cantharidae Podistra_schoenherri 1 0.93 yes yes no 0 1 0 0 predator
2015 June R69 2 humus Diptera Empididae Phyllodromia_melanocephala 1 0.13 yes yes no 0 1 0 0 predator
2015 June R69 2 humus Coleoptera Polyphaga . 1 2.29 . . . .
2015 June R69 2 humus Coleoptera Polyphaga . 1 4.02 . . . .
2015 June R69 2 humus_mineral Coleoptera Staphylinidae Tachinus_elongatus 1 2.87 yes yes no 0 0.5 0.5 0 combined
2015 June R69 2 humus_mineral Coleoptera Carabidae Notiophilus_biguttatus 1 2.15 yes yes no 0 1 0 0 predator
2015 June R69 2 humus_mineral Araneae Linyphiidae Gen_sp 1 0.15 yes yes no 0 1 0 0 predator
2015 June R69 3 humus Diptera Lauxaniidae Homoneura_sp 1 0.93 yes yes no 0 0 1 0 detritivore
2015 June R69 3 humus Diptera Lauxaniidae Homoneura_sp 1 1.18 yes yes no 0 0 1 0 detritivore
2015 June R69 3 humus_mineral Araneae Linyphiidae Gen_sp 1 0.29 yes yes no 0 1 0 0 predator
2015 June R69 3 humus_mineral Coleoptera Staphylinidae Oxypoda_annularis 1 0.09 yes yes no 0 1 0 0 predator
2015 June R69 3 humus_mineral Araneae Theridiidae Robertus_sp 1 0.00 yes yes no 0 1 0 0 predator
2015 June R69 3 mineral Coleoptera Elateridae Liotrichus_affinis 1 10.68 yes yes no 0 0.5 0.5 0 combined
2015 June R69 4 humus Coleoptera Elateridae Liotrichus_affinis 1 7.25 yes yes no 0 0.5 0.5 0 combined
2015 June R69 4 humus_mineral Araneae Linyphiidae Gen_sp 1 0.11 yes yes no 0 1 0 0 predator
2015 June R69 4 humus_mineral Coleoptera Staphylinidae Atheta_aeneipennis 1 0.19 yes yes no 0 1 0 0 predator
2015 June R69 5 humus Coleoptera Elateridae Eanus_costalis 2 14.62 yes yes no 0 0.5 0.5 0 combined
2015 June R69 5 humus Coleoptera Elateridae Liotrichus_affinis 3 34.10 yes yes no 0 0.5 0.5 0 combined
2015 June R69 5 humus_mineral Coleoptera Staphylinidae Liogluta_micans 1 0.31 yes yes no 0 1 0 0 predator
2015 June R69 5 humus_mineral Coleoptera Staphylinidae Othius_lapidicola 1 0.83 yes yes no 0 1 0 0 predator
2015 June R69 5 humus_mineral Araneae Theridiidae Robertus_sp 1 0.28 yes yes no 0 1 0 0 predator
2015 June R69 5 mineral Coleoptera Elateridae Eanus_costalis 1 3.53 yes yes no 0 0.5 0.5 0 combined
2015 June R69 6 humus Diptera Bibionidae Bibio_pomonae 1 28.50 yes yes no 0 0 1 0 detritivore
2015 June R69 6 humus Diptera Bibionidae Bibio_pomonae 1 36.88 yes yes no 0 0 1 0 detritivore
2015 June R69 6 humus Coleoptera Elateridae Eanus_costalis 1 0.33 yes yes no 0 0.5 0.5 0 combined
2015 June R69 6 humus Coleoptera Elateridae Liotrichus_affinis 1 0.28 yes yes no 0 0.5 0.5 0 combined
2015 June R69 6 humus_mineral Coleoptera Staphylinidae Tachinus_elongatus 1 1.11 yes yes no 0 0.5 0.5 0 combined
2015 June R69 6 humus_mineral Coleoptera Staphylinidae Oxypoda_annularis 1 0.08 yes yes no 0 1 0 0 predator
2015 June R69 6 mineral Diptera Bibionidae Bibio_pomonae 1 34.41 yes yes no 0 0 1 0 detritivore
2015 June R69 6 mineral Coleoptera Curculionidae Otiorhynchus_nodosus 1 13.26 yes yes yes 1 0 0 0 herbivore
2015 June R69 7 humus Diptera Cecidomyidae Aprionus_dentifer 1 0.37 yes yes no 0 0 1 0 detritivore

2015 June R69 7 humus Diptera Cecidomyidae Aprionus_dentifer 1 0.37 yes yes no 0 0 1 0 detritivore
2015 June R69 7 humus Diptera Cecidomyidae Aprionus_dentifer 1 0.39 yes yes no 0 0 1 0 detritivore
2015 June R69 7 humus Diptera Cecidomyidae Aprionus_dentifer 1 0.39 yes yes no 0 0 1 0 detritivore
2015 June R69 7 humus Coleoptera Elateridae Eanus_costalis 2 13.62 yes yes no 0 0.5 0.5 0 combined
2015 June R69 7 humus Coleoptera Elateridae Liotrichus_affinis 2 4.70 yes yes no 0 0.5 0.5 0 combined
2015 June R69 7 humus_mineral Coleoptera Staphylinidae Oxypoda_annularis 1 0.09 yes yes no 0 1 0 0 predator
2015 June R69 7 humus_mineral Coleoptera Staphylinidae Quedius_sp 1 0.72 yes yes no 0 1 0 0 predator
2015 June R69 7 humus_mineral Coleoptera Staphylinidae Stenus_palustris 1 0.21 yes yes no 0 1 0 0 predator
2015 June R69 7 humus_mineral Araneae Theridiidae Robertus_scoticus 1 0.33 yes yes no 0 1 0 0 predator
2015 June R69 8 humus Annelida Lumbricidae Dendrobaena_octaedra 1 20.04 yes yes no 0 0 1 0 detritivore
2015 June R69 8 humus Coleoptera Elateridae Eanus_costalis 3 15.25 yes yes no 0 0.5 0.5 0 combined
2015 June R69 8 humus_mineral Araneae Linyphiidae Tapinocyba_insecta 2 0.31 yes yes no 0 1 0 0 predator
2015 June R69 8 humus_mineral Coleoptera Staphylinidae Ischnoglossa_sp 1 0.12 yes yes no 0 1 0 0 predator
2015 June R69 8 humus_mineral Coleoptera Staphylinidae Oxypoda_annularis 1 0.07 yes yes no 0 1 0 0 predator
2015 June R69 8 humus_mineral Coleoptera Staphylinidae Oxypoda_annularis 1 0.08 yes yes no 0 1 0 0 predator
2015 June R69 8 humus_mineral Coleoptera Staphylinidae Oxypoda_annularis 1 0.09 yes yes no 0 1 0 0 predator
2015 June R69 8 humus_mineral Coleoptera Staphylinidae Oxypoda_annularis 1 0.11 yes yes no 0 1 0 0 predator
2015 June R69 8 humus_mineral Coleoptera Staphylinidae Oxypoda_annularis 1 0.12 yes yes no 0 1 0 0 predator
2015 June R69 9 humus Coleoptera Elateridae Eanus_costalis 2 3.47 yes yes no 0 0.5 0.5 0 combined
2015 June R69 9 humus_mineral Coleoptera Staphylinidae Oxypoda_annularis 1 0.08 yes yes no 0 1 0 0 predator
2015 June R69 9 humus_mineral Coleoptera Staphylinidae Quedius_sp 1 0.86 yes yes no 0 1 0 0 predator
2015 June R69 9 humus_mineral Coleoptera Staphylinidae Stenus_palustris 1 0.28 yes yes no 0 1 0 0 predator
2015 June R69 10 humus Coleoptera Elateridae Eanus_costalis 4 16.96 yes yes no 0 0.5 0.5 0 combined
2015 June R69 10 humus Coleoptera Elateridae Liotrichus_affinis 1 14.23 yes yes no 0 0.5 0.5 0 combined
2015 June R69 10 humus_mineral Araneae Linyphiidae Gen_sp 3 1.29 yes yes no 0 1 0 0 predator
2015 June R69 10 humus_mineral Araneae Linyphiidae Semljicola_latus 1 0.00 yes yes no 0 1 0 0 predator
2015 June R69 10 humus_mineral Araneae Theridiidae Robertus_scoticus 2 0.00 yes yes no 0 1 0 0 predator
2015 August R60 1 humus Coleoptera Cryptophagidae Atomaria_nitidula 1 0.10 yes yes no 0 0 0.5 0.5 combined
2015 August R60 1 humus Annelida Lumbricidae Lumbricus_rubellus 1 144.48 yes yes no 0 0 1 0 detritivore
2015 August R60 1 humus Diptera Rhagionidae Rhagio_lineola 1 0.08 yes yes no 0 1 0 0 predator
2015 August R60 1 humus Coleoptera Curculionidae Phyllobius_arborator 1 3.37 yes yes yes 1 0 0 0 herbivore
2015 August R60 1 humus Lepidoptera Hepialidae Korscheltellus_fusconebulosa 1 5.48 Yes Yes yes 1 0 0 0 herbivore
2015 August R60 1 humus Hemiptera Lygaeidae Drymus_brunneus 1 1.01 yes yes no 1 0 0 0 herbivore
2015 August R60 1 humus_mineral Coleoptera Staphylinidae Amischa_bifoveolata 1 0.12 yes yes no 0 1 0 0 predator
2015 August R60 1 humus_mineral Araneae Theridiidae Robertus_sp 1 0.08 yes yes no 0 1 0 0 predator
2015 August R60 2 humus Lepidoptera Hepialidae Phymatopus_hecta 1 17.70 Yes Yes yes 1 0 0 0 herbivore
2015 August R60 2 humus_mineral Hymenoptera Formicidae Myrmica_ruginodis 1 0.43 Yes Yes no 0 1 0 0 predator
2015 August R60 3 humus Annelida Lumbricidae Dendrodrilus_rubidus_tenuis 1 18.84 yes yes no 0 0 1 0 detritivore
2015 August R60 3 humus Diptera Rhagionidae Rhagio_lineola 1 0.76 yes yes no 0 1 0 0 predator
2015 August R60 3 humus_mineral Hymenoptera Formicidae Myrmica_ruginodis 1 0.76 Yes Yes no 0 1 0 0 predator
2015 August R60 3 humus_mineral Araneae Hahniidae Hahnia_pusilla 1 0.00 yes yes no 0 1 0 0 predator
2015 August R60 3 humus_mineral Araneae Hahniidae Hahnia_sp 1 0.00 yes yes no 0 1 0 0 predator
2015 August R60 3 humus_mineral Araneae Linyphiidae Centromerus_arcanus 1 0.00 yes yes no 0 1 0 0 predator
2015 August R60 3 humus_mineral Araneae Linyphiidae Gen_sp 2 0.00 yes yes no 0 1 0 0 predator
2015 August R60 3 humus_mineral Araneae Linyphiidae Macrargus_rufus 1 0.00 yes yes no 0 1 0 0 predator
2015 August R60 3 humus_mineral Araneae Linyphiidae Tapinocyba_pallens 1 4.78 yes yes no 0 1 0 0 predator
2015 August R60 3 humus_mineral Myriapoda Lithobiidae Lithobius_sp 1 0.69 yes yes no 0 1 0 0 predator
2015 August R60 3 humus_mineral Araneae Lycosidae Pirata_Piratula_sp 1 0.00 yes yes no 0 1 0 0 predator
2015 August R60 3 humus_mineral Coleoptera Staphylinidae Othius_subuliformis 1 0.52 yes yes no 0 1 0 0 predator
2015 August R60 3 humus_mineral Coleoptera Staphylinidae Quedius_curtipennis 1 4.80 yes yes no 0 1 0 0 predator
2015 August R60 4 humus_mineral Araneae Lycosidae Pirata_Piratula_sp 1 1.47 yes yes no 0 1 0 0 predator
2015 August R60 4 humus_mineral Coleoptera Staphylinidae Amischa_decipiens 1 0.06 yes yes no 0 1 0 0 predator
2015 August R60 4 mineral Coleoptera Elateridae Athous_subfuscus 1 2.82 yes yes yes 0 0.8 0.1 0.1 combined

2015 August R60 5 humus Coleoptera Staphylinidae . 1 0.25 yes yes no 0 1 0 0 predator
2015 August R60 5 humus Diptera Chironomidae . 1 0.04 yes yes yes 0.25 0 0.75 0 combined
2015 August R60 5 humus Coleoptera Curculionidae Otiorhynchus_carinatopunctatus 1 4.42 yes yes yes 1 0 0 0 herbivore
2015 August R60 5 humus_mineral Araneae Linyphiidae Gen_sp 2 1.28 yes yes no 0 1 0 0 predator
2015 August R60 5 humus_mineral Myriapoda Lithobiidae Lithobius_sp 1 0.29 yes yes no 0 1 0 0 predator
2015 August R60 6 humus Myriapoda Polydesmidae Polydesmus_denticulatus 1 0.37 yes yes no 0 0 1 0 detritivore
2015 August R60 6 humus Coleoptera Staphylinidae Xantholinus_tricolor 1 0.84 yes yes no 0 1 0 0 predator
2015 August R60 6 humus_mineral Hymenoptera Formicidae Myrmica_rubra 1 0.65 Yes Yes no 0 1 0 0 predator
2015 August R60 6 humus_mineral Araneae Linyphiidae Tenuiphantes_cristatus 1 0.00 yes yes no 0 1 0 0 predator
2015 August R60 6 humus_mineral Araneae Lycosidae Pirata_Piratula_sp 2 0.94 yes yes no 0 1 0 0 predator
2015 August R60 7 humus Coleoptera Elateridae Athous_subfuscus 1 3.59 yes yes yes 0 0.8 0.1 0.1 combined
2015 August R60 7 humus Coleoptera Staphylinidae Othius_subuliformis 1 0.45 yes yes no 0 1 0 0 predator
2015 August R60 7 humus Coleoptera Elateridae Paraphotistus_impressus 1 0.82 yes yes yes 0.6 0.13 0.13 0.13 combined
2015 August R60 7 humus_mineral Hymenoptera Formicidae Myrmica_ruginodis 1 0.37 Yes Yes no 0 1 0 0 predator
2015 August R60 7 humus_mineral Araneae Linyphiidae Maro_minutus 2 0.00 yes yes no 0 1 0 0 predator
2015 August R60 7 humus_mineral Araneae Linyphiidae Tapinocyba_pallens 1 0.24 yes yes no 0 1 0 0 predator
2015 August R60 7 humus_mineral Myriapoda Lithobiidae Lithobius_curtipes 1 0.73 yes yes no 0 1 0 0 predator
2015 August R60 7 humus_mineral Myriapoda Lithobiidae Lithobius_curtipes 1 1.10 yes yes no 0 1 0 0 predator
2015 August R60 7 humus_mineral Myriapoda Lithobiidae Lithobius_sp 1 0.05 yes yes no 0 1 0 0 predator
2015 August R60 7 humus_mineral Coleoptera Staphylinidae Amischa_bifoveolata 1 0.07 yes yes no 0 1 0 0 predator
2015 August R60 7 humus_mineral Coleoptera Staphylinidae Oxypoda_annularis 1 0.07 yes yes no 0 1 0 0 predator
2015 August R60 7 humus_mineral Coleoptera Staphylinidae Oxypoda_annularis 1 0.07 yes yes no 0 1 0 0 predator
2015 August R60 7 humus_mineral Coleoptera Staphylinidae Philonthus_subvirescens 1 3.20 yes yes no 0 1 0 0 predator
2015 August R60 8 humus Annelida Lumbricidae Dendrobaena_octaedra 1 0.04 yes yes no 0 0 1 0 detritivore
2015 August R60 8 humus Coleoptera Chrysomelidae Lochmaea_caprea 1 2.69 no no no 1 0 0 0 herbivore
2015 August R60 8 humus_mineral Araneae Linyphiidae Tapinocyba_pallens 2 0.27 yes yes no 0 1 0 0 predator
2015 August R60 8 humus_mineral Coleoptera Staphylinidae Geostiba_circellaris 1 0.15 yes yes no 0 1 0 0 predator
2015 August R60 8 humus_mineral Coleoptera Staphylinidae Othius_subuliformis 1 0.65 yes yes no 0 1 0 0 predator
2015 August R60 9 humus Coleoptera Elateridae Athous_subfuscus 2 7.36 yes yes yes 0 0.8 0.1 0.1 combined
2015 August R60 9 humus Coleoptera Elateridae Dalopius_marginatus 1 3.09 yes yes yes 0 0.8 0.1 0.1 combined
2015 August R60 9 humus Coleoptera Staphylinidae Brachygluta_fossulata 1 0.11 yes yes no 0 1 0 0 predator
2015 August R60 9 humus Coleoptera Staphylinidae Othius_subuliformis 1 0.29 yes yes no 0 1 0 0 predator
2015 August R60 9 humus Hemiptera Ortheziidae Newsteadia_floccosa 1 0.14 yes yes yes 1 0 0 0 herbivore
2015 August R60 9 humus_mineral Hymenoptera Formicidae Myrmica_rubra 12 7.75 Yes Yes no 0 1 0 0 predator
2015 August R60 9 humus_mineral Hymenoptera Formicidae Myrmica_ruginodis 1 0.42 Yes Yes no 0 1 0 0 predator
2015 August R60 9 humus_mineral Araneae Linyphiidae Gen_sp 1 0.00 yes yes no 0 1 0 0 predator
2015 August R60 9 humus_mineral Araneae Linyphiidae Tapinocyba_pallens 2 2.03 yes yes no 0 1 0 0 predator
2015 August R60 9 humus_mineral Araneae Linyphiidae Tapinopa_longidens 1 0.00 yes yes no 0 1 0 0 predator
2015 August R60 9 humus_mineral Myriapoda Lithobiidae Lithobius_curtipes 1 1.51 yes yes no 0 1 0 0 predator
2015 August R60 9 humus_mineral Myriapoda Lithobiidae Lithobius_curtipes 1 1.87 yes yes no 0 1 0 0 predator
2015 August R60 9 humus_mineral Araneae Lycosidae Pirata_Piratula_sp 1 0.00 yes yes no 0 1 0 0 predator
2015 August R60 9 humus_mineral Coleoptera Staphylinidae Geostiba_circellaris 1 0.06 yes yes no 0 1 0 0 predator
2015 August R60 10 humus Coleoptera Elateridae Athous_subfuscus 1 0.12 yes yes yes 0 0.8 0.1 0.1 combined
2015 August R60 10 humus Araneae Linyphiidae Tapinocyba_pallens 1 0.17 yes yes no 0 1 0 0 predator
2015 August R60 10 humus Lepidoptera Hepialidae Phymatopus_hecta 1 23.76 Yes Yes yes 1 0 0 0 herbivore
2015 August R60 10 humus Hymenoptera Pamphilidae . 1 61.55 Yes no no 1 0 0 0 herbivore
2015 August R60 10 humus_mineral Araneae Linyphiidae Porrhomma_pallidum 1 0.00 yes yes no 0 1 0 0 predator
2015 August R60 10 humus_mineral Araneae Linyphiidae Tapinocyba_pallens 1 0.00 yes yes no 0 1 0 0 predator
2015 August R60 10 humus_mineral Araneae Lycosidae Pirata_Piratula_sp 1 1.74 yes yes no 0 1 0 0 predator
2015 August R60 10 humus_mineral Coleoptera Staphylinidae Philonthus_subvirescens 1 1.81 yes yes no 0 1 0 0 predator
2015 August R60 10 humus_mineral Araneae Theridiidae Robertus_sp 1 0.00 yes yes no 0 1 0 0 predator
2015 August R60 10 mineral Diptera Asilidae Neoitamus_socius 1 7.84 yes yes no 0 1 0 0 predator
2015 August R61 1 humus_mineral Hymenoptera Formicidae Tetramorium_caespitum 3 0.48 Yes Yes no 0.6 0.2 0.2 0 combined

2015 August R61 2 humus Coleoptera Elateridae Athous_subfuscus 1 5.22 yes yes yes 0 0.8 0.1 0.1 combined
2015 August R61 2 humus Coleoptera Staphylinidae Othius_subuliformis 1 0.08 yes yes no 0 1 0 0 predator
2015 August R61 2 humus_mineral Araneae Hahniidae Hahnia_sp 1 0.00 yes yes no 0 1 0 0 predator
2015 August R61 2 humus_mineral Araneae Linyphiidae Macrargus_multesimus 1 0.71 yes yes no 0 1 0 0 predator
2015 August R61 3 humus Diptera Rhagionidae Rhagio_lineola 1 1.08 yes yes no 0 1 0 0 predator
2015 August R61 3 humus Coleoptera Staphylinidae Xantholinus_tricolor 1 1.86 yes yes no 0 1 0 0 predator
2015 August R61 3 humus Coleoptera Curculionidae Strophosoma_capitatum 1 2.03 yes yes yes 1 0 0 0 herbivore
2015 August R61 3 humus_mineral Araneae Hahniidae Hahnia_sp 1 0.46 yes yes no 0 1 0 0 predator
2015 August R61 3 humus_mineral Araneae Linyphiidae Maso_sundevalli 1 0.00 yes yes no 0 1 0 0 predator
2015 August R61 3 mineral Coleoptera Elateridae Athous_subfuscus 2 9.37 yes yes yes 0 0.8 0.1 0.1 combined
2015 August R61 3 mineral Coleoptera Staphylinidae Xantholinus_tricolor 1 1.52 yes yes no 0 1 0 0 predator
2015 August R61 4 humus Coleoptera Staphylinidae Othius_subuliformis 1 0.37 yes yes no 0 1 0 0 predator
2015 August R61 4 humus_mineral Araneae Theridiidae Euryopis_sp 1 0.50 yes yes no 0 1 0 0 predator
2015 August R61 5 humus Coleoptera Elateridae Athous_subfuscus 1 4.52 yes yes yes 0 0.8 0.1 0.1 combined
2015 August R61 5 humus Coleoptera Staphylinidae . 1 0.18 yes yes no 0 1 0 0 predator
2015 August R61 5 humus Coleoptera Staphylinidae . 1 0.84 yes yes no 0 1 0 0 predator
2015 August R61 5 humus Coleoptera Staphylinidae Othius_subuliformis 1 0.28 yes yes no 0 1 0 0 predator
2015 August R61 5 humus Coleoptera Staphylinidae Othius_subuliformis 1 0.29 yes yes no 0 1 0 0 predator
2015 August R61 5 humus Hemiptera Lygaeidae Drymus_brunneus 1 0.20 yes yes no 1 0 0 0 herbivore
2015 August R61 5 humus_mineral Araneae Linyphiidae Tapinocyba_pallens 1 0.00 yes yes no 0 1 0 0 predator
2015 August R61 5 humus_mineral Araneae Linyphiidae Walckenaeria_dysderoides 1 0.00 yes yes no 0 1 0 0 predator
2015 August R61 5 humus_mineral Araneae Lycosidae Gen_sp 1 0.69 yes yes no 0 1 0 0 predator
2015 August R61 5 humus_mineral Coleoptera Staphylinidae Othius_subuliformis 1 0.50 yes yes no 0 1 0 0 predator
2015 August R61 6 humus Coleoptera Elateridae Athous_subfuscus 1 6.63 yes yes yes 0 0.8 0.1 0.1 combined
2015 August R61 6 humus Diptera Rhagionidae Rhagio_lineola 1 2.18 yes yes no 0 1 0 0 predator
2015 August R61 6 humus Coleoptera Chrysomelidae Lochmaea_caprea 1 3.91 no no no 1 0 0 0 herbivore
2015 August R61 6 humus Hemiptera Lygaeidae Stygnocoris_sabulosus 1 0.43 yes no no 1 0 0 0 herbivore
2015 August R61 6 humus_mineral Coleoptera Staphylinidae Tachyporus_chrysomelinus 1 0.31 yes yes no 0 0.5 0.5 0 combined
2015 August R61 6 humus_mineral Araneae Gnaphosidae Haplodrassus_sp 1 0.87 yes yes no 0 1 0 0 predator
2015 August R61 6 humus_mineral Coleoptera Staphylinidae Othius_subuliformis 1 0.34 yes yes no 0 1 0 0 predator
2015 August R61 7 humus Diptera Therevidae Thereva_handlirschi 1 23.34 yes yes no 0 1 0 0 predator
2015 August R61 7 humus_mineral Araneae Linyphiidae Gen_sp 1 0.64 yes yes no 0 1 0 0 predator
2015 August R61 7 humus_mineral Myriapoda Lithobiidae Lithobius_sp 1 0.20 yes yes no 0 1 0 0 predator
2015 August R61 7 humus_mineral Araneae Lycosidae Gen_sp 1 0.00 yes yes no 0 1 0 0 predator
2015 August R61 8 humus Coleoptera Elateridae Dalopius_marginatus 1 2.79 yes yes yes 0 0.8 0.1 0.1 combined
2015 August R61 8 humus_mineral Hymenoptera Formicidae Myrmica_ruginodis 1 0.39 Yes Yes no 0 1 0 0 predator
2015 August R61 8 humus_mineral Araneae Linyphiidae Tapinocyba_pallens 2 0.07 yes yes no 0 1 0 0 predator
2015 August R61 9 humus Annelida Lumbricidae Dendrobaena_octaedra 1 35.76 yes yes no 0 0 1 0 detritivore
2015 August R61 9 humus Diptera Rhagionidae Rhagio_lineola 1 1.14 yes yes no 0 1 0 0 predator
2015 August R61 9 humus Diptera Rhagionidae Rhagio_lineola 1 3.47 yes yes no 0 1 0 0 predator
2015 August R61 9 humus Coleoptera Staphylinidae Othius_subuliformis 1 0.29 yes yes no 0 1 0 0 predator
2015 August R61 9 humus Coleoptera Curculionidae Strophosoma_capitatum 1 1.76 yes yes yes 1 0 0 0 herbivore
2015 August R61 9 humus_mineral Coleoptera Carabidae Calathus_micropterus 1 6.67 yes yes no 0 1 0 0 predator
2015 August R61 9 humus_mineral Araneae Hahniidae Hahnia_pusilla 1 0.00 yes yes no 0 1 0 0 predator
2015 August R61 9 humus_mineral Araneae Hahniidae Hahnia_sp 1 0.00 yes yes no 0 1 0 0 predator
2015 August R61 9 humus_mineral Araneae Linyphiidae Gen_sp 1 0.54 yes yes no 0 1 0 0 predator
2015 August R61 9 humus_mineral Araneae Lycosidae Gen_sp 1 0.00 yes yes no 0 1 0 0 predator
2015 August R61 10 humus Coleoptera Elateridae Athous_subfuscus 1 2.94 yes yes yes 0 0.8 0.1 0.1 combined
2015 August R61 10 humus_mineral Araneae Hahniidae Hahnia_pusilla 1 0.00 yes yes no 0 1 0 0 predator
2015 August R61 10 humus_mineral Myriapoda Lithobiidae Lithobius_curtipes 1 1.58 yes yes no 0 1 0 0 predator
2015 August R61 10 humus_mineral Araneae Salticidae Euophrys_frontalis 1 1.75 yes yes no 0 1 0 0 predator
2015 August R61 10 humus_mineral Coleoptera Staphylinidae Othius_subuliformis 1 0.44 yes yes no 0 1 0 0 predator
2015 August R62 1 humus Diptera Heleomyzidae Neoleria_sp 1 0.23 yes yes no 0 0 1 0 detritivore

2015 August R62 1 humus Annelida Lumbricidae Dendrobaena_octaedra 2 40.20 yes yes no 0 0 1 0 detritivore
2015 August R62 1 humus Coleoptera Staphylinidae . 1 1.65 yes yes no 0 1 0 0 predator
2015 August R62 1 humus Hemiptera Lygaeidae Drymus_brunneus 1 1.08 yes yes no 1 0 0 0 herbivore
2015 August R62 1 humus_mineral Coleoptera Staphylinidae Tachinus_rufipes 1 1.92 yes yes no 0 0.5 0.5 0 combined
2015 August R62 1 humus_mineral Hymenoptera Formicidae Myrmica_rubra 1 0.68 Yes Yes no 0 1 0 0 predator
2015 August R62 1 humus_mineral Hymenoptera Formicidae Myrmica_ruginodis 2 1.42 Yes Yes no 0 1 0 0 predator
2015 August R62 1 humus_mineral Araneae Linyphiidae Anguliphantes_angulipalpis 1 0.00 yes yes no 0 1 0 0 predator
2015 August R62 1 humus_mineral Araneae Linyphiidae Gen_sp 1 0.00 yes yes no 0 1 0 0 predator
2015 August R62 1 humus_mineral Araneae Linyphiidae Minyriolus_pusillus 1 0.00 yes yes no 0 1 0 0 predator
2015 August R62 1 humus_mineral Araneae Linyphiidae Tapinocyba_insecta 4 0.99 yes yes no 0 1 0 0 predator
2015 August R62 1 humus_mineral Myriapoda Lithobiidae Lithobius_curtipes 1 2.43 yes yes no 0 1 0 0 predator
2015 August R62 1 humus_mineral Myriapoda Lithobiidae Lithobius_curtipes 1 2.65 yes yes no 0 1 0 0 predator
2015 August R62 1 humus_mineral Coleoptera Staphylinidae Drusilla_canaliculata 1 0.82 yes yes no 0 1 0 0 predator
2015 August R62 1 humus_mineral Araneae Theridiidae Gen_sp 1 0.00 yes yes no 0 1 0 0 predator
2015 August R62 1 mineral Coleoptera Elateridae Dalopius_marginatus 1 5.78 yes yes yes 0 0.8 0.1 0.1 combined
2015 August R62 1 mineral Coleoptera Elateridae Selatosomus_aeneus 1 23.86 yes yes yes 0.6 0.13 0.13 0.13 combined
2015 August R62 2 humus Annelida Lumbricidae Dendrobaena_octaedra 1 5.38 yes yes no 0 0 1 0 detritivore
2015 August R62 2 humus Annelida Lumbricidae Eiseniella_tetraedra 6 3.09 yes yes no 0 0 1 0 detritivore
2015 August R62 2 humus_mineral Myriapoda Julidae Ommatoiulus_sabulosus 1 24.74 yes yes no 0 0 1 0 detritivore
2015 August R62 2 humus_mineral Myriapoda Julidae Ommatoiulus_sabulosus 1 27.22 yes yes no 0 0 1 0 detritivore
2015 August R62 2 humus_mineral Myriapoda Lithobiidae Lithobius_curtipes 1 0.51 yes yes no 0 1 0 0 predator
2015 August R62 2 mineral Coleoptera Elateridae Paraphotistus_impressus 1 4.64 yes yes yes 0.6 0.13 0.13 0.13 combined
2015 August R62 2 mineral Coleoptera Elateridae Selatosomus_aeneus 1 0.95 yes yes yes 0.6 0.13 0.13 0.13 combined
2015 August R62 3 humus Annelida Lumbricidae Dendrobaena_octaedra 1 2.74 yes yes no 0 0 1 0 detritivore
2015 August R62 3 humus Diptera . . 1 0.08 . . . .
2015 August R62 3 humus_mineral Hymenoptera Formicidae Myrmica_ruginodis 51 22.36 Yes Yes no 0 1 0 0 predator
2015 August R62 3 humus_mineral Araneae Linyphiidae Gen_sp 1 0.00 yes yes no 0 1 0 0 predator
2015 August R62 3 humus_mineral Araneae Linyphiidae Tapinocyba_pallens 1 0.00 yes yes no 0 1 0 0 predator
2015 August R62 3 humus_mineral Myriapoda Lithobiidae Lithobius_curtipes 1 0.06 yes yes no 0 1 0 0 predator
2015 August R62 3 humus_mineral Myriapoda Lithobiidae Lithobius_curtipes 1 0.21 yes yes no 0 1 0 0 predator
2015 August R62 3 humus_mineral Myriapoda Lithobiidae Lithobius_curtipes 1 0.90 yes yes no 0 1 0 0 predator
2015 August R62 3 humus_mineral Myriapoda Lithobiidae Lithobius_curtipes 1 1.00 yes yes no 0 1 0 0 predator
2015 August R62 3 humus_mineral Myriapoda Lithobiidae Lithobius_curtipes 1 1.02 yes yes no 0 1 0 0 predator
2015 August R62 3 humus_mineral Myriapoda Lithobiidae Lithobius_curtipes 1 1.92 yes yes no 0 1 0 0 predator
2015 August R62 3 humus_mineral Araneae Lycosidae Gen_sp 1 0.50 yes yes no 0 1 0 0 predator
2015 August R62 3 humus_mineral Coleoptera Staphylinidae Acrotona_silvicola 1 0.12 yes yes no 0 1 0 0 predator
2015 August R62 3 humus_mineral Coleoptera Staphylinidae Drusilla_canaliculata 1 0.84 yes yes no 0 1 0 0 predator
2015 August R62 3 humus_mineral Coleoptera Staphylinidae Quedius_curtipennis 1 4.68 yes yes no 0 1 0 0 predator
2015 August R62 3 mineral Coleoptera Staphylinidae Xantholinus_distans 1 0.55 yes yes no 0 1 0 0 predator
2015 August R62 4 humus Coleoptera Elateridae Athous_subfuscus 1 5.34 yes yes yes 0 0.8 0.1 0.1 combined
2015 August R62 4 humus Coleoptera Elateridae Dalopius_marginatus 1 4.30 yes yes yes 0 0.8 0.1 0.1 combined
2015 August R62 4 humus Coleoptera Carabidae Calathus_micropterus 1 0.88 yes yes no 0 1 0 0 predator
2015 August R62 4 humus Hemiptera Lygaeidae Drymus_brunneus 1 0.69 yes yes no 1 0 0 0 herbivore
2015 August R62 4 humus_mineral Araneae Dictynidae Lathys_heterophthalma 1 0.00 yes yes no 0 1 0 0 predator
2015 August R62 4 humus_mineral Hymenoptera Formicidae Myrmica_ruginodis 12 5.38 Yes Yes no 0 1 0 0 predator
2015 August R62 4 humus_mineral Araneae Hahniidae Hahnia_pusilla 2 0.00 yes yes no 0 1 0 0 predator
2015 August R62 4 humus_mineral Araneae Linyphiidae Dicymbium_tibiale 1 0.00 yes yes no 0 1 0 0 predator
2015 August R62 4 humus_mineral Araneae Linyphiidae Diplostyla_concolor 1 0.00 yes yes no 0 1 0 0 predator
2015 August R62 4 humus_mineral Araneae Linyphiidae Gen_sp 1 3.95 yes yes no 0 1 0 0 predator
2015 August R62 4 humus_mineral Araneae Linyphiidae Minyriolus_pusillus 1 0.00 yes yes no 0 1 0 0 predator
2015 August R62 4 humus_mineral Araneae Linyphiidae Tapinopa_longidens 1 0.00 yes yes no 0 1 0 0 predator
2015 August R62 4 humus_mineral Myriapoda Lithobiidae Lithobius_curtipes 1 0.54 yes yes no 0 1 0 0 predator
2015 August R62 4 humus_mineral Myriapoda Lithobiidae Lithobius_curtipes 1 0.55 yes yes no 0 1 0 0 predator

2015 August R62 4 humus_mineral Araneae Salticidae Euophrys_sp 1 0.00 yes yes no 0 1 0 0 predator
2015 August R62 4 humus_mineral Hymenoptera Formicidae Lasius_flavus 9 2.37 Yes Yes no 0.8 0.2 0 0 combined
2015 August R62 5 humus Annelida Lumbricidae Lumbricus_rubellus 1 95.28 yes yes no 0 0 1 0 detritivore
2015 August R62 5 humus Coleoptera Elateridae Athous_subfuscus 1 4.12 yes yes yes 0 0.8 0.1 0.1 combined
2015 August R62 5 humus Coleoptera Elateridae Dalopius_marginatus 1 4.25 yes yes yes 0 0.8 0.1 0.1 combined
2015 August R62 5 humus Diptera Rhagionidae Rhagio_lineola 1 3.76 yes yes no 0 1 0 0 predator
2015 August R62 5 humus_mineral Hymenoptera Formicidae Myrmica_ruginodis 1 0.62 Yes Yes no 0 1 0 0 predator
2015 August R62 5 humus_mineral Araneae Linyphiidae Dicymbium_tibiale 1 0.55 yes yes no 0 1 0 0 predator
2015 August R62 5 humus_mineral Coleoptera Staphylinidae Acrotona_silvicola 1 0.13 yes yes no 0 1 0 0 predator
2015 August R62 5 humus_mineral Coleoptera Staphylinidae Lathrobium_geminum 1 1.23 yes yes no 0 1 0 0 predator
2015 August R62 5 humus_mineral Coleoptera Staphylinidae Lathrobium_geminum 1 1.83 yes yes no 0 1 0 0 predator
2015 August R62 5 humus_mineral Coleoptera Staphylinidae Quedius_fulvicollis 1 1.06 yes yes no 0 1 0 0 predator
2015 August R62 5 humus_mineral Coleoptera Staphylinidae Stenus_clavicornis 1 0.66 yes yes no 0 1 0 0 predator
2015 August R62 5 humus_mineral Coleoptera Staphylinidae Xantholinus_laevigatus 1 1.19 yes yes no 0 1 0 0 predator
2015 August R62 5 humus_mineral Coleoptera Staphylinidae Xantholinus_tricolor 1 2.31 yes yes no 0 1 0 0 predator
2015 August R62 5 humus_mineral Coleoptera Staphylinidae Xantholinus_tricolor 1 2.62 yes yes no 0 1 0 0 predator
2015 August R62 6 humus Blattoptera Ectobiidae Ectobius_sylvestris 1 2.75 yes yes no 0 0 1 0 detritivore
2015 August R62 6 humus Annelida Lumbricidae Dendrodrilus_rubidus_tenuis 1 14.40 yes yes no 0 0 1 0 detritivore
2015 August R62 6 humus Coleoptera Elateridae Athous_subfuscus 1 3.02 yes yes yes 0 0.8 0.1 0.1 combined
2015 August R62 6 humus Diptera Rhagionidae Rhagio_lineola 1 2.02 yes yes no 0 1 0 0 predator
2015 August R62 6 humus Coleoptera Staphylinidae . 1 0.37 yes yes no 0 1 0 0 predator
2015 August R62 6 humus Coleoptera Scirtidae Cyphon_padi 1 0.17 no no no 0.5 0 0.5 0 combined
2015 August R62 6 humus Coleoptera Elateridae Paraphotistus_impressus 1 3.26 yes yes yes 0.6 0.13 0.13 0.13 combined
2015 August R62 6 humus Hemiptera Lygaeidae Drymus_brunneus 1 0.92 yes yes no 1 0 0 0 herbivore
2015 August R62 6 humus Hemiptera Lygaeidae Drymus_brunneus 1 1.02 yes yes no 1 0 0 0 herbivore
2015 August R62 6 humus_mineral Araneae Hahniidae Hahnia_sp 1 0.25 yes yes no 0 1 0 0 predator
2015 August R62 6 humus_mineral Araneae Linyphiidae Erigonella_hiemalis 1 0.00 yes yes no 0 1 0 0 predator
2015 August R62 6 humus_mineral Coleoptera Staphylinidae Oxypoda_annularis 1 0.07 yes yes no 0 1 0 0 predator
2015 August R62 6 humus_mineral Coleoptera Staphylinidae Stenus_palustris 1 0.30 yes yes no 0 1 0 0 predator
2015 August R62 6 humus_mineral Araneae Theridiidae Gen_sp 1 0.00 yes yes no 0 1 0 0 predator
2015 August R62 7 humus Diptera Lonchaeidae Lonchaea_ragnari 1 0.74 yes yes no 0 0 1 0 detritivore
2015 August R62 7 humus Annelida Lumbricidae Dendrobaena_octaedra 3 67.32 yes yes no 0 0 1 0 detritivore
2015 August R62 7 humus Annelida Lumbricidae Dendrodrilus_rubidus_tenuis 1 0.57 yes yes no 0 0 1 0 detritivore
2015 August R62 7 humus Annelida Lumbricidae Dendrodrilus_rubidus_tenuis 2 14.02 yes yes no 0 0 1 0 detritivore
2015 August R62 7 humus Coleoptera Elateridae Athous_subfuscus 1 0.87 yes yes yes 0 0.8 0.1 0.1 combined
2015 August R62 7 humus Coleoptera Staphylinidae Xantholinus_distans 1 0.19 yes yes no 0 1 0 0 predator
2015 August R62 7 humus Coleoptera Curculionidae Strophosoma_capitatum 1 2.01 yes yes yes 1 0 0 0 herbivore
2015 August R62 7 humus_mineral Hymenoptera Formicidae Myrmica_ruginodis 13 5.96 Yes Yes no 0 1 0 0 predator
2015 August R62 7 humus_mineral Araneae Linyphiidae Walckenaeria_dysderoides 1 0.20 yes yes no 0 1 0 0 predator
2015 August R62 7 humus_mineral Myriapoda Lithobiidae Lithobius_curtipes 1 0.90 yes yes no 0 1 0 0 predator
2015 August R62 8 humus Annelida Lumbricidae Dendrodrilus_rubidus_tenuis 1 18.72 yes yes no 0 0 1 0 detritivore
2015 August R62 8 humus Coleoptera Elateridae Athous_subfuscus 1 5.09 yes yes yes 0 0.8 0.1 0.1 combined
2015 August R62 8 humus Hemiptera Lygaeidae Drymus_brunneus 1 0.96 yes yes no 1 0 0 0 herbivore
2015 August R62 8 humus_mineral Araneae Linyphiidae Gen_sp 1 0.00 yes yes no 0 1 0 0 predator
2015 August R62 8 humus_mineral Araneae Linyphiidae Tapinocyba_pallens 1 0.45 yes yes no 0 1 0 0 predator
2015 August R62 8 mineral Coleoptera Elateridae Dalopius_marginatus 1 1.43 yes yes yes 0 0.8 0.1 0.1 combined
2015 August R62 9 humus Coleoptera Elateridae Athous_subfuscus 1 5.52 yes yes yes 0 0.8 0.1 0.1 combined
2015 August R62 9 humus Coleoptera Elateridae Dalopius_marginatus 3 8.07 yes yes yes 0 0.8 0.1 0.1 combined
2015 August R62 9 humus Hemiptera Lygaeidae Drymus_brunneus 1 0.93 yes yes no 1 0 0 0 herbivore
2015 August R62 9 humus Hemiptera Lygaeidae Drymus_brunneus 1 1.47 yes yes no 1 0 0 0 herbivore
2015 August R62 9 humus_mineral Hymenoptera Formicidae Myrmica_rubra 1 0.68 Yes Yes no 0 1 0 0 predator
2015 August R62 9 humus_mineral Hymenoptera Formicidae Myrmica_ruginodis 3 1.20 Yes Yes no 0 1 0 0 predator
2015 August R62 9 humus_mineral Araneae Linyphiidae Tapinocyba_pallens 1 1.02 yes yes no 0 1 0 0 predator

2015 August R62 9 humus_mineral Araneae Linyphiidae Walckenaeria_dysderoides 1 0.00 yes yes no 0 1 0 0 predator
2015 August R62 9 humus_mineral Myriapoda Lithobiidae Lithobius_curtipes 1 0.37 yes yes no 0 1 0 0 predator
2015 August R62 9 humus_mineral Myriapoda Lithobiidae Lithobius_curtipes 1 1.86 yes yes no 0 1 0 0 predator
2015 August R62 9 humus_mineral Coleoptera Staphylinidae Othius_punctulatus 1 3.67 yes yes no 0 1 0 0 predator
2015 August R62 9 mineral Coleoptera Elateridae Athous_subfuscus 1 3.86 yes yes yes 0 0.8 0.1 0.1 combined
2015 August R62 10 humus Annelida Lumbricidae Eiseniella_tetraedra 1 8.69 yes yes no 0 0 1 0 detritivore
2015 August R62 10 humus Annelida Lumbricidae Lumbricus_rubellus 1 163.20 yes yes no 0 0 1 0 detritivore
2015 August R62 10 humus Coleoptera Elateridae Selatosomus_aeneus 1 6.92 yes yes yes 0.6 0.13 0.13 0.13 combined
2015 August R62 10 humus_mineral Hymenoptera Formicidae Myrmica_ruginodis 1 0.56 Yes Yes no 0 1 0 0 predator
2015 August R62 10 humus_mineral Araneae Linyphiidae Gen_sp 2 0.00 yes yes no 0 1 0 0 predator
2015 August R62 10 humus_mineral Araneae Linyphiidae Maso_sundevalli 1 0.47 yes yes no 0 1 0 0 predator
2015 August R62 10 humus_mineral Myriapoda Lithobiidae Lithobius_curtipes 1 1.01 yes yes no 0 1 0 0 predator
2015 August R62 10 humus_mineral Myriapoda Lithobiidae Lithobius_sp 1 0.10 yes yes no 0 1 0 0 predator
2015 August R62 10 humus_mineral Myriapoda Lithobiidae Lithobius_sp 1 0.21 yes yes no 0 1 0 0 predator
2015 August R62 10 mineral Coleoptera Elateridae Selatosomus_aeneus 2 22.12 yes yes yes 0.6 0.13 0.13 0.13 combined
2015 August R63 1 humus Diptera Heleomyzidae Neoleria_sp 1 2.42 yes yes no 0 0 1 0 detritivore
2015 August R63 1 humus Coleoptera Staphylinidae Stenus_clavicornis 1 0.48 yes yes no 0 1 0 0 predator
2015 August R63 1 humus_mineral Hymenoptera Formicidae Myrmica_ruginodis 6 4.11 Yes Yes no 0 1 0 0 predator
2015 August R63 1 humus_mineral Myriapoda Lithobiidae Lithobius_curtipes 1 1.04 yes yes no 0 1 0 0 predator
2015 August R63 1 humus_mineral Coleoptera Staphylinidae Othius_subuliformis 1 0.37 yes yes no 0 1 0 0 predator
2015 August R63 1 humus_mineral Coleoptera Staphylinidae Othius_subuliformis 1 0.55 yes yes no 0 1 0 0 predator
2015 August R63 1 mineral Coleoptera Elateridae Selatosomus_melancholicus 1 7.27 yes yes yes 0.6 0.13 0.13 0.13 combined
2015 August R63 2 humus Coleoptera Elateridae Paraphotistus_impressus 1 8.38 yes yes yes 0.6 0.13 0.13 0.13 combined
2015 August R63 2 humus_mineral Hymenoptera Formicidae Myrmica_ruginodis 2 1.43 Yes Yes no 0 1 0 0 predator
2015 August R63 2 humus_mineral Araneae Linyphiidae Asthenargus_paganus 2 0.00 yes yes no 0 1 0 0 predator
2015 August R63 2 humus_mineral Araneae Linyphiidae Tapinocyba_pallens 3 0.00 yes yes no 0 1 0 0 predator
2015 August R63 2 humus_mineral Myriapoda Lithobiidae Lithobius_curtipes 1 0.55 yes yes no 0 1 0 0 predator
2015 August R63 2 humus_mineral Myriapoda Lithobiidae Lithobius_sp 1 0.24 yes yes no 0 1 0 0 predator
2015 August R63 2 humus_mineral Araneae Lycosidae Pardosa_sp 2 1.08 yes yes no 0 1 0 0 predator
2015 August R63 2 mineral Coleoptera Staphylinidae Xantholinus_tricolor 1 0.25 yes yes no 0 1 0 0 predator
2015 August R63 3 humus Annelida Lumbricidae Dendrodrilus_rubidus_tenuis 1 8.45 yes yes no 0 0 1 0 detritivore
2015 August R63 3 humus Hemiptera Miridae Lygus_punctatus 1 5.16 no no no 1 0 0 0 herbivore
2015 August R63 3 humus_mineral Araneae Linyphiidae Gen_sp 1 1.84 yes yes no 0 1 0 0 predator
2015 August R63 3 humus_mineral Araneae Salticidae Gen_sp 1 0.00 yes yes no 0 1 0 0 predator
2015 August R63 3 humus_mineral Coleoptera Staphylinidae Liogluta_micans 1 0.55 yes yes no 0 1 0 0 predator
2015 August R63 3 humus_mineral Coleoptera Staphylinidae Stenus_clavicornis 1 0.68 yes yes no 0 1 0 0 predator
2015 August R63 3 mineral Coleoptera Staphylinidae Xantholinus_distans 1 1.13 yes yes no 0 1 0 0 predator
2015 August R63 4 humus Annelida Lumbricidae Dendrobaena_octaedra 1 30.48 yes yes no 0 0 1 0 detritivore
2015 August R63 5 humus Diptera Empididae Phyllodromia_melanocephala 1 0.50 yes yes no 0 1 0 0 predator
2015 August R63 5 humus Diptera Rhagionidae Rhagio_lineola 1 1.24 yes yes no 0 1 0 0 predator
2015 August R63 5 humus Diptera Rhagionidae Rhagio_lineola 1 1.76 yes yes no 0 1 0 0 predator
2015 August R63 5 humus Diptera Rhagionidae Rhagio_lineola 1 1.95 yes yes no 0 1 0 0 predator
2015 August R63 5 humus Diptera Rhagionidae Rhagio_lineola 1 1.96 yes yes no 0 1 0 0 predator
2015 August R63 5 humus Coleoptera Staphylinidae Oxypoda_annularis 1 0.08 yes yes no 0 1 0 0 predator
2015 August R63 5 humus Coleoptera Elateridae Paraphotistus_impressus 2 31.32 yes yes yes 0.6 0.13 0.13 0.13 combined
2015 August R63 5 humus Coleoptera Curculionidae Strophosoma_capitatum 1 3.44 yes yes yes 1 0 0 0 herbivore
2015 August R63 5 humus Hemiptera Cydnidae Adomerus_biguttatus 1 5.16 yes yes yes 1 0 0 0 herbivore
2015 August R63 5 humus_mineral Araneae Linyphiidae Minyriolus_pusillus 1 0.00 yes yes no 0 1 0 0 predator
2015 August R63 5 humus_mineral Araneae Linyphiidae Tapinocyba_pallens 1 0.42 yes yes no 0 1 0 0 predator
2015 August R63 5 humus_mineral Araneae Linyphiidae Walckenaeria_dysderoides 1 0.00 yes yes no 0 1 0 0 predator
2015 August R63 5 humus_mineral Myriapoda Lithobiidae Lithobius_sp 1 0.40 yes yes no 0 1 0 0 predator
2015 August R63 5 humus_mineral Coleoptera Staphylinidae Geostiba_circellaris 1 0.07 yes yes no 0 1 0 0 predator
2015 August R63 5 mineral Coleoptera Elateridae Paraphotistus_impressus 1 3.80 yes yes yes 0.6 0.13 0.13 0.13 combined

2015 August R63 6 humus Annelida Lumbricidae Eiseniella_tetraedra 1 1.04 yes yes no 0 0 1 0 detritivore
2015 August R63 6 humus Coleoptera Staphylinidae Xantholinus_distans 1 0.71 yes yes no 0 1 0 0 predator
2015 August R63 6 humus Coleoptera Curculionidae Strophosoma_capitatum 1 2.32 yes yes yes 1 0 0 0 herbivore
2015 August R63 6 humus_mineral Coleoptera Carabidae Calathus_micropterus 1 4.45 yes yes no 0 1 0 0 predator
2015 August R63 6 humus_mineral Araneae Linyphiidae Minyriolus_pusillus 1 0.00 yes yes no 0 1 0 0 predator
2015 August R63 6 humus_mineral Araneae Lycosidae Trochosa_sp 1 12.41 yes yes no 0 1 0 0 predator
2015 August R63 6 humus_mineral Coleoptera Staphylinidae Philonthus_nigrita 1 1.50 yes yes no 0 1 0 0 predator
2015 August R63 6 humus_mineral Araneae Theridiidae Robertus_sp 1 0.00 yes yes no 0 1 0 0 predator
2015 August R63 6 mineral Coleoptera Elateridae Dalopius_marginatus 1 3.70 yes yes yes 0 0.8 0.1 0.1 combined
2015 August R63 7 humus Coleoptera Elateridae Dalopius_marginatus 3 7.95 yes yes yes 0 0.8 0.1 0.1 combined
2015 August R63 7 humus Diptera Rhagionidae Rhagio_lineola 1 0.50 yes yes no 0 1 0 0 predator
2015 August R63 7 humus Diptera Rhagionidae Rhagio_lineola 1 0.82 yes yes no 0 1 0 0 predator
2015 August R63 7 humus Diptera Rhagionidae Rhagio_lineola 1 1.87 yes yes no 0 1 0 0 predator
2015 August R63 7 humus Diptera Rhagionidae Rhagio_lineola 1 2.67 yes yes no 0 1 0 0 predator
2015 August R63 7 humus Coleoptera Elateridae Sericus_brunneus 1 18.56 yes yes yes 0.5 0 0 0.5 combined
2015 August R63 7 humus_mineral Hymenoptera Formicidae Myrmica_ruginodis 1 1.46 Yes Yes no 0 1 0 0 predator
2015 August R63 7 humus_mineral Araneae Linyphiidae Gen_sp 1 0.05 yes yes no 0 1 0 0 predator
2015 August R63 7 humus_mineral Coleoptera Staphylinidae Othius_punctulatus 1 1.68 yes yes no 0 1 0 0 predator
2015 August R63 7 humus_mineral Coleoptera Staphylinidae Othius_subuliformis 1 0.58 yes yes no 0 1 0 0 predator
2015 August R63 7 mineral Coleoptera Elateridae Dalopius_marginatus 4 6.10 yes yes yes 0 0.8 0.1 0.1 combined
2015 August R63 8 humus_mineral Hymenoptera Formicidae Myrmica_ruginodis 1 1.03 Yes Yes no 0 1 0 0 predator
2015 August R63 8 humus_mineral Araneae Linyphiidae Centromerus_incilium 1 0.00 yes yes no 0 1 0 0 predator
2015 August R63 8 humus_mineral Araneae Theridiidae Robertus_sp 1 0.72 yes yes no 0 1 0 0 predator
2015 August R63 8 mineral Coleoptera Elateridae Paraphotistus_impressus 1 2.32 yes yes yes 0.6 0.13 0.13 0.13 combined
2015 August R63 9 humus Coleoptera Elateridae Athous_subfuscus 2 5.51 yes yes yes 0 0.8 0.1 0.1 combined
2015 August R63 9 humus Diptera Rhagionidae Rhagio_lineola 1 1.31 yes yes no 0 1 0 0 predator
2015 August R63 9 humus Diptera Rhagionidae Rhagio_lineola 1 1.59 yes yes no 0 1 0 0 predator
2015 August R63 9 humus Coleoptera Staphylinidae . 1 0.43 yes yes no 0 1 0 0 predator
2015 August R63 9 humus Coleoptera Staphylinidae . 1 1.49 yes yes no 0 1 0 0 predator
2015 August R63 9 humus_mineral Coleoptera Carabidae Calathus_micropterus 1 2.76 yes yes no 0 1 0 0 predator
2015 August R63 9 humus_mineral Coleoptera Carabidae Calathus_micropterus 1 4.23 yes yes no 0 1 0 0 predator
2015 August R63 9 humus_mineral Araneae Linyphiidae Centromerus_arcanus 1 0.25 yes yes no 0 1 0 0 predator
2015 August R63 9 humus_mineral Araneae Linyphiidae Gen_sp 1 0.00 yes yes no 0 1 0 0 predator
2015 August R63 9 mineral Coleoptera Elateridae Athous_subfuscus 1 4.13 yes yes yes 0 0.8 0.1 0.1 combined
2015 August R63 10 humus Coleoptera Staphylinidae Othius_subuliformis 1 0.08 yes yes no 0 1 0 0 predator
2015 August R63 10 humus Coleoptera Staphylinidae Othius_subuliformis 1 0.48 yes yes no 0 1 0 0 predator
2015 August R63 10 humus Coleoptera Chrysomelidae Lochmaea_caprea 1 1.66 no no no 1 0 0 0 herbivore
2015 August R63 10 humus Lepidoptera Hepialidae Korscheltellus_fusconebulosa 1 0.21 Yes Yes yes 1 0 0 0 herbivore
2015 August R63 10 humus_mineral Hymenoptera Formicidae Myrmica_ruginodis 1 1.30 Yes Yes no 0 1 0 0 predator
2015 August R63 10 humus_mineral Araneae Hahniidae Hahnia_pusilla 1 0.00 yes yes no 0 1 0 0 predator
2015 August R63 10 humus_mineral Araneae Hahniidae Hahnia_sp 3 0.00 yes yes no 0 1 0 0 predator
2015 August R63 10 humus_mineral Araneae Linyphiidae Diplostyla_concolor 1 0.00 yes yes no 0 1 0 0 predator
2015 August R63 10 humus_mineral Araneae Linyphiidae Porrhomma_pallidum 1 0.00 yes yes no 0 1 0 0 predator
2015 August R63 10 humus_mineral Araneae Linyphiidae Tapinocyba_pallens 1 4.02 yes yes no 0 1 0 0 predator
2015 August R63 10 humus_mineral Araneae Linyphiidae Walckenaeria_alticeps 1 0.00 yes yes no 0 1 0 0 predator
2015 August R63 10 humus_mineral Araneae Linyphiidae Walckenaeria_cucullata 1 0.00 yes yes no 0 1 0 0 predator
2015 August R63 10 humus_mineral Araneae Lycosidae Pardosa_sp 2 0.00 yes yes no 0 1 0 0 predator
2015 August R63 10 humus_mineral Coleoptera Staphylinidae Othius_subuliformis 1 0.53 yes yes no 0 1 0 0 predator
2015 August R63 10 humus_mineral Coleoptera Staphylinidae Othius_subuliformis 1 0.54 yes yes no 0 1 0 0 predator
2015 August R63 10 humus_mineral Araneae Thomisidae Ozyptila_sp 1 0.00 yes yes no 0 1 0 0 predator
2015 August R64 1 humus Myriapoda Polyzonidae Polyzonium_germanicum 1 0.94 yes yes no 0 0 1 0 detritivore
2015 August R64 1 humus Coleoptera Elateridae Athous_subfuscus 1 3.59 yes yes yes 0 0.8 0.1 0.1 combined
2015 August R64 1 humus Coleoptera Cantharidae . 1 0.16 yes yes no 0 1 0 0 predator

2015 August R64 1 humus Diptera Rhagionidae Rhagio_scolopaceus 1 13.64 yes yes no 0 1 0 0 predator
2015 August R64 1 humus Coleoptera Staphylinidae . 1 0.30 yes yes no 0 1 0 0 predator
2015 August R64 1 humus Coleoptera . . 1 0.69 . . . .
2015 August R64 1 humus_mineral Araneae Linyphiidae Centromerus_arcanus 1 0.00 yes yes no 0 1 0 0 predator
2015 August R64 1 humus_mineral Myriapoda Lithobiidae Lithobius_curtipes 1 1.42 yes yes no 0 1 0 0 predator
2015 August R64 1 humus_mineral Coleoptera Staphylinidae Atheta_myrmecobia 1 0.11 yes yes no 0 1 0 0 predator
2015 August R64 1 humus_mineral Coleoptera Staphylinidae Atheta_myrmecobia 1 0.15 yes yes no 0 1 0 0 predator
2015 August R64 1 humus_mineral Coleoptera Staphylinidae Othius_subuliformis 1 0.60 yes yes no 0 1 0 0 predator
2015 August R64 1 humus_mineral Araneae Theridiidae Robertus_scoticus 1 0.00 yes yes no 0 1 0 0 predator
2015 August R64 1 humus_mineral Araneae Theridiidae Robertus_sp 1 0.55 yes yes no 0 1 0 0 predator
2015 August R64 2 humus Diptera Rhagionidae Rhagio_scolopaceus 1 0.41 yes yes no 0 1 0 0 predator
2015 August R64 2 humus_mineral Araneae Linyphiidae Gen_sp 1 0.15 yes yes no 0 1 0 0 predator
2015 August R64 2 humus_mineral Myriapoda Lithobiidae Lithobius_curtipes 1 0.47 yes yes no 0 1 0 0 predator
2015 August R64 2 humus_mineral Myriapoda Lithobiidae Lithobius_curtipes 1 1.67 yes yes no 0 1 0 0 predator
2015 August R64 2 humus_mineral Myriapoda Lithobiidae Lithobius_curtipes 1 2.02 yes yes no 0 1 0 0 predator
2015 August R64 3 humus Coleoptera Elateridae Athous_subfuscus 8 20.64 yes yes yes 0 0.8 0.1 0.1 combined
2015 August R64 3 humus Coleoptera Cantharidae Podistra_schoenherri 1 4.19 yes yes no 0 1 0 0 predator
2015 August R64 3 humus_mineral Araneae Theridiidae Robertus_sp 2 0.00 yes yes no 0 1 0 0 predator
2015 August R64 3 humus_mineral Araneae Thomisidae Ozyptila_sp 1 1.89 yes yes no 0 1 0 0 predator
2015 August R64 4 humus Annelida Lumbricidae Eiseniella_tetraedra 1 0.18 yes yes no 0 0 1 0 detritivore
2015 August R64 4 humus Coleoptera Elateridae Eanus_costalis 1 3.58 yes yes no 0 0.5 0.5 0 combined
2015 August R64 4 humus_mineral Coleoptera Staphylinidae Othius_subuliformis 1 0.68 yes yes no 0 1 0 0 predator
2015 August R64 4 humus_mineral Araneae Theridiidae Robertus_scoticus 2 8.32 yes yes no 0 1 0 0 predator
2015 August R64 4 humus_mineral Araneae Thomisidae Ozyptila_sp 2 0.00 yes yes no 0 1 0 0 predator
2015 August R64 4 humus_mineral Araneae Thomisidae Ozyptila_trux 1 0.00 yes yes no 0 1 0 0 predator
2015 August R64 5 humus Coleoptera Leiodidae Amphicyllis_globus 1 0.80 yes yes no 0 0 0 1 fungivore
2015 August R64 5 humus Annelida Lumbricidae Dendrodrilus_rubidus_tenuis 1 15.24 yes yes no 0 0 1 0 detritivore
2015 August R64 5 humus Coleoptera Staphylinidae Othius_subuliformis 1 0.48 yes yes no 0 1 0 0 predator
2015 August R64 5 humus_mineral Coleoptera Carabidae Calathus_micropterus 1 5.01 yes yes no 0 1 0 0 predator
2015 August R64 5 humus_mineral Coleoptera Staphylinidae Mocyta_fungi 1 0.16 yes yes no 0 1 0 0 predator
2015 August R64 6 humus Coleoptera Elateridae Eanus_costalis 1 1.16 yes yes no 0 0.5 0.5 0 combined
2015 August R64 6 humus Coleoptera Staphylinidae . 1 0.43 yes yes no 0 1 0 0 predator
2015 August R64 6 humus_mineral Araneae Linyphiidae Porrhomma_pallidum 1 0.24 yes yes no 0 1 0 0 predator
2015 August R64 6 humus_mineral Myriapoda Lithobiidae Lithobius_sp 1 1.03 yes yes no 0 1 0 0 predator
2015 August R64 7 humus Coleoptera Cantharidae Malthodes_brevicollis 1 0.15 yes yes no 0 1 0 0 predator
2015 August R64 7 humus Diptera Rhagionidae Rhagio_scolopaceus 1 0.16 yes yes no 0 1 0 0 predator
2015 August R64 7 humus Diptera Rhagionidae Rhagio_scolopaceus 1 0.16 yes yes no 0 1 0 0 predator
2015 August R64 7 humus_mineral Myriapoda Lithobiidae Lithobius_sp 1 0.61 yes yes no 0 1 0 0 predator
2015 August R64 7 humus_mineral Araneae Theridiidae Robertus_scoticus 1 0.00 yes yes no 0 1 0 0 predator
2015 August R64 7 humus_mineral Araneae Theridiidae Robertus_sp 1 0.37 yes yes no 0 1 0 0 predator
2015 August R64 8 humus Diptera Rhagionidae Rhagio_scolopaceus 1 1.24 yes yes no 0 1 0 0 predator
2015 August R64 8 humus_mineral Araneae Linyphiidae Tapinocyba_pallens 1 0.14 yes yes no 0 1 0 0 predator
2015 August R64 8 humus_mineral Myriapoda Lithobiidae Lithobius_curtipes 1 0.47 yes yes no 0 1 0 0 predator
2015 August R64 9 humus Diptera Lonchaeidae Lonchaea_ragnari 1 1.73 yes yes no 0 0 1 0 detritivore
2015 August R64 9 humus Diptera Lonchaeidae Lonchaea_ragnari 1 2.71 yes yes no 0 0 1 0 detritivore
2015 August R64 9 humus Annelida Lumbricidae Dendrobaena_octaedra 1 3.40 yes yes no 0 0 1 0 detritivore
2015 August R64 9 humus Coleoptera Elateridae Eanus_costalis 1 9.17 yes yes yes 0 0.5 0.5 0 combined
2015 August R64 9 humus_mineral Coleoptera Staphylinidae Acrotona_silvicola 1 0.06 yes yes no 0 1 0 0 predator
2015 August R64 9 humus_mineral Coleoptera Staphylinidae Oxypoda_annularis 1 0.07 yes yes no 0 1 0 0 predator
2015 August R64 9 humus_mineral Coleoptera Staphylinidae Philonthus_decorus 1 2.52 yes yes no 0 1 0 0 predator
2015 August R64 10 humus_mineral Coleoptera Staphylinidae Sepedophilus_immaculatus 1 0.23 yes yes no 0 0 0 1 fungivore
2015 August R64 10 humus_mineral Araneae Linyphiidae Tapinocyba_pallens 2 2.37 yes yes no 0 1 0 0 predator
2015 August R64 10 humus_mineral Araneae Theridiidae Robertus_sp 4 0.00 yes yes no 0 1 0 0 predator

2015 August R64 10 mineral Coleoptera Elateridae Paraphotistus_impressus 2 43.36 yes yes yes 0.6 0.13 0.13 0.13 combined
2015 August R65 1 humus Annelida Lumbricidae Dendrobaena_octaedra 2 9.16 yes yes no 0 0 1 0 detritivore
2015 August R65 1 humus Annelida Lumbricidae Eiseniella_tetraedra 1 0.23 yes yes no 0 0 1 0 detritivore
2015 August R65 1 humus Coleoptera Elateridae Eanus_costalis 1 1.60 yes yes no 0 0.5 0.5 0 combined
2015 August R65 1 humus Myriapoda Lithobiidae Lithobius_curtipes 1 0.20 yes yes no 0 1 0 0 predator
2015 August R65 1 humus Coleoptera Staphylinidae . 1 0.53 yes yes no 0 1 0 0 predator
2015 August R65 1 humus Coleoptera Elateridae Paraphotistus_impressus 1 3.54 yes yes yes 0.6 0.13 0.13 0.13 combined
2015 August R65 1 humus_mineral Araneae Linyphiidae Minyriolus_pusillus 1 0.00 yes yes no 0 1 0 0 predator
2015 August R65 1 humus_mineral Araneae Linyphiidae Tapinocyba_pallens 1 0.00 yes yes no 0 1 0 0 predator
2015 August R65 1 humus_mineral Myriapoda Lithobiidae Lithobius_curtipes 1 0.59 yes yes no 0 1 0 0 predator
2015 August R65 1 humus_mineral Coleoptera Staphylinidae Stenus_clavicornis 1 0.75 yes yes no 0 1 0 0 predator
2015 August R65 1 humus_mineral Araneae Theridiidae Robertus_scoticus 1 0.46 yes yes no 0 1 0 0 predator
2015 August R65 2 humus Coleoptera Elateridae Ampedus_balteatus 1 2.05 yes yes no 0 0.5 0.5 0 combined
2015 August R65 2 humus Coleoptera Elateridae Athous_subfuscus 1 1.10 yes yes yes 0 0.8 0.1 0.1 combined
2015 August R65 2 humus Coleoptera Staphylinidae Othius_subuliformis 1 0.62 yes yes no 0 1 0 0 predator
2015 August R65 2 humus_mineral Hymenoptera Formicidae Myrmica_ruginodis 1 0.56 Yes Yes no 0 1 0 0 predator
2015 August R65 2 humus_mineral Araneae Linyphiidae Tapinocyba_pallens 1 0.00 yes yes no 0 1 0 0 predator
2015 August R65 2 humus_mineral Myriapoda Lithobiidae Lithobius_curtipes 1 2.05 yes yes no 0 1 0 0 predator
2015 August R65 2 humus_mineral Araneae Thomisidae Xysticus_sp 1 6.06 yes yes no 0 1 0 0 predator
2015 August R65 3 humus Coleoptera Elateridae Ampedus_cf_balteatus 1 0.80 yes yes no 0 0.5 0.5 0 combined
2015 August R65 3 humus Coleoptera Elateridae Eanus_costalis 1 0.78 yes yes no 0 0.5 0.5 0 combined
2015 August R65 3 humus Coleoptera Elateridae Paraphotistus_impressus 1 4.12 yes yes yes 0.6 0.13 0.13 0.13 combined
2015 August R65 3 humus_mineral Coleoptera Elateridae Eanus_costalis 1 12.07 yes yes yes 0 0.5 0.5 0 combined
2015 August R65 3 humus_mineral Coleoptera Elateridae Eanus_costalis 1 8.07 yes yes yes 0 0.5 0.5 0 combined
2015 August R65 3 humus_mineral Araneae Theridiidae Robertus_sp 1 0.09 yes yes no 0 1 0 0 predator
2015 August R65 4 humus Coleoptera Elateridae Eanus_costalis 1 10.39 yes yes no 0 0.5 0.5 0 combined
2015 August R65 4 humus Coleoptera Elateridae Athous_subfuscus 1 2.27 yes yes yes 0 0.8 0.1 0.1 combined
2015 August R65 4 humus Coleoptera Cantharidae . 1 0.11 yes yes no 0 1 0 0 predator
2015 August R65 4 humus Coleoptera Elateridae Paraphotistus_impressus 1 4.34 yes yes yes 0.6 0.13 0.13 0.13 combined
2015 August R65 4 humus_mineral Araneae Linyphiidae Tapinocyba_pallens 1 0.11 yes yes no 0 1 0 0 predator
2015 August R65 5 humus Coleoptera Cantharidae Malthodes_fuscus 1 0.48 yes yes no 0 1 0 0 predator
2015 August R65 5 mineral Coleoptera Elateridae Paraphotistus_impressus 1 1.22 yes yes yes 0.6 0.13 0.13 0.13 combined
2015 August R65 6 humus Coleoptera Elateridae Athous_subfuscus 1 0.33 yes yes yes 0 0.8 0.1 0.1 combined
2015 August R65 6 humus Coleoptera Cantharidae . 1 0.13 yes yes no 0 1 0 0 predator
2015 August R65 6 humus Coleoptera Cantharidae Podistra_schoenherri 1 1.84 yes yes no 0 1 0 0 predator
2015 August R65 6 humus Hemiptera Lygaeidae Drymus_brunneus 1 0.89 yes yes no 1 0 0 0 herbivore
2015 August R65 6 humus_mineral Hymenoptera Formicidae Myrmica_ruginodis 1 1.02 Yes Yes no 0 1 0 0 predator
2015 August R65 6 humus_mineral Araneae Linyphiidae Tapinocyba_pallens 1 0.00 yes yes no 0 1 0 0 predator
2015 August R65 6 humus_mineral Araneae Miturgidae Zora_sp 1 1.18 yes yes no 0 1 0 0 predator
2015 August R65 6 humus_mineral Araneae Theridiidae Robertus_sp 1 0.00 yes yes no 0 1 0 0 predator
2015 August R65 7 humus Annelida Lumbricidae Dendrobaena_octaedra 1 24.24 yes yes no 0 0 1 0 detritivore
2015 August R65 7 humus Coleoptera Cantharidae Podistra_schoenherri 1 1.82 yes yes no 0 1 0 0 predator
2015 August R65 7 humus Coleoptera Carabidae Calathus_melanocephalus 1 1.20 yes yes no 0 1 0 0 predator
2015 August R65 7 humus Coleoptera Staphylinidae . 1 0.38 yes yes no 0 1 0 0 predator
2015 August R65 7 humus_mineral Araneae Linyphiidae Gen_sp 1 0.00 yes yes no 0 1 0 0 predator
2015 August R65 7 humus_mineral Araneae Linyphiidae Tapinocyba_pallens 3 0.88 yes yes no 0 1 0 0 predator
2015 August R65 7 humus_mineral Araneae Linyphiidae Tibioplus_diversus 1 0.00 yes yes no 0 1 0 0 predator
2015 August R65 7 humus_mineral Myriapoda Lithobiidae Lithobius_curtipes 1 0.21 yes yes no 0 1 0 0 predator
2015 August R65 7 humus_mineral Myriapoda Lithobiidae Lithobius_curtipes 1 0.71 yes yes no 0 1 0 0 predator
2015 August R65 7 humus_mineral Myriapoda Lithobiidae Lithobius_curtipes 1 1.07 yes yes no 0 1 0 0 predator
2015 August R65 8 humus Coleoptera Cantharidae Malthodes_mysticus 1 0.41 yes yes no 0 1 0 0 predator
2015 August R65 8 humus Coleoptera Staphylinidae Othius_lapidicola 1 0.66 yes yes no 0 1 0 0 predator
2015 August R65 8 humus Hemiptera Lygaeidae Eremocoris_abietis 1 1.99 yes no no 1 0 0 0 herbivore

2015 August R65 8 humus_mineral Araneae Linyphiidae Tapinocyba_pallens 1 2.18 yes yes no 0 1 0 0 predator
2015 August R65 8 humus_mineral Araneae Thomisidae Ozyptila_sp 1 0.00 yes yes no 0 1 0 0 predator
2015 August R65 8 mineral Coleoptera Elateridae Eanus_costalis 1 2.55 yes yes no 0 0.5 0.5 0 combined
2015 August R65 8 mineral Coleoptera Elateridae Athous_subfuscus 1 2.15 yes yes yes 0 0.8 0.1 0.1 combined
2015 August R65 9 humus Coleoptera Elateridae Athous_subfuscus 2 15.05 yes yes yes 0 0.8 0.1 0.1 combined
2015 August R65 10 humus Annelida Lumbricidae Dendrobaena_octaedra 1 21.60 yes yes no 0 0 1 0 detritivore
2015 August R65 10 humus Coleoptera Elateridae Athous_subfuscus 1 4.80 yes yes yes 0 0.8 0.1 0.1 combined
2015 August R65 10 humus Coleoptera Curculionidae Otiorhynchus_nodosus 1 5.87 yes yes yes 1 0 0 0 herbivore
2015 August R65 10 humus_mineral Coleoptera Staphylinidae Othius_subuliformis 1 0.61 yes yes no 0 1 0 0 predator
2015 August R66 1 humus_mineral Myriapoda Lithobiidae Lithobius_sp 1 0.21 yes yes no 0 1 0 0 predator
2015 August R66 2 humus_mineral Araneae Linyphiidae Tapinocyba_pallens 1 0.13 yes yes no 0 1 0 0 predator
2015 August R66 3 humus_mineral Coleoptera Staphylinidae Bolitochara_pulchra 1 0.42 yes yes no 0 1 0 0 predator
2015 August R66 3 humus_mineral Coleoptera Staphylinidae Oxypoda_annularis 1 0.07 yes yes no 0 1 0 0 predator
2015 August R66 4 humus Coleoptera Elateridae Eanus_costalis 2 4.88 yes yes no 0 0.5 0.5 0 combined
2015 August R66 4 humus Coleoptera Staphylinidae Stenus_clavicornis 1 0.51 yes yes no 0 1 0 0 predator
2015 August R66 4 humus_mineral Araneae Theridiidae Robertus_scoticus 1 0.55 yes yes no 0 1 0 0 predator
2015 August R66 5 humus Coleoptera Elateridae Athous_subfuscus 1 0.41 yes yes yes 0 0.8 0.1 0.1 combined
2015 August R66 5 humus Coleoptera Staphylinidae Othius_lapidicola 1 0.21 yes yes no 0 1 0 0 predator
2015 August R66 5 humus_mineral Myriapoda Lithobiidae Lithobius_curtipes 1 0.55 yes yes no 0 1 0 0 predator
2015 August R66 6 humus Annelida Lumbricidae . 1 0.31 yes yes no 0 0 1 0 detritivore
2015 August R66 6 humus Coleoptera Elateridae Paraphotistus_impressus 1 2.24 yes yes yes 0.6 0.13 0.13 0.13 combined
2015 August R66 7 humus Araneae Linyphiidae Gen_sp 1 0.04 yes yes no 0 1 0 0 predator
2015 August R66 7 humus_mineral Araneae Linyphiidae Tapinocyba_pallens 2 0.16 yes yes no 0 1 0 0 predator
2015 August R66 7 humus_mineral Myriapoda Lithobiidae Lithobius_curtipes 1 0.86 yes yes no 0 1 0 0 predator
2015 August R66 8 humus Coleoptera Elateridae Athous_subfuscus 1 2.93 yes yes yes 0 0.8 0.1 0.1 combined
2015 August R66 8 humus Coleoptera Curculionidae Otiorhynchus_nodosus 1 10.80 yes yes yes 1 0 0 0 herbivore
2015 August R66 8 humus_mineral Araneae Linyphiidae Tenuiphantes_alacris 1 1.02 yes yes no 0 1 0 0 predator
2015 August R66 9 humus Coleoptera Cantharidae Podistra_schoenherri 1 0.42 yes yes no 0 1 0 0 predator
2015 August R66 9 humus Coleoptera Cantharidae Podistra_schoenherri 1 5.41 yes yes no 0 1 0 0 predator
2015 August R66 9 humus_mineral Araneae Linyphiidae Gen_sp 1 0.09 yes yes no 0 1 0 0 predator
2015 August R66 9 humus_mineral Myriapoda Lithobiidae Lithobius_curtipes 1 0.33 yes yes no 0 1 0 0 predator
2015 August R66 10 humus Coleoptera Staphylinidae Othius_lapidicola 1 0.28 yes yes no 0 1 0 0 predator
2015 August R66 10 humus Coleoptera Staphylinidae Othius_lapidicola 1 0.32 yes yes no 0 1 0 0 predator
2015 August R66 10 humus Coleoptera Elateridae Paraphotistus_impressus 1 6.60 yes yes yes 0.6 0.13 0.13 0.13 combined
2015 August R66 10 humus_mineral Myriapoda Lithobiidae Lithobius_curtipes 1 1.85 yes yes no 0 1 0 0 predator
2015 August R66 10 humus_mineral Coleoptera Staphylinidae Mocyta_fungi 1 0.08 yes yes no 0 1 0 0 predator
2015 August R66 10 humus_mineral Coleoptera Staphylinidae Othius_lapidicola 1 0.80 yes yes no 0 1 0 0 predator
2015 August R67 1 humus Coleoptera Elateridae Eanus_costalis 4 19.26 yes yes no 0 0.5 0.5 0 combined
2015 August R67 1 humus Coleoptera Cantharidae Podistra_schoenherri 1 0.84 yes yes no 0 1 0 0 predator
2015 August R67 1 humus_mineral Araneae Linyphiidae Centromerus_arcanus 1 0.00 yes yes no 0 1 0 0 predator
2015 August R67 1 humus_mineral Araneae Linyphiidae Diplocentria_rectangulata 1 0.00 yes yes no 0 1 0 0 predator
2015 August R67 1 humus_mineral Araneae Linyphiidae Minyriolus_pusillus 1 0.00 yes yes no 0 1 0 0 predator
2015 August R67 1 humus_mineral Araneae Linyphiidae Sisicus_apertus 1 0.00 yes yes no 0 1 0 0 predator
2015 August R67 1 humus_mineral Araneae Linyphiidae Tapinocyba_pallens 6 0.00 yes yes no 0 1 0 0 predator
2015 August R67 1 humus_mineral Myriapoda Lithobiidae Lithobius_curtipes 1 1.26 yes yes no 0 1 0 0 predator
2015 August R67 1 humus_mineral Myriapoda Lithobiidae Lithobius_curtipes 1 2.24 yes yes no 0 1 0 0 predator
2015 August R67 1 humus_mineral Coleoptera Staphylinidae Ischnosoma_splendidum 1 0.35 yes yes no 0 1 0 0 predator
2015 August R67 1 humus_mineral Araneae Theridiidae Robertus_scoticus 2 0.00 yes yes no 0 1 0 0 predator
2015 August R67 1 humus_mineral Araneae Theridiidae Robertus_sp 5 0.00 yes yes no 0 1 0 0 predator
2015 August R67 1 humus_mineral Araneae Thomisidae Ozyptila_sp 1 11.60 yes yes no 0 1 0 0 predator
2015 August R67 1 humus_mineral Araneae Thomisidae Ozyptila_trux 1 0.00 yes yes no 0 1 0 0 predator
2015 August R67 2 humus Annelida Lumbricidae Eiseniella_tetraedra 1 0.49 yes yes no 0 0 1 0 detritivore
2015 August R67 2 humus Coleoptera Elateridae Eanus_costalis 1 1.47 yes yes no 0 0.5 0.5 0 combined

2015 August R67 2 humus Diptera Rhagionidae Rhagio_scolopaceus 1 13.43 yes yes no 0 1 0 0 predator
2015 August R67 2 humus_mineral Araneae Linyphiidae Sisicus_apertus 2 0.00 yes yes no 0 1 0 0 predator
2015 August R67 2 humus_mineral Araneae Linyphiidae Tapinocyba_pallens 4 0.00 yes yes no 0 1 0 0 predator
2015 August R67 2 humus_mineral Araneae Theridiidae Robertus_scoticus 3 2.39 yes yes no 0 1 0 0 predator
2015 August R67 3 humus Coleoptera Elateridae Eanus_costalis 2 1.49 yes yes no 0 0.5 0.5 0 combined
2015 August R67 3 humus Coleoptera Elateridae Eanus_costalis 1 7.15 yes yes yes 0 0.5 0.5 0 combined
2015 August R67 3 humus Coleoptera Cantharidae Malthodes_fuscus 1 0.05 yes yes no 0 1 0 0 predator
2015 August R67 3 humus Diptera Rhagionidae Rhagio_scolopaceus 1 2.02 yes yes no 0 1 0 0 predator
2015 August R67 3 humus Coleoptera Staphylinidae Oxypoda_annularis 1 0.03 yes yes no 0 1 0 0 predator
2015 August R67 3 humus Hemiptera Lygaeidae Drymus_brunneus 1 0.37 yes yes no 1 0 0 0 herbivore
2015 August R67 3 humus Coleoptera Polyphaga . 1 0.08 . . . .
2015 August R67 3 humus Coleoptera Polyphaga . 1 0.34 . . . .
2015 August R67 3 humus_mineral Coleoptera Carabidae Notiophilus_biguttatus 1 1.66 yes yes no 0 1 0 0 predator
2015 August R67 3 humus_mineral Araneae Linyphiidae Gen_sp 1 0.00 yes yes no 0 1 0 0 predator
2015 August R67 3 humus_mineral Araneae Linyphiidae Tapinocyba_pallens 1 0.00 yes yes no 0 1 0 0 predator
2015 August R67 3 humus_mineral Myriapoda Lithobiidae Lithobius_sp 1 0.48 yes yes no 0 1 0 0 predator
2015 August R67 3 humus_mineral Araneae Lycosidae Alopecosa_sp 1 0.00 yes yes no 0 1 0 0 predator
2015 August R67 3 humus_mineral Coleoptera Staphylinidae Mycetoporus_monticola 1 0.26 yes yes no 0 1 0 0 predator
2015 August R67 3 humus_mineral Araneae Theridiidae Robertus_sp 3 6.80 yes yes no 0 1 0 0 predator
2015 August R67 4 humus Annelida Lumbricidae Dendrobaena_octaedra 1 1.25 yes yes no 0 0 1 0 detritivore
2015 August R67 4 humus_mineral Araneae Linyphiidae Tapinocyba_pallens 4 0.00 yes yes no 0 1 0 0 predator
2015 August R67 4 humus_mineral Myriapoda Lithobiidae Lithobius_curtipes 1 0.48 yes yes no 0 1 0 0 predator
2015 August R67 4 humus_mineral Araneae Theridiidae Robertus_scoticus 2 1.21 yes yes no 0 1 0 0 predator
2015 August R67 5 humus Annelida Lumbricidae . 1 0.21 yes yes no 0 0 1 0 detritivore
2015 August R67 5 humus Coleoptera Elateridae Eanus_costalis 3 13.28 yes yes no 0 0.5 0.5 0 combined
2015 August R67 5 humus Coleoptera Elateridae Athous_subfuscus 1 0.29 yes yes yes 0 0.8 0.1 0.1 combined
2015 August R67 5 humus_mineral Araneae Linyphiidae Gen_sp 1 0.00 yes yes no 0 1 0 0 predator
2015 August R67 5 humus_mineral Araneae Linyphiidae Sisicus_apertus 1 0.00 yes yes no 0 1 0 0 predator
2015 August R67 5 humus_mineral Araneae Linyphiidae Tapinocyba_pallens 2 0.00 yes yes no 0 1 0 0 predator
2015 August R67 5 humus_mineral Araneae Linyphiidae Tenuiphantes_alacris 1 0.00 yes yes no 0 1 0 0 predator
2015 August R67 5 humus_mineral Myriapoda Lithobiidae Lithobius_curtipes 1 1.18 yes yes no 0 1 0 0 predator
2015 August R67 5 humus_mineral Myriapoda Lithobiidae Lithobius_sp 1 0.08 yes yes no 0 1 0 0 predator
2015 August R67 5 humus_mineral Myriapoda Lithobiidae Lithobius_sp 1 0.09 yes yes no 0 1 0 0 predator
2015 August R67 5 humus_mineral Araneae Theridiidae Robertus_scoticus 3 0.00 yes yes no 0 1 0 0 predator
2015 August R67 5 humus_mineral Araneae Theridiidae Robertus_scoticus 1 2.10 yes yes no 0 1 0 0 predator
2015 August R67 6 humus Annelida Lumbricidae . 1 0.62 yes yes no 0 0 1 0 detritivore
2015 August R67 6 humus Diptera Rhagionidae Rhagio_scolopaceus 1 0.31 yes yes no 0 1 0 0 predator
2015 August R67 6 humus_mineral Araneae Linyphiidae Gen_sp 1 0.00 yes yes no 0 1 0 0 predator
2015 August R67 6 humus_mineral Araneae Linyphiidae Sisicus_apertus 1 0.00 yes yes no 0 1 0 0 predator
2015 August R67 6 humus_mineral Araneae Linyphiidae Tapinocyba_pallens 1 0.34 yes yes no 0 1 0 0 predator
2015 August R67 6 humus_mineral Myriapoda Lithobiidae Lithobius_curtipes 1 0.80 yes yes no 0 1 0 0 predator
2015 August R67 6 humus_mineral Myriapoda Lithobiidae Lithobius_curtipes 1 1.42 yes yes no 0 1 0 0 predator
2015 August R67 6 mineral Diptera Rhagionidae Rhagio_scolopaceus 1 2.32 yes yes no 0 1 0 0 predator
2015 August R67 7 humus Coleoptera Elateridae Eanus_costalis 1 5.58 yes yes no 0 0.5 0.5 0 combined
2015 August R67 7 humus_mineral Hymenoptera Formicidae Myrmica_ruginodis 1 0.54 Yes Yes no 0 1 0 0 predator
2015 August R67 7 humus_mineral Araneae Linyphiidae Gen_sp 1 0.00 yes yes no 0 1 0 0 predator
2015 August R67 7 humus_mineral Myriapoda Lithobiidae Lithobius_curtipes 1 0.31 yes yes no 0 1 0 0 predator
2015 August R67 7 humus_mineral Myriapoda Lithobiidae Lithobius_curtipes 1 0.31 yes yes no 0 1 0 0 predator
2015 August R67 7 humus_mineral Myriapoda Lithobiidae Lithobius_curtipes 1 0.57 yes yes no 0 1 0 0 predator
2015 August R67 7 humus_mineral Araneae Theridiidae Robertus_scoticus 2 0.71 yes yes no 0 1 0 0 predator
2015 August R67 8 humus Diptera Rhagionidae Rhagio_scolopaceus 1 1.52 yes yes no 0 1 0 0 predator
2015 August R67 8 humus_mineral Araneae Linyphiidae Tapinocyba_pallens 1 0.56 yes yes no 0 1 0 0 predator
2015 August R67 8 humus_mineral Araneae Theridiidae Robertus_scoticus 1 0.00 yes yes no 0 1 0 0 predator

2015 August R67 9 humus Coleoptera Elateridae Eanus_costalis 1 4.10 yes yes no 0 0.5 0.5 0 combined
2015 August R67 9 humus Coleoptera Elateridae Athous_subfuscus 1 1.19 yes yes yes 0 0.8 0.1 0.1 combined
2015 August R67 9 humus Coleoptera Cantharidae Podistra_schoenherri 1 0.47 yes yes no 0 1 0 0 predator
2015 August R67 9 humus Coleoptera Cantharidae Podistra_schoenherri 1 4.53 yes yes no 0 1 0 0 predator
2015 August R67 9 humus Coleoptera Elateridae Paraphotistus_impressus 1 1.27 yes yes yes 0.6 0.13 0.13 0.13 combined
2015 August R67 9 humus_mineral Araneae Linyphiidae Gen_sp 1 0.00 yes yes no 0 1 0 0 predator
2015 August R67 9 humus_mineral Araneae Linyphiidae Minyriolus_pusillus 1 0.00 yes yes no 0 1 0 0 predator
2015 August R67 9 humus_mineral Araneae Linyphiidae Sisicus_apertus 1 0.00 yes yes no 0 1 0 0 predator
2015 August R67 9 humus_mineral Coleoptera Staphylinidae Oxypoda_annularis 1 0.09 yes yes no 0 1 0 0 predator
2015 August R67 9 humus_mineral Coleoptera Staphylinidae Oxypoda_annularis 1 0.10 yes yes no 0 1 0 0 predator
2015 August R67 9 humus_mineral Araneae Theridiidae Robertus_scoticus 3 1.60 yes yes no 0 1 0 0 predator
2015 August R67 9 humus_mineral Araneae Theridiidae Robertus_sp 3 0.00 yes yes no 0 1 0 0 predator
2015 August R67 10 humus Coleoptera Elateridae Eanus_costalis 1 1.30 yes yes no 0 0.5 0.5 0 combined
2015 August R67 10 humus Coleoptera Cantharidae Malthodes_fuscus 1 0.63 yes yes no 0 1 0 0 predator
2015 August R67 10 humus_mineral Araneae Linyphiidae Gen_sp 1 0.07 yes yes no 0 1 0 0 predator
2015 August R67 10 humus_mineral Myriapoda Lithobiidae Lithobius_curtipes 1 0.32 yes yes no 0 1 0 0 predator
2015 August R67 10 humus_mineral Myriapoda Lithobiidae Lithobius_curtipes 1 0.80 yes yes no 0 1 0 0 predator
2015 August R67 10 humus_mineral Myriapoda Lithobiidae Lithobius_sp 1 0.07 yes yes no 0 1 0 0 predator
2015 August R68 2 humus Diptera Chironomidae . 1 0.05 yes yes yes 0.25 0 0.75 0 combined
2015 August R68 2 humus Diptera Chironomidae . 1 0.05 yes yes yes 0.25 0 0.75 0 combined
2015 August R68 2 humus Diptera Chironomidae . 1 0.05 yes yes yes 0.25 0 0.75 0 combined
2015 August R68 2 humus Coleoptera Elateridae Sericus_cf_brunneus 1 1.18 yes yes yes 0.5 0 0 0.5 combined
2015 August R68 2 humus_mineral Araneae Linyphiidae Agyneta_gulosa 1 0.16 yes yes no 0 1 0 0 predator
2015 August R68 3 humus Coleoptera Coccinellidae Calvia_quatuordecimguttata 1 3.13 no no no 0 1 0 0 predator
2015 August R68 3 humus Hemiptera Lygaeidae Drymus_brunneus 1 0.27 yes yes no 1 0 0 0 herbivore
2015 August R68 3 humus_mineral Araneae Linyphiidae Gen_sp 1 2.36 yes yes no 0 1 0 0 predator
2015 August R68 3 humus_mineral Myriapoda Lithobiidae Lithobius_curtipes 1 0.78 yes yes no 0 1 0 0 predator
2015 August R68 3 humus_mineral Araneae Theridiidae Robertus_scoticus 1 0.00 yes yes no 0 1 0 0 predator
2015 August R68 3 humus_mineral Araneae Theridiidae Robertus_sp 1 0.00 yes yes no 0 1 0 0 predator
2015 August R68 3 humus_mineral Araneae Thomisidae Ozyptila_sp 1 0.00 yes yes no 0 1 0 0 predator
2015 August R68 4 humus_mineral Araneae Linyphiidae Gen_sp 1 0.08 yes yes no 0 1 0 0 predator
2015 August R68 5 humus Diptera Chironomidae . 1 0.03 yes yes yes 0.25 0 0.75 0 combined
2015 August R68 5 humus Diptera Chironomidae . 1 0.03 yes yes yes 0.25 0 0.75 0 combined
2015 August R68 5 humus Diptera Chironomidae . 1 0.03 yes yes yes 0.25 0 0.75 0 combined
2015 August R68 5 humus Diptera Chironomidae . 1 0.03 yes yes yes 0.25 0 0.75 0 combined
2015 August R68 5 humus Diptera Chironomidae . 1 0.03 yes yes yes 0.25 0 0.75 0 combined
2015 August R68 5 humus Diptera Chironomidae . 1 0.03 yes yes yes 0.25 0 0.75 0 combined
2015 August R68 5 humus Diptera Chironomidae . 1 0.03 yes yes yes 0.25 0 0.75 0 combined
2015 August R68 5 humus Diptera Chironomidae . 1 0.03 yes yes yes 0.25 0 0.75 0 combined
2015 August R68 5 humus_mineral Araneae Linyphiidae Maso_sundevalli 1 0.65 yes yes no 0 1 0 0 predator
2015 August R68 5 humus_mineral Coleoptera Staphylinidae Othius_lapidicola 1 0.68 yes yes no 0 1 0 0 predator
2015 August R68 5 humus_mineral Araneae Thomisidae Ozyptila_sp 1 0.00 yes yes no 0 1 0 0 predator
2015 August R68 6 humus_mineral Araneae Linyphiidae Microneta_viaria 1 0.32 yes yes no 0 1 0 0 predator
2015 August R68 6 humus_mineral Myriapoda Lithobiidae Lithobius_curtipes 1 0.87 yes yes no 0 1 0 0 predator
2015 August R68 7 humus Coleoptera Carabidae Amara_brunnea 1 2.68 yes yes yes 1 0 0 0 herbivore
2015 August R68 7 humus Coleoptera Carabidae Amara_brunnea 1 3.07 yes yes yes 1 0 0 0 herbivore
2015 August R68 8 humus Coleoptera Cantharidae . 1 0.08 yes yes no 0 1 0 0 predator
2015 August R68 8 humus_mineral Coleoptera Elateridae Eanus_costalis 1 6.24 yes yes yes 0 0.5 0.5 0 combined
2015 August R68 8 humus_mineral Araneae Linyphiidae Gen_sp 1 0.00 yes yes no 0 1 0 0 predator
2015 August R68 8 humus_mineral Araneae Linyphiidae Porrhomma_pallidum 1 0.00 yes yes no 0 1 0 0 predator
2015 August R68 8 humus_mineral Myriapoda Lithobiidae Lithobius_sp 1 0.27 yes yes no 0 1 0 0 predator
2015 August R68 8 humus_mineral Araneae Theridiidae Robertus_scoticus 1 0.74 yes yes no 0 1 0 0 predator
2015 August R68 8 mineral Coleoptera Elateridae Paraphotistus_impressus 1 0.36 yes yes yes 0.6 0.13 0.13 0.13 combined

2015 August R68 9 humus Coleoptera Cantharidae Malthodes_mysticus 1 0.65 yes yes no 0 1 0 0 predator
2015 August R68 9 humus Coleoptera Carabidae Amara_brunnea 1 1.70 yes yes yes 1 0 0 0 herbivore
2015 August R68 9 humus Coleoptera Carabidae Amara_brunnea 1 2.58 yes yes yes 1 0 0 0 herbivore
2015 August R68 9 humus_mineral Araneae Dictynidae Dictyna_uncinata 1 0.00 yes yes no 0 1 0 0 predator
2015 August R68 9 humus_mineral Araneae Linyphiidae Gen_sp 1 0.81 yes yes no 0 1 0 0 predator
2015 August R68 9 mineral Coleoptera Elateridae Athous_subfuscus 1 2.49 yes yes yes 0 0.8 0.1 0.1 combined
2015 August R68 9 mineral Coleoptera Elateridae Paraphotistus_impressus 1 4.25 yes yes yes 0.6 0.13 0.13 0.13 combined
2015 August R68 10 humus_mineral Myriapoda Lithobiidae Lithobius_curtipes 1 1.30 yes yes no 0 1 0 0 predator
2015 August R69 1 humus Annelida Lumbricidae Dendrobaena_octaedra 1 3.59 yes yes no 0 0 1 0 detritivore
2015 August R69 1 humus Coleoptera Elateridae Eanus_costalis 1 3.24 yes yes no 0 0.5 0.5 0 combined
2015 August R69 1 humus Diptera Empididae Phyllodromia_melanocephala 1 0.12 yes yes no 0 1 0 0 predator
2015 August R69 1 humus Diptera Empididae Phyllodromia_melanocephala 1 1.08 yes yes no 0 1 0 0 predator
2015 August R69 1 humus Coleoptera Staphylinidae . 1 0.08 yes yes no 0 1 0 0 predator
2015 August R69 1 humus Coleoptera Staphylinidae . 1 0.45 yes yes no 0 1 0 0 predator
2015 August R69 1 humus Coleoptera Staphylinidae Othius_angustus 1 0.21 yes yes no 0 1 0 0 predator
2015 August R69 1 humus Coleoptera Staphylinidae Othius_lapidicola 1 0.44 yes yes no 0 1 0 0 predator
2015 August R69 1 humus Coleoptera Staphylinidae Othius_lapidicola 1 0.68 yes yes no 0 1 0 0 predator
2015 August R69 1 humus Coleoptera Staphylinidae Oxypoda_annularis 1 0.08 yes yes no 0 1 0 0 predator
2015 August R69 1 humus_mineral Coleoptera Staphylinidae Othius_lapidicola 1 0.30 yes yes no 0 1 0 0 predator
2015 August R69 1 humus_mineral Coleoptera Staphylinidae Othius_lapidicola 1 0.54 yes yes no 0 1 0 0 predator
2015 August R69 1 humus_mineral Coleoptera Staphylinidae Othius_lapidicola 1 0.61 yes yes no 0 1 0 0 predator
2015 August R69 1 humus_mineral Coleoptera Staphylinidae Quedius_fulvicollis 1 0.79 yes yes no 0 1 0 0 predator
2015 August R69 1 humus_mineral Coleoptera Staphylinidae Quedius_sp 1 1.00 yes yes no 0 1 0 0 predator
2015 August R69 2 humus Coleoptera Elateridae Eanus_costalis 1 2.68 yes yes no 0 0.5 0.5 0 combined
2015 August R69 2 humus Coleoptera . . 1 0.52 . . . .
2015 August R69 2 humus_mineral Coleoptera Carabidae Notiophilus_biguttatus 1 2.05 yes yes no 0 1 0 0 predator
2015 August R69 2 humus_mineral Myriapoda Lithobiidae Lithobius_sp 1 0.33 yes yes no 0 1 0 0 predator
2015 August R69 2 humus_mineral Coleoptera Staphylinidae Othius_subuliformis 1 0.50 yes yes no 0 1 0 0 predator
2015 August R69 3 humus Diptera Lauxaniidae Homoneura_sp 1 1.18 yes yes no 0 0 1 0 detritivore
2015 August R69 3 humus Diptera Lauxaniidae Homoneura_sp 1 1.24 yes yes no 0 0 1 0 detritivore
2015 August R69 3 humus Diptera Lauxaniidae Homoneura_sp 1 1.68 yes yes no 0 0 1 0 detritivore
2015 August R69 3 humus Coleoptera Elateridae Eanus_costalis 4 10.94 yes yes no 0 0.5 0.5 0 combined
2015 August R69 3 humus Coleoptera Cantharidae . 1 0.13 yes yes no 0 1 0 0 predator
2015 August R69 3 humus Coleoptera Cantharidae . 1 0.25 yes yes no 0 1 0 0 predator
2015 August R69 3 humus Coleoptera Cantharidae Malthodes_fuscus 1 0.27 yes yes no 0 1 0 0 predator
2015 August R69 3 humus Coleoptera Carabidae Calathus_melanocephalus 1 1.12 yes yes no 0 1 0 0 predator
2015 August R69 3 humus Coleoptera . . 1 0.15 . . . .
2015 August R69 3 humus_mineral Coleoptera Carabidae Notiophilus_biguttatus 1 2.09 yes yes no 0 1 0 0 predator
2015 August R69 3 humus_mineral Araneae Linyphiidae Gen_sp 2 0.00 yes yes no 0 1 0 0 predator
2015 August R69 3 humus_mineral Araneae Linyphiidae Tapinocyba_insecta 1 0.00 yes yes no 0 1 0 0 predator
2015 August R69 3 humus_mineral Coleoptera Staphylinidae Othius_lapidicola 1 0.87 yes yes no 0 1 0 0 predator
2015 August R69 3 humus_mineral Coleoptera Staphylinidae Oxypoda_annularis 1 0.07 yes yes no 0 1 0 0 predator
2015 August R69 3 humus_mineral Coleoptera Staphylinidae Oxypoda_annularis 1 0.08 yes yes no 0 1 0 0 predator
2015 August R69 3 humus_mineral Coleoptera Staphylinidae Oxypoda_annularis 1 0.10 yes yes no 0 1 0 0 predator
2015 August R69 3 humus_mineral Coleoptera Staphylinidae Oxypoda_annularis 1 0.10 yes yes no 0 1 0 0 predator
2015 August R69 3 humus_mineral Coleoptera Staphylinidae Oxypoda_annularis 1 0.10 yes yes no 0 1 0 0 predator
2015 August R69 3 humus_mineral Coleoptera Staphylinidae Oxypoda_annularis 1 0.10 yes yes no 0 1 0 0 predator
2015 August R69 3 humus_mineral Coleoptera Staphylinidae Oxypoda_annularis 1 0.11 yes yes no 0 1 0 0 predator
2015 August R69 3 humus_mineral Coleoptera Staphylinidae Oxypoda_annularis 1 0.11 yes yes no 0 1 0 0 predator
2015 August R69 3 humus_mineral Coleoptera Staphylinidae Oxypoda_annularis 1 0.12 yes yes no 0 1 0 0 predator
2015 August R69 3 humus_mineral Araneae Theridiidae Robertus_scoticus 1 1.05 yes yes no 0 1 0 0 predator
2015 August R69 3 humus_mineral Araneae Theridiidae Robertus_sp 1 0.00 yes yes no 0 1 0 0 predator
2015 August R69 4 humus Coleoptera Elateridae Eanus_costalis 5 8.07 yes yes no 0 0.5 0.5 0 combined

2015 August R69 4 humus Coleoptera Cantharidae Podistra_schoenherri 1 2.47 yes yes no 0 1 0 0 predator
2015 August R69 4 humus Coleoptera Cantharidae Podistra_schoenherri 1 5.96 yes yes no 0 1 0 0 predator
2015 August R69 4 humus Coleoptera Cantharidae Podistra_schoenherri 1 6.38 yes yes no 0 1 0 0 predator
2015 August R69 4 humus Coleoptera Elateridae . 1 15.49 yes yes . . . . . .
2015 August R69 4 humus_mineral Araneae Linyphiidae Gen_sp 2 0.00 yes yes no 0 1 0 0 predator
2015 August R69 4 humus_mineral Coleoptera Staphylinidae Liogluta_micans 1 0.46 yes yes no 0 1 0 0 predator
2015 August R69 4 humus_mineral Coleoptera Staphylinidae Liogluta_micans 1 0.48 yes yes no 0 1 0 0 predator
2015 August R69 4 humus_mineral Coleoptera Staphylinidae Oxypoda_annularis 1 0.10 yes yes no 0 1 0 0 predator
2015 August R69 4 humus_mineral Araneae Theridiidae Robertus_scoticus 1 0.00 yes yes no 0 1 0 0 predator
2015 August R69 4 humus_mineral Araneae Theridiidae Robertus_scoticus 1 1.18 yes yes no 0 1 0 0 predator
2015 August R69 4 mineral Coleoptera Elateridae . 1 1.35 yes yes . . . . . .
2015 August R69 5 humus Annelida Lumbricidae Eiseniella_tetraedra 1 0.17 yes yes no 0 0 1 0 detritivore
2015 August R69 5 humus_mineral Araneae Linyphiidae Zornella_cultrigera 1 1.31 yes yes no 0 1 0 0 predator
2015 August R69 5 humus_mineral Coleoptera Staphylinidae Liogluta_micans 1 0.34 yes yes no 0 1 0 0 predator
2015 August R69 5 humus_mineral Coleoptera Staphylinidae Liogluta_micans 1 0.67 yes yes no 0 1 0 0 predator
2015 August R69 5 humus_mineral Coleoptera Staphylinidae Othius_lapidicola 1 0.93 yes yes no 0 1 0 0 predator
2015 August R69 6 humus Coleoptera Cantharidae Podistra_schoenherri 1 0.90 yes yes no 0 1 0 0 predator
2015 August R69 6 humus Diptera Empididae Phyllodromia_melanocephala 1 0.52 yes yes no 0 1 0 0 predator
2015 August R69 6 humus Coleoptera Staphylinidae Othius_lapidicola 1 0.12 yes yes no 0 1 0 0 predator
2015 August R69 6 humus Coleoptera Staphylinidae Othius_lapidicola 1 0.38 yes yes no 0 1 0 0 predator
2015 August R69 6 humus Coleoptera . . 1 1.19 . . . .
2015 August R69 6 humus_mineral Coleoptera Carabidae Notiophilus_biguttatus 1 2.08 yes yes no 0 1 0 0 predator
2015 August R69 6 humus_mineral Araneae Linyphiidae Gen_sp 1 0.00 yes yes no 0 1 0 0 predator
2015 August R69 6 humus_mineral Araneae Linyphiidae Micrargus_herbigradus 1 0.00 yes yes no 0 1 0 0 predator
2015 August R69 6 humus_mineral Araneae Linyphiidae Tapinocyba_pallens 1 0.00 yes yes no 0 1 0 0 predator
2015 August R69 6 humus_mineral Coleoptera Staphylinidae Atheta_subtilis 1 0.06 yes yes no 0 1 0 0 predator
2015 August R69 6 humus_mineral Coleoptera Staphylinidae Othius_subuliformis 1 0.72 yes yes no 0 1 0 0 predator
2015 August R69 7 humus Coleoptera Carabidae Calathus_melanocephalus 1 0.34 yes yes no 0 1 0 0 predator
2015 August R69 7 humus Coleoptera Staphylinidae Oxypoda_annularis 1 0.06 yes yes no 0 1 0 0 predator
2015 August R69 7 humus Coleoptera Staphylinidae Oxypoda_annularis 1 0.06 yes yes no 0 1 0 0 predator
2015 August R69 7 humus_mineral Araneae Linyphiidae Microneta_viaria 1 0.30 yes yes no 0 1 0 0 predator
2015 August R69 7 humus_mineral Myriapoda Lithobiidae Lithobius_sp 1 0.56 yes yes no 0 1 0 0 predator
2015 August R69 7 humus_mineral Coleoptera Staphylinidae Liogluta_micans 1 0.42 yes yes no 0 1 0 0 predator
2015 August R69 7 humus_mineral Coleoptera Staphylinidae Oxypoda_annularis 1 0.11 yes yes no 0 1 0 0 predator
2015 August R69 8 humus Coleoptera Elateridae Eanus_costalis 2 5.48 yes yes no 0 0.5 0.5 0 combined
2015 August R69 8 humus Coleoptera Elateridae Liotrichus_affinis 1 2.60 yes yes no 0 0.5 0.5 0 combined
2015 August R69 8 humus Coleoptera Cantharidae Malthodes_fuscus 1 0.65 yes yes no 0 1 0 0 predator
2015 August R69 8 humus_mineral Coleoptera Carabidae Notiophilus_biguttatus 1 2.10 yes yes no 0 1 0 0 predator
2015 August R69 8 humus_mineral Coleoptera Carabidae Notiophilus_germinyi 1 0.72 yes yes no 0 1 0 0 predator
2015 August R69 8 humus_mineral Araneae Linyphiidae Tapinocyba_pallens 1 0.16 yes yes no 0 1 0 0 predator
2015 August R69 8 humus_mineral Coleoptera Staphylinidae Oxypoda_annularis 1 0.09 yes yes no 0 1 0 0 predator
2015 August R69 9 humus Annelida Lumbricidae Dendrobaena_octaedra 1 22.20 yes yes no 0 0 1 0 detritivore
2015 August R69 9 humus Coleoptera Elateridae Eanus_costalis 1 3.07 yes yes no 0 0.5 0.5 0 combined
2015 August R69 9 humus Coleoptera Cantharidae Malthodes_fuscus 1 0.06 yes yes no 0 1 0 0 predator
2015 August R69 9 humus Coleoptera Cantharidae Podistra_schoenherri 1 4.59 yes yes no 0 1 0 0 predator
2015 August R69 9 humus Diptera Empididae Phyllodromia_melanocephala 1 1.55 yes yes no 0 1 0 0 predator
2015 August R69 9 humus Coleoptera Staphylinidae Oxypoda_annularis 1 0.13 yes yes no 0 1 0 0 predator
2015 August R69 9 humus Coleoptera . . 1 0.23 . . . .
2015 August R69 9 humus_mineral Araneae Linyphiidae Gen_sp 1 0.00 yes yes no 0 1 0 0 predator
2015 August R69 9 humus_mineral Coleoptera Staphylinidae Bolitochara_pulchra 1 0.42 yes yes no 0 1 0 0 predator
2015 August R69 9 humus_mineral Coleoptera Staphylinidae Oxypoda_annularis 1 0.08 yes yes no 0 1 0 0 predator
2015 August R69 9 humus_mineral Coleoptera Staphylinidae Oxypoda_annularis 1 0.09 yes yes no 0 1 0 0 predator
2015 August R69 9 humus_mineral Coleoptera Staphylinidae Oxypoda_annularis 1 0.10 yes yes no 0 1 0 0 predator

2015 August R69 9 humus_mineral Araneae Theridiidae Robertus_scoticus 1 0.48 yes yes no 0 1 0 0 predator
2015 August R69 10 humus Coleoptera Elateridae Eanus_costalis 5 18.23 yes yes no 0 0.5 0.5 0 combined
2015 August R69 10 humus Hemiptera Lygaeidae Eremocoris_abietis 1 1.77 yes no no 1 0 0 0 herbivore
2015 August R69 10 humus Coleoptera . . 1 0.06 . . . .
2015 August R69 10 humus Coleoptera . . 1 0.09 . . . .
2015 August R69 10 humus Coleoptera Polyphaga . 1 0.15 . . . .
2015 August R69 10 humus_mineral Araneae Linyphiidae Semljicola_faustus 1 0.00 yes yes no 0 1 0 0 predator
2015 August R69 10 humus_mineral Araneae Linyphiidae Tapinocyba_pallens 1 0.00 yes yes no 0 1 0 0 predator
2015 August R69 10 humus_mineral Coleoptera Staphylinidae Oxypoda_annularis 1 0.09 yes yes no 0 1 0 0 predator
2015 August R69 10 humus_mineral Coleoptera Staphylinidae Quedius_sp 1 1.10 yes yes no 0 1 0 0 predator
2015 August R69 10 humus_mineral Coleoptera Staphylinidae Quedius_sp 1 1.11 yes yes no 0 1 0 0 predator
2015 August R69 10 humus_mineral Araneae Theridiidae Robertus_sp 1 0.45 yes yes no 0 1 0 0 predator
2016 June R60 1 humus Myriapoda Polyzonidae Polyzonium_germanicum 1 3.33 yes yes no 0 0 1 0 detritivore
2016 June R60 1 humus Coleoptera Elateridae Ampedus_nigrinus 1 3.35 yes yes no 0 0.5 0.5 0 combined
2016 June R60 1 humus Coleoptera Elateridae Athous_subfuscus 1 9.71 yes yes yes 0 0.8 0.1 0.1 combined
2016 June R60 1 humus Araneae Linyphiidae Palliduphantes_pallidus 1 0.39 yes yes no 0 1 0 0 predator
2016 June R60 1 humus Myriapoda Lithobiidae Lithobius_curtipes 1 1.10 yes yes no 0 1 0 0 predator
2016 June R60 1 humus Diptera Rhagionidae Rhagio_lineola 1 3.59 yes yes no 0 1 0 0 predator
2016 June R60 2 humus Coleoptera Elateridae Athous_subfuscus 1 4.76 yes yes yes 0 0.8 0.1 0.1 combined
2016 June R60 2 humus Coleoptera Carabidae Trechus_secalis 1 0.38 yes yes no 0 1 0 0 predator
2016 June R60 2 humus Myriapoda Lithobiidae Lithobius_curtipes 1 1.39 yes yes no 0 1 0 0 predator
2016 June R60 2 humus Diptera Rhagionidae Rhagio_lineola 1 1.40 yes yes no 0 1 0 0 predator
2016 June R60 2 humus Coleoptera Curculionidae Sciaphilus_asperatus 1 3.77 yes yes yes 1 0 0 0 herbivore
2016 June R60 2 humus Lepidoptera Hepialidae Korscheltellus_fusconebulosa 1 10.84 Yes Yes yes 1 0 0 0 herbivore
2016 June R60 2 humus Lepidoptera Hepialidae Korscheltellus_fusconebulosa 1 6.41 yes yes yes 1 0 0 0 herbivore
2016 June R60 2 humus Lepidoptera Hepialidae Korscheltellus_fusconebulosa 1 8.05 Yes Yes yes 1 0 0 0 herbivore
2016 June R60 3 humus Coleoptera Elateridae Athous_subfuscus 1 2.99 yes yes yes 0 0.8 0.1 0.1 combined
2016 June R60 3 humus Coleoptera Elateridae Athous_subfuscus 1 6.38 yes yes yes 0 0.8 0.1 0.1 combined
2016 June R60 3 humus Coleoptera Elateridae Dalopius_marginatus 1 4.98 yes yes yes 0 0.8 0.1 0.1 combined
2016 June R60 3 humus Coleoptera Carabidae Trechus_secalis 1 0.72 yes yes no 0 1 0 0 predator
2016 June R60 3 humus Araneae Hahniidae Hahnia_pusilla 2 0.47 yes yes no 0 1 0 0 predator
2016 June R60 3 humus Araneae Hahniidae Hahnia_sp 1 0.13 yes yes no 0 1 0 0 predator
2016 June R60 3 humus Araneae Linyphiidae Tapinocyba_pallens 1 0.13 yes yes no 0 1 0 0 predator
2016 June R60 3 humus Coleoptera Staphylinidae Atheta_graminicola 1 0.37 yes yes no 0 1 0 0 predator
2016 June R60 3 humus Lepidoptera Hepialidae Phymatopus_hecta 1 10.32 Yes Yes yes 1 0 0 0 herbivore
2016 June R60 3 humus Lepidoptera Hepialidae Phymatopus_hecta 1 27.45 Yes Yes yes 1 0 0 0 herbivore
2016 June R60 3 humus Lepidoptera Hepialidae Phymatopus_hecta 1 64.05 Yes Yes yes 1 0 0 0 herbivore
2016 June R60 4 humus Annelida Lumbricidae Dendrodrilus_rubidus_tenuis 1 13.92 yes yes no 0 0 1 0 detritivore
2016 June R60 4 humus Coleoptera Elateridae Athous_subfuscus 1 3.73 yes yes yes 0 0.8 0.1 0.1 combined
2016 June R60 4 humus Araneae Lycosidae Gen_sp 1 0.46 yes yes no 0 1 0 0 predator
2016 June R60 4 humus Diptera Rhagionidae Rhagio_lineola 1 0.74 yes yes no 0 1 0 0 predator
2016 June R60 4 humus Coleoptera Staphylinidae Lathrobium_brunnipes 1 0.21 yes yes no 0 1 0 0 predator
2016 June R60 4 humus Lepidoptera Hepialidae Phymatopus_hecta 1 10.63 Yes Yes yes 1 0 0 0 herbivore
2016 June R60 5 humus Annelida Lumbricidae Dendrodrilus_rubidus_tenuis 3 37.15 yes yes no 0 0 1 0 detritivore
2016 June R60 5 humus Myriapoda Lithobiidae Lithobius_curtipes 1 0.77 yes yes no 0 1 0 0 predator
2016 June R60 5 humus Diptera Rhagionidae Rhagio_lineola 1 0.66 yes yes no 0 1 0 0 predator
2016 June R60 6 humus Annelida Lumbricidae Dendrodrilus_rubidus_tenuis 2 31.44 yes yes no 0 0 1 0 detritivore
2016 June R60 6 humus Coleoptera Elateridae Athous_subfuscus 1 1.39 yes yes yes 0 0.8 0.1 0.1 combined
2016 June R60 6 humus Araneae Linyphiidae Agyneta_subtilis 1 0.45 yes yes no 0 1 0 0 predator
2016 June R60 6 humus Araneae Linyphiidae Gen_sp 4 0.42 yes yes no 0 1 0 0 predator
2016 June R60 6 humus Araneae Linyphiidae Maro_minutus 1 0.13 yes yes no 0 1 0 0 predator
2016 June R60 6 humus Araneae Linyphiidae Tapinocyba_pallens 1 0.24 yes yes no 0 1 0 0 predator
2016 June R60 6 humus Myriapoda Lithobiidae Lithobius_curtipes 1 0.54 yes yes no 0 1 0 0 predator

2016 June R60 6 humus Myriapoda Lithobiidae Lithobius_curtipes 1 1.50 yes yes no 0 1 0 0 predator
2016 June R60 6 humus Hemiptera Loriculidae Loricula_exilis 1 0.05 yes yes no 0 1 0 0 predator
2016 June R60 7 humus Coleoptera Cryptophagidae Cryptophagus_setulosus 1 0.13 yes yes no 0 0 0.5 0.5 combined
2016 June R60 7 humus Araneae Linyphiidae Tapinocyba_pallens 1 1.17 yes yes no 0 1 0 0 predator
2016 June R60 7 humus Myriapoda Lithobiidae Lithobius_curtipes 1 0.81 yes yes no 0 1 0 0 predator
2016 June R60 7 humus Myriapoda Lithobiidae Lithobius_curtipes 1 1.83 yes yes no 0 1 0 0 predator
2016 June R60 7 humus Myriapoda Lithobiidae Lithobius_curtipes 1 2.17 yes yes no 0 1 0 0 predator
2016 June R60 7 humus Araneae Thomisidae Ozyptila_sp 1 0.14 yes yes no 0 1 0 0 predator
2016 June R60 7 mineral Coleoptera Carabidae Leistus_terminatus 1 2.72 yes yes no 0 1 0 0 predator
2016 June R60 8 humus Coleoptera Elateridae Ampedus_nigrinus 1 2.44 yes yes no 0 0.5 0.5 0 combined
2016 June R60 8 humus Coleoptera Elateridae Dalopius_marginatus 1 1.72 yes yes yes 0 0.8 0.1 0.1 combined
2016 June R60 8 mineral Coleoptera Carabidae Leistus_terminatus 1 2.72 yes yes no 0 1 0 0 predator
2016 June R60 9 humus Coleoptera Elateridae Athous_subfuscus 1 1.61 yes yes yes 0 0.8 0.1 0.1 combined
2016 June R60 9 humus Coleoptera Elateridae Athous_subfuscus 1 3.71 yes yes yes 0 0.8 0.1 0.1 combined
2016 June R60 9 humus Hymenoptera Formicidae Myrmica_ruginodis 3 2.01 Yes Yes no 0 1 0 0 predator
2016 June R60 9 humus Araneae Hahniidae Hahnia_sp 1 0.25 yes yes no 0 1 0 0 predator
2016 June R60 9 humus Araneae Linyphiidae Minyriolus_pusillus 1 1.47 yes yes no 0 1 0 0 predator
2016 June R60 9 humus Myriapoda Lithobiidae Lithobius_curtipes 1 2.14 yes yes no 0 1 0 0 predator
2016 June R60 9 humus Myriapoda Lithobiidae Lithobius_sp 1 0.49 yes yes no 0 1 0 0 predator
2016 June R60 9 humus Hemiptera Loriculidae Loricula_exilis 1 0.07 yes yes no 0 1 0 0 predator
2016 June R60 9 humus Hemiptera Loriculidae Loricula_exilis 1 0.07 yes yes no 0 1 0 0 predator
2016 June R60 9 humus Araneae Lycosidae Pirata_Piratula_sp 2 0.23 yes yes no 0 1 0 0 predator
2016 June R60 9 humus Diptera Rhagionidae Rhagio_sp 1 0.87 yes yes no 0 1 0 0 predator
2016 June R60 9 humus Coleoptera Staphylinidae Drusilla_canaliculata 1 0.67 yes yes no 0 1 0 0 predator
2016 June R60 9 humus Coleoptera Staphylinidae Geostiba_circellaris 1 0.09 yes yes no 0 1 0 0 predator
2016 June R60 9 humus Coleoptera Staphylinidae Pselaphus_heisei 1 0.08 yes yes no 0 1 0 0 predator
2016 June R60 9 humus Coleoptera Staphylinidae Xantholinus_tricolor 1 0.10 yes yes no 0 1 0 0 predator
2016 June R60 9 humus Araneae Theridiidae Robertus_scoticus 1 1.05 yes yes no 0 1 0 0 predator
2016 June R60 9 humus Lepidoptera Hepialidae Phymatopus_hecta 1 19.97 Yes Yes yes 1 0 0 0 herbivore
2016 June R60 9 humus Coleoptera . . 1 0.24 . . . .
2016 June R60 10 humus Coleoptera Elateridae Dalopius_marginatus 1 5.44 yes yes yes 0 0.8 0.1 0.1 combined
2016 June R60 10 humus Araneae Hahniidae Gen_sp 1 0.13 yes yes no 0 1 0 0 predator
2016 June R60 10 humus Araneae Linyphiidae Tapinocyba_pallens 1 0.25 yes yes no 0 1 0 0 predator
2016 June R60 10 humus Myriapoda Lithobiidae Lithobius_curtipes 1 0.43 yes yes no 0 1 0 0 predator
2016 June R60 10 humus Diptera Rhagionidae Rhagio_lineola 1 0.48 yes yes no 0 1 0 0 predator
2016 June R60 10 humus Coleoptera Staphylinidae . 1 2.88 yes yes no 0 1 0 0 predator
2016 June R60 10 humus Coleoptera Elateridae . 1 1.14 yes yes . . . . . .
2016 June R61 1 humus Araneae Thomisidae Ozyptila_sp 2 5.49 yes yes no 0 1 0 0 predator
2016 June R61 1 humus Lepidoptera Hepialidae Phymatopus_hecta 1 18.67 Yes Yes yes 1 0 0 0 herbivore
2016 June R61 1 mineral Araneae Araneidae Araneus_alsine 1 8.58 no no no 0 1 0 0 predator
2016 June R61 2 humus Coleoptera Staphylinidae Stenus_clavicornis 1 0.85 yes yes no 0 1 0 0 predator
2016 June R61 2 mineral Araneae Araneidae Araneus_alsine 1 8.58 no no no 0 1 0 0 predator
2016 June R61 3 humus Annelida Lumbricidae Eiseniella_tetraedra 1 0.21 yes yes no 0 0 1 0 detritivore
2016 June R61 3 humus Myriapoda Lithobiidae Lithobius_curtipes 1 0.74 yes yes no 0 1 0 0 predator
2016 June R61 3 humus Coleoptera Staphylinidae Atheta_myrmecobia 1 0.06 yes yes no 0 1 0 0 predator
2016 June R61 3 humus Coleoptera Staphylinidae Atheta_myrmecobia 1 0.06 yes yes no 0 1 0 0 predator
2016 June R61 3 humus Coleoptera Staphylinidae Atheta_myrmecobia 1 0.10 yes yes no 0 1 0 0 predator
2016 June R61 4 humus Hymenoptera Formicidae Myrmica_rubra 1 0.39 Yes Yes no 0 1 0 0 predator
2016 June R61 4 humus Myriapoda Lithobiidae Lithobius_curtipes 1 1.04 yes yes no 0 1 0 0 predator
2016 June R61 4 humus Myriapoda Lithobiidae Lithobius_curtipes 1 1.43 yes yes no 0 1 0 0 predator
2016 June R61 4 humus Coleoptera . . 1 3.93 . . . .
2016 June R61 7 humus Coleoptera Elateridae Ampedus_cf_balteatus 1 8.66 yes yes no 0 0.5 0.5 0 combined
2016 June R61 7 humus Araneae Lycosidae Gen_sp 1 0.67 yes yes no 0 1 0 0 predator

2016 June R61 7 humus Hemiptera Lygaeidae Eremocoris_sp 1 0.71 yes no no 1 0 0 0 herbivore
2016 June R61 7 humus Hemiptera Lygaeidae Eremocoris_sp 1 0.94 yes no no 1 0 0 0 herbivore
2016 June R61 9 humus Araneae Hahniidae Hahnia_pusilla 2 0.00 yes yes no 0 1 0 0 predator
2016 June R61 9 humus Araneae Hahniidae Hahnia_sp 1 1.31 yes yes no 0 1 0 0 predator
2016 June R61 9 humus Araneae Linyphiidae Gen_sp 1 0.17 yes yes no 0 1 0 0 predator
2016 June R61 9 humus Myriapoda Lithobiidae Lithobius_curtipes 1 0.39 yes yes no 0 1 0 0 predator
2016 June R61 9 humus Coleoptera Staphylinidae Ochthephilum_fracticorne 1 0.11 yes yes no 0 1 0 0 predator
2016 June R61 9 humus Coleoptera Staphylinidae Ochthephilum_fracticorne 1 0.22 yes yes no 0 1 0 0 predator
2016 June R61 9 humus Hemiptera Tingidae Acalypta_parvula 1 0.14 yes yes no 1 0 0 0 herbivore
2016 June R61 9 mineral Coleoptera Elateridae Paraphotistus_impressus 1 0.84 yes yes yes 0.6 0.13 0.13 0.13 combined
2016 June R61 9 mineral Coleoptera Curculionidae Otiorhynchus_scaber 1 7.27 yes yes yes 1 0 0 0 herbivore
2016 June R61 10 humus Coleoptera Cantharidae . 1 0.95 yes yes no 0 1 0 0 predator
2016 June R61 10 humus Lepidoptera Oecophoridae Pleurota_bicostella 1 2.60 Yes no no 1 0 0 0 herbivore
2016 June R61 10 mineral Coleoptera Elateridae Paraphotistus_impressus 1 0.84 yes yes yes 0.6 0.13 0.13 0.13 combined
2016 June R61 10 mineral Coleoptera Curculionidae Otiorhynchus_scaber 1 7.27 yes yes yes 1 0 0 0 herbivore
2016 June R62 1 humus Coleoptera Staphylinidae Sepedophilus_pedicularius 1 0.16 yes yes no 0 0 0 1 fungivore
2016 June R62 1 humus Coleoptera Elateridae Athous_subfuscus 1 1.26 yes yes yes 0 0.8 0.1 0.1 combined
2016 June R62 1 humus Coleoptera Elateridae Athous_subfuscus 1 1.48 yes yes yes 0 0.8 0.1 0.1 combined
2016 June R62 1 humus Coleoptera Elateridae Athous_subfuscus 1 3.94 yes yes yes 0 0.8 0.1 0.1 combined
2016 June R62 1 humus Myriapoda Lithobiidae Lithobius_curtipes 1 0.97 yes yes no 0 1 0 0 predator
2016 June R62 1 humus Coleoptera Staphylinidae Stenus_clavicornis 1 0.70 yes yes no 0 1 0 0 predator
2016 June R62 1 humus Araneae Thomisidae Ozyptila_sp 1 0.82 yes yes no 0 1 0 0 predator
2016 June R62 1 humus Coleoptera . . 1 0.61 . . . .
2016 June R62 1 mineral Myriapoda Julidae Ommatoiulus_sabulosus 1 14.47 yes yes no 0 0 1 0 detritivore
2016 June R62 2 humus Coleoptera Leiodidae Agathidium_nigripenne 1 0.84 yes yes no 0 0 0 1 fungivore
2016 June R62 2 humus Myriapoda Julidae Ommatoiulus_sabulosus 1 32.42 yes yes no 0 0 1 0 detritivore
2016 June R62 2 humus Coleoptera Cantharidae . 1 11.17 yes yes no 0 1 0 0 predator
2016 June R62 2 humus Araneae Linyphiidae Gen_sp 4 0.92 yes yes no 0 1 0 0 predator
2016 June R62 2 humus Araneae Liocranidae Gen_sp 1 1.04 yes yes no 0 1 0 0 predator
2016 June R62 2 humus Myriapoda Lithobiidae Lithobius_curtipes 1 0.31 yes yes no 0 1 0 0 predator
2016 June R62 2 humus Myriapoda Lithobiidae Lithobius_sp 1 0.31 yes yes no 0 1 0 0 predator
2016 June R62 2 humus Myriapoda Lithobiidae Lithobius_sp 1 0.42 yes yes no 0 1 0 0 predator
2016 June R62 2 humus Diptera Rhagionidae Rhagio_scolopaceus 1 1.22 yes yes no 0 1 0 0 predator
2016 June R62 2 humus Diptera Rhagionidae Rhagio_scolopaceus 1 4.09 yes yes no 0 1 0 0 predator
2016 June R62 2 humus Coleoptera Staphylinidae Atheta_boleticola 1 0.16 yes yes no 0 1 0 0 predator
2016 June R62 2 mineral Myriapoda Julidae Ommatoiulus_sabulosus 1 14.47 yes yes no 0 0 1 0 detritivore
2016 June R62 3 humus Annelida Lumbricidae . 1 0.21 yes yes no 0 0 1 0 detritivore
2016 June R62 3 humus Coleoptera Elateridae Dalopius_marginatus 1 0.73 yes yes yes 0 0.8 0.1 0.1 combined
2016 June R62 3 humus Coleoptera Elateridae Dalopius_marginatus 1 1.45 yes yes yes 0 0.8 0.1 0.1 combined
2016 June R62 3 humus Coleoptera Cantharidae Malthodes_mysticus 1 0.07 yes yes no 0 1 0 0 predator
2016 June R62 3 humus Hymenoptera Formicidae Myrmica_ruginodis 2 0.66 Yes Yes no 0 1 0 0 predator
2016 June R62 3 humus Araneae Linyphiidae Gen_sp 1 0.33 yes yes no 0 1 0 0 predator
2016 June R62 3 humus Coleoptera Staphylinidae Stenus_clavicornis 1 0.54 yes yes no 0 1 0 0 predator
2016 June R62 3 humus Lepidoptera Tortricidae Acleris_laterana 1 1.85 no no no 1 0 0 0 herbivore
2016 June R62 4 humus Coleoptera Elateridae Dalopius_marginatus 1 2.52 yes yes yes 0 0.8 0.1 0.1 combined
2016 June R62 4 humus Araneae Linyphiidae Gen_sp 3 1.11 yes yes no 0 1 0 0 predator
2016 June R62 4 humus Myriapoda Lithobiidae Lithobius_curtipes 1 0.24 yes yes no 0 1 0 0 predator
2016 June R62 4 humus Myriapoda Lithobiidae Lithobius_curtipes 1 0.85 yes yes no 0 1 0 0 predator
2016 June R62 4 humus Myriapoda Lithobiidae Lithobius_curtipes 1 1.95 yes yes no 0 1 0 0 predator
2016 June R62 4 humus Coleoptera Staphylinidae Ischnosoma_sp 1 0.27 yes yes no 0 1 0 0 predator
2016 June R62 5 humus Coleoptera Elateridae Athous_subfuscus 1 1.04 yes yes yes 0 0.8 0.1 0.1 combined
2016 June R62 5 humus Coleoptera Elateridae Athous_subfuscus 1 2.05 yes yes yes 0 0.8 0.1 0.1 combined
2016 June R62 5 humus Hymenoptera Formicidae Myrmica_ruginodis 1 0.48 Yes Yes no 0 1 0 0 predator

2016 June R62 5 humus Myriapoda Lithobiidae Lithobius_curtipes 1 0.67 yes yes no 0 1 0 0 predator
2016 June R62 5 humus Diptera Rhagionidae Rhagio_lineola 1 2.49 yes yes no 0 1 0 0 predator
2016 June R62 6 humus Coleoptera Cantharidae . 1 2.76 yes yes no 0 1 0 0 predator
2016 June R62 6 humus Araneae Linyphiidae Asthenargus_paganus 1 0.25 yes yes no 0 1 0 0 predator
2016 June R62 6 humus Araneae Linyphiidae Centromerus_arcanus 1 0.25 yes yes no 0 1 0 0 predator
2016 June R62 6 humus Araneae Linyphiidae Diplocephalus_latifrons 1 0.25 yes yes no 0 1 0 0 predator
2016 June R62 6 humus Araneae Linyphiidae Gen_sp 2 0.25 yes yes no 0 1 0 0 predator
2016 June R62 6 humus Araneae Linyphiidae Micrargus_herbigradus 1 0.25 yes yes no 0 1 0 0 predator
2016 June R62 6 humus Araneae Linyphiidae Tapinocyba_pallens 1 0.29 yes yes no 0 1 0 0 predator
2016 June R62 6 humus Diptera Rhagionidae Rhagio_tringarius 1 8.49 yes yes no 0 1 0 0 predator
2016 June R62 7 humus Coleoptera Elateridae Dalopius_marginatus 1 0.36 yes yes yes 0 0.8 0.1 0.1 combined
2016 June R62 7 humus Araneae Linyphiidae Gen_sp 1 0.13 yes yes no 0 1 0 0 predator
2016 June R62 7 humus Myriapoda Lithobiidae Lithobius_sp 1 0.08 yes yes no 0 1 0 0 predator
2016 June R62 7 humus Coleoptera Staphylinidae Stenus_clavicornis 1 0.75 yes yes no 0 1 0 0 predator
2016 June R62 7 humus Araneae Theridiidae Robertus_lividus 1 1.78 yes yes no 0 1 0 0 predator
2016 June R62 7 humus Diptera Chironomidae Metriocnemus_sp 1 0.06 yes yes yes 0.25 0 0.75 0 combined
2016 June R62 7 humus Lepidoptera Hepialidae Phymatopus_hecta 1 2.50 Yes Yes yes 1 0 0 0 herbivore
2016 June R62 7 humus Coleoptera . . 1 0.23 . . . .
2016 June R62 8 humus Annelida Lumbricidae Dendrobaena_octaedra 1 3.74 yes yes no 0 0 1 0 detritivore
2016 June R62 8 humus Diptera Tipulidae Tipula_selene 1 39.87 yes yes no 0 0 1 0 detritivore
2016 June R62 8 humus Coleoptera Elateridae Athous_subfuscus 1 2.02 yes yes yes 0 0.8 0.1 0.1 combined
2016 June R62 8 humus Coleoptera Elateridae Dalopius_marginatus 1 2.23 yes yes yes 0 0.8 0.1 0.1 combined
2016 June R62 8 humus Araneae Linyphiidae Centromerus_arcanus 2 0.45 yes yes no 0 1 0 0 predator
2016 June R62 8 humus Araneae Linyphiidae Gen_sp 2 0.34 yes yes no 0 1 0 0 predator
2016 June R62 8 humus Myriapoda Lithobiidae Lithobius_curtipes 1 2.12 yes yes no 0 1 0 0 predator
2016 June R62 8 humus Coleoptera Staphylinidae Geostiba_circellaris 1 0.11 yes yes no 0 1 0 0 predator
2016 June R62 8 humus Coleoptera Staphylinidae Oxypoda_annularis 1 0.06 yes yes no 0 1 0 0 predator
2016 June R62 9 humus Annelida Lumbricidae . 1 0.62 yes yes no 0 0 1 0 detritivore
2016 June R62 9 humus Coleoptera Elateridae Athous_subfuscus 1 5.73 yes yes yes 0 0.8 0.1 0.1 combined
2016 June R62 9 humus Coleoptera Elateridae Dalopius_marginatus 1 1.56 yes yes yes 0 0.8 0.1 0.1 combined
2016 June R62 9 humus Araneae Linyphiidae Gen_sp 1 0.15 yes yes no 0 1 0 0 predator
2016 June R62 9 humus Myriapoda Lithobiidae Lithobius_sp 1 0.03 yes yes no 0 1 0 0 predator
2016 June R62 9 humus Coleoptera Staphylinidae Drusilla_canaliculata 1 0.68 yes yes no 0 1 0 0 predator
2016 June R62 9 mineral Coleoptera Elateridae Athous_subfuscus 1 5.99 yes yes yes 0 0.8 0.1 0.1 combined
2016 June R62 10 humus Coleoptera Elateridae Athous_subfuscus 1 1.43 yes yes yes 0 0.8 0.1 0.1 combined
2016 June R62 10 humus Coleoptera Elateridae Dalopius_marginatus 1 0.92 yes yes yes 0 0.8 0.1 0.1 combined
2016 June R62 10 humus Hymenoptera Formicidae Myrmica_ruginodis 1 0.31 Yes Yes no 0 1 0 0 predator
2016 June R62 10 humus Araneae Linyphiidae Gen_sp 1 0.17 yes yes no 0 1 0 0 predator
2016 June R62 10 humus Araneae Linyphiidae Minyriolus_pusillus 1 0.33 yes yes no 0 1 0 0 predator
2016 June R62 10 humus Myriapoda Lithobiidae Lithobius_curtipes 1 0.39 yes yes no 0 1 0 0 predator
2016 June R62 10 humus Myriapoda Lithobiidae Lithobius_curtipes 1 1.44 yes yes no 0 1 0 0 predator
2016 June R62 10 humus Myriapoda Lithobiidae Lithobius_sp 1 0.45 yes yes no 0 1 0 0 predator
2016 June R62 10 humus Araneae Lycosidae Gen_sp 1 0.17 yes yes no 0 1 0 0 predator
2016 June R62 10 humus Coleoptera Staphylinidae Platydracus_fulvipes 1 21.32 yes yes no 0 1 0 0 predator
2016 June R62 10 humus Araneae Tetragnathidae Pachygnatha_sp 1 0.17 yes yes no 0 1 0 0 predator
2016 June R62 10 humus Coleoptera . . 1 0.44 . . . .
2016 June R62 10 mineral Coleoptera Elateridae Athous_subfuscus 1 5.99 yes yes yes 0 0.8 0.1 0.1 combined
2016 June R63 1 humus Hymenoptera Formicidae Formica_fusca 1 0.92 Yes Yes no 0 1 0 0 predator
2016 June R63 1 mineral Raphidioptera Raphidiidae Xanthostigma_xanthostigma 1 3.32 no no no 0 1 0 0 predator
2016 June R63 1 mineral Coleoptera Elateridae Selatosomus_melancholicus 1 10.59 yes yes yes 0.6 0.13 0.13 0.13 combined
2016 June R63 2 humus Coleoptera Leiodidae Agathidium_nigripenne 1 0.88 yes yes no 0 0 0 1 fungivore
2016 June R63 2 humus Annelida Lumbricidae Dendrobaena_octaedra 1 5.63 yes yes no 0 0 1 0 detritivore
2016 June R63 2 humus Coleoptera Elateridae Athous_subfuscus 1 0.55 yes yes yes 0 0.8 0.1 0.1 combined

2016 June R63 2 humus Hymenoptera Formicidae Myrmica_ruginodis 1 0.72 Yes Yes no 0 1 0 0 predator
2016 June R63 2 humus Araneae Hahniidae Gen_sp 1 0.13 yes yes no 0 1 0 0 predator
2016 June R63 2 humus Myriapoda Lithobiidae Lithobius_sp 1 0.09 yes yes no 0 1 0 0 predator
2016 June R63 2 humus Hemiptera Loriculidae Loricula_exilis 1 0.06 yes yes no 0 1 0 0 predator
2016 June R63 2 humus Coleoptera Staphylinidae Bolitochara_pulchra 1 0.46 yes yes no 0 1 0 0 predator
2016 June R63 2 humus Coleoptera Staphylinidae Drusilla_canaliculata 1 0.69 yes yes no 0 1 0 0 predator
2016 June R63 2 humus Coleoptera Staphylinidae Drusilla_canaliculata 1 0.89 yes yes no 0 1 0 0 predator
2016 June R63 2 mineral Raphidioptera Raphidiidae Xanthostigma_xanthostigma 1 3.32 no no no 0 1 0 0 predator
2016 June R63 2 mineral Coleoptera Elateridae Selatosomus_melancholicus 1 10.59 yes yes yes 0.6 0.13 0.13 0.13 combined
2016 June R63 3 humus Coleoptera Elateridae Dalopius_marginatus 1 1.70 yes yes yes 0 0.8 0.1 0.1 combined
2016 June R63 3 mineral Coleoptera Elateridae Dalopius_marginatus 1 1.75 yes yes yes 0 0.8 0.1 0.1 combined
2016 June R63 3 mineral Coleoptera Elateridae Selatosomus_melancholicus 1 21.42 yes yes yes 0.6 0.13 0.13 0.13 combined
2016 June R63 4 humus Coleoptera Elateridae Athous_subfuscus 1 0.91 yes yes yes 0 0.8 0.1 0.1 combined
2016 June R63 4 humus Coleoptera Elateridae Athous_subfuscus 1 1.13 yes yes yes 0 0.8 0.1 0.1 combined
2016 June R63 4 humus Coleoptera Elateridae Athous_subfuscus 1 1.74 yes yes yes 0 0.8 0.1 0.1 combined
2016 June R63 4 humus Coleoptera Carabidae Calathus_micropterus 1 4.46 yes yes no 0 1 0 0 predator
2016 June R63 4 humus Araneae Linyphiidae Gen_sp 3 0.39 yes yes no 0 1 0 0 predator
2016 June R63 4 humus Araneae Linyphiidae Neriene_clathrata 1 1.68 yes yes no 0 1 0 0 predator
2016 June R63 4 humus Coleoptera Staphylinidae . 1 1.37 yes yes no 0 1 0 0 predator
2016 June R63 4 mineral Coleoptera Elateridae Dalopius_marginatus 1 1.75 yes yes yes 0 0.8 0.1 0.1 combined
2016 June R63 4 mineral Coleoptera Elateridae Selatosomus_melancholicus 1 21.42 yes yes yes 0.6 0.13 0.13 0.13 combined
2016 June R63 5 humus Annelida Lumbricidae . 1 2.40 yes yes no 0 0 1 0 detritivore
2016 June R63 5 humus Annelida Lumbricidae Dendrobaena_octaedra 1 11.21 yes yes no 0 0 1 0 detritivore
2016 June R63 5 humus Annelida Lumbricidae Dendrodrilus_rubidus_tenuis 1 16.42 yes yes no 0 0 1 0 detritivore
2016 June R63 5 humus Araneae Linyphiidae Tapinocyba_pallens 2 0.38 yes yes no 0 1 0 0 predator
2016 June R63 5 humus Coleoptera Staphylinidae . 1 0.37 yes yes no 0 1 0 0 predator
2016 June R63 5 humus Coleoptera Staphylinidae Xantholinus_tricolor 1 2.04 yes yes no 0 1 0 0 predator
2016 June R63 5 mineral Coleoptera Elateridae Paraphotistus_impressus 1 21.19 yes yes yes 0.6 0.13 0.13 0.13 combined
2016 June R63 6 humus Coleoptera Staphylinidae Tachyporus_sp 1 0.24 yes yes no 0 0.5 0.5 0 combined
2016 June R63 6 humus Coleoptera Elateridae Dalopius_marginatus 1 2.49 yes yes yes 0 0.8 0.1 0.1 combined
2016 June R63 6 humus Coleoptera Elateridae Dalopius_marginatus 1 2.81 yes yes yes 0 0.8 0.1 0.1 combined
2016 June R63 6 humus Coleoptera Staphylinidae Oxypoda_annularis 1 0.08 yes yes no 0 1 0 0 predator
2016 June R63 6 humus Coleoptera . . 1 0.38 . . . .
2016 June R63 6 mineral Coleoptera Elateridae Paraphotistus_impressus 1 21.19 yes yes yes 0.6 0.13 0.13 0.13 combined
2016 June R63 7 humus Coleoptera Elateridae Dalopius_marginatus 1 0.60 yes yes yes 0 0.8 0.1 0.1 combined
2016 June R63 7 humus Coleoptera Elateridae Dalopius_marginatus 1 1.55 yes yes yes 0 0.8 0.1 0.1 combined
2016 June R63 7 humus Coleoptera Elateridae Dalopius_marginatus 1 3.85 yes yes yes 0 0.8 0.1 0.1 combined
2016 June R63 7 humus Diptera Rhagionidae Rhagio_lineola 1 1.85 yes yes no 0 1 0 0 predator
2016 June R63 7 humus Diptera Rhagionidae Rhagio_lineola 1 2.17 yes yes no 0 1 0 0 predator
2016 June R63 7 mineral Coleoptera Elateridae Dalopius_marginatus 1 0.29 yes yes yes 0 0.8 0.1 0.1 combined
2016 June R63 8 humus Araneae Linyphiidae Agyneta_conigera 1 0.48 yes yes no 0 1 0 0 predator
2016 June R63 8 humus Araneae Linyphiidae Gen_sp 3 0.50 yes yes no 0 1 0 0 predator
2016 June R63 8 humus Coleoptera Staphylinidae Geostiba_circellaris 1 0.09 yes yes no 0 1 0 0 predator
2016 June R63 8 humus Hemiptera Cydnidae Adomerus_biguttatus 1 0.87 yes yes yes 1 0 0 0 herbivore
2016 June R63 8 mineral Coleoptera Elateridae Dalopius_marginatus 1 0.29 yes yes yes 0 0.8 0.1 0.1 combined
2016 June R63 9 humus Coleoptera Elateridae Paraphotistus_impressus 1 4.56 yes yes yes 0.6 0.13 0.13 0.13 combined
2016 June R63 9 mineral Coleoptera Elateridae Athous_subfuscus 1 4.63 yes yes yes 0 0.8 0.1 0.1 combined
2016 June R63 9 mineral Coleoptera Elateridae Dalopius_marginatus 1 0.99 yes yes yes 0 0.8 0.1 0.1 combined
2016 June R63 9 mineral Coleoptera Elateridae Dalopius_marginatus 1 1.67 yes yes yes 0 0.8 0.1 0.1 combined
2016 June R63 9 mineral Diptera Asilidae Dioctria_hyalipennis 1 2.52 yes yes no 0 1 0 0 predator
2016 June R63 9 mineral Coleoptera Elateridae Selatosomus_melancholicus 1 5.61 yes yes yes 0.6 0.13 0.13 0.13 combined
2016 June R63 9 mineral Coleoptera Elateridae Selatosomus_melancholicus 1 6.48 yes yes yes 0.6 0.13 0.13 0.13 combined
2016 June R63 10 humus Coleoptera Elateridae Dalopius_marginatus 1 0.67 yes yes yes 0 0.8 0.1 0.1 combined

2016 June R63 10 humus Araneae Clubionidae Gen_sp 1 0.41 yes yes no 0 1 0 0 predator
2016 June R63 10 humus Araneae Linyphiidae Gen_sp 2 0.25 yes yes no 0 1 0 0 predator
2016 June R63 10 humus Araneae Linyphiidae Tapinocyba_pallens 1 1.14 yes yes no 0 1 0 0 predator
2016 June R63 10 humus Myriapoda Lithobiidae Lithobius_curtipes 1 0.59 yes yes no 0 1 0 0 predator
2016 June R63 10 humus Araneae Theridiidae Gen_sp 1 0.59 yes yes no 0 1 0 0 predator
2016 June R63 10 humus Hemiptera Cydnidae Adomerus_biguttatus 1 3.53 yes yes yes 1 0 0 0 herbivore
2016 June R63 10 mineral Coleoptera Elateridae Athous_subfuscus 1 4.63 yes yes yes 0 0.8 0.1 0.1 combined
2016 June R63 10 mineral Coleoptera Elateridae Dalopius_marginatus 1 0.99 yes yes yes 0 0.8 0.1 0.1 combined
2016 June R63 10 mineral Coleoptera Elateridae Dalopius_marginatus 1 1.67 yes yes yes 0 0.8 0.1 0.1 combined
2016 June R63 10 mineral Diptera Asilidae Dioctria_hyalipennis 1 2.52 yes yes no 0 1 0 0 predator
2016 June R63 10 mineral Coleoptera Elateridae Selatosomus_melancholicus 1 5.61 yes yes yes 0.6 0.13 0.13 0.13 combined
2016 June R63 10 mineral Coleoptera Elateridae Selatosomus_melancholicus 1 6.48 yes yes yes 0.6 0.13 0.13 0.13 combined
2016 June R64 1 humus Coleoptera Elateridae Eanus_costalis 1 4.79 yes yes no 0 0.5 0.5 0 combined
2016 June R64 1 humus Coleoptera Elateridae Athous_subfuscus 1 0.17 yes yes yes 0 0.8 0.1 0.1 combined
2016 June R64 1 humus Coleoptera Elateridae Athous_subfuscus 1 0.83 yes yes yes 0 0.8 0.1 0.1 combined
2016 June R64 1 humus Araneae Linyphiidae Gen_sp 1 1.30 yes yes no 0 1 0 0 predator
2016 June R64 1 humus Araneae Linyphiidae Porrhomma_pallidum 1 0.97 yes yes no 0 1 0 0 predator
2016 June R64 1 humus Diptera Rhagionidae Rhagio_scolopaceus 1 1.47 yes yes no 0 1 0 0 predator
2016 June R64 1 humus Diptera Rhagionidae Rhagio_sp 1 1.10 yes yes no 0 1 0 0 predator
2016 June R64 1 mineral Araneae Linyphiidae Gen_sp 1 0.13 yes yes no 0 1 0 0 predator
2016 June R64 2 humus Myriapoda Lithobiidae Lithobius_sp 1 0.09 yes yes no 0 1 0 0 predator
2016 June R64 2 mineral Araneae Linyphiidae Gen_sp 1 0.13 yes yes no 0 1 0 0 predator
2016 June R64 3 humus Annelida Lumbricidae Eiseniella_tetraedra 1 0.42 yes yes no 0 0 1 0 detritivore
2016 June R64 3 humus Coleoptera Cantharidae Podistra_schoenherri 1 0.18 yes yes no 0 1 0 0 predator
2016 June R64 3 humus Araneae Linyphiidae Gen_sp 1 0.13 yes yes no 0 1 0 0 predator
2016 June R64 3 humus Araneae Linyphiidae Porrhomma_pallidum 1 1.64 yes yes no 0 1 0 0 predator
2016 June R64 3 humus Diptera Rhagionidae Rhagio_lineola 1 2.13 yes yes no 0 1 0 0 predator
2016 June R64 3 humus Coleoptera Staphylinidae Oxypoda_annularis 1 0.04 yes yes no 0 1 0 0 predator
2016 June R64 3 humus Araneae Theridiidae Robertus_scoticus 2 1.11 yes yes no 0 1 0 0 predator
2016 June R64 3 humus Araneae Theridiidae Robertus_sp 2 0.37 yes yes no 0 1 0 0 predator
2016 June R64 4 humus Myriapoda Lithobiidae Lithobius_curtipes 1 1.48 yes yes no 0 1 0 0 predator
2016 June R64 4 humus Diptera Rhagionidae Rhagio_scolopaceus 1 3.51 yes yes no 0 1 0 0 predator
2016 June R64 5 humus Annelida Lumbricidae Dendrobaena_octaedra 1 8.55 yes yes no 0 0 1 0 detritivore
2016 June R64 5 humus Coleoptera Staphylinidae Mocyta_fungi 1 0.09 yes yes no 0 1 0 0 predator
2016 June R64 5 mineral Hemiptera Lygaeidae Drymus_brunneus 1 1.15 yes yes no 1 0 0 0 herbivore
2016 June R64 6 humus Coleoptera Elateridae Ampedus_cf_nigrinus 1 4.46 yes yes no 0 0.5 0.5 0 combined
2016 June R64 6 humus Coleoptera Elateridae Athous_subfuscus 1 0.82 yes yes yes 0 0.8 0.1 0.1 combined
2016 June R64 6 humus Coleoptera Elateridae Athous_subfuscus 1 4.13 yes yes yes 0 0.8 0.1 0.1 combined
2016 June R64 6 humus Araneae Linyphiidae Gen_sp 1 0.69 yes yes no 0 1 0 0 predator
2016 June R64 6 humus Coleoptera Staphylinidae Mocyta_fungi 1 0.08 yes yes no 0 1 0 0 predator
2016 June R64 6 humus Coleoptera Staphylinidae Mocyta_fungi 1 0.09 yes yes no 0 1 0 0 predator
2016 June R64 6 humus Coleoptera Staphylinidae Mocyta_fungi 1 0.10 yes yes no 0 1 0 0 predator
2016 June R64 6 mineral Hemiptera Lygaeidae Drymus_brunneus 1 1.15 yes yes no 1 0 0 0 herbivore
2016 June R64 7 humus Annelida Lumbricidae Dendrobaena_octaedra 2 19.10 yes yes no 0 0 1 0 detritivore
2016 June R64 7 humus Coleoptera Elateridae Athous_subfuscus 1 4.03 yes yes yes 0 0.8 0.1 0.1 combined
2016 June R64 7 humus Myriapoda Lithobiidae Lithobius_curtipes 1 1.19 yes yes no 0 1 0 0 predator
2016 June R64 7 humus Myriapoda Lithobiidae Lithobius_curtipes 1 1.98 yes yes no 0 1 0 0 predator
2016 June R64 7 humus Diptera Rhagionidae Rhagio_sp 1 0.22 yes yes no 0 1 0 0 predator
2016 June R64 7 humus Coleoptera Staphylinidae Oxypoda_annularis 1 0.06 yes yes no 0 1 0 0 predator
2016 June R64 7 humus Araneae Theridiidae Robertus_sp 1 0.25 yes yes no 0 1 0 0 predator
2016 June R64 7 humus Diptera Chironomidae Bryophaenocladius_sp 1 0.06 yes yes yes 0.25 0 0.75 0 combined
2016 June R64 7 mineral Coleoptera Elateridae Athous_subfuscus 1 0.34 yes yes yes 0 0.8 0.1 0.1 combined
2016 June R64 7 mineral Coleoptera Elateridae Athous_subfuscus 1 2.49 yes yes yes 0 0.8 0.1 0.1 combined

2016 June R64 7 mineral Coleoptera Elateridae Athous_subfuscus 1 4.58 yes yes yes 0 0.8 0.1 0.1 combined
2016 June R64 8 humus Coleoptera Leiodidae Agathidium_confusum 1 0.27 yes yes no 0 0 0 1 fungivore
2016 June R64 8 humus Coleoptera Elateridae Athous_subfuscus 1 0.34 yes yes yes 0 0.8 0.1 0.1 combined
2016 June R64 8 humus Coleoptera Elateridae Athous_subfuscus 1 0.35 yes yes yes 0 0.8 0.1 0.1 combined
2016 June R64 8 humus Coleoptera Elateridae Athous_subfuscus 1 1.57 yes yes yes 0 0.8 0.1 0.1 combined
2016 June R64 8 humus Coleoptera Elateridae Athous_subfuscus 1 1.84 yes yes yes 0 0.8 0.1 0.1 combined
2016 June R64 8 humus Coleoptera Cantharidae Rhagonycha_atra 1 0.26 yes yes no 0 1 0 0 predator
2016 June R64 8 humus Araneae Linyphiidae Gen_sp 1 0.08 yes yes no 0 1 0 0 predator
2016 June R64 8 humus Coleoptera Staphylinidae Oxypoda_annularis 1 0.09 yes yes no 0 1 0 0 predator
2016 June R64 8 mineral Coleoptera Elateridae Athous_subfuscus 1 0.34 yes yes yes 0 0.8 0.1 0.1 combined
2016 June R64 8 mineral Coleoptera Elateridae Athous_subfuscus 1 2.49 yes yes yes 0 0.8 0.1 0.1 combined
2016 June R64 8 mineral Coleoptera Elateridae Athous_subfuscus 1 4.58 yes yes yes 0 0.8 0.1 0.1 combined
2016 June R64 9 humus Coleoptera Leiodidae Agathidium_nigripenne 1 0.81 yes yes no 0 0 0 1 fungivore
2016 June R64 9 humus Coleoptera Elateridae Athous_subfuscus 1 1.47 yes yes yes 0 0.8 0.1 0.1 combined
2016 June R64 9 humus Diptera Rhagionidae Rhagio_scolopaceus 1 0.53 yes yes no 0 1 0 0 predator
2016 June R64 9 humus Coleoptera Staphylinidae Othius_punctulatus 1 3.28 yes yes no 0 1 0 0 predator
2016 June R64 10 humus Araneae Linyphiidae Centromerus_arcanus 1 0.08 yes yes no 0 1 0 0 predator
2016 June R64 10 humus Araneae Linyphiidae Tapinocyba_pallens 1 0.29 yes yes no 0 1 0 0 predator
2016 June R64 10 humus Myriapoda Lithobiidae Lithobius_curtipes 1 1.17 yes yes no 0 1 0 0 predator
2016 June R64 10 humus Myriapoda Lithobiidae Lithobius_curtipes 1 1.57 yes yes no 0 1 0 0 predator
2016 June R65 1 humus Coleoptera Elateridae Ampedus_balteatus 1 0.52 yes yes no 0 0.5 0.5 0 combined
2016 June R65 1 humus Coleoptera Cantharidae Podistra_schoenherri 1 0.25 yes yes no 0 1 0 0 predator
2016 June R65 2 humus Coleoptera Elateridae Athous_subfuscus 1 0.37 yes yes yes 0 0.8 0.1 0.1 combined
2016 June R65 2 humus Coleoptera Elateridae Athous_subfuscus 1 3.68 yes yes yes 0 0.8 0.1 0.1 combined
2016 June R65 2 humus Coleoptera Cantharidae Podistra_schoenherri 1 0.39 yes yes no 0 1 0 0 predator
2016 June R65 2 humus Araneae Linyphiidae Gen_sp 1 0.29 yes yes no 0 1 0 0 predator
2016 June R65 2 humus Araneae Linyphiidae Tibioplus_diversus 1 1.02 yes yes no 0 1 0 0 predator
2016 June R65 2 humus Myriapoda Lithobiidae Lithobius_curtipes 1 0.27 yes yes no 0 1 0 0 predator
2016 June R65 2 humus Myriapoda Lithobiidae Lithobius_curtipes 1 1.75 yes yes no 0 1 0 0 predator
2016 June R65 2 humus Myriapoda Lithobiidae Lithobius_sp 1 0.10 yes yes no 0 1 0 0 predator
2016 June R65 2 humus Diptera Rhagionidae Rhagio_scolopaceus 1 1.05 yes yes no 0 1 0 0 predator
2016 June R65 2 humus Coleoptera Staphylinidae . 1 0.16 yes yes no 0 1 0 0 predator
2016 June R65 2 humus Coleoptera Staphylinidae Stenus_clavicornis 1 0.69 yes yes no 0 1 0 0 predator
2016 June R65 2 humus Araneae Theridiidae Robertus_scoticus 1 0.19 yes yes no 0 1 0 0 predator
2016 June R65 2 humus Araneae Theridiidae Robertus_sp 1 0.29 yes yes no 0 1 0 0 predator
2016 June R65 3 humus Diptera Hybotidae Bicellaria_intermedia 1 0.23 yes yes no 0 1 0 0 predator
2016 June R65 3 humus Araneae Linyphiidae Gen_sp 6 3.02 yes yes no 0 1 0 0 predator
2016 June R65 3 humus Coleoptera Staphylinidae Anthophagus_omalinus 1 0.28 no no no 0 1 0 0 predator
2016 June R65 3 humus Coleoptera Staphylinidae Stenus_geniculatus 1 0.34 yes yes no 0 1 0 0 predator
2016 June R65 3 humus Araneae Theridiidae Robertus_scoticus 2 1.06 yes yes no 0 1 0 0 predator
2016 June R65 3 humus Diptera Chironomidae Bryophaenocladius_ictericus 1 0.05 yes yes yes 0.25 0 0.75 0 combined
2016 June R65 4 humus Coleoptera Elateridae Eanus_costalis 1 0.44 yes yes no 0 0.5 0.5 0 combined
2016 June R65 4 humus Araneae Salticidae Neon_reticulatus 1 0.72 yes yes no 0 1 0 0 predator
2016 June R65 4 humus Coleoptera Elateridae Paraphotistus_impressus 1 8.82 yes yes yes 0.6 0.13 0.13 0.13 combined
2016 June R65 5 humus Coleoptera Elateridae Eanus_costalis 1 2.77 yes yes no 0 0.5 0.5 0 combined
2016 June R65 5 humus Coleoptera Elateridae Eanus_costalis 1 3.99 yes yes no 0 0.5 0.5 0 combined
2016 June R65 5 humus Coleoptera Elateridae Eanus_costalis 1 6.10 yes yes no 0 0.5 0.5 0 combined
2016 June R65 5 humus Coleoptera Elateridae Athous_subfuscus 1 0.66 yes yes yes 0 0.8 0.1 0.1 combined
2016 June R65 5 humus Coleoptera Elateridae Athous_subfuscus 1 0.81 yes yes yes 0 0.8 0.1 0.1 combined
2016 June R65 5 humus Coleoptera Elateridae Athous_subfuscus 1 2.84 yes yes yes 0 0.8 0.1 0.1 combined
2016 June R65 5 humus Coleoptera Elateridae Athous_subfuscus 1 4.08 yes yes yes 0 0.8 0.1 0.1 combined
2016 June R65 5 humus Araneae Linyphiidae Gen_sp 1 0.13 yes yes no 0 1 0 0 predator
2016 June R65 5 humus Araneae Linyphiidae Tapinocyba_pallens 1 0.21 yes yes no 0 1 0 0 predator

2016 June R65 5 humus Coleoptera Staphylinidae Othius_subuliformis 1 0.57 yes yes no 0 1 0 0 predator
2016 June R65 5 humus Coleoptera Staphylinidae Oxypoda_annularis 1 0.07 yes yes no 0 1 0 0 predator
2016 June R65 5 humus Coleoptera Staphylinidae Stenus_sp 1 0.24 yes yes no 0 1 0 0 predator
2016 June R65 5 humus Coleoptera Rhynchitidae Deporaus_betulae 1 2.26 yes no no 1 0 0 0 herbivore
2016 June R65 5 humus Coleoptera . . 1 0.11 . . . .
2016 June R65 5 mineral Coleoptera Elateridae Eanus_costalis 1 2.90 yes yes no 0 0.5 0.5 0 combined
2016 June R65 6 humus Coleoptera Elateridae Athous_subfuscus 1 1.64 yes yes yes 0 0.8 0.1 0.1 combined
2016 June R65 6 humus Coleoptera Elateridae Athous_subfuscus 1 3.98 yes yes yes 0 0.8 0.1 0.1 combined
2016 June R65 6 humus Coleoptera Cantharidae Podistra_schoenherri 1 3.61 yes yes no 0 1 0 0 predator
2016 June R65 6 humus Myriapoda Lithobiidae Lithobius_curtipes 1 0.50 yes yes no 0 1 0 0 predator
2016 June R65 6 humus Araneae Thomisidae Ozyptila_trux 1 5.17 yes yes no 0 1 0 0 predator
2016 June R65 6 humus Coleoptera Elateridae Paraphotistus_impressus 1 0.64 yes yes yes 0.6 0.13 0.13 0.13 combined
2016 June R65 6 mineral Coleoptera Elateridae Eanus_costalis 1 2.90 yes yes no 0 0.5 0.5 0 combined
2016 June R65 7 humus Coleoptera Cantharidae Podistra_schoenherri 1 2.63 yes yes no 0 1 0 0 predator
2016 June R65 7 humus Coleoptera Cantharidae Podistra_schoenherri 1 3.62 yes yes no 0 1 0 0 predator
2016 June R65 7 humus Araneae Linyphiidae Gen_sp 14 1.18 yes yes no 0 1 0 0 predator
2016 June R65 7 humus Araneae Linyphiidae Tapinocyba_pallens 1 0.23 yes yes no 0 1 0 0 predator
2016 June R65 7 humus Coleoptera Elateridae Paraphotistus_impressus 1 5.75 yes yes yes 0.6 0.13 0.13 0.13 combined
2016 June R65 7 mineral Coleoptera Curculionidae Polydrusus_tereticollis 1 6.25 yes yes yes 1 0 0 0 herbivore
2016 June R65 8 humus Coleoptera Elateridae Eanus_costalis 1 0.34 yes yes no 0 0.5 0.5 0 combined
2016 June R65 8 humus Coleoptera Elateridae Eanus_costalis 1 0.34 yes yes no 0 0.5 0.5 0 combined
2016 June R65 8 humus Coleoptera Elateridae Eanus_costalis 1 0.42 yes yes no 0 0.5 0.5 0 combined
2016 June R65 8 humus Hymenoptera Formicidae Myrmica_ruginodis 1 0.99 Yes Yes no 0 1 0 0 predator
2016 June R65 8 humus Araneae Linyphiidae Gen_sp 1 0.13 yes yes no 0 1 0 0 predator
2016 June R65 8 mineral Coleoptera Curculionidae Polydrusus_tereticollis 1 6.25 yes yes yes 1 0 0 0 herbivore
2016 June R65 9 humus Coleoptera Elateridae Ampedus_cf_balteatus 1 2.60 yes yes no 0 0.5 0.5 0 combined
2016 June R65 9 humus Coleoptera Elateridae Eanus_costalis 1 1.46 yes yes no 0 0.5 0.5 0 combined
2016 June R65 9 humus Coleoptera Elateridae Eanus_costalis 1 2.60 yes yes no 0 0.5 0.5 0 combined
2016 June R65 9 humus Hymenoptera Formicidae Formica_aquilonia 1 0.87 Yes Yes no 0 0.8 0.2 0 combined
2016 June R65 9 humus Coleoptera Cantharidae Podistra_schoenherri 1 0.19 yes yes no 0 1 0 0 predator
2016 June R65 9 humus Coleoptera Cantharidae Podistra_schoenherri 1 0.23 yes yes no 0 1 0 0 predator
2016 June R65 9 humus Coleoptera Staphylinidae Oxypoda_annularis 1 0.07 yes yes no 0 1 0 0 predator
2016 June R65 9 humus Coleoptera Staphylinidae Oxypoda_annularis 1 0.07 yes yes no 0 1 0 0 predator
2016 June R65 9 humus Coleoptera Staphylinidae Oxypoda_annularis 1 0.08 yes yes no 0 1 0 0 predator
2016 June R65 9 humus Coleoptera Curculionidae Otiorhynchus_ligustici 1 11.01 yes yes yes 1 0 0 0 herbivore
2016 June R65 10 humus Annelida Lumbricidae Dendrodrilus_rubidus_tenuis 2 50.14 yes yes no 0 0 1 0 detritivore
2016 June R65 10 humus Araneae Linyphiidae Gen_sp 1 0.17 yes yes no 0 1 0 0 predator
2016 June R65 10 humus Myriapoda Lithobiidae Lithobius_curtipes 1 0.69 yes yes no 0 1 0 0 predator
2016 June R65 10 humus Diptera Rhagionidae Rhagio_scolopaceus 1 0.66 yes yes no 0 1 0 0 predator
2016 June R66 1 humus Coleoptera Elateridae Athous_subfuscus 1 1.14 yes yes yes 0 0.8 0.1 0.1 combined
2016 June R66 1 humus Coleoptera Elateridae Athous_subfuscus 1 1.18 yes yes yes 0 0.8 0.1 0.1 combined
2016 June R66 1 humus Coleoptera Elateridae Athous_subfuscus 1 1.97 yes yes yes 0 0.8 0.1 0.1 combined
2016 June R66 2 humus Coleoptera Elateridae Eanus_costalis 1 0.29 yes yes no 0 0.5 0.5 0 combined
2016 June R66 2 humus Coleoptera Elateridae Athous_subfuscus 1 3.04 yes yes yes 0 0.8 0.1 0.1 combined
2016 June R66 2 humus Coleoptera Elateridae Athous_subfuscus 1 3.47 yes yes yes 0 0.8 0.1 0.1 combined
2016 June R66 2 humus Araneae Liocranidae Gen_sp 1 0.68 yes yes no 0 1 0 0 predator
2016 June R66 2 humus Myriapoda Lithobiidae Lithobius_sp 1 0.14 yes yes no 0 1 0 0 predator
2016 June R66 3 humus Annelida Lumbricidae Eiseniella_tetraedra 1 0.31 yes yes no 0 0 1 0 detritivore
2016 June R66 3 humus Coleoptera Elateridae Athous_subfuscus 1 1.51 yes yes yes 0 0.8 0.1 0.1 combined
2016 June R66 3 humus Coleoptera Elateridae Athous_subfuscus 1 1.82 yes yes yes 0 0.8 0.1 0.1 combined
2016 June R66 3 humus Coleoptera Elateridae Athous_subfuscus 1 6.29 yes yes yes 0 0.8 0.1 0.1 combined
2016 June R66 3 humus Araneae Linyphiidae Gen_sp 1 0.13 yes yes no 0 1 0 0 predator
2016 June R66 3 humus Coleoptera Staphylinidae Othius_lapidicola 1 0.07 yes yes no 0 1 0 0 predator

2016 June R66 4 humus Coleoptera Elateridae Eanus_costalis 1 2.23 yes yes no 0 0.5 0.5 0 combined
2016 June R66 4 humus Coleoptera Elateridae Athous_subfuscus 1 1.36 yes yes yes 0 0.8 0.1 0.1 combined
2016 June R66 4 humus Coleoptera Elateridae Athous_subfuscus 1 1.65 yes yes yes 0 0.8 0.1 0.1 combined
2016 June R66 4 humus Hemiptera Anthocoridae Anthocoris_sp 1 0.12 yes no no 0 1 0 0 predator
2016 June R66 5 humus Coleoptera Elateridae Athous_subfuscus 1 0.42 yes yes yes 0 0.8 0.1 0.1 combined
2016 June R66 5 humus Coleoptera Elateridae Athous_subfuscus 1 0.60 yes yes yes 0 0.8 0.1 0.1 combined
2016 June R66 5 humus Coleoptera Staphylinidae Anthophagus_omalinus 1 0.23 no no no 0 1 0 0 predator
2016 June R66 5 humus Coleoptera Staphylinidae Oxypoda_annularis 1 0.08 yes yes no 0 1 0 0 predator
2016 June R66 6 humus Coleoptera Elateridae Athous_subfuscus 1 0.85 yes yes yes 0 0.8 0.1 0.1 combined
2016 June R66 6 humus Coleoptera Elateridae Athous_subfuscus 1 2.58 yes yes yes 0 0.8 0.1 0.1 combined
2016 June R66 6 humus Coleoptera Elateridae Athous_subfuscus 1 4.99 yes yes yes 0 0.8 0.1 0.1 combined
2016 June R66 6 humus Hymenoptera Formicidae Myrmica_ruginodis 1 0.44 Yes Yes no 0 1 0 0 predator
2016 June R66 6 humus Myriapoda Lithobiidae Lithobius_curtipes 1 1.47 yes yes no 0 1 0 0 predator
2016 June R66 6 humus Coleoptera Staphylinidae Drusilla_canaliculata 1 0.55 yes yes no 0 1 0 0 predator
2016 June R66 6 humus Araneae Theridiidae Robertus_scoticus 1 0.37 yes yes no 0 1 0 0 predator
2016 June R66 7 humus Annelida Lumbricidae Dendrobaena_octaedra 1 5.24 yes yes no 0 0 1 0 detritivore
2016 June R66 7 humus Coleoptera Elateridae Eanus_costalis 1 5.55 yes yes no 0 0.5 0.5 0 combined
2016 June R66 7 humus Araneae Linyphiidae Tapinocyba_pallens 1 0.13 yes yes no 0 1 0 0 predator
2016 June R66 7 humus Coleoptera Staphylinidae Anthophagus_omalinus 1 0.31 no no no 0 1 0 0 predator
2016 June R66 7 humus Araneae Theridiidae Robertus_sp 1 0.13 yes yes no 0 1 0 0 predator
2016 June R66 8 humus Coleoptera Elateridae Eanus_costalis 1 0.77 yes yes no 0 0.5 0.5 0 combined
2016 June R66 8 humus Hemiptera Anthocoridae Anthocoris_sp 1 0.11 yes no no 0 1 0 0 predator
2016 June R66 8 humus Coleoptera Cantharidae . 1 0.38 yes yes no 0 1 0 0 predator
2016 June R66 8 humus Myriapoda Lithobiidae Lithobius_curtipes 1 1.42 yes yes no 0 1 0 0 predator
2016 June R66 9 humus Annelida Lumbricidae Dendrodrilus_rubidus_tenuis 1 26.64 yes yes no 0 0 1 0 detritivore
2016 June R66 9 humus Coleoptera Elateridae Eanus_costalis 1 7.16 yes yes no 0 0.5 0.5 0 combined
2016 June R66 9 humus Coleoptera Elateridae Athous_subfuscus 1 5.01 yes yes yes 0 0.8 0.1 0.1 combined
2016 June R66 10 humus Coleoptera Elateridae Athous_subfuscus 1 0.93 yes yes yes 0 0.8 0.1 0.1 combined
2016 June R66 10 humus Coleoptera Elateridae Athous_subfuscus 1 2.09 yes yes yes 0 0.8 0.1 0.1 combined
2016 June R66 10 humus Coleoptera Elateridae Athous_subfuscus 1 2.10 yes yes yes 0 0.8 0.1 0.1 combined
2016 June R66 10 humus Coleoptera Cantharidae Podistra_schoenherri 1 1.63 yes yes no 0 1 0 0 predator
2016 June R66 10 humus Myriapoda Lithobiidae Lithobius_sp 1 0.25 yes yes no 0 1 0 0 predator
2016 June R66 10 humus Coleoptera Staphylinidae Oxypoda_annularis 1 0.07 yes yes no 0 1 0 0 predator
2016 June R67 1 humus Coleoptera Elateridae Athous_subfuscus 1 1.43 yes yes yes 0 0.8 0.1 0.1 combined
2016 June R67 2 humus Coleoptera Elateridae Eanus_costalis 1 1.49 yes yes no 0 0.5 0.5 0 combined
2016 June R67 2 humus Coleoptera Elateridae Athous_subfuscus 1 1.60 yes yes yes 0 0.8 0.1 0.1 combined
2016 June R67 3 humus Coleoptera Elateridae Eanus_costalis 1 0.65 yes yes no 0 0.5 0.5 0 combined
2016 June R67 3 humus Coleoptera Elateridae Eanus_costalis 1 2.70 yes yes no 0 0.5 0.5 0 combined
2016 June R67 3 humus Coleoptera Elateridae Eanus_costalis 1 7.42 yes yes no 0 0.5 0.5 0 combined
2016 June R67 3 humus Coleoptera Elateridae Athous_subfuscus 1 2.07 yes yes yes 0 0.8 0.1 0.1 combined
2016 June R67 3 humus Coleoptera Elateridae Athous_subfuscus 1 4.31 yes yes yes 0 0.8 0.1 0.1 combined
2016 June R67 3 mineral Coleoptera Elateridae Eanus_costalis 1 2.74 yes yes no 0 0.5 0.5 0 combined
2016 June R67 3 mineral Coleoptera Elateridae Eanus_costalis 1 2.95 yes yes no 0 0.5 0.5 0 combined
2016 June R67 4 mineral Coleoptera Elateridae Eanus_costalis 1 2.74 yes yes no 0 0.5 0.5 0 combined
2016 June R67 4 mineral Coleoptera Elateridae Eanus_costalis 1 2.95 yes yes no 0 0.5 0.5 0 combined
2016 June R67 5 humus Coleoptera Elateridae Eanus_costalis 1 3.39 yes yes no 0 0.5 0.5 0 combined
2016 June R67 6 humus Coleoptera Elateridae Eanus_costalis 1 1.06 yes yes no 0 0.5 0.5 0 combined
2016 June R67 6 humus Coleoptera Elateridae Eanus_costalis 1 3.96 yes yes no 0 0.5 0.5 0 combined
2016 June R67 6 humus Coleoptera Elateridae Athous_subfuscus 1 0.43 yes yes yes 0 0.8 0.1 0.1 combined
2016 June R67 7 humus Coleoptera Elateridae Paraphotistus_impressus 1 7.94 yes yes yes 0.6 0.13 0.13 0.13 combined
2016 June R67 10 humus Coleoptera Elateridae Eanus_costalis 1 0.21 yes yes no 0 0.5 0.5 0 combined
2016 June R67 10 humus Coleoptera Staphylinidae Acidota_crenata 1 1.17 yes yes no 0 1 0 0 predator
2016 June R68 1 humus Hymenoptera Formicidae Myrmica_ruginodis 1 0.62 Yes Yes no 0 1 0 0 predator

2016 June R68 1 humus Araneae Theridiidae Robertus_sp 1 0.17 yes yes no 0 1 0 0 predator
2016 June R68 2 humus Coleoptera Cryptophagidae Cryptophagus_setulosus 1 0.26 yes yes no 0 0 0.5 0.5 combined
2016 June R68 2 humus Diptera Syrphidae Parasyrphus_tarsatus 1 8.92 no no no 0 1 0 0 predator
2016 June R68 3 humus Myriapoda Lithobiidae Lithobius_cf_curtipes 1 0.33 yes yes no 0 1 0 0 predator
2016 June R68 4 humus Hymenoptera Formicidae Myrmica_ruginodis 1 0.49 Yes Yes no 0 1 0 0 predator
2016 June R68 4 humus Araneae Gnaphosidae Haplodrassus_sp 1 3.36 yes yes no 0 1 0 0 predator
2016 June R68 5 humus Coleoptera Cantharidae Malthodes_maurus 1 1.09 yes yes no 0 1 0 0 predator
2016 June R68 5 humus Diptera Therevidae Thereva_handlirschi 1 7.30 yes yes no 0 1 0 0 predator
2016 June R68 7 mineral Araneae Theridiidae Robertus_sp 1 0.64 yes yes no 0 1 0 0 predator
2016 June R68 7 mineral Coleoptera Curculionidae Otiorhynchus_nodosus 1 14.21 yes yes yes 1 0 0 0 herbivore
2016 June R68 8 humus Coleoptera Elateridae Eanus_costalis 1 7.61 yes yes no 0 0.5 0.5 0 combined
2016 June R68 8 humus Coleoptera Elateridae Athous_subfuscus 1 0.21 yes yes yes 0 0.8 0.1 0.1 combined
2016 June R68 8 humus Araneae Gnaphosidae Haplodrassus_sp 1 0.13 yes yes no 0 1 0 0 predator
2016 June R68 8 humus Myriapoda Lithobiidae Lithobius_curtipes 1 1.21 yes yes no 0 1 0 0 predator
2016 June R68 8 humus Araneae Theridiidae Gen_sp 1 2.60 yes yes no 0 1 0 0 predator
2016 June R68 8 mineral Araneae Theridiidae Robertus_sp 1 0.64 yes yes no 0 1 0 0 predator
2016 June R68 8 mineral Coleoptera Curculionidae Otiorhynchus_nodosus 1 14.21 yes yes yes 1 0 0 0 herbivore
2016 June R68 9 humus Araneae Philodromidae Tibellus_sp 1 3.06 yes yes no 0 1 0 0 predator
2016 June R68 10 humus Coleoptera Cantharidae Rhagonycha_elongata 1 0.68 yes yes no 0 1 0 0 predator
2016 June R68 10 humus Araneae Liocranidae Gen_sp 1 1.08 yes yes no 0 1 0 0 predator
2016 June R69 1 humus Coleoptera Elateridae Eanus_costalis 1 1.71 yes yes no 0 0.5 0.5 0 combined
2016 June R69 1 humus Coleoptera Elateridae Eanus_costalis 1 2.22 yes yes no 0 0.5 0.5 0 combined
2016 June R69 1 humus Coleoptera Elateridae Liotrichus_affinis 1 12.52 yes yes no 0 0.5 0.5 0 combined
2016 June R69 1 humus Coleoptera Staphylinidae Othius_lapidicola 1 0.69 yes yes no 0 1 0 0 predator
2016 June R69 1 humus Coleoptera Staphylinidae Othius_lapidicola 1 0.72 yes yes no 0 1 0 0 predator
2016 June R69 1 humus Coleoptera Staphylinidae Stenus_sp 1 0.21 yes yes no 0 1 0 0 predator
2016 June R69 1 mineral Coleoptera Elateridae Eanus_costalis 1 11.93 yes yes no 0 0.5 0.5 0 combined
2016 June R69 2 humus Coleoptera Elateridae Eanus_costalis 1 0.54 yes yes no 0 0.5 0.5 0 combined
2016 June R69 2 humus Coleoptera Elateridae Eanus_costalis 1 2.08 yes yes no 0 0.5 0.5 0 combined
2016 June R69 2 humus Coleoptera Elateridae Eanus_costalis 1 5.09 yes yes no 0 0.5 0.5 0 combined
2016 June R69 2 humus Coleoptera Elateridae Liotrichus_affinis 1 7.90 yes yes no 0 0.5 0.5 0 combined
2016 June R69 2 humus Araneae Linyphiidae Semljicola_latus 1 0.11 yes yes no 0 1 0 0 predator
2016 June R69 2 humus Araneae Linyphiidae Tapinocyba_pallens 1 0.08 yes yes no 0 1 0 0 predator
2016 June R69 2 humus Coleoptera Staphylinidae Oxypoda_annularis 1 0.10 yes yes no 0 1 0 0 predator
2016 June R69 2 mineral Coleoptera Elateridae Eanus_costalis 1 11.93 yes yes no 0 0.5 0.5 0 combined
2016 June R69 3 humus Coleoptera Elateridae Eanus_costalis 1 0.99 yes yes no 0 0.5 0.5 0 combined
2016 June R69 3 humus Coleoptera Elateridae Eanus_costalis 1 1.87 yes yes no 0 0.5 0.5 0 combined
2016 June R69 3 humus Coleoptera Elateridae Eanus_costalis 1 2.37 yes yes no 0 0.5 0.5 0 combined
2016 June R69 3 humus Coleoptera Staphylinidae Mycetoporus_sp 1 0.26 yes yes no 0 1 0 0 predator
2016 June R69 3 humus Coleoptera Staphylinidae Mycetoporus_sp 1 0.29 yes yes no 0 1 0 0 predator
2016 June R69 3 humus Coleoptera Staphylinidae Oxypoda_annularis 1 0.08 yes yes no 0 1 0 0 predator
2016 June R69 3 humus Araneae Theridiidae Gen_sp 1 0.36 yes yes no 0 1 0 0 predator
2016 June R69 3 mineral Araneae Theridiidae Robertus_sp 1 0.12 yes yes no 0 1 0 0 predator
2016 June R69 4 humus Coleoptera Elateridae Eanus_costalis 1 2.39 yes yes no 0 0.5 0.5 0 combined
2016 June R69 4 humus Coleoptera Cantharidae Podistra_schoenherri 1 1.91 yes yes no 0 1 0 0 predator
2016 June R69 4 humus Araneae Theridiidae Robertus_sp 1 0.36 yes yes no 0 1 0 0 predator
2016 June R69 4 mineral Araneae Theridiidae Robertus_sp 1 0.12 yes yes no 0 1 0 0 predator
2016 June R69 5 humus Coleoptera Elateridae Eanus_costalis 1 1.77 yes yes no 0 0.5 0.5 0 combined
2016 June R69 5 humus Coleoptera Elateridae Eanus_costalis 1 2.56 yes yes no 0 0.5 0.5 0 combined
2016 June R69 5 humus Coleoptera Elateridae Eanus_costalis 1 4.35 yes yes no 0 0.5 0.5 0 combined
2016 June R69 5 humus Araneae Theridiidae Robertus_sp 1 1.94 yes yes no 0 1 0 0 predator
2016 June R69 5 mineral Coleoptera Elateridae Eanus_costalis 1 6.52 yes yes no 0 0.5 0.5 0 combined
2016 June R69 5 mineral Araneae Theridiidae Robertus_sp 1 0.50 yes yes no 0 1 0 0 predator

2016 June R69 6 humus Coleoptera Elateridae Eanus_costalis 1 5.97 yes yes no 0 0.5 0.5 0 combined
2016 June R69 6 humus Araneae Linyphiidae Ceratinella_brevipes 1 0.41 yes yes no 0 1 0 0 predator
2016 June R69 6 humus Myriapoda Lithobiidae Lithobius_curtipes 1 0.39 yes yes no 0 1 0 0 predator
2016 June R69 6 humus Coleoptera Staphylinidae Oxypoda_annularis 1 0.09 yes yes no 0 1 0 0 predator
2016 June R69 6 mineral Coleoptera Elateridae Eanus_costalis 1 6.52 yes yes no 0 0.5 0.5 0 combined
2016 June R69 6 mineral Araneae Theridiidae Robertus_sp 1 0.50 yes yes no 0 1 0 0 predator
2016 June R69 7 humus Coleoptera Elateridae Eanus_costalis 1 0.40 yes yes no 0 0.5 0.5 0 combined
2016 June R69 7 humus Coleoptera Elateridae Eanus_costalis 1 1.21 yes yes no 0 0.5 0.5 0 combined
2016 June R69 7 humus Diptera Empididae Hilara_abdominalis 1 0.74 yes yes no 0 1 0 0 predator
2016 June R69 7 humus Araneae Theridiidae Robertus_scoticus 1 0.45 yes yes no 0 1 0 0 predator
2016 June R69 8 humus Diptera Heleomyzidae Neoleria_sp 1 1.02 yes yes no 0 0 1 0 detritivore
2016 June R69 8 humus Coleoptera Elateridae Liotrichus_affinis 1 8.45 yes yes no 0 0.5 0.5 0 combined
2016 June R69 8 humus Araneae Linyphiidae Tapinocyba_pallens 1 0.16 yes yes no 0 1 0 0 predator
2016 June R69 8 humus Myriapoda Lithobiidae Lithobius_curtipes 1 2.29 yes yes no 0 1 0 0 predator
2016 June R69 8 humus Coleoptera Staphylinidae Acidota_crenata 1 1.32 yes yes no 0 1 0 0 predator
2016 June R69 9 humus Coleoptera Staphylinidae Atheta_sp 1 0.16 yes yes no 0 1 0 0 predator
2016 June R69 10 humus Coleoptera Elateridae Eanus_costalis 1 1.75 yes yes no 0 0.5 0.5 0 combined
2016 June R69 10 humus Coleoptera Elateridae Eanus_costalis 1 2.03 yes yes no 0 0.5 0.5 0 combined
2016 June R69 10 humus Coleoptera Cantharidae Podistra_schoenherri 1 2.60 yes yes no 0 1 0 0 predator
2016 June R69 10 humus Araneae Theridiidae Robertus_sp 1 0.41 yes yes no 0 1 0 0 predator
2016 August R60 1 humus Annelida Lumbricidae Dendrobaena_octaedra 1 5.52 yes yes no 0 0 1 0 detritivore
2016 August R60 1 humus Annelida Lumbricidae Eiseniella_tetraedra 1 0.23 yes yes no 0 0 1 0 detritivore
2016 August R60 1 humus Annelida Lumbricidae Eiseniella_tetraedra 1 0.31 yes yes no 0 0 1 0 detritivore
2016 August R60 1 humus Annelida Lumbricidae Lumbricus_rubellus 1 80.79 yes yes no 0 0 1 0 detritivore
2016 August R60 1 humus Coleoptera Elateridae Athous_subfuscus 1 4.91 yes yes yes 0 0.8 0.1 0.1 combined
2016 August R60 1 humus Coleoptera Elateridae Dalopius_marginatus 1 1.43 yes yes yes 0 0.8 0.1 0.1 combined
2016 August R60 1 humus Hymenoptera Formicidae Myrmica_ruginodis 1 0.68 Yes Yes no 0 1 0 0 predator
2016 August R60 1 humus Myriapoda Lithobiidae Lithobius_curtipes 1 1.13 yes yes no 0 1 0 0 predator
2016 August R60 1 humus Myriapoda Lithobiidae Lithobius_curtipes 1 1.20 yes yes no 0 1 0 0 predator
2016 August R60 1 humus Myriapoda Lithobiidae Lithobius_curtipes 1 1.25 yes yes no 0 1 0 0 predator
2016 August R60 1 humus Myriapoda Lithobiidae Lithobius_curtipes 1 1.61 yes yes no 0 1 0 0 predator
2016 August R60 1 humus Myriapoda Lithobiidae Lithobius_forficatos 1 11.76 yes yes no 0 1 0 0 predator
2016 August R60 1 humus Diptera Rhagionidae Rhagio_lineola 1 0.29 yes yes no 0 1 0 0 predator
2016 August R60 1 humus Diptera Rhagionidae Rhagio_lineola 1 2.48 yes yes no 0 1 0 0 predator
2016 August R60 1 humus Coleoptera Staphylinidae Amischa_analis 1 0.05 yes yes no 0 1 0 0 predator
2016 August R60 1 humus Coleoptera Staphylinidae Geostiba_circellaris 1 0.10 yes yes no 0 1 0 0 predator
2016 August R60 1 humus Coleoptera Staphylinidae Habrocerus_capillaricornis 1 0.17 yes yes no 0 1 0 0 predator
2016 August R60 2 humus Annelida Lumbricidae Dendrobaena_octaedra 1 26.96 yes yes no 0 0 1 0 detritivore
2016 August R60 2 humus Annelida Lumbricidae Lumbricus_sp 1 0.83 yes yes no 0 0 1 0 detritivore
2016 August R60 2 humus Coleoptera Cantharidae Malthodes_fuscus 1 0.28 yes yes no 0 1 0 0 predator
2016 August R60 2 humus Hymenoptera Formicidae Myrmica_ruginodis 1 0.67 Yes Yes no 0 1 0 0 predator
2016 August R60 2 humus Araneae Linyphiidae Ceratinella_brevis 1 0.37 yes yes no 0 1 0 0 predator
2016 August R60 2 humus Coleoptera Staphylinidae Othius_subuliformis 1 0.49 yes yes no 0 1 0 0 predator
2016 August R60 3 humus Coleoptera Carabidae Pterostichus_diligens 1 1.92 yes yes no 0 1 0 0 predator
2016 August R60 3 humus Araneae Lycosidae Pirata_Piratula_sp 1 0.14 yes yes no 0 1 0 0 predator
2016 August R60 3 humus Coleoptera Staphylinidae Lathrobium_brunnipes 1 1.89 yes yes no 0 1 0 0 predator
2016 August R60 3 humus Coleoptera Staphylinidae Lathrobium_longulum 1 0.28 yes yes no 0 1 0 0 predator
2016 August R60 3 humus Coleoptera Staphylinidae Philhygra_sp 1 0.21 yes yes no 0 1 0 0 predator
2016 August R60 3 mineral Coleoptera Elateridae Athous_subfuscus 1 3.49 yes yes yes 0 0.8 0.1 0.1 combined
2016 August R60 3 mineral Araneae Lycosidae Pirata_Piratula_sp 1 5.06 yes yes no 0 1 0 0 predator
2016 August R60 4 humus Annelida Lumbricidae Dendrodrilus_rubidus_tenuis 1 6.12 yes yes no 0 0 1 0 detritivore
2016 August R60 4 humus Coleoptera Staphylinidae Tachinus_rufipes 1 1.56 yes yes no 0 0.5 0.5 0 combined
2016 August R60 4 humus Coleoptera Cantharidae Malthodes_fuscus 1 0.32 yes yes no 0 1 0 0 predator

2016 August R60 4 humus Hymenoptera Formicidae Myrmica_ruginodis 1 0.55 Yes Yes no 0 1 0 0 predator
2016 August R60 4 humus Araneae Linyphiidae Gen_sp 2 0.20 yes yes no 0 1 0 0 predator
2016 August R60 4 humus Araneae Linyphiidae Tapinocyba_pallens 2 4.94 yes yes no 0 1 0 0 predator
2016 August R60 4 humus Myriapoda Lithobiidae Lithobius_curtipes 1 1.39 yes yes no 0 1 0 0 predator
2016 August R60 4 humus Araneae Lycosidae Pirata_Piratula_sp 1 0.10 yes yes no 0 1 0 0 predator
2016 August R60 4 humus Coleoptera Staphylinidae Geostiba_circellaris 1 0.09 yes yes no 0 1 0 0 predator
2016 August R60 4 humus Coleoptera Staphylinidae Geostiba_circellaris 1 0.10 yes yes no 0 1 0 0 predator
2016 August R60 4 humus Coleoptera Staphylinidae Geostiba_circellaris 1 0.11 yes yes no 0 1 0 0 predator
2016 August R60 4 humus Coleoptera Staphylinidae Xantholinus_tricolor 1 2.01 yes yes no 0 1 0 0 predator
2016 August R60 4 humus Hemiptera Ortheziidae Newsteadia_floccosa 1 0.27 yes yes yes 1 0 0 0 herbivore
2016 August R60 4 humus Hemiptera Ortheziidae Newsteadia_floccosa 1 0.33 yes yes yes 1 0 0 0 herbivore
2016 August R60 4 mineral Coleoptera Elateridae Athous_subfuscus 1 3.49 yes yes yes 0 0.8 0.1 0.1 combined
2016 August R60 4 mineral Araneae Lycosidae Pirata_Piratula_sp 1 5.06 yes yes no 0 1 0 0 predator
2016 August R60 5 humus Coleoptera Hydrophilidae Cercyon_unipunctatus 1 0.71 yes yes no 0 0 1 0 detritivore
2016 August R60 5 humus Coleoptera Elateridae Athous_subfuscus 1 2.60 yes yes yes 0 0.8 0.1 0.1 combined
2016 August R60 5 humus Coleoptera Carabidae Notiophilus_germinyi 1 1.44 yes yes no 0 1 0 0 predator
2016 August R60 5 humus Coleoptera Carabidae Pterostichus_diligens 1 1.68 yes yes no 0 1 0 0 predator
2016 August R60 5 humus Hymenoptera Formicidae Myrmica_ruginodis 7 4.13 Yes Yes no 0 1 0 0 predator
2016 August R60 5 humus Araneae Hahniidae Hahnia_pusilla 2 0.35 yes yes no 0 1 0 0 predator
2016 August R60 5 humus Araneae Linyphiidae Gen_sp 1 0.14 yes yes no 0 1 0 0 predator
2016 August R60 5 humus Araneae Linyphiidae Tapinocyba_pallens 2 1.03 yes yes no 0 1 0 0 predator
2016 August R60 5 humus Araneae Lycosidae Pirata_Piratula_sp 3 0.27 yes yes no 0 1 0 0 predator
2016 August R60 5 humus Diptera Rhagionidae Rhagio_lineola 1 0.29 yes yes no 0 1 0 0 predator
2016 August R60 5 humus Coleoptera Staphylinidae Geostiba_circellaris 1 0.06 yes yes no 0 1 0 0 predator
2016 August R60 5 humus Coleoptera Staphylinidae Geostiba_circellaris 1 0.08 yes yes no 0 1 0 0 predator
2016 August R60 5 humus Coleoptera Staphylinidae Geostiba_circellaris 1 0.10 yes yes no 0 1 0 0 predator
2016 August R60 5 humus Coleoptera Staphylinidae Geostiba_circellaris 1 0.12 yes yes no 0 1 0 0 predator
2016 August R60 5 humus Coleoptera Staphylinidae Othius_punctulatus 1 2.72 yes yes no 0 1 0 0 predator
2016 August R60 5 humus Coleoptera Staphylinidae Xantholinus_tricolor 1 0.71 yes yes no 0 1 0 0 predator
2016 August R60 5 humus Lepidoptera Hepialidae Phymatopus_hecta 1 18.39 Yes Yes yes 1 0 0 0 herbivore
2016 August R60 6 humus Myriapoda Julidae Ommatoiulus_sabulosus 1 15.19 yes yes no 0 0 1 0 detritivore
2016 August R60 6 humus Annelida Lumbricidae Lumbricus_sp 1 0.42 yes yes no 0 0 1 0 detritivore
2016 August R60 6 humus Araneae Gnaphosidae Haplodrassus_sp 1 0.20 yes yes no 0 1 0 0 predator
2016 August R60 6 humus Araneae Linyphiidae Ceratinella_brevis 1 0.32 yes yes no 0 1 0 0 predator
2016 August R60 6 humus Myriapoda Lithobiidae Lithobius_sp 1 0.10 yes yes no 0 1 0 0 predator
2016 August R60 6 humus Araneae Lycosidae Pirata_Piratula_sp 1 0.25 yes yes no 0 1 0 0 predator
2016 August R60 6 humus Coleoptera Staphylinidae Mocyta_simulans 1 0.13 yes yes no 0 1 0 0 predator
2016 August R60 6 humus Coleoptera Staphylinidae Othius_subuliformis 1 0.40 yes yes no 0 1 0 0 predator
2016 August R60 6 humus Coleoptera Staphylinidae Xantholinus_tricolor 1 0.23 yes yes no 0 1 0 0 predator
2016 August R60 7 humus Annelida Lumbricidae Dendrodrilus_rubidus_tenuis 3 28.20 yes yes no 0 0 1 0 detritivore
2016 August R60 7 humus Annelida Lumbricidae Lumbricus_rubellus 3 134.42 yes yes no 0 0 1 0 detritivore
2016 August R60 7 humus Coleoptera Staphylinidae Tachinus_rufipes 1 1.86 yes yes no 0 0.5 0.5 0 combined
2016 August R60 7 humus Coleoptera Elateridae Athous_subfuscus 1 2.10 yes yes yes 0 0.8 0.1 0.1 combined
2016 August R60 7 humus Myriapoda Lithobiidae Lithobius_curtipes 1 1.25 yes yes no 0 1 0 0 predator
2016 August R60 7 humus Myriapoda Lithobiidae Lithobius_curtipes 1 1.34 yes yes no 0 1 0 0 predator
2016 August R60 7 humus Diptera Rhagionidae Rhagio_lineola 1 0.67 yes yes no 0 1 0 0 predator
2016 August R60 7 humus Diptera Rhagionidae Rhagio_lineola 1 0.97 yes yes no 0 1 0 0 predator
2016 August R60 7 humus Diptera Rhagionidae Rhagio_lineola 1 1.13 yes yes no 0 1 0 0 predator
2016 August R60 7 humus Diptera Rhagionidae Rhagio_lineola 1 1.93 yes yes no 0 1 0 0 predator
2016 August R60 7 humus Coleoptera Staphylinidae Acrotona_silvicola 1 0.10 yes yes no 0 1 0 0 predator
2016 August R60 7 humus Coleoptera Staphylinidae Geostiba_circellaris 1 0.10 yes yes no 0 1 0 0 predator
2016 August R60 7 humus Coleoptera Staphylinidae Mocyta_fungi 1 0.11 yes yes no 0 1 0 0 predator
2016 August R60 7 humus Coleoptera Staphylinidae Othius_punctulatus 1 2.28 yes yes no 0 1 0 0 predator

2016 August R60 7 humus Coleoptera Staphylinidae Othius_subuliformis 1 0.32 yes yes no 0 1 0 0 predator
2016 August R60 7 humus Coleoptera Staphylinidae Quedius_fuliginosus 1 5.35 yes yes no 0 1 0 0 predator
2016 August R60 7 humus Coleoptera Staphylinidae Xantholinus_tricolor 1 1.48 yes yes no 0 1 0 0 predator
2016 August R60 7 mineral Hymenoptera Formicidae Myrmica_ruginodis 1 0.48 Yes Yes no 0 1 0 0 predator
2016 August R60 7 mineral Coleoptera Elateridae Selatosomus_aeneus 1 2.48 yes yes yes 0.6 0.13 0.13 0.13 combined
2016 August R60 8 humus Annelida Lumbricidae Dendrodrilus_rubidus_tenuis 2 24.38 yes yes no 0 0 1 0 detritivore
2016 August R60 8 humus Annelida Lumbricidae Eiseniella_tetraedra 1 0.21 yes yes no 0 0 1 0 detritivore
2016 August R60 8 humus Annelida Lumbricidae Lumbricus_sp 1 8.19 yes yes no 0 0 1 0 detritivore
2016 August R60 8 humus Coleoptera Scarabaeidae Aphodius_niger 1 1.36 yes yes no 0 0 1 0 detritivore
2016 August R60 8 humus Coleoptera Elateridae Athous_subfuscus 1 0.17 yes yes yes 0 0.8 0.1 0.1 combined
2016 August R60 8 humus Coleoptera Elateridae Athous_subfuscus 1 0.21 yes yes yes 0 0.8 0.1 0.1 combined
2016 August R60 8 humus Coleoptera Cantharidae Rhagonycha_lignosa 1 0.19 yes yes no 0 1 0 0 predator
2016 August R60 8 humus Coleoptera Carabidae Notiophilus_palustris 1 1.67 yes yes no 0 1 0 0 predator
2016 August R60 8 humus Hymenoptera Formicidae Myrmica_ruginodis 18 10.64 Yes Yes no 0 1 0 0 predator
2016 August R60 8 humus Araneae Linyphiidae Tapinocyba_pallens 1 1.79 yes yes no 0 1 0 0 predator
2016 August R60 8 humus Araneae Liocranidae Gen_sp 2 1.95 yes yes no 0 1 0 0 predator
2016 August R60 8 humus Myriapoda Lithobiidae Lithobius_curtipes 1 0.38 yes yes no 0 1 0 0 predator
2016 August R60 8 humus Araneae Lycosidae Pirata_Piratula_sp 12 0.83 yes yes no 0 1 0 0 predator
2016 August R60 8 humus Araneae Phrurolithidae Phrurolithus_sp 1 0.63 yes yes no 0 1 0 0 predator
2016 August R60 8 humus Diptera Rhagionidae Rhagio_lineola 1 2.06 yes yes no 0 1 0 0 predator
2016 August R60 8 humus Coleoptera Staphylinidae Acrotona_silvicola 1 0.07 yes yes no 0 1 0 0 predator
2016 August R60 8 humus Coleoptera Staphylinidae Platydracus_latebricola 1 1.97 yes yes no 0 1 0 0 predator
2016 August R60 8 humus Coleoptera Elateridae Selatosomus_gloriosus 1 0.50 yes yes yes 0.6 0.13 0.13 0.13 combined
2016 August R60 8 mineral Hymenoptera Formicidae Myrmica_ruginodis 1 0.48 Yes Yes no 0 1 0 0 predator
2016 August R60 8 mineral Coleoptera Elateridae Selatosomus_aeneus 1 2.48 yes yes yes 0.6 0.13 0.13 0.13 combined
2016 August R60 9 humus Annelida Lumbricidae . 1 1.09 yes yes no 0 0 1 0 detritivore
2016 August R60 9 humus Annelida Lumbricidae Dendrobaena_octaedra 1 23.09 yes yes no 0 0 1 0 detritivore
2016 August R60 9 humus Annelida Lumbricidae Dendrodrilus_rubidus_tenuis 1 6.92 yes yes no 0 0 1 0 detritivore
2016 August R60 9 humus Coleoptera Elateridae Ampedus_cf_balteatus 1 8.48 yes yes no 0 0.5 0.5 0 combined
2016 August R60 9 humus Coleoptera Carabidae Pterostichus_rhaeticus 1 6.75 yes yes no 0 1 0 0 predator
2016 August R60 9 humus Myriapoda Lithobiidae Lithobius_curtipes 1 1.34 yes yes no 0 1 0 0 predator
2016 August R60 9 humus Myriapoda Lithobiidae Lithobius_sp 1 0.10 yes yes no 0 1 0 0 predator
2016 August R60 9 humus Coleoptera Staphylinidae Brachygluta_fossulata 1 0.09 yes yes no 0 1 0 0 predator
2016 August R60 9 humus Coleoptera Staphylinidae Xantholinus_tricolor 1 0.24 yes yes no 0 1 0 0 predator
2016 August R60 9 humus Coleoptera Staphylinidae Xantholinus_tricolor 1 1.55 yes yes no 0 1 0 0 predator
2016 August R60 9 humus Hymenoptera Formicidae Lasius_platythorax 417 163.07 Yes Yes no 0.8 0.2 0 0 combined
2016 August R60 9 humus Coleoptera Curculionidae Hylobius_abietis 1 30.23 yes yes yes 1 0 0 0 herbivore
2016 August R60 9 humus Lepidoptera Hepialidae Phymatopus_hecta 1 3.33 Yes Yes yes 1 0 0 0 herbivore
2016 August R60 9 humus Hemiptera Ortheziidae Newsteadia_floccosa 1 0.08 yes yes yes 1 0 0 0 herbivore
2016 August R60 9 humus Hemiptera Ortheziidae Newsteadia_floccosa 1 0.09 yes yes yes 1 0 0 0 herbivore
2016 August R60 9 humus Hemiptera Ortheziidae Newsteadia_floccosa 1 0.13 yes yes yes 1 0 0 0 herbivore
2016 August R60 9 humus Myriapoda . . 1 0.10 yes yes no . . . . .
2016 August R60 9 humus Myriapoda . . 1 0.55 yes yes no . . . . .
2016 August R60 10 humus Blattoptera Ectobiidae Ectobius_sylvestris 1 1.59 yes yes no 0 0 1 0 detritivore
2016 August R60 10 humus Annelida Lumbricidae Dendrobaena_octaedra 1 21.18 yes yes no 0 0 1 0 detritivore
2016 August R60 10 humus Annelida Lumbricidae Eiseniella_tetraedra 1 0.52 yes yes no 0 0 1 0 detritivore
2016 August R60 10 humus Coleoptera Elateridae Dalopius_marginatus 1 0.97 yes yes yes 0 0.8 0.1 0.1 combined
2016 August R60 10 humus Hymenoptera Formicidae Myrmica_ruginodis 3 1.75 Yes Yes no 0 1 0 0 predator
2016 August R60 10 humus Araneae Hahniidae Hahnia_pusilla 1 0.21 yes yes no 0 1 0 0 predator
2016 August R60 10 humus Araneae Linyphiidae Tapinocyba_pallens 1 0.10 yes yes no 0 1 0 0 predator
2016 August R60 10 humus Myriapoda Lithobiidae Lithobius_curtipes 1 2.23 yes yes no 0 1 0 0 predator
2016 August R60 10 humus Myriapoda Lithobiidae Lithobius_curtipes 1 2.38 yes yes no 0 1 0 0 predator
2016 August R60 10 humus Diptera Rhagionidae Rhagio_lineola 1 1.93 yes yes no 0 1 0 0 predator

2016 August R60 10 humus Coleoptera Staphylinidae Mocyta_simulans 1 0.18 yes yes no 0 1 0 0 predator
2016 August R60 10 humus Coleoptera Staphylinidae Xantholinus_tricolor 1 2.07 yes yes no 0 1 0 0 predator
2016 August R60 10 humus Coleoptera Elateridae Paraphotistus_impressus 1 3.44 yes yes yes 0.6 0.13 0.13 0.13 combined
2016 August R60 10 humus Hymenoptera Formicidae Lasius_platythorax 1 0.39 Yes Yes no 0.8 0.2 0 0 combined
2016 August R60 10 humus Coleoptera Curculionidae Sciaphilus_asperatus 1 4.45 yes yes yes 1 0 0 0 herbivore
2016 August R61 1 humus Coleoptera Elateridae Ampedus_cf_balteatus 1 1.89 yes yes no 0 0.5 0.5 0 combined
2016 August R61 1 humus Hymenoptera Formicidae Leptothorax_acervorum 1 0.28 Yes Yes no 0 0.8 0.2 0 combined
2016 August R61 1 humus Araneae Hahniidae Hahnia_pusilla 2 0.20 yes yes no 0 1 0 0 predator
2016 August R61 1 humus Araneae Salticidae Gen_sp 1 0.32 yes yes no 0 1 0 0 predator
2016 August R61 1 humus Coleoptera Staphylinidae Othius_subuliformis 1 0.26 yes yes no 0 1 0 0 predator
2016 August R61 1 humus Araneae Theridiidae Robertus_sp 1 0.13 yes yes no 0 1 0 0 predator
2016 August R61 1 humus Coleoptera Elateridae Paraphotistus_impressus 1 0.76 yes yes yes 0.6 0.13 0.13 0.13 combined
2016 August R61 1 humus Hemiptera Ortheziidae Newsteadia_floccosa 1 0.01 yes yes yes 1 0 0 0 herbivore
2016 August R61 1 humus Hemiptera Ortheziidae Newsteadia_floccosa 1 0.08 yes yes yes 1 0 0 0 herbivore
2016 August R61 1 mineral Diptera Asilidae Neoitamus_socius 1 10.46 yes yes no 0 1 0 0 predator
2016 August R61 1 mineral Coleoptera Curculionidae Strophosoma_capitatum 1 6.00 yes yes yes 1 0 0 0 herbivore
2016 August R61 2 humus Hymenoptera Formicidae Leptothorax_acervorum 23 4.30 Yes Yes no 0 0.8 0.2 0 combined
2016 August R61 2 humus Coleoptera Staphylinidae . 1 0.08 yes yes no 0 1 0 0 predator
2016 August R61 2 humus Coleoptera Staphylinidae Xantholinus_distans 1 0.18 yes yes no 0 1 0 0 predator
2016 August R61 2 humus Coleoptera Elateridae Paraphotistus_impressus 1 26.71 yes yes yes 0.6 0.13 0.13 0.13 combined
2016 August R61 2 humus Hemiptera Lygaeidae Eremocoris_abietis 1 1.46 yes no no 1 0 0 0 herbivore
2016 August R61 2 humus Hemiptera Lygaeidae Eremocoris_plebejus 1 1.31 yes no no 1 0 0 0 herbivore
2016 August R61 2 humus Hymenoptera Pamphilidae Cephalcia_intermedia 1 11.26 Yes no no 1 0 0 0 herbivore
2016 August R61 2 humus Hemiptera Tingidae Acalypta_parvula 1 0.10 yes yes no 1 0 0 0 herbivore
2016 August R61 2 mineral Diptera Asilidae Neoitamus_socius 1 10.46 yes yes no 0 1 0 0 predator
2016 August R61 2 mineral Coleoptera Curculionidae Strophosoma_capitatum 1 6.00 yes yes yes 1 0 0 0 herbivore
2016 August R61 3 humus Araneae Linyphiidae Cnephalocotes_obscurus 1 0.17 yes yes no 0 1 0 0 predator
2016 August R61 3 humus Araneae Linyphiidae Tapinocyba_pallens 1 0.09 yes yes no 0 1 0 0 predator
2016 August R61 3 humus Diptera Rhagionidae Rhagio_lineola 1 1.39 yes yes no 0 1 0 0 predator
2016 August R61 4 humus Annelida Lumbricidae Dendrobaena_octaedra 1 3.77 yes yes no 0 0 1 0 detritivore
2016 August R61 4 humus Coleoptera Cantharidae Malthodes_fuscus 1 0.22 yes yes no 0 1 0 0 predator
2016 August R61 4 humus Coleoptera Carabidae Calathus_micropterus 1 0.29 yes yes no 0 1 0 0 predator
2016 August R61 4 humus Araneae Hahniidae Hahnia_ononidum 1 0.26 yes yes no 0 1 0 0 predator
2016 August R61 4 humus Araneae Linyphiidae Ceratinella_brevis 1 0.08 yes yes no 0 1 0 0 predator
2016 August R61 4 humus Araneae Linyphiidae Gen_sp 1 0.27 yes yes no 0 1 0 0 predator
2016 August R61 4 humus Myriapoda Lithobiidae Lithobius_curtipes 1 0.77 yes yes no 0 1 0 0 predator
2016 August R61 4 humus Araneae Theridiidae Robertus_sp 1 0.29 yes yes no 0 1 0 0 predator
2016 August R61 5 humus Annelida Lumbricidae Eiseniella_tetraedra 1 0.10 yes yes no 0 0 1 0 detritivore
2016 August R61 5 humus Coleoptera Elateridae Athous_subfuscus 1 0.21 yes yes yes 0 0.8 0.1 0.1 combined
2016 August R61 5 humus Coleoptera Elateridae Dalopius_marginatus 1 0.13 yes yes yes 0 0.8 0.1 0.1 combined
2016 August R61 5 humus Coleoptera Staphylinidae Atheta_myrmecobia 1 0.06 yes yes no 0 1 0 0 predator
2016 August R61 6 humus Coleoptera Elateridae Dalopius_marginatus 1 1.76 yes yes yes 0 0.8 0.1 0.1 combined
2016 August R61 6 humus Coleoptera Elateridae Dalopius_marginatus 1 3.99 yes yes yes 0 0.8 0.1 0.1 combined
2016 August R61 6 humus Araneae Hahniidae Hahnia_pusilla 1 0.20 yes yes no 0 1 0 0 predator
2016 August R61 6 humus Araneae Hahniidae Hahnia_sp 1 0.66 yes yes no 0 1 0 0 predator
2016 August R61 6 humus Araneae Lycosidae Gen_sp 1 0.05 yes yes no 0 1 0 0 predator
2016 August R61 6 humus Diptera Rhagionidae Rhagio_lineola 1 2.27 yes yes no 0 1 0 0 predator
2016 August R61 6 humus Coleoptera Staphylinidae Philonthus_carbonarius 1 0.99 yes yes no 0 1 0 0 predator
2016 August R61 6 humus Coleoptera Staphylinidae Philonthus_carbonarius 1 1.13 yes yes no 0 1 0 0 predator
2016 August R61 6 humus Coleoptera Staphylinidae Philonthus_carbonarius 1 1.55 yes yes no 0 1 0 0 predator
2016 August R61 6 humus Coleoptera Staphylinidae Philonthus_carbonarius 1 1.68 yes yes no 0 1 0 0 predator
2016 August R61 6 humus Coleoptera Staphylinidae Philonthus_carbonarius 1 1.82 yes yes no 0 1 0 0 predator
2016 August R61 6 humus Araneae Theridiidae Robertus_scoticus 1 0.30 yes yes no 0 1 0 0 predator

2016 August R61 6 humus Hemiptera Lygaeidae Eremocoris_plebejus 1 1.71 yes no no 1 0 0 0 herbivore
2016 August R61 6 mineral Coleoptera Staphylinidae Philonthus_carbonarius 1 1.30 yes yes no 0 1 0 0 predator
2016 August R61 6 mineral Coleoptera Staphylinidae Philonthus_carbonarius 1 1.30 yes yes no 0 1 0 0 predator
2016 August R61 7 humus Coleoptera Cantharidae Malthodes_fuscus 1 0.11 yes yes no 0 1 0 0 predator
2016 August R61 7 humus Araneae Linyphiidae Macrargus_rufus 1 0.05 yes yes no 0 1 0 0 predator
2016 August R61 7 humus Araneae Theridiidae Robertus_sp 1 1.36 yes yes no 0 1 0 0 predator
2016 August R61 8 humus Coleoptera Elateridae Athous_subfuscus 1 1.89 yes yes yes 0 0.8 0.1 0.1 combined
2016 August R61 8 humus Coleoptera Elateridae Dalopius_marginatus 1 0.42 yes yes yes 0 0.8 0.1 0.1 combined
2016 August R61 8 humus Araneae Lycosidae Trochosa_terricola 1 30.44 yes yes no 0 1 0 0 predator
2016 August R61 8 humus Coleoptera Staphylinidae Ochthephilum_fracticorne 1 0.42 yes yes no 0 1 0 0 predator
2016 August R61 8 humus Coleoptera Staphylinidae Ochthephilum_fracticorne 1 0.58 yes yes no 0 1 0 0 predator
2016 August R61 8 humus Coleoptera Elateridae Paraphotistus_impressus 1 35.45 yes yes yes 0.6 0.13 0.13 0.13 combined
2016 August R61 9 humus Blattoptera Ectobiidae Ectobius_sylvestris 1 0.43 yes yes no 0 0 1 0 detritivore
2016 August R61 9 humus Coleoptera Elateridae Ampedus_nigrinus 1 1.05 yes yes no 0 0.5 0.5 0 combined
2016 August R61 9 humus Hymenoptera Formicidae Myrmica_ruginodis 1 0.47 Yes Yes no 0 1 0 0 predator
2016 August R61 9 humus Araneae Hahniidae Hahnia_pusilla 1 0.19 yes yes no 0 1 0 0 predator
2016 August R61 9 humus Araneae Linyphiidae Gen_sp 1 0.06 yes yes no 0 1 0 0 predator
2016 August R61 9 humus Myriapoda Lithobiidae Lithobius_cf_curtipes 1 0.19 yes yes no 0 1 0 0 predator
2016 August R61 9 humus Diptera Rhagionidae Rhagio_lineola 1 2.44 yes yes no 0 1 0 0 predator
2016 August R61 9 humus Coleoptera Staphylinidae Philonthus_carbonarius 1 1.43 yes yes no 0 1 0 0 predator
2016 August R61 9 humus Hemiptera Lygaeidae Eremocoris_sp 1 0.29 yes no no 1 0 0 0 herbivore
2016 August R61 10 humus Hemiptera Ceratocombidae Ceratocombus_brevipennis 1 0.03 yes yes no 0 1 0 0 predator
2016 August R61 10 humus Araneae Hahniidae Hahnia_pusilla 1 0.15 yes yes no 0 1 0 0 predator
2016 August R61 10 humus Araneae Linyphiidae Gen_sp 1 0.21 yes yes no 0 1 0 0 predator
2016 August R61 10 humus Araneae Linyphiidae Macrargus_multesimus 2 1.05 yes yes no 0 1 0 0 predator
2016 August R61 10 humus Diptera Rhagionidae Rhagio_lineola 1 0.08 yes yes no 0 1 0 0 predator
2016 August R61 10 humus Coleoptera Staphylinidae Mocyta_fungi 1 0.09 yes yes no 0 1 0 0 predator
2016 August R61 10 humus Coleoptera Curculionidae Otiorhynchus_nodosus 1 0.17 yes yes yes 1 0 0 0 herbivore
2016 August R62 1 humus Coleoptera Cantharidae Malthodes_brevicollis 1 0.26 yes yes no 0 1 0 0 predator
2016 August R62 1 humus Hymenoptera Formicidae Myrmica_ruginodis 1 0.57 Yes Yes no 0 1 0 0 predator
2016 August R62 1 humus Myriapoda Lithobiidae Lithobius_curtipes 1 2.28 yes yes no 0 1 0 0 predator
2016 August R62 1 humus Coleoptera Staphylinidae Atheta_subtilis 1 0.02 yes yes no 0 1 0 0 predator
2016 August R62 1 humus Coleoptera Staphylinidae Atheta_subtilis 1 0.03 yes yes no 0 1 0 0 predator
2016 August R62 1 humus Coleoptera Staphylinidae Gabrius_trossulus 1 0.23 yes yes no 0 1 0 0 predator
2016 August R62 1 humus Coleoptera Staphylinidae Philonthus_carbonarius 1 1.74 yes yes no 0 1 0 0 predator
2016 August R62 1 humus Coleoptera Staphylinidae Quedius_curtipennis 1 5.71 yes yes no 0 1 0 0 predator
2016 August R62 1 humus Coleoptera Staphylinidae Quedius_sp 1 0.39 yes yes no 0 1 0 0 predator
2016 August R62 2 humus Araneae Linyphiidae Gen_sp 1 0.04 yes yes no 0 1 0 0 predator
2016 August R62 2 humus Araneae Linyphiidae Maso_sp 1 0.08 yes yes no 0 1 0 0 predator
2016 August R62 2 humus Araneae Linyphiidae Minyriolus_pusillus 1 0.06 yes yes no 0 1 0 0 predator
2016 August R62 2 humus Araneae Linyphiidae Porrhomma_pallidum 1 0.10 yes yes no 0 1 0 0 predator
2016 August R62 2 humus Araneae Linyphiidae Tapinocyba_pallens 1 0.12 yes yes no 0 1 0 0 predator
2016 August R62 2 humus Myriapoda Lithobiidae Lithobius_curtipes 1 0.65 yes yes no 0 1 0 0 predator
2016 August R62 2 humus Coleoptera Staphylinidae Othius_punctulatus 1 2.77 yes yes no 0 1 0 0 predator
2016 August R62 2 humus Coleoptera Staphylinidae Philhygra_hygrobia 1 0.08 yes yes no 0 1 0 0 predator
2016 August R62 2 humus Araneae Theridiidae Robertus_sp 1 0.09 yes yes no 0 1 0 0 predator
2016 August R62 2 humus Diptera Chironomidae . 1 0.04 yes yes yes 0.25 0 0.75 0 combined
2016 August R62 3 humus Coleoptera Hydrophilidae Cryptopleurum_minutum 1 0.26 yes yes no 0 0 1 0 detritivore
2016 August R62 3 humus Annelida Lumbricidae Dendrobaena_octaedra 3 6.50 yes yes no 0 0 1 0 detritivore
2016 August R62 3 humus Annelida Lumbricidae Lumbricus_sp 2 106.82 yes yes no 0 0 1 0 detritivore
2016 August R62 3 humus Araneae Linyphiidae Tapinocyba_pallens 2 1.70 yes yes no 0 1 0 0 predator
2016 August R62 3 humus Araneae Linyphiidae Tapinocyba_pallens 2 12.20 yes yes no 0 1 0 0 predator
2016 August R62 3 humus Araneae Lycosidae Trochosa_sp 1 0.30 yes yes no 0 1 0 0 predator

2016 August R62 3 humus Coleoptera Staphylinidae Zyras_collaris 1 0.71 yes yes no 0 1 0 0 predator
2016 August R62 3 mineral Coleoptera Elateridae Athous_subfuscus 1 3.65 yes yes yes 0 0.8 0.1 0.1 combined
2016 August R62 4 humus Myriapoda Polydesmidae Polydesmus_denticulatus 1 2.09 yes yes no 0 0 1 0 detritivore
2016 August R62 4 humus Coleoptera Elateridae Dalopius_marginatus 1 3.78 yes yes yes 0 0.8 0.1 0.1 combined
2016 August R62 4 humus Coleoptera Carabidae Calathus_micropterus 1 3.89 yes yes no 0 1 0 0 predator
2016 August R62 4 humus Hymenoptera Formicidae Myrmica_ruginodis 1 0.61 Yes Yes no 0 1 0 0 predator
2016 August R62 4 humus Araneae Linyphiidae Gen_sp 2 0.34 yes yes no 0 1 0 0 predator
2016 August R62 4 humus Myriapoda Lithobiidae Lithobius_cf_curtipes 1 1.32 yes yes no 0 1 0 0 predator
2016 August R62 4 humus Myriapoda Lithobiidae Lithobius_curtipes 1 1.37 yes yes no 0 1 0 0 predator
2016 August R62 4 mineral Coleoptera Elateridae Athous_subfuscus 1 3.65 yes yes yes 0 0.8 0.1 0.1 combined
2016 August R62 5 humus Coleoptera Staphylinidae Sepedophilus_pedicularius 1 0.17 yes yes no 0 0 0 1 fungivore
2016 August R62 5 humus Annelida Lumbricidae . 3 73.85 yes yes no 0 0 1 0 detritivore
2016 August R62 5 humus Annelida Lumbricidae Dendrodrilus_rubidus_tenuis 3 38.38 yes yes no 0 0 1 0 detritivore
2016 August R62 5 humus Annelida Lumbricidae Lumbricus_rubellus 2 170.90 yes yes no 0 0 1 0 detritivore
2016 August R62 5 humus Myriapoda Polydesmidae Polydesmus_denticulatus 1 3.67 yes yes no 0 0 1 0 detritivore
2016 August R62 5 humus Diptera Rhagionidae Rhagio_tringarius 1 1.01 yes yes no 0 1 0 0 predator
2016 August R62 5 humus Diptera Rhagionidae Rhagio_tringarius 1 10.42 yes yes no 0 1 0 0 predator
2016 August R62 5 humus Araneae Theridiidae Robertus_sp 1 0.08 yes yes no 0 1 0 0 predator
2016 August R62 5 humus Coleoptera . . 1 0.09 . . . .
2016 August R62 5 mineral Coleoptera Staphylinidae Quedius_fuliginosus 1 6.18 yes yes no 0 1 0 0 predator
2016 August R62 6 humus Annelida Lumbricidae . 1 1.25 yes yes no 0 0 1 0 detritivore
2016 August R62 6 humus Annelida Lumbricidae Dendrobaena_octaedra 1 7.57 yes yes no 0 0 1 0 detritivore
2016 August R62 6 humus Annelida Lumbricidae Dendrodrilus_rubidus_tenuis 4 31.49 yes yes no 0 0 1 0 detritivore
2016 August R62 6 humus Annelida Lumbricidae Eiseniella_tetraedra 1 0.83 yes yes no 0 0 1 0 detritivore
2016 August R62 6 mineral Coleoptera Staphylinidae Quedius_fuliginosus 1 6.18 yes yes no 0 1 0 0 predator
2016 August R62 6 mineral Coleoptera Scarabaeidae Melolontha_melolontha 1 610.47 yes yes yes 1 0 0 0 herbivore
2016 August R62 7 humus Annelida Lumbricidae Dendrobaena_octaedra 2 39.14 yes yes no 0 0 1 0 detritivore
2016 August R62 7 humus Hymenoptera Formicidae Myrmica_ruginodis 1 0.53 Yes Yes no 0 1 0 0 predator
2016 August R62 7 humus Araneae Linyphiidae Microneta_viaria 1 0.51 yes yes no 0 1 0 0 predator
2016 August R62 7 humus Coleoptera Staphylinidae Mocyta_fungi 1 0.08 yes yes no 0 1 0 0 predator
2016 August R62 7 mineral Myriapoda Julidae Ommatoiulus_sabulosus 1 29.50 yes yes no 0 0 1 0 detritivore
2016 August R62 8 humus Annelida Lumbricidae . 1 1.56 yes yes no 0 0 1 0 detritivore
2016 August R62 8 humus Annelida Lumbricidae Dendrobaena_octaedra 2 11.71 yes yes no 0 0 1 0 detritivore
2016 August R62 8 humus Annelida Lumbricidae Eiseniella_tetraedra 1 0.73 yes yes no 0 0 1 0 detritivore
2016 August R62 8 humus Diptera Tipulidae Tipula_vernalis 1 8.15 yes yes no 0 0 1 0 detritivore
2016 August R62 8 humus Coleoptera Carabidae Calathus_micropterus 1 2.62 yes yes no 0 1 0 0 predator
2016 August R62 8 humus Araneae Linyphiidae Tapinopa_longidens 1 1.32 yes yes no 0 1 0 0 predator
2016 August R62 8 humus Coleoptera Staphylinidae Lathrobium_brunnipes 1 1.81 yes yes no 0 1 0 0 predator
2016 August R62 8 humus Coleoptera Staphylinidae Quedius_fuliginosus 1 5.47 yes yes no 0 1 0 0 predator
2016 August R62 8 humus Lepidoptera Hepialidae Korscheltellus_fusconebulosa 1 34.78 Yes Yes yes 1 0 0 0 herbivore
2016 August R62 8 mineral Myriapoda Julidae Ommatoiulus_sabulosus 1 29.50 yes yes no 0 0 1 0 detritivore
2016 August R62 9 humus Myriapoda Polydesmidae Polydesmus_denticulatus 1 2.28 yes yes no 0 0 1 0 detritivore
2016 August R62 9 humus Coleoptera Elateridae Dalopius_marginatus 1 2.23 yes yes yes 0 0.8 0.1 0.1 combined
2016 August R62 9 humus Coleoptera Cantharidae . 1 0.10 yes yes no 0 1 0 0 predator
2016 August R62 9 humus Coleoptera Cantharidae Malthodes_mysticus 1 0.34 yes yes no 0 1 0 0 predator
2016 August R62 9 humus Araneae Clubionidae Clubiona_sp 1 0.18 yes yes no 0 1 0 0 predator
2016 August R62 9 humus Hymenoptera Formicidae Myrmica_ruginodis 5 2.69 Yes Yes no 0 1 0 0 predator
2016 August R62 9 humus Araneae Linyphiidae Maso_sundevalli 1 0.21 yes yes no 0 1 0 0 predator
2016 August R62 9 humus Araneae Linyphiidae Tapinocyba_pallens 1 3.99 yes yes no 0 1 0 0 predator
2016 August R62 9 humus Diptera Rhagionidae Rhagio_tringarius 1 13.48 yes yes no 0 1 0 0 predator
2016 August R62 9 humus Coleoptera Staphylinidae Aleochara_brevipennis 1 1.09 yes yes no 0 1 0 0 predator
2016 August R62 9 humus Coleoptera Staphylinidae Oxypoda_annularis 1 0.07 yes yes no 0 1 0 0 predator
2016 August R62 9 humus Coleoptera Curculionidae Sciaphilus_asperatus 1 4.31 yes yes yes 1 0 0 0 herbivore

2016 August R62 9 humus Hemiptera Lygaeidae Drymus_brunneus 1 1.05 yes yes no 1 0 0 0 herbivore
2016 August R62 9 mineral Coleoptera Staphylinidae Mocyta_fungi 1 0.07 yes yes no 0 1 0 0 predator
2016 August R62 9 mineral Coleoptera Elateridae Paraphotistus_impressus 1 4.87 yes yes yes 0.6 0.13 0.13 0.13 combined
2016 August R62 10 humus Coleoptera Leiodidae Agathidium_confusum 1 0.57 yes yes no 0 0 0 1 fungivore
2016 August R62 10 humus Annelida Lumbricidae Dendrobaena_octaedra 1 6.55 yes yes no 0 0 1 0 detritivore
2016 August R62 10 humus Coleoptera Staphylinidae Tachinus_laticollis 1 0.57 yes yes no 0 0.5 0.5 0 combined
2016 August R62 10 humus Araneae Agelenidae Gen_sp 1 0.09 yes yes no 0 1 0 0 predator
2016 August R62 10 humus Araneae Linyphiidae Tapinocyba_pallens 1 0.58 yes yes no 0 1 0 0 predator
2016 August R62 10 humus Araneae Linyphiidae Walckenaeria_dysderoides 1 0.21 yes yes no 0 1 0 0 predator
2016 August R62 10 humus Myriapoda Lithobiidae Lithobius_curtipes 1 0.89 yes yes no 0 1 0 0 predator
2016 August R62 10 humus Araneae Salticidae Gen_sp 1 0.11 yes yes no 0 1 0 0 predator
2016 August R62 10 humus Coleoptera Staphylinidae Ischnosoma_splendidum 1 0.34 yes yes no 0 1 0 0 predator
2016 August R62 10 humus Coleoptera Staphylinidae Mocyta_fungi 1 0.08 yes yes no 0 1 0 0 predator
2016 August R62 10 humus Coleoptera Staphylinidae Quedius_molochinus 1 0.55 yes yes no 0 1 0 0 predator
2016 August R62 10 humus Coleoptera Staphylinidae Xantholinus_tricolor 1 1.98 yes yes no 0 1 0 0 predator
2016 August R62 10 humus Lepidoptera Hepialidae Phymatopus_hecta 1 3.78 Yes Yes yes 1 0 0 0 herbivore
2016 August R62 10 mineral Coleoptera Staphylinidae Mocyta_fungi 1 0.07 yes yes no 0 1 0 0 predator
2016 August R62 10 mineral Coleoptera Elateridae Paraphotistus_impressus 1 4.87 yes yes yes 0.6 0.13 0.13 0.13 combined
2016 August R63 1 humus Coleoptera Staphylinidae Tachyporus_quadriscopulatus 1 0.16 yes yes no 0 0.5 0.5 0 combined
2016 August R63 1 humus Coleoptera Staphylinidae Acrotona_silvicola 1 0.10 yes yes no 0 1 0 0 predator
2016 August R63 1 humus Coleoptera Staphylinidae Bisnius_sp 1 0.51 yes yes no 0 1 0 0 predator
2016 August R63 1 humus Coleoptera Staphylinidae Geostiba_circellaris 1 0.12 yes yes no 0 1 0 0 predator
2016 August R63 1 humus Coleoptera Staphylinidae Xantholinus_distans 1 0.41 yes yes no 0 1 0 0 predator
2016 August R63 1 mineral Coleoptera Staphylinidae . 1 1.08 yes yes no 0 1 0 0 predator
2016 August R63 1 mineral Hymenoptera Formicidae Lasius_platythorax 1 0.40 Yes Yes no 0.8 0.2 0 0 combined
2016 August R63 2 humus Hymenoptera Formicidae Myrmica_ruginodis 1 1.09 Yes Yes no 0 1 0 0 predator
2016 August R63 2 humus Araneae Linyphiidae Gen_sp 1 0.12 yes yes no 0 1 0 0 predator
2016 August R63 2 humus Araneae Linyphiidae Macrargus_rufus 1 2.19 yes yes no 0 1 0 0 predator
2016 August R63 2 humus Myriapoda Lithobiidae Lithobius_curtipes 1 1.25 yes yes no 0 1 0 0 predator
2016 August R63 2 humus Myriapoda Lithobiidae Lithobius_curtipes 1 1.63 yes yes no 0 1 0 0 predator
2016 August R63 2 humus Araneae Miturgidae Zora_spinimana 1 2.25 yes yes no 0 1 0 0 predator
2016 August R63 2 humus Diptera Rhagionidae Rhagio_lineola 1 3.74 yes yes no 0 1 0 0 predator
2016 August R63 2 humus Coleoptera Staphylinidae Stenus_clavicornis 1 0.73 yes yes no 0 1 0 0 predator
2016 August R63 2 humus Coleoptera Staphylinidae Xantholinus_distans 1 0.34 yes yes no 0 1 0 0 predator
2016 August R63 2 mineral Coleoptera Staphylinidae . 1 1.08 yes yes no 0 1 0 0 predator
2016 August R63 2 mineral Hymenoptera Formicidae Lasius_platythorax 1 0.40 Yes Yes no 0.8 0.2 0 0 combined
2016 August R63 3 humus Araneae Linyphiidae Centromerus_arcanus 1 1.11 yes yes no 0 1 0 0 predator
2016 August R63 3 humus Araneae Linyphiidae Gen_sp 1 0.11 yes yes no 0 1 0 0 predator
2016 August R63 3 humus Araneae Linyphiidae Minyriolus_pusillus 1 0.15 yes yes no 0 1 0 0 predator
2016 August R63 3 humus Araneae Theridiidae Robertus_scoticus 1 0.24 yes yes no 0 1 0 0 predator
2016 August R63 3 humus Araneae Theridiidae Robertus_sp 2 0.20 yes yes no 0 1 0 0 predator
2016 August R63 4 humus Hymenoptera Formicidae Myrmica_ruginodis 2 2.27 Yes Yes no 0 1 0 0 predator
2016 August R63 4 humus Coleoptera Staphylinidae Xantholinus_distans 1 0.23 yes yes no 0 1 0 0 predator
2016 August R63 5 humus Coleoptera Elateridae Athous_subfuscus 1 1.22 yes yes yes 0 0.8 0.1 0.1 combined
2016 August R63 5 humus Araneae Linyphiidae Gen_sp 1 0.19 yes yes no 0 1 0 0 predator
2016 August R63 5 humus Myriapoda Lithobiidae Lithobius_curtipes 1 1.68 yes yes no 0 1 0 0 predator
2016 August R63 5 humus Araneae Lycosidae Pardosa_sp 1 0.42 yes yes no 0 1 0 0 predator
2016 August R63 5 humus Diptera Rhagionidae Rhagio_lineola 1 1.09 yes yes no 0 1 0 0 predator
2016 August R63 5 humus Coleoptera Staphylinidae Geostiba_circellaris 1 0.10 yes yes no 0 1 0 0 predator
2016 August R63 5 humus Coleoptera Elateridae Selatosomus_aeneus 1 1.18 yes yes yes 0.6 0.13 0.13 0.13 combined
2016 August R63 5 humus Hemiptera Cydnidae Adomerus_biguttatus 1 2.94 yes yes yes 1 0 0 0 herbivore
2016 August R63 5 humus Hemiptera Cydnidae Adomerus_biguttatus 1 3.45 yes yes yes 1 0 0 0 herbivore
2016 August R63 5 humus Hemiptera Cydnidae Adomerus_biguttatus 1 5.00 yes yes yes 1 0 0 0 herbivore

2016 August R63 5 humus Hemiptera Cydnidae Adomerus_biguttatus 1 5.82 yes yes yes 1 0 0 0 herbivore
2016 August R63 5 humus Hemiptera Cydnidae Adomerus_biguttatus 1 6.13 yes yes yes 1 0 0 0 herbivore
2016 August R63 5 mineral Araneae Linyphiidae Centromerus_incilium 1 0.41 yes yes no 0 1 0 0 predator
2016 August R63 6 humus Annelida Lumbricidae Dendrodrilus_rubidus_tenuis 2 41.50 yes yes no 0 0 1 0 detritivore
2016 August R63 6 humus Araneae Linyphiidae Centromerus_arcanus 1 0.13 yes yes no 0 1 0 0 predator
2016 August R63 6 humus Araneae Linyphiidae Tapinocyba_pallens 1 0.18 yes yes no 0 1 0 0 predator
2016 August R63 6 humus Coleoptera Staphylinidae . 1 0.65 yes yes no 0 1 0 0 predator
2016 August R63 6 mineral Araneae Linyphiidae Centromerus_incilium 1 0.41 yes yes no 0 1 0 0 predator
2016 August R63 7 humus Annelida Lumbricidae Dendrobaena_octaedra 2 51.31 yes yes no 0 0 1 0 detritivore
2016 August R63 7 humus Araneae Linyphiidae Centromerus_incilium 1 0.37 yes yes no 0 1 0 0 predator
2016 August R63 7 humus Diptera Rhagionidae Rhagio_lineola 1 0.63 yes yes no 0 1 0 0 predator
2016 August R63 7 humus Coleoptera Staphylinidae Geostiba_circellaris 1 0.08 yes yes no 0 1 0 0 predator
2016 August R63 7 humus Coleoptera Staphylinidae Xantholinus_tricolor 1 1.24 yes yes no 0 1 0 0 predator
2016 August R63 7 humus Coleoptera Elateridae Paraphotistus_impressus 1 2.90 yes yes yes 0.6 0.13 0.13 0.13 combined
2016 August R63 7 humus Hemiptera Cydnidae Adomerus_biguttatus 1 6.71 yes yes yes 1 0 0 0 herbivore
2016 August R63 8 humus Annelida Lumbricidae Dendrobaena_octaedra 1 7.38 yes yes no 0 0 1 0 detritivore
2016 August R63 8 humus Coleoptera Carabidae Calathus_micropterus 1 3.44 yes yes no 0 1 0 0 predator
2016 August R63 8 humus Araneae Lycosidae Pardosa_sp 1 0.18 yes yes no 0 1 0 0 predator
2016 August R63 8 humus Coleoptera Staphylinidae Geostiba_circellaris 1 0.09 yes yes no 0 1 0 0 predator
2016 August R63 8 humus Coleoptera Staphylinidae Othius_subuliformis 1 0.55 yes yes no 0 1 0 0 predator
2016 August R63 8 humus Coleoptera Staphylinidae Xantholinus_tricolor 1 1.62 yes yes no 0 1 0 0 predator
2016 August R63 8 humus Hemiptera Cydnidae Adomerus_biguttatus 1 5.40 yes yes yes 1 0 0 0 herbivore
2016 August R63 9 humus Araneae Gnaphosidae Zelotes_sp 1 0.24 yes yes no 0 1 0 0 predator
2016 August R63 9 humus Araneae Linyphiidae Asthenargus_paganus 1 0.74 yes yes no 0 1 0 0 predator
2016 August R63 9 humus Araneae Linyphiidae Gen_sp 4 0.20 yes yes no 0 1 0 0 predator
2016 August R63 9 humus Araneae Linyphiidae Porrhomma_pallidum 2 0.21 yes yes no 0 1 0 0 predator
2016 August R63 9 humus Araneae Linyphiidae Tapinocyba_pallens 1 1.27 yes yes no 0 1 0 0 predator
2016 August R63 9 humus Araneae Linyphiidae Tapinopa_longidens 1 0.36 yes yes no 0 1 0 0 predator
2016 August R63 9 humus Araneae Lycosidae Pardosa_sp 1 0.14 yes yes no 0 1 0 0 predator
2016 August R63 9 humus Diptera Rhagionidae Rhagio_lineola 1 2.90 yes yes no 0 1 0 0 predator
2016 August R63 9 humus Araneae Theridiidae Gen_sp 1 0.08 yes yes no 0 1 0 0 predator
2016 August R63 9 humus Araneae Theridiidae Robertus_lividus 1 1.37 yes yes no 0 1 0 0 predator
2016 August R63 9 humus Hymenoptera Formicidae Lasius_platythorax 1 0.36 Yes Yes no 0.8 0.2 0 0 combined
2016 August R63 9 mineral Coleoptera Carabidae Calathus_micropterus 1 4.05 yes yes no 0 1 0 0 predator
2016 August R63 9 mineral Coleoptera Carabidae Calathus_micropterus 1 4.06 yes yes no 0 1 0 0 predator
2016 August R63 10 humus Coleoptera Elateridae Athous_subfuscus 1 5.93 yes yes yes 0 0.8 0.1 0.1 combined
2016 August R63 10 humus Coleoptera Elateridae Dalopius_marginatus 1 1.00 yes yes yes 0 0.8 0.1 0.1 combined
2016 August R63 10 humus Araneae Linyphiidae Gen_sp 1 0.03 yes yes no 0 1 0 0 predator
2016 August R63 10 humus Coleoptera Staphylinidae Oxypoda_annularis 1 0.07 yes yes no 0 1 0 0 predator
2016 August R63 10 humus Araneae Theridiidae Robertus_lividus 1 1.62 yes yes no 0 1 0 0 predator
2016 August R63 10 humus Araneae Theridiidae Robertus_sp 1 0.26 yes yes no 0 1 0 0 predator
2016 August R63 10 mineral Coleoptera Carabidae Calathus_micropterus 1 4.05 yes yes no 0 1 0 0 predator
2016 August R63 10 mineral Coleoptera Carabidae Calathus_micropterus 1 4.06 yes yes no 0 1 0 0 predator
2016 August R64 1 humus Coleoptera Cantharidae Malthodes_fuscus 1 0.32 yes yes no 0 1 0 0 predator
2016 August R64 1 humus Coleoptera Cantharidae Malthodes_mysticus 1 0.53 yes yes no 0 1 0 0 predator
2016 August R64 1 humus Coleoptera Cantharidae Podistra_schoenherri 1 1.26 yes yes no 0 1 0 0 predator
2016 August R64 1 humus Araneae Theridiidae Robertus_scoticus 1 0.26 yes yes no 0 1 0 0 predator
2016 August R64 1 humus Araneae Theridiidae Robertus_sp 1 0.03 yes yes no 0 1 0 0 predator
2016 August R64 2 humus Araneae Linyphiidae Sisicus_apertus 1 0.06 yes yes no 0 1 0 0 predator
2016 August R64 2 humus Coleoptera Staphylinidae Othius_sp 1 0.23 yes yes no 0 1 0 0 predator
2016 August R64 3 humus Coleoptera Elateridae Eanus_costalis 1 5.88 yes yes no 0 0.5 0.5 0 combined
2016 August R64 3 humus Coleoptera Elateridae Athous_subfuscus 1 3.36 yes yes yes 0 0.8 0.1 0.1 combined
2016 August R64 3 humus Diptera Muscidae Phaonia_subventa 1 2.77 yes yes no 0 1 0 0 predator

2016 August R64 3 humus Coleoptera Staphylinidae Drusilla_canaliculata 1 0.77 yes yes no 0 1 0 0 predator
2016 August R64 3 mineral Araneae Theridiidae Robertus_sp 1 0.65 yes yes no 0 1 0 0 predator
2016 August R64 4 humus Diptera Lauxaniidae Sapromyza_hyalinata 1 0.29 yes yes no 0 0 1 0 detritivore
2016 August R64 4 humus Coleoptera Elateridae Eanus_costalis 1 5.21 yes yes no 0 0.5 0.5 0 combined
2016 August R64 4 humus Coleoptera Elateridae Athous_subfuscus 1 5.00 yes yes yes 0 0.8 0.1 0.1 combined
2016 August R64 4 humus Coleoptera Cantharidae Podistra_schoenherri 1 0.52 yes yes no 0 1 0 0 predator
2016 August R64 4 humus Coleoptera Cantharidae Podistra_schoenherri 1 1.23 yes yes no 0 1 0 0 predator
2016 August R64 4 humus Araneae Linyphiidae Tapinocyba_pallens 2 0.36 yes yes no 0 1 0 0 predator
2016 August R64 4 humus Myriapoda Lithobiidae Lithobius_curtipes 1 1.34 yes yes no 0 1 0 0 predator
2016 August R64 4 humus Coleoptera Staphylinidae Oxypoda_annularis 1 0.06 yes yes no 0 1 0 0 predator
2016 August R64 4 humus Araneae Theridiidae Robertus_lividus 2 1.89 yes yes no 0 1 0 0 predator
2016 August R64 4 humus Araneae Theridiidae Robertus_scoticus 1 1.28 yes yes no 0 1 0 0 predator
2016 August R64 4 humus Araneae Theridiidae Robertus_sp 6 0.33 yes yes no 0 1 0 0 predator
2016 August R64 4 humus Hymenoptera Formicidae Lasius_platythorax 2 0.64 Yes Yes no 0.8 0.2 0 0 combined
2016 August R64 4 humus Lepidoptera Hepialidae Phymatopus_hecta 1 18.03 Yes Yes yes 1 0 0 0 herbivore
2016 August R64 4 mineral Araneae Theridiidae Robertus_sp 1 0.65 yes yes no 0 1 0 0 predator
2016 August R64 5 humus Diptera Lonchaeidae Lonchaea_ragnari 1 1.97 yes yes no 0 0 1 0 detritivore
2016 August R64 5 humus Coleoptera Elateridae Eanus_costalis 1 0.42 yes yes no 0 0.5 0.5 0 combined
2016 August R64 5 humus Coleoptera Elateridae Eanus_costalis 1 1.55 yes yes no 0 0.5 0.5 0 combined
2016 August R64 5 humus Coleoptera Elateridae Athous_subfuscus 1 0.13 yes yes yes 0 0.8 0.1 0.1 combined
2016 August R64 5 humus Coleoptera Staphylinidae Mocyta_fungi 1 0.11 yes yes no 0 1 0 0 predator
2016 August R64 5 humus Coleoptera Staphylinidae Othius_subuliformis 1 0.49 yes yes no 0 1 0 0 predator
2016 August R64 5 humus Araneae Theridiidae Robertus_lividus 1 0.30 yes yes no 0 1 0 0 predator
2016 August R64 5 humus Araneae Theridiidae Robertus_scoticus 2 2.10 yes yes no 0 1 0 0 predator
2016 August R64 5 humus Hemiptera Lygaeidae Eremocoris_abietis 1 1.38 yes no no 1 0 0 0 herbivore
2016 August R64 5 humus Hemiptera Lygaeidae Eremocoris_sp 1 0.29 yes no no 1 0 0 0 herbivore
2016 August R64 6 humus Diptera Lonchaeidae Lonchaea_ragnari 1 2.27 yes yes no 0 0 1 0 detritivore
2016 August R64 6 humus Araneae Linyphiidae Gen_sp 1 3.02 yes yes no 0 1 0 0 predator
2016 August R64 6 humus Araneae Philodromidae Philodromus_sp 1 2.94 yes yes no 0 1 0 0 predator
2016 August R64 6 humus Coleoptera Elateridae Paraphotistus_impressus 1 10.58 yes yes yes 0.6 0.13 0.13 0.13 combined
2016 August R64 7 humus Coleoptera Cantharidae Podistra_schoenherri 1 0.94 yes yes no 0 1 0 0 predator
2016 August R64 7 humus Coleoptera Staphylinidae Ischnosoma_longicorne 1 0.24 yes yes no 0 1 0 0 predator
2016 August R64 7 humus Araneae Theridiidae Robertus_scoticus 1 0.17 yes yes no 0 1 0 0 predator
2016 August R64 7 humus Araneae Theridiidae Robertus_sp 1 0.26 yes yes no 0 1 0 0 predator
2016 August R64 7 mineral Araneae Theridiidae Robertus_sp 1 0.21 yes yes no 0 1 0 0 predator
2016 August R64 8 humus Coleoptera Elateridae Athous_subfuscus 1 0.21 yes yes yes 0 0.8 0.1 0.1 combined
2016 August R64 8 humus Coleoptera Elateridae Athous_subfuscus 1 1.76 yes yes yes 0 0.8 0.1 0.1 combined
2016 August R64 8 humus Coleoptera Elateridae Athous_subfuscus 1 1.93 yes yes yes 0 0.8 0.1 0.1 combined
2016 August R64 8 humus Coleoptera Carabidae Calathus_micropterus 1 2.72 yes yes no 0 1 0 0 predator
2016 August R64 8 mineral Araneae Theridiidae Robertus_sp 1 0.21 yes yes no 0 1 0 0 predator
2016 August R64 9 humus Coleoptera Elateridae Athous_subfuscus 1 1.68 yes yes yes 0 0.8 0.1 0.1 combined
2016 August R64 9 humus Coleoptera Elateridae Athous_subfuscus 1 1.85 yes yes yes 0 0.8 0.1 0.1 combined
2016 August R64 9 humus Coleoptera Elateridae Athous_subfuscus 1 2.35 yes yes yes 0 0.8 0.1 0.1 combined
2016 August R64 10 humus Hymenoptera Formicidae Myrmica_ruginodis 1 0.65 Yes Yes no 0 1 0 0 predator
2016 August R64 10 humus Araneae Linyphiidae Tapinocyba_pallens 1 0.10 yes yes no 0 1 0 0 predator
2016 August R64 10 humus Coleoptera Staphylinidae Othius_subuliformis 1 0.53 yes yes no 0 1 0 0 predator
2016 August R65 1 humus Coleoptera Elateridae Ampedus_cf_nigrinus 1 1.13 yes yes no 0 0.5 0.5 0 combined
2016 August R65 1 humus Coleoptera Elateridae Eanus_costalis 1 1.26 yes yes no 0 0.5 0.5 0 combined
2016 August R65 1 humus Coleoptera Elateridae Eanus_costalis 1 1.89 yes yes no 0 0.5 0.5 0 combined
2016 August R65 1 humus Coleoptera Elateridae Eanus_costalis 1 2.60 yes yes no 0 0.5 0.5 0 combined
2016 August R65 1 humus Coleoptera Elateridae Paraphotistus_impressus 1 1.93 yes yes yes 0.6 0.13 0.13 0.13 combined
2016 August R65 2 humus Coleoptera Elateridae Eanus_costalis 1 2.90 yes yes no 0 0.5 0.5 0 combined
2016 August R65 2 humus Coleoptera Elateridae Eanus_costalis 1 3.11 yes yes no 0 0.5 0.5 0 combined

2016 August R65 2 humus Coleoptera Elateridae Eanus_costalis 1 4.07 yes yes no 0 0.5 0.5 0 combined
2016 August R65 2 humus Coleoptera Elateridae Athous_subfuscus 1 0.46 yes yes yes 0 0.8 0.1 0.1 combined
2016 August R65 2 humus Coleoptera Elateridae Athous_subfuscus 1 2.35 yes yes yes 0 0.8 0.1 0.1 combined
2016 August R65 2 humus Araneae Linyphiidae Tapinocyba_pallens 3 3.16 yes yes no 0 1 0 0 predator
2016 August R65 2 humus Myriapoda Lithobiidae Lithobius_cf_curtipes 1 0.60 yes yes no 0 1 0 0 predator
2016 August R65 2 humus Coleoptera Staphylinidae Othius_lapidicola 1 0.72 yes yes no 0 1 0 0 predator
2016 August R65 2 humus Coleoptera Staphylinidae Othius_subuliformis 1 0.49 yes yes no 0 1 0 0 predator
2016 August R65 2 humus Coleoptera Staphylinidae Quedius_meridiocarpathicus 1 4.99 yes yes no 0 1 0 0 predator
2016 August R65 2 humus Araneae Thomisidae Ozyptila_sp 1 0.12 yes yes no 0 1 0 0 predator
2016 August R65 2 humus Araneae Thomisidae Xysticus_sp 1 0.10 yes yes no 0 1 0 0 predator
2016 August R65 3 humus Coleoptera Elateridae Ampedus_cf_nigrinus 1 4.49 yes yes no 0 0.5 0.5 0 combined
2016 August R65 3 humus Myriapoda Lithobiidae Lithobius_curtipes 1 0.96 yes yes no 0 1 0 0 predator
2016 August R65 3 humus Coleoptera Staphylinidae Drusilla_canaliculata 1 0.73 yes yes no 0 1 0 0 predator
2016 August R65 3 mineral Araneae Linyphiidae Neriene_sp 1 0.12 yes yes no 0 1 0 0 predator
2016 August R65 4 humus Coleoptera Elateridae Eanus_costalis 1 0.17 yes yes no 0 0.5 0.5 0 combined
2016 August R65 4 humus Coleoptera Elateridae Eanus_costalis 1 2.69 yes yes no 0 0.5 0.5 0 combined
2016 August R65 4 humus Araneae Linyphiidae Centromerus_arcanus 1 0.05 yes yes no 0 1 0 0 predator
2016 August R65 4 humus Araneae Linyphiidae Gen_sp 2 0.50 yes yes no 0 1 0 0 predator
2016 August R65 4 humus Coleoptera Staphylinidae Drusilla_canaliculata 1 0.82 yes yes no 0 1 0 0 predator
2016 August R65 4 humus Araneae Theridiidae Robertus_scoticus 1 0.10 yes yes no 0 1 0 0 predator
2016 August R65 4 mineral Araneae Linyphiidae Neriene_sp 1 0.12 yes yes no 0 1 0 0 predator
2016 August R65 5 humus Myriapoda Lithobiidae Lithobius_sp 1 0.34 yes yes no 0 1 0 0 predator
2016 August R65 5 mineral Araneae Linyphiidae Tapinocyba_pallens 1 0.13 yes yes no 0 1 0 0 predator
2016 August R65 6 humus Coleoptera Carabidae Calathus_micropterus 1 5.25 yes yes no 0 1 0 0 predator
2016 August R65 6 humus Hymenoptera Formicidae Myrmica_lobicornis 1 0.45 Yes Yes no 0 1 0 0 predator
2016 August R65 6 mineral Araneae Linyphiidae Tapinocyba_pallens 1 0.13 yes yes no 0 1 0 0 predator
2016 August R65 7 humus Diptera Cecidomyidae Lestremia_cinerea 1 0.42 yes yes no 0 0 0 1 fungivore
2016 August R65 7 humus Annelida Lumbricidae . 1 0.93 yes yes no 0 0 1 0 detritivore
2016 August R65 7 humus Hymenoptera Formicidae Myrmica_ruginodis 1 0.49 Yes Yes no 0 1 0 0 predator
2016 August R65 7 humus Araneae Linyphiidae Gen_sp 1 0.34 yes yes no 0 1 0 0 predator
2016 August R65 7 humus Araneae Linyphiidae Tapinocyba_pallens 7 1.13 yes yes no 0 1 0 0 predator
2016 August R65 7 humus Araneae Lycosidae Gen_sp 1 0.13 yes yes no 0 1 0 0 predator
2016 August R65 7 humus Coleoptera Staphylinidae Othius_subuliformis 1 0.52 yes yes no 0 1 0 0 predator
2016 August R65 7 humus Coleoptera Staphylinidae Othius_subuliformis 1 0.57 yes yes no 0 1 0 0 predator
2016 August R65 7 humus Coleoptera Staphylinidae Othius_subuliformis 1 0.65 yes yes no 0 1 0 0 predator
2016 August R65 7 humus Coleoptera Curculionidae Polydrusus_fulvicornis 1 1.09 yes yes yes 1 0 0 0 herbivore
2016 August R65 8 humus Annelida Lumbricidae Dendrobaena_octaedra 1 2.79 yes yes no 0 0 1 0 detritivore
2016 August R65 8 humus Annelida Lumbricidae Eiseniella_tetraedra 1 0.31 yes yes no 0 0 1 0 detritivore
2016 August R65 8 humus Myriapoda Lithobiidae Lithobius_curtipes 1 0.96 yes yes no 0 1 0 0 predator
2016 August R65 9 humus Annelida Lumbricidae . 1 0.39 yes yes no 0 0 1 0 detritivore
2016 August R65 9 humus Coleoptera Elateridae Eanus_costalis 1 3.40 yes yes no 0 0.5 0.5 0 combined
2016 August R65 9 humus Coleoptera Elateridae Eanus_costalis 1 6.64 yes yes no 0 0.5 0.5 0 combined
2016 August R65 9 humus Coleoptera Elateridae Athous_subfuscus 1 2.14 yes yes yes 0 0.8 0.1 0.1 combined
2016 August R65 9 humus Coleoptera Carabidae Calathus_micropterus 1 5.62 yes yes no 0 1 0 0 predator
2016 August R65 9 humus Coleoptera Staphylinidae Othius_subuliformis 1 0.55 yes yes no 0 1 0 0 predator
2016 August R65 9 humus Araneae Theridiidae Robertus_sp 1 0.14 yes yes no 0 1 0 0 predator
2016 August R65 9 humus Coleoptera Rhynchitidae Deporaus_betulae 1 0.94 yes no no 1 0 0 0 herbivore
2016 August R65 9 mineral Coleoptera Elateridae Athous_subfuscus 1 1.18 yes yes yes 0 0.8 0.1 0.1 combined
2016 August R65 9 mineral Coleoptera Curculionidae . 1 1.08 yes yes yes 1 0 0 0 herbivore
2016 August R65 10 humus Coleoptera Elateridae Athous_subfuscus 1 1.81 yes yes yes 0 0.8 0.1 0.1 combined
2016 August R65 10 humus Diptera Rhagionidae Rhagio_scolopaceus 1 0.92 yes yes no 0 1 0 0 predator
2016 August R65 10 humus Araneae Theridiidae Robertus_sp 1 0.25 yes yes no 0 1 0 0 predator
2016 August R65 10 humus Hemiptera Lygaeidae Eremocoris_sp 1 0.42 yes no no 1 0 0 0 herbivore

2016 August R65 10 mineral Coleoptera Elateridae Athous_subfuscus 1 1.18 yes yes yes 0 0.8 0.1 0.1 combined
2016 August R65 10 mineral Coleoptera Curculionidae . 1 1.08 yes yes yes 1 0 0 0 herbivore
2016 August R66 1 humus Coleoptera Elateridae Athous_subfuscus 1 4.25 yes yes yes 0 0.8 0.1 0.1 combined
2016 August R66 1 humus Coleoptera Carabidae Notiophilus_biguttatus 1 1.43 yes yes no 0 1 0 0 predator
2016 August R66 1 humus Myriapoda Lithobiidae Lithobius_cf_curtipes 1 0.29 yes yes no 0 1 0 0 predator
2016 August R66 1 humus Myriapoda Lithobiidae Lithobius_cf_curtipes 1 0.58 yes yes no 0 1 0 0 predator
2016 August R66 1 humus Araneae Theridiidae Robertus_sp 1 0.38 yes yes no 0 1 0 0 predator
2016 August R66 1 humus Coleoptera Scirtidae Cyphon_padi 1 0.30 no no no 0.5 0 0.5 0 combined
2016 August R66 1 humus Coleoptera Curculionidae Otiorhynchus_ligustici 1 8.59 yes yes yes 1 0 0 0 herbivore
2016 August R66 2 humus Coleoptera Elateridae Athous_subfuscus 1 0.88 yes yes yes 0 0.8 0.1 0.1 combined
2016 August R66 2 humus Hymenoptera Formicidae Myrmica_ruginodis 1 0.49 Yes Yes no 0 1 0 0 predator
2016 August R66 2 humus Coleoptera Scirtidae Cyphon_padi 1 0.14 no no no 0.5 0 0.5 0 combined
2016 August R66 3 humus Araneae Linyphiidae Centromerus_incilium 1 0.60 yes yes no 0 1 0 0 predator
2016 August R66 3 humus Coleoptera Staphylinidae Othius_lapidicola 1 0.87 yes yes no 0 1 0 0 predator
2016 August R66 3 humus Araneae Thomisidae Ozyptila_sp 1 0.71 yes yes no 0 1 0 0 predator
2016 August R66 3 mineral Coleoptera Cantharidae Malthodes_fuscus 1 0.35 yes yes no 0 1 0 0 predator
2016 August R66 4 humus Coleoptera Elateridae Eanus_costalis 1 1.43 yes yes no 0 0.5 0.5 0 combined
2016 August R66 4 humus Coleoptera Elateridae Athous_subfuscus 1 1.26 yes yes yes 0 0.8 0.1 0.1 combined
2016 August R66 4 humus Coleoptera Elateridae Athous_subfuscus 1 1.34 yes yes yes 0 0.8 0.1 0.1 combined
2016 August R66 4 humus Araneae Linyphiidae Tenuiphantes_alacris 1 0.32 yes yes no 0 1 0 0 predator
2016 August R66 4 humus Myriapoda Lithobiidae Lithobius_curtipes 1 2.04 yes yes no 0 1 0 0 predator
2016 August R66 4 humus Coleoptera Staphylinidae Othius_subuliformis 1 0.56 yes yes no 0 1 0 0 predator
2016 August R66 4 humus Araneae Theridiidae Robertus_scoticus 2 0.81 yes yes no 0 1 0 0 predator
2016 August R66 4 humus Araneae Theridiidae Robertus_sp 5 0.81 yes yes no 0 1 0 0 predator
2016 August R66 4 mineral Coleoptera Cantharidae Malthodes_fuscus 1 0.35 yes yes no 0 1 0 0 predator
2016 August R66 5 humus Coleoptera Elateridae Eanus_costalis 1 5.17 yes yes no 0 0.5 0.5 0 combined
2016 August R66 5 humus Coleoptera Elateridae Eanus_costalis 1 6.26 yes yes no 0 0.5 0.5 0 combined
2016 August R66 5 humus Coleoptera Elateridae Athous_subfuscus 1 1.09 yes yes yes 0 0.8 0.1 0.1 combined
2016 August R66 5 humus Coleoptera Elateridae Athous_subfuscus 1 2.35 yes yes yes 0 0.8 0.1 0.1 combined
2016 August R66 5 humus Coleoptera Elateridae Athous_subfuscus 1 4.12 yes yes yes 0 0.8 0.1 0.1 combined
2016 August R66 5 humus Araneae Linyphiidae Tenuiphantes_sp 1 0.19 yes yes no 0 1 0 0 predator
2016 August R66 5 humus Coleoptera Staphylinidae Othius_lapidicola 1 0.85 yes yes no 0 1 0 0 predator
2016 August R66 5 humus Coleoptera Staphylinidae Oxypoda_annularis 1 0.09 yes yes no 0 1 0 0 predator
2016 August R66 5 mineral Coleoptera Elateridae Paraphotistus_impressus 1 0.67 yes yes yes 0.6 0.13 0.13 0.13 combined
2016 August R66 6 humus Annelida Lumbricidae Eiseniella_tetraedra 1 0.62 yes yes no 0 0 1 0 detritivore
2016 August R66 6 humus Araneae Linyphiidae Tapinocyba_pallens 1 0.08 yes yes no 0 1 0 0 predator
2016 August R66 6 humus Araneae Linyphiidae Tapinocyba_sp 1 0.56 yes yes no 0 1 0 0 predator
2016 August R66 6 humus Coleoptera Staphylinidae Othius_lapidicola 1 0.68 yes yes no 0 1 0 0 predator
2016 August R66 6 humus Coleoptera Staphylinidae Othius_lapidicola 1 0.89 yes yes no 0 1 0 0 predator
2016 August R66 6 humus Coleoptera Staphylinidae Oxypoda_annularis 1 0.02 yes yes no 0 1 0 0 predator
2016 August R66 6 humus Araneae Theridiidae Robertus_sp 1 0.10 yes yes no 0 1 0 0 predator
2016 August R66 6 mineral Coleoptera Elateridae Paraphotistus_impressus 1 0.67 yes yes yes 0.6 0.13 0.13 0.13 combined
2016 August R66 7 humus Annelida Lumbricidae Dendrobaena_octaedra 1 18.71 yes yes no 0 0 1 0 detritivore
2016 August R66 7 humus Coleoptera Elateridae Eanus_costalis 1 1.89 yes yes no 0 0.5 0.5 0 combined
2016 August R66 7 humus Coleoptera Elateridae Athous_subfuscus 1 1.18 yes yes yes 0 0.8 0.1 0.1 combined
2016 August R66 7 humus Coleoptera Cantharidae Podistra_schoenherri 1 0.66 yes yes no 0 1 0 0 predator
2016 August R66 7 humus Araneae Linyphiidae Gen_sp 2 0.34 yes yes no 0 1 0 0 predator
2016 August R66 7 humus Araneae Linyphiidae Tapinocyba_pallens 3 3.67 yes yes no 0 1 0 0 predator
2016 August R66 7 humus Coleoptera Staphylinidae Oxypoda_annularis 1 0.04 yes yes no 0 1 0 0 predator
2016 August R66 7 humus Coleoptera Scirtidae Cyphon_padi 1 0.21 no no no 0.5 0 0.5 0 combined
2016 August R66 7 humus Coleoptera Scirtidae Cyphon_padi 1 0.25 no no no 0.5 0 0.5 0 combined
2016 August R66 8 humus Coleoptera Elateridae Eanus_costalis 1 1.55 yes yes no 0 0.5 0.5 0 combined
2016 August R66 8 humus Coleoptera Cantharidae Podistra_schoenherri 1 0.71 yes yes no 0 1 0 0 predator

2016 August R66 8 humus Myriapoda Lithobiidae Lithobius_curtipes 1 1.13 yes yes no 0 1 0 0 predator
2016 August R66 8 humus Coleoptera Staphylinidae Oxypoda_annularis 1 0.07 yes yes no 0 1 0 0 predator
2016 August R66 8 humus Coleoptera Staphylinidae Oxypoda_annularis 1 0.09 yes yes no 0 1 0 0 predator
2016 August R66 8 humus Coleoptera Scirtidae Cyphon_padi 1 0.16 no no no 0.5 0 0.5 0 combined
2016 August R66 8 humus Coleoptera Scirtidae Cyphon_padi 1 0.19 no no no 0.5 0 0.5 0 combined
2016 August R66 8 humus Coleoptera Scirtidae Cyphon_padi 1 0.22 no no no 0.5 0 0.5 0 combined
2016 August R66 8 humus Coleoptera Scirtidae Cyphon_padi 1 0.24 no no no 0.5 0 0.5 0 combined
2016 August R66 8 humus Coleoptera Scirtidae Cyphon_padi 1 0.25 no no no 0.5 0 0.5 0 combined
2016 August R66 9 humus Annelida Lumbricidae Eiseniella_tetraedra 1 0.10 yes yes no 0 0 1 0 detritivore
2016 August R66 9 humus Araneae Cybaeidae Cryphoeca_silvicola 1 1.31 yes yes no 0 1 0 0 predator
2016 August R66 9 humus Myriapoda Lithobiidae Lithobius_curtipes 1 0.67 yes yes no 0 1 0 0 predator
2016 August R66 9 humus Myriapoda Lithobiidae Lithobius_curtipes 1 0.70 yes yes no 0 1 0 0 predator
2016 August R66 9 humus Myriapoda Lithobiidae Lithobius_curtipes 1 1.01 yes yes no 0 1 0 0 predator
2016 August R66 9 humus Myriapoda Lithobiidae Lithobius_curtipes 1 1.49 yes yes no 0 1 0 0 predator
2016 August R66 9 humus Myriapoda Lithobiidae Lithobius_curtipes 1 1.54 yes yes no 0 1 0 0 predator
2016 August R66 9 humus Myriapoda Lithobiidae Lithobius_curtipes 1 1.70 yes yes no 0 1 0 0 predator
2016 August R66 9 humus Diptera Rhagionidae Rhagio_tringarius 1 7.56 yes yes no 0 1 0 0 predator
2016 August R66 9 humus Coleoptera Staphylinidae Oxypoda_annularis 1 0.08 yes yes no 0 1 0 0 predator
2016 August R66 9 mineral Coleoptera Elateridae Athous_subfuscus 1 3.40 yes yes yes 0 0.8 0.1 0.1 combined
2016 August R66 9 mineral Coleoptera Elateridae Athous_subfuscus 1 4.33 yes yes yes 0 0.8 0.1 0.1 combined
2016 August R66 10 humus Coleoptera Elateridae Athous_subfuscus 1 0.84 yes yes yes 0 0.8 0.1 0.1 combined
2016 August R66 10 humus Myriapoda Lithobiidae Lithobius_curtipes 1 0.48 yes yes no 0 1 0 0 predator
2016 August R66 10 humus Myriapoda Lithobiidae Lithobius_curtipes 1 0.94 yes yes no 0 1 0 0 predator
2016 August R66 10 humus Diptera Muscidae Spilogona_contractifrons 1 0.25 yes yes no 0 1 0 0 predator
2016 August R66 10 humus Coleoptera Staphylinidae . 1 0.64 yes yes no 0 1 0 0 predator
2016 August R66 10 humus Coleoptera Staphylinidae Othius_lapidicola 1 0.91 yes yes no 0 1 0 0 predator
2016 August R66 10 humus Coleoptera Staphylinidae Oxypoda_annularis 1 0.05 yes yes no 0 1 0 0 predator
2016 August R66 10 humus Coleoptera Staphylinidae Oxypoda_annularis 1 0.10 yes yes no 0 1 0 0 predator
2016 August R66 10 humus Coleoptera Scirtidae Cyphon_padi 1 0.17 no no no 0.5 0 0.5 0 combined
2016 August R66 10 humus Coleoptera Scirtidae Cyphon_padi 1 0.22 no no no 0.5 0 0.5 0 combined
2016 August R66 10 humus Coleoptera . . 1 0.04 . . . .
2016 August R66 10 mineral Coleoptera Elateridae Athous_subfuscus 1 3.40 yes yes yes 0 0.8 0.1 0.1 combined
2016 August R66 10 mineral Coleoptera Elateridae Athous_subfuscus 1 4.33 yes yes yes 0 0.8 0.1 0.1 combined
2016 August R67 1 humus Annelida Lumbricidae Eiseniella_tetraedra 1 0.42 yes yes no 0 0 1 0 detritivore
2016 August R67 1 humus Coleoptera Elateridae Athous_subfuscus 1 3.07 yes yes yes 0 0.8 0.1 0.1 combined
2016 August R67 1 humus Araneae Linyphiidae Tapinocyba_pallens 1 0.17 yes yes no 0 1 0 0 predator
2016 August R67 1 humus Diptera Muscidae Spilogona_contractifrons 1 0.25 yes yes no 0 1 0 0 predator
2016 August R67 1 humus Araneae Theridiidae Robertus_sp 1 0.10 yes yes no 0 1 0 0 predator
2016 August R67 1 humus Coleoptera Elateridae Paraphotistus_impressus 1 28.85 yes yes yes 0.6 0.13 0.13 0.13 combined
2016 August R67 2 humus Coleoptera Cantharidae Podistra_schoenherri 1 0.80 yes yes no 0 1 0 0 predator
2016 August R67 2 humus Araneae Linyphiidae Tapinocyba_pallens 4 0.41 yes yes no 0 1 0 0 predator
2016 August R67 2 humus Araneae Theridiidae Robertus_scoticus 2 1.08 yes yes no 0 1 0 0 predator
2016 August R67 2 humus Araneae Theridiidae Robertus_sp 1 0.23 yes yes no 0 1 0 0 predator
2016 August R67 2 humus Hemiptera Lygaeidae Eremocoris_abietis 1 0.33 yes no no 1 0 0 0 herbivore
2016 August R67 3 humus Annelida Lumbricidae Eiseniella_tetraedra 1 0.52 yes yes no 0 0 1 0 detritivore
2016 August R67 3 humus Annelida Lumbricidae Eiseniella_tetraedra 1 0.73 yes yes no 0 0 1 0 detritivore
2016 August R67 3 humus Araneae Cybaeidae Cryphoeca_silvicola 1 1.55 yes yes no 0 1 0 0 predator
2016 August R67 3 humus Araneae Linyphiidae Tapinocyba_pallens 1 0.34 yes yes no 0 1 0 0 predator
2016 August R67 3 humus Myriapoda Lithobiidae Lithobius_curtipes 1 2.06 yes yes no 0 1 0 0 predator
2016 August R67 3 humus Myriapoda Lithobiidae Lithobius_sp 1 0.19 yes yes no 0 1 0 0 predator
2016 August R67 3 humus Araneae Theridiidae Robertus_scoticus 1 0.08 yes yes no 0 1 0 0 predator
2016 August R67 4 humus Annelida Lumbricidae Eiseniella_tetraedra 1 0.31 yes yes no 0 0 1 0 detritivore
2016 August R67 4 humus Coleoptera Elateridae Eanus_costalis 1 1.26 yes yes no 0 0.5 0.5 0 combined

2016 August R67 4 humus Coleoptera Cantharidae Podistra_schoenherri 1 5.47 yes yes no 0 1 0 0 predator
2016 August R67 4 humus Coleoptera Staphylinidae Oxypoda_annularis 1 0.03 yes yes no 0 1 0 0 predator
2016 August R67 4 humus Araneae Theridiidae Robertus_sp 1 0.15 yes yes no 0 1 0 0 predator
2016 August R67 4 humus Hemiptera Lygaeidae Eremocoris_sp 1 0.75 yes no no 1 0 0 0 herbivore
2016 August R67 5 humus Araneae Theridiidae Robertus_scoticus 1 0.38 yes yes no 0 1 0 0 predator
2016 August R67 6 humus Araneae Theridiidae Robertus_scoticus 1 0.26 yes yes no 0 1 0 0 predator
2016 August R67 6 humus Coleoptera Elateridae . 1 7.71 yes yes . . . . . .
2016 August R67 7 humus Coleoptera Elateridae Eanus_costalis 1 5.83 yes yes yes 0 0.5 0.5 0 combined
2016 August R67 7 humus Araneae Linyphiidae Ceratinella_brevipes 1 0.24 yes yes no 0 1 0 0 predator
2016 August R67 7 humus Araneae Theridiidae Robertus_scoticus 1 0.12 yes yes no 0 1 0 0 predator
2016 August R67 7 humus Araneae Theridiidae Robertus_sp 1 0.23 yes yes no 0 1 0 0 predator
2016 August R67 7 mineral Hymenoptera Pamphilidae Acantholyda_posticalis 1 18.35 Yes no no 1 0 0 0 herbivore
2016 August R67 8 humus Diptera Lonchaeidae Lonchaea_ragnari 1 2.31 yes yes no 0 0 1 0 detritivore
2016 August R67 8 humus Diptera Lonchaeidae Lonchaea_ragnari 1 2.73 yes yes no 0 0 1 0 detritivore
2016 August R67 8 humus Coleoptera Elateridae Athous_subfuscus 1 3.70 yes yes yes 0 0.8 0.1 0.1 combined
2016 August R67 8 humus Araneae Linyphiidae Walckenaeria_clavicornis 1 0.44 yes yes no 0 1 0 0 predator
2016 August R67 8 humus Coleoptera Staphylinidae Oxypoda_annularis 1 0.08 yes yes no 0 1 0 0 predator
2016 August R67 8 humus Coleoptera Staphylinidae Oxypoda_annularis 1 0.10 yes yes no 0 1 0 0 predator
2016 August R67 8 humus Araneae Theridiidae Robertus_scoticus 1 0.34 yes yes no 0 1 0 0 predator
2016 August R67 8 mineral Hymenoptera Pamphilidae Acantholyda_posticalis 1 18.35 Yes no no 1 0 0 0 herbivore
2016 August R67 9 humus Coleoptera Elateridae Athous_subfuscus 1 0.34 yes yes yes 0 0.8 0.1 0.1 combined
2016 August R67 9 humus Coleoptera Elateridae Athous_subfuscus 1 2.81 yes yes yes 0 0.8 0.1 0.1 combined
2016 August R67 9 humus Coleoptera Staphylinidae Mycetoporus_clavicornis 1 0.23 yes yes no 0 1 0 0 predator
2016 August R67 9 humus Coleoptera Staphylinidae Oxypoda_annularis 1 0.08 yes yes no 0 1 0 0 predator
2016 August R67 9 humus Coleoptera Staphylinidae Oxypoda_annularis 1 0.08 yes yes no 0 1 0 0 predator
2016 August R67 9 humus Coleoptera Staphylinidae Oxypoda_annularis 1 0.09 yes yes no 0 1 0 0 predator
2016 August R67 9 humus Araneae Theridiidae Gen_sp 2 0.36 yes yes no 0 1 0 0 predator
2016 August R67 9 humus Araneae Theridiidae Robertus_scoticus 3 0.74 yes yes no 0 1 0 0 predator
2016 August R67 9 mineral Coleoptera Carabidae Calathus_micropterus 1 3.66 yes yes no 0 1 0 0 predator
2016 August R67 9 mineral Coleoptera Elateridae Paraphotistus_impressus 1 13.61 yes yes yes 0.6 0.13 0.13 0.13 combined
2016 August R67 10 humus Coleoptera Elateridae Eanus_costalis 1 0.88 yes yes no 0 0.5 0.5 0 combined
2016 August R67 10 humus Coleoptera Elateridae Eanus_costalis 1 2.86 yes yes no 0 0.5 0.5 0 combined
2016 August R67 10 humus Araneae Linyphiidae Gen_sp 1 0.13 yes yes no 0 1 0 0 predator
2016 August R67 10 humus Araneae Linyphiidae Tapinocyba_pallens 1 0.24 yes yes no 0 1 0 0 predator
2016 August R67 10 humus Araneae Theridiidae Robertus_scoticus 1 0.32 yes yes no 0 1 0 0 predator
2016 August R67 10 humus Araneae Theridiidae Robertus_sp 4 0.45 yes yes no 0 1 0 0 predator
2016 August R67 10 humus Diptera Chironomidae Metriocnemus_sp 1 0.09 yes yes yes 0.25 0 0.75 0 combined
2016 August R67 10 mineral Coleoptera Carabidae Calathus_micropterus 1 3.66 yes yes no 0 1 0 0 predator
2016 August R67 10 mineral Coleoptera Elateridae Paraphotistus_impressus 1 13.61 yes yes yes 0.6 0.13 0.13 0.13 combined
2016 August R68 2 humus Coleoptera Nitidulidae Thelycra_fervida 1 2.69 yes yes no 0 0 0.5 0.5 combined
2016 August R68 2 humus Araneae Linyphiidae Maso_sundevalli 1 0.16 yes yes no 0 1 0 0 predator
2016 August R68 3 humus Coleoptera Elateridae Liotrichus_affinis 1 0.56 yes yes no 0 0.5 0.5 0 combined
2016 August R68 3 humus Coleoptera Cantharidae Malthodes_fuscus 1 0.21 yes yes no 0 1 0 0 predator
2016 August R68 3 humus Hymenoptera Formicidae Myrmica_ruginodis 1 1.15 Yes Yes no 0 1 0 0 predator
2016 August R68 3 humus Araneae Linyphiidae Microneta_viaria 1 0.57 yes yes no 0 1 0 0 predator
2016 August R68 3 humus Myriapoda Lithobiidae Lithobius_curtipes 1 1.78 yes yes no 0 1 0 0 predator
2016 August R68 3 humus Coleoptera . . 1 0.16 . . . .
2016 August R68 3 mineral Coleoptera Curculionidae Polydrusus_fulvicornis 1 6.55 yes yes yes 1 0 0 0 herbivore
2016 August R68 4 humus Araneae Gnaphosidae Haplodrassus_sp 1 2.12 yes yes no 0 1 0 0 predator
2016 August R68 4 mineral Coleoptera Curculionidae Polydrusus_fulvicornis 1 6.55 yes yes yes 1 0 0 0 herbivore
2016 August R68 5 humus Coleoptera Cantharidae Malthodes_fuscus 1 0.45 yes yes no 0 1 0 0 predator
2016 August R68 5 humus Araneae Linyphiidae Macrargus_multesimus 1 1.18 yes yes no 0 1 0 0 predator
2016 August R68 5 humus Araneae Theridiidae Robertus_scoticus 1 0.59 yes yes no 0 1 0 0 predator

2016 August R68 5 mineral Araneae Linyphiidae Macrargus_rufus 1 0.32 yes yes no 0 1 0 0 predator
2016 August R68 6 humus Araneae Theridiidae Robertus_scoticus 1 1.40 yes yes no 0 1 0 0 predator
2016 August R68 6 mineral Araneae Linyphiidae Macrargus_rufus 1 0.32 yes yes no 0 1 0 0 predator
2016 August R68 8 humus Coleoptera Cantharidae Rhagonycha_elongata 1 1.34 yes yes no 0 1 0 0 predator
2016 August R68 9 humus Coleoptera Staphylinidae Atheta_subtilis 1 0.06 yes yes no 0 1 0 0 predator
2016 August R68 9 humus Diptera Chironomidae . 1 0.05 yes yes yes 0.25 0 0.75 0 combined
2016 August R68 9 mineral Hymenoptera Formicidae Myrmica_ruginodis 1 0.44 Yes Yes no 0 1 0 0 predator
2016 August R68 10 humus Araneae Linyphiidae Gen_sp 1 0.13 yes yes no 0 1 0 0 predator
2016 August R68 10 humus Araneae Linyphiidae Microneta_viaria 1 0.11 yes yes no 0 1 0 0 predator
2016 August R68 10 humus Myriapoda Lithobiidae Lithobius_curtipes 1 0.53 yes yes no 0 1 0 0 predator
2016 August R68 10 humus Araneae Theridiidae Gen_sp 1 0.24 yes yes no 0 1 0 0 predator
2016 August R68 10 humus Diptera Chironomidae . 1 0.05 yes yes yes 0.25 0 0.75 0 combined
2016 August R68 10 humus Diptera Chironomidae . 1 0.07 yes yes yes 0.25 0 0.75 0 combined
2016 August R68 10 mineral Hymenoptera Formicidae Myrmica_ruginodis 1 0.44 Yes Yes no 0 1 0 0 predator
2016 August R69 1 humus Coleoptera Elateridae Eanus_costalis 1 3.65 yes yes no 0 0.5 0.5 0 combined
2016 August R69 1 humus Coleoptera Elateridae Eanus_costalis 1 4.33 yes yes no 0 0.5 0.5 0 combined
2016 August R69 1 humus Coleoptera Cantharidae Podistra_schoenherri 1 2.22 yes yes no 0 1 0 0 predator
2016 August R69 1 humus Araneae Linyphiidae Gen_sp 1 0.08 yes yes no 0 1 0 0 predator
2016 August R69 1 humus Myriapoda Lithobiidae Lithobius_curtipes 1 2.04 yes yes no 0 1 0 0 predator
2016 August R69 1 humus Coleoptera Staphylinidae . 1 0.47 yes yes no 0 1 0 0 predator
2016 August R69 1 humus Coleoptera Staphylinidae Liogluta_micans 1 0.26 yes yes no 0 1 0 0 predator
2016 August R69 1 humus Coleoptera Staphylinidae Othius_lapidicola 1 0.61 yes yes no 0 1 0 0 predator
2016 August R69 1 mineral Coleoptera Staphylinidae Othius_lapidicola 1 0.72 yes yes no 0 1 0 0 predator
2016 August R69 2 humus Coleoptera Elateridae Eanus_costalis 1 3.70 yes yes no 0 0.5 0.5 0 combined
2016 August R69 2 humus Coleoptera Elateridae Eanus_costalis 1 8.19 yes yes no 0 0.5 0.5 0 combined
2016 August R69 2 humus Coleoptera Elateridae Liotrichus_affinis 1 6.26 yes yes no 0 0.5 0.5 0 combined
2016 August R69 2 humus Coleoptera Cantharidae Podistra_schoenherri 1 5.03 yes yes no 0 1 0 0 predator
2016 August R69 2 humus Diptera Empididae Phyllodromia_sp 1 0.21 yes yes no 0 1 0 0 predator
2016 August R69 2 humus Diptera Empididae Rhamphomyia_anomalina 1 0.25 yes yes no 0 1 0 0 predator
2016 August R69 2 humus Araneae Linyphiidae Gen_sp 2 0.18 yes yes no 0 1 0 0 predator
2016 August R69 2 humus Araneae Linyphiidae Tapinocyba_pallens 5 0.63 yes yes no 0 1 0 0 predator
2016 August R69 2 humus Myriapoda Lithobiidae Lithobius_curtipes 1 0.77 yes yes no 0 1 0 0 predator
2016 August R69 2 humus Coleoptera Staphylinidae Liogluta_micans 1 0.28 yes yes no 0 1 0 0 predator
2016 August R69 2 humus Coleoptera Staphylinidae Othius_lapidicola 1 0.53 yes yes no 0 1 0 0 predator
2016 August R69 2 humus Coleoptera Staphylinidae Oxypoda_annularis 1 0.08 yes yes no 0 1 0 0 predator
2016 August R69 2 humus Coleoptera Staphylinidae Oxypoda_annularis 1 0.10 yes yes no 0 1 0 0 predator
2016 August R69 2 humus Coleoptera Staphylinidae Oxypoda_annularis 1 0.10 yes yes no 0 1 0 0 predator
2016 August R69 2 humus Coleoptera Staphylinidae Oxypoda_skalitzkyi 1 0.08 yes yes no 0 1 0 0 predator
2016 August R69 2 mineral Coleoptera Staphylinidae Othius_lapidicola 1 0.72 yes yes no 0 1 0 0 predator
2016 August R69 3 humus Coleoptera Elateridae Eanus_costalis 1 1.60 yes yes no 0 0.5 0.5 0 combined
2016 August R69 3 humus Coleoptera Elateridae Eanus_costalis 1 3.95 yes yes no 0 0.5 0.5 0 combined
2016 August R69 3 humus Coleoptera Elateridae Eanus_costalis 1 7.85 yes yes no 0 0.5 0.5 0 combined
2016 August R69 3 humus Coleoptera Cantharidae Podistra_schoenherri 1 0.21 yes yes no 0 1 0 0 predator
2016 August R69 3 humus Coleoptera Cantharidae Podistra_schoenherri 1 2.39 yes yes no 0 1 0 0 predator
2016 August R69 3 humus Araneae Linyphiidae Tapinocyba_pallens 2 0.45 yes yes no 0 1 0 0 predator
2016 August R69 3 humus Myriapoda Lithobiidae Lithobius_curtipes 1 1.37 yes yes no 0 1 0 0 predator
2016 August R69 3 humus Coleoptera Staphylinidae Othius_subuliformis 1 0.52 yes yes no 0 1 0 0 predator
2016 August R69 3 humus Araneae Theridiidae Robertus_scoticus 1 0.13 yes yes no 0 1 0 0 predator
2016 August R69 4 humus Coleoptera Elateridae Eanus_costalis 1 1.18 yes yes no 0 0.5 0.5 0 combined
2016 August R69 4 humus Coleoptera Cantharidae Podistra_schoenherri 1 3.18 yes yes no 0 1 0 0 predator
2016 August R69 4 humus Araneae Gnaphosidae Gen_sp 1 0.68 yes yes no 0 1 0 0 predator
2016 August R69 4 humus Araneae Linyphiidae Gen_sp 1 0.30 yes yes no 0 1 0 0 predator
2016 August R69 4 humus Coleoptera Staphylinidae Liogluta_alpestris 1 0.08 yes yes no 0 1 0 0 predator

2016 August R69 4 humus Coleoptera Staphylinidae Liogluta_micans 1 0.29 yes yes no 0 1 0 0 predator
2016 August R69 4 humus Coleoptera Staphylinidae Mycetoporus_clavicornis 1 0.37 yes yes no 0 1 0 0 predator
2016 August R69 4 humus Coleoptera Staphylinidae Othius_sp 1 0.22 yes yes no 0 1 0 0 predator
2016 August R69 4 humus Coleoptera Staphylinidae Quedius_sp 1 0.93 yes yes no 0 1 0 0 predator
2016 August R69 4 humus Araneae Theridiidae Robertus_scoticus 1 0.05 yes yes no 0 1 0 0 predator
2016 August R69 5 humus Diptera Bolitophilidae Bolitophila_saundersii 1 1.09 no no no 0 0 0 1 fungivore
2016 August R69 5 humus Coleoptera Elateridae Eanus_costalis 1 4.20 yes yes no 0 0.5 0.5 0 combined
2016 August R69 5 humus Coleoptera Elateridae Eanus_costalis 1 11.50 yes yes yes 0 0.5 0.5 0 combined
2016 August R69 5 humus Araneae Linyphiidae Sisicus_apertus 1 0.09 yes yes no 0 1 0 0 predator
2016 August R69 5 humus Coleoptera Staphylinidae Liogluta_micans 1 0.31 yes yes no 0 1 0 0 predator
2016 August R69 5 mineral Araneae Linyphiidae Gen_sp 1 0.37 yes yes no 0 1 0 0 predator
2016 August R69 6 humus Coleoptera Elateridae Eanus_costalis 1 1.76 yes yes no 0 0.5 0.5 0 combined
2016 August R69 6 humus Coleoptera Elateridae Eanus_costalis 1 4.41 yes yes no 0 0.5 0.5 0 combined
2016 August R69 6 humus Araneae Linyphiidae Hilaira_herniosa 1 1.87 yes yes no 0 1 0 0 predator
2016 August R69 6 humus Araneae Linyphiidae Tapinocyba_pallens 1 0.15 yes yes no 0 1 0 0 predator
2016 August R69 6 humus Coleoptera Staphylinidae Othius_lapidicola 1 0.88 yes yes no 0 1 0 0 predator
2016 August R69 6 mineral Araneae Linyphiidae Gen_sp 1 0.37 yes yes no 0 1 0 0 predator
2016 August R69 7 humus Araneae Linyphiidae Gen_sp 1 0.50 yes yes no 0 1 0 0 predator
2016 August R69 7 humus Araneae Linyphiidae Hilaira_herniosa 1 0.08 yes yes no 0 1 0 0 predator
2016 August R69 7 humus Araneae Linyphiidae Oryphantes_angulatus 1 2.37 yes yes no 0 1 0 0 predator
2016 August R69 7 humus Coleoptera Staphylinidae Mycetoporus_clavicornis 1 0.19 yes yes no 0 1 0 0 predator
2016 August R69 7 humus Coleoptera Staphylinidae Oxypoda_annularis 1 0.04 yes yes no 0 1 0 0 predator
2016 August R69 7 humus Coleoptera Staphylinidae Oxypoda_annularis 1 0.05 yes yes no 0 1 0 0 predator
2016 August R69 7 humus Coleoptera Staphylinidae Oxypoda_annularis 1 0.06 yes yes no 0 1 0 0 predator
2016 August R69 7 humus Coleoptera Staphylinidae Oxypoda_annularis 1 0.12 yes yes no 0 1 0 0 predator
2016 August R69 7 mineral Araneae Linyphiidae Hilaira_herniosa 1 1.53 yes yes no 0 1 0 0 predator
2016 August R69 8 humus Araneae Linyphiidae Hilaira_herniosa 1 1.69 yes yes no 0 1 0 0 predator
2016 August R69 8 humus Coleoptera Staphylinidae Mniusa_incrassata 1 0.11 yes yes no 0 1 0 0 predator
2016 August R69 8 humus Coleoptera Staphylinidae Othius_subuliformis 1 0.15 yes yes no 0 1 0 0 predator
2016 August R69 8 humus Coleoptera Staphylinidae Oxypoda_elongatula 1 0.13 yes yes no 0 1 0 0 predator
2016 August R69 8 humus Coleoptera . . 1 11.49 . . . .
2016 August R69 8 mineral Araneae Linyphiidae Hilaira_herniosa 1 1.53 yes yes no 0 1 0 0 predator
2016 August R69 9 humus Coleoptera Elateridae Eanus_costalis 1 2.23 yes yes no 0 0.5 0.5 0 combined
2016 August R69 9 humus Araneae Lycosidae Pirata_Piratula_sp 1 0.00 yes yes no 0 1 0 0 predator
2016 August R69 9 humus Coleoptera Staphylinidae Othius_lapidicola 1 0.87 yes yes no 0 1 0 0 predator
2016 August R69 9 humus Coleoptera Staphylinidae Othius_subuliformis 1 0.48 yes yes no 0 1 0 0 predator
2016 August R69 10 humus Coleoptera Cantharidae Podistra_schoenherri 1 6.01 yes yes no 0 1 0 0 predator
2016 August R69 10 humus Diptera Empididae Phyllodromia_melanocephala 1 0.21 yes yes no 0 1 0 0 predator
2016 August R69 10 humus Diptera Empididae Phyllodromia_melanocephala 1 1.97 yes yes no 0 1 0 0 predator
2016 August R69 10 humus Araneae Linyphiidae Semljicola_latus 1 0.13 yes yes no 0 1 0 0 predator
2016 August R69 10 humus Araneae Linyphiidae Tapinocyba_pallens 1 0.11 yes yes no 0 1 0 0 predator
2016 August R69 10 humus Coleoptera Staphylinidae Othius_lapidicola 1 0.61 yes yes no 0 1 0 0 predator
2016 August R69 10 humus Coleoptera Staphylinidae Othius_lapidicola 1 0.75 yes yes no 0 1 0 0 predator
2016 August R69 10 humus Coleoptera Staphylinidae Oxypoda_annularis 1 0.08 yes yes no 0 1 0 0 predator
2016 August R69 10 humus Coleoptera Staphylinidae Oxypoda_annularis 1 0.08 yes yes no 0 1 0 0 predator
2016 August R69 10 humus Araneae Theridiidae Robertus_sp 1 0.02 yes yes no 0 1 0 0 predator
